# Building mass support for global pandemic recovery efforts in the United States

**DOI:** 10.1093/pnasnexus/pgac123

**Published:** 2022-08-16

**Authors:** Gautam Nair, Kyle Peyton

**Affiliations:** John F. Kennedy School of Government, Harvard University, Cambridge, MA 02138, USA; Institute for Humanities and Social Sciences, Australian Catholic University, Melbourne, VIC 3002, Australia

**Keywords:** COVID-19, vaccine nationalism, international cooperation, redistribution

## Abstract

Containing the COVID-19 pandemic will confer global benefits that greatly exceed the costs but effective solutions require the redistribution of vaccines, technology, and other scarce resources from high-income to low-income countries. The United States has played a central role in coordinating responses to previous global health challenges, and its policy choices in the current pandemic will have a far-reaching impact on the rest of the world. Yet little is known about domestic support for international recovery efforts. We use a series of conjoint and persuasive messaging experiments, fielded on two national surveys of the US adult population (*N* = 5,965), to study mass support for international redistribution. We find clear evidence that the general population strongly supports allocating vaccines to own-country recipients before others. But despite this “vaccine nationalism,” Americans are also willing to support the US government playing a major role in global pandemic recovery efforts, provided policymakers forge international agreements that ensure moderate domestic costs, burden-sharing with other countries, and priority for certain types of resources, such as domestically manufactured vaccines and patent buyouts. Finally, we test five different persuasive messaging strategies and find that emphasizing the relatively low costs and large economic benefits of global vaccination is the most promising means of increasing domestic support for international redistribution. Overall, our results demonstrate that policymakers can secure broad public support for costly international cooperation by crafting responses aligned with the economic interests of the United States.

Significance Statement Wealthy countries such as the United States will play a pivotal role in the global recovery from the COVID-19 pandemic. We conduct large-scale surveys and experiments in the United States to illuminate pathways for building domestic support for redistributing scarce vaccines, technology, and other resources across national borders. Although Americans strongly favor vaccinating own-country recipients before others, we find that policymakers can secure broad support for international recovery efforts by ensuring moderate domestic costs, increasing burden-sharing with other countries, prioritizing redistribution schemes that advance US interests, and highlighting the substantial economic benefits of global vaccination in political communications with the mass public.

Vaccinating the world against COVID-19 will save lives, prevent the emergence of new disease variants, and spur economic growth, generating global economic benefits that vastly exceed the costs (1,2). Securing these benefits and reducing international inequalities in access to COVID-19 vaccines (3–5) will require transferring vaccines and other scarce resources from rich countries to poor countries, but little is known about mass attitudes on such redistributive policies. Here, we leverage a series of novel experiments embedded in large-scale national surveys of the US population to examine mass attitudes towards global pandemic recovery efforts. Over the last seven decades, the United States has been a critical actor in the creation and maintenance of international institutions, a significant provider of funding for global health initiatives (6–8), and a major center of pharmaceutical technology, development, and production (9). More recently, however, US participation in international institutions and the costs it bears in providing global collective goods have become politically salient and intensely debated domestic policy issues (10,11). The backing of the United States for the international distribution of COVID-19 vaccines, technology, and other assistance have been contentious issues among policy makers, political elites, and the scientific community. Some favor extensive vaccine sharing, substantial financial commitments, and interventions such as intellectual property waivers (12, 13), while others strongly oppose such policies (14). A better understanding of mass preferences can help policymakers craft coalitions that durably support international cooperation in the context of the ongoing pandemic, as well as future global public health challenges.

To date, we know relatively little about preferences among the general public, such as whether “vaccine nationalism” reflects generic opposition to international redistribution, or how different political messaging strategies and agreements to redistribute scarce resources across borders could foster (or erode) domestic support for US cooperation in global pandemic recovery efforts. Our research represents a significant departure from prior work on international redistribution of foreign aid, in general (15–21), and COVID-19 vaccine allocation mechanisms, in particular (22–24). First, we examine the causal effects that multidimensional characteristics of potential vaccine recipients have on public support for the distribution of vaccine across borders. Second, building on prior research (25), we quantify how key features of cooperative international regimes—such as domestic costs, burden-sharing with other countries, and the form of redistributive transfers—shape policy attitudes in the mass public. Third, we experimentally test the efficacy of various persuasive messaging strategies for building public support for international cooperation using a high-quality probability sample of the mass public that incorporates measures of both stated preferences and behavior.

Our data come from two large surveys of the US adult population (combined *N* = 5,965). The first survey, conducted on the Lucid platform (hereafter, Survey 1), was fielded in 2021 April (*N* = 1,751). The second survey, conducted on the NORC/Amerispeak panel (hereafter, Survey 2), was fielded between 2021 September 8 and 2021 October 15 (*N* = 4,214). Survey 1 was a quota sample of adults with demographic characteristics (e.g. region, race/ethnicity) matched to US census margins, and Survey 2 was a probability sample based on a sampling frame of US adults maintained by NORC/Amerispeak. The Supplementary Material Appendix provides a detailed description of each survey, comparisons to population benchmarks, the sampling procedures and question wordings, and the experiments that were embedded within each (SM Sections S1 and S2). Both survey samples were similar to each other and broadly representative of the US adult population on demographic characteristics (Table S2). We report unweighted estimates here and provide estimates after applying survey weights in Supplementary Material Appendix S3.1 (Figures S3 to S9, S15 to S20, and Table S3); none of the minor differences between our weighted and unweighted estimates are statistically significant or substantively meaningful.

We focus on several related dimensions of public support for global pandemic recovery efforts, using four different preregistered experimental designs. Our first three rely on conjoint survey experiments (26), which have been widely used in the social and behavioral sciences to study preferences on complex political topics, such as immigration (27), global climate change mitigation (25), the European debt crisis (28), and natural disaster responses (29). A key advantage of conjoint experiments is that they make trade-offs salient in the minds of respondents, and the randomization of multidimensional treatments allows the researcher to identify the marginal effects of various components of interest, as well as their relative importance. Our first conjoint experiment (hereafter, vaccine recipient experiment) quantifies the importance of “vaccine nationalism”—a preference for allocating vaccines to own-country recipients over others—in a multidimensional choice context that incorporates other relevant features such as the risks of exposure to, and severe illness from, COVID-19. Second, we use two conjoint experiments (hereafter, international agreement experiments), each on an independent sample of the US adult population, to quantify the relative effects that various policy design features have on support for US participation in global pandemic recovery efforts. These features, though hypothetical, are based on potential cooperative agreements between countries that have been widely discussed in the public, scientific, and international policy domains (1,2, 30). Finally, we quantify the effects that five different types of persuasive messaging strategies have on public support for these efforts using a randomized experiment (hereafter, persuasive messaging experiment) conducted on a probability sample of more than 4,000 Americans that incorporates both attitudinal and behavioral measures of preferences.

## Do Americans support distributing vaccines to individuals in other countries?

Amidst salient tensions between values and self-interest in the international context of the COVID-19 vaccine allocations, proponents of ethical frameworks for allocating vaccines such as the “Fair Priority Model” (31) argue that these frameworks place strong normative constraints on the extent, to which relatively rich countries should prioritize vaccinating their own citizens over those in other countries.

The mass public, however, does not necessarily share these same ethical principles. For example, in Survey 1 (2021 April), 67% believed the US “should ensure that there are enough vaccines for people in the U.S., even if it means people in developing countries need to wait longer to get vaccines.” Those asked the same question in a representative sample of US adults surveyed by Pew Research Center in 2021 February expressed nearly identical views, suggesting widespread “vaccine nationalism” (i.e. using nationality as an important vaccine allocation criteria) in the United States (32).^a^

Though informative, standard survey questions do not force respondents to evaluate the relative importance of a potential vaccine recipient’s nationality against other ethically relevant criteria such as individual’s risk of exposure to COVID-19 and the potential for harm if infected. Therefore, it is unclear whether Americans prioritize own-country nationality over ethically relevant factors such as medical risk. For example, does own-country nationality have a stronger effect on Americans’ vaccine allocation preferences than age, occupation, or risk of exposure to COVID-19?

To shed light on such questions, we designed a conjoint experiment (embedded in Survey 1, fielded in 2021 April) that presented a sample of 1,751 Americans with five different pairings of potential vaccine recipients, each with randomized information about their country of origin. At the time, more than 40% of the US adult population had received at least one dose of a COVID-19 vaccine. Country of origin was randomized to be the United States or one of eight other countries (Australia, Brazil, Canada, China, India, Nigeria, Pakistan, or South Africa) with varying access to COVID-19 vaccines and cultural and diplomatic proximity to the United States.

In addition to country of origin, respondents were presented with the sex (randomized to be male or female) of each potential recipient as well as five ethically relevant vaccine allocation criteria: (1) risk of exposure to COVID-19 (low, moderate, or high); (2) risk of serious illness if infected (low, moderate, or high); (3) occupation group (a nonessential worker or one of four essential worker categories); (4) age group (ranging from 18 through 24 to 75+); and (5) whether they can work from home. These five dimensions represent widely used within-country vaccine allocation criteria and recent conjoint experiments have demonstrated their ethical relevance to domestic populations across the globe (24).

We used the standard conjoint experiment design (26), in which all attributes are independently and uniformly randomized with levels in each attribute shown with equal probability. In our conjoint experiment, each of the 1,751 respondents made five binary choices over potential vaccine recipients (a total of 17,510 pairwise comparisons) that varied independently across country of origin and the other attributes (see Supplementary Material Appendix S1.2 for design details; S4 for preregistration).

### Results

We focus here on the subset of randomized profiles that forced respondents to make pairwise comparisons between a potential vaccine recipient in their own country (the United States) and another country (*N* = 3,418). Results for the full sample of pairwise comparisons (including those between two US recipients) are provided in Supplementary Material Appendix S3.1.

**Fig. 1.**
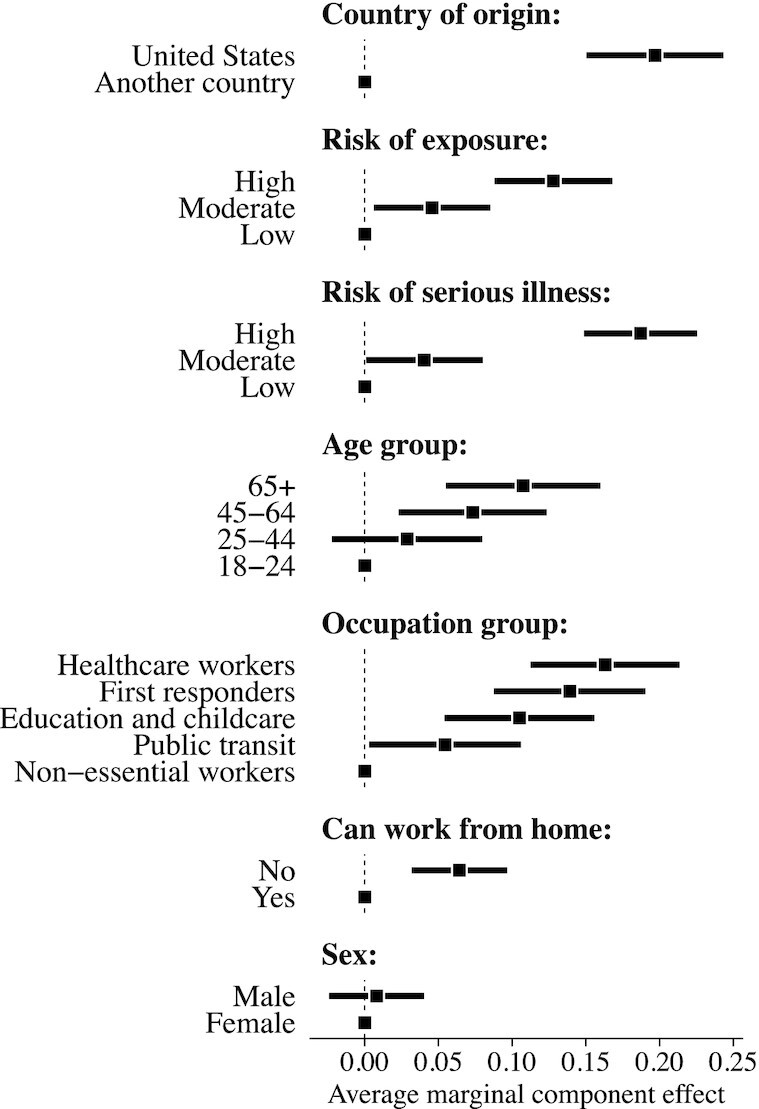
Effects of randomly assigned background characteristics on probability of selecting individual as potential vaccine recipient. Point estimates and 95% CIs estimated via ordinary least squares (OLS) regression with robust SEs clustered at respondent level to correct for within-respondent clustering. Source: Lucid survey of US adults fielded in 2021 April (*N* = 1,751 respondents × 5 pairings × 2 agreements per pair = 17,510 observations). The estimation sample is restricted to the subset of randomized profiles with pairwise comparisons between the United States and another country (*N* = 3,418 observations). See Supplementary Material, Figures S3 and S4 for full sample results and comparisons to estimates with survey weights.

Figure 1 shows the estimated effects of each randomized attribute level against a baseline reference category (denoted by points without CIs). We see clear evidence that Americans prefer allocating vaccines to own-country recipients, independent of all other potentially relevant criteria. The estimated effect of US country of origin corresponds to an increase in the probability of selecting that individual, relative to someone from another country, of 0.20 ($\widehat{\text{SE}} = 0.02, P \lt 0.01$). We find strong evidence of bias against potential vaccine recipients from all other countries, ranging from −0.26 ($\widehat{\text{SE}} = 0.04, P \lt 0.01$) for China to −0.13 ($\widehat{\text{SE}} = 0.04, P \lt 0.01$) for South Africa (see Supplementary Material Appendix Figures S3 and S4).

Notably, this experiment was fielded during a period, in which vaccines were widely available in the United States. We do not find evidence of significant causal interactions between country of origin and other randomized features (see Supplementary Material Appendix Section S3.2 for these analyses). Instead, we find that the conditional effects of being outside the US are uniformly negative and statistically significant across all other randomized characteristics (see Figures S10 to S12). We also find consistent evidence of effect heterogeneity for the country of origin attribute as a function of nationalistic attitudes: Estimated average marginal component effects (AMCEs) for “nationalists” are significantly larger than “non-nationalists” across multiple measures of nationalism. This demonstrates that, all else equal, nationalism (33) predicts greater bias against potential vaccine recipients from other countries (see Appendix S3.5, Figures S21 to S26).^b^ We do not find systematic effect heterogeneity as a function of other preregistered background covariates.^c^

In contrast to ethical frameworks that argue that nationality should not be prioritized, we find strong evidence of nationalistic bias in favor of vaccinating Americans first. However, this bias can be mitigated under some circumstances, such as wide disparities in medical risk (see Supplementary Material Appendix Figure S12). Averaging across all pairwise comparisons between US and non-US recipients, Americans with a low risk of serious illness were selected with probability 0.52, whereas non-Americans with a high risk were selected with a similar probability 0.49. The pooled country of origin effect is of similar magnitude to the 0.19 ($\widehat{\text{SE}} = 0.02, P\lt 0.01$) effect for a person with a high, relative to low, risk of serious illness, if infected with COVID-19. Thus, while Americans clearly prioritize own-country recipients over other-country recipients, the mass public is not fundamentally opposed to international vaccine redistribution (34).^d^ We now turn to understanding the specific conditions and institutional arrangements that are most conducive to securing support for such policies.

## How does policy design affect domestic support for international cooperation?

Mitigating the tremendous human and economic costs of the COVID-19 pandemic, as well as future pandemics, is a significant global policy challenge. While the benefits of international cooperation greatly outweigh the costs, conservative estimates suggest the price of vaccination alone exceeds ${\$}$50 billion (2). In the United States, as well as other countries with elections, policy makers may be reluctant to make substantial contributions to these international costs if they anticipate domestic opposition. As demonstrated in the previous section, for example, there exist strong political incentives for electorally minded politicians to ensure their domestic populations have priority access to vaccinations.

Global pandemic recovery efforts are therefore not unlike international climate change initiatives, which also require both international cooperation and strong domestic support to be politically sustainable (25, 35, 36). Prior research in this domain demonstrates that institutional design features—such as the domestic costs, enforcement mechanisms, and the participation of other countries—can have strong effects on public support for international cooperation (25). Building on this work, we designed two experiments to examine how key institutional design features affect domestic support for international cooperation on global pandemic recovery agreements.

The first experiment, embedded in Survey 1 (*N* = 1,751; 2021 April), examines whether public support for international cooperation on COVID-19 vaccine redistribution agreements is affected by four features that condition support for global climate change agreements: domestic costs and their distribution across countries, participation by other countries, and enforcement mechanisms. To this list, we add three potentially important pandemic-specific features: how the benefits (i.e. vaccines) will be distributed across countries, and whether these agreements mandate sharing of vaccine technology or impose restrictions on external supply agreements. As with the conjoint experiment from the previous section, respondents were presented with four different pairings of hypothetical agreements between countries, each with randomized information about the seven features of study (this conjoint was presented before or after the vaccine recipient conjoint, in randomized order; see Supplementary Material Section S1.3 for design details; S5 for preregistration).

The second experiment, embedded in Survey 2 (*N* = 4,214; 2021 September/October), narrows the focus on five salient features of potential pandemic recovery efforts: the total costs and proportion that would be paid by the United States, the specific form of redistribution that will occur, the criteria for selecting beneficiary countries, and the duration of the agreement. This design replicates the first experiment along key features of costs, as well as burden-sharing with other countries (i.e. the proportion of costs paid by the United States), but also broadens the scope beyond vaccines to include other types of redistribution, such as vaccine production technology and economic aid. Here, respondents were presented with two different pairings of hypothetical agreements, each with randomized information about the four different features (see Supplementary Material Appendix Section S2.2 for design details; S6 for preregistration).

### Results

**Fig. 2.**
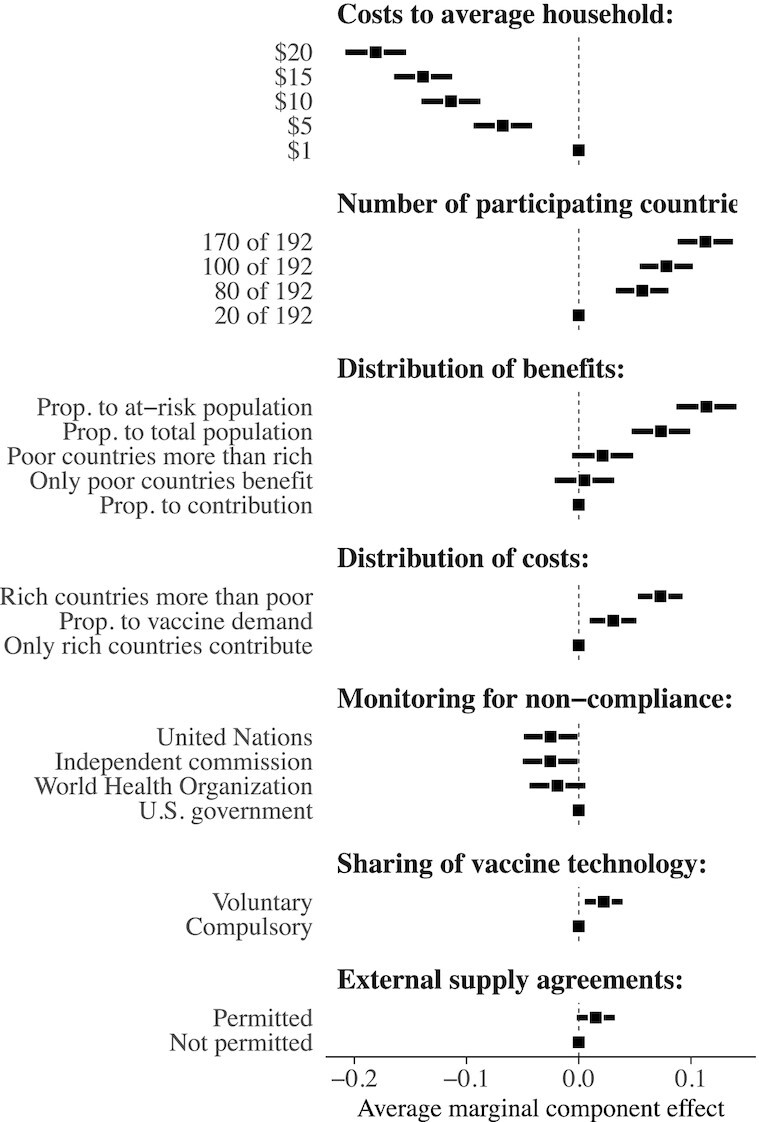
Average marginal component effects of randomly assigned design features on probability of selecting an agreement (1/0). Point estimates and 95% CIs estimated via OLS regression with robust SEs clustered at respondent level to correct for within-respondent clustering. Source: Lucid survey of US adults fielded in 2021 April (*N* = 1,751 respondents × 4 pairings × 2 agreements per pair = 14,008 observations). See Supplementary Material Figures S5 and S6 for comparisons to estimates with survey weights.

Figure 2 shows the estimated effects that different agreement features have on average public support, with the interpretation of each estimate relative to the reference category (denoted by dots without CIs). These results demonstrate that the potential costs to US households have the strongest effect on public support for international cooperation on global vaccination efforts. The estimated effect of agreements that entail costs of ${\$}$20 per household—versus counterfactual agreements that cost ${\$}$1—is a decrease in the probability of support by 0.18 ($\widehat{\text{SE}} = 0.01, P \lt 0.01$). On the lower end, even agreements with a relatively modest cost of ${\$}$5 per household cause a decrease in support by 0.07 ($\widehat{\text{SE}} = 0.01, P \lt 0.01$). For context, there are approximately 120 million households in the United States, so a cost of ${\$}$20 per household—totalling approximately ${\$}$2.4 billion—would fall far short of the estimated ${\$}$50 billion required to vaccinate 70% of the world’s population.

Though lower costs are clearly preferable to higher costs, our results also demonstrate that prices are not the only determinate of public support for global vaccination efforts. We find clear evidence that crafting agreements that ensure broad participation and burden-sharing among countries significantly increases public support for international cooperation. For example, increasing the number of participating countries from 20 to 170 causes an increase in support of approximately 11 percentage points. Similarly, agreements that require rich countries to contribute more than poor countries cause a roughly 7 percentage point increase in public support relative to those that place the entire burden on rich countries. These results are consistent with prior work on climate change agreements (25), which shows that accounting for the public’s opposition to perceived free-riding and underlying fairness norms can help to secure mass support for international cooperation.^e^

In the vaccine recipient experiment, we found that medical risk is a key determinant of how respondents choose to hypothetically allocate vaccines across individuals. These results are reflected in respondent’s preferences in the policy setting of the institutional agreements experiment. We find here that changing the potential agreement to instead allocate vaccines in proportion to the size of the at-risk population causes an 11 percentage point increase in public support relative to a market-oriented “ability to pay” mechanism ($\widehat{\text{AMCE}} = 0.11, \widehat{\text{SE}} = 0.01, P \lt 0.01$). Whether these potential agreements mandate sharing of vaccine technology, or impose restrictions on external supply agreements, are not major determinants of public support.

Unlike prior work on climate change agreements (25), we do not find evidence that the specific institution responsible for enforcing the terms of a potential agreement is a major determinant of public support in general. We do, however, find evidence of effect heterogeneity for this attribute as a function of nationalistic attitudes: estimated AMCEs are significantly more negative among respondents with above-median levels of nationalism.^f^ We also find evidence that levels of nationalism are prognostic of stronger preference for low-cost agreements, vaccine allocations in proportion to a country’s financial contribution, and prioritization of domestic manufacturing and patents (see Supplementary Material Appendix Figures S39 to S44).^g^

**Fig. 3.**
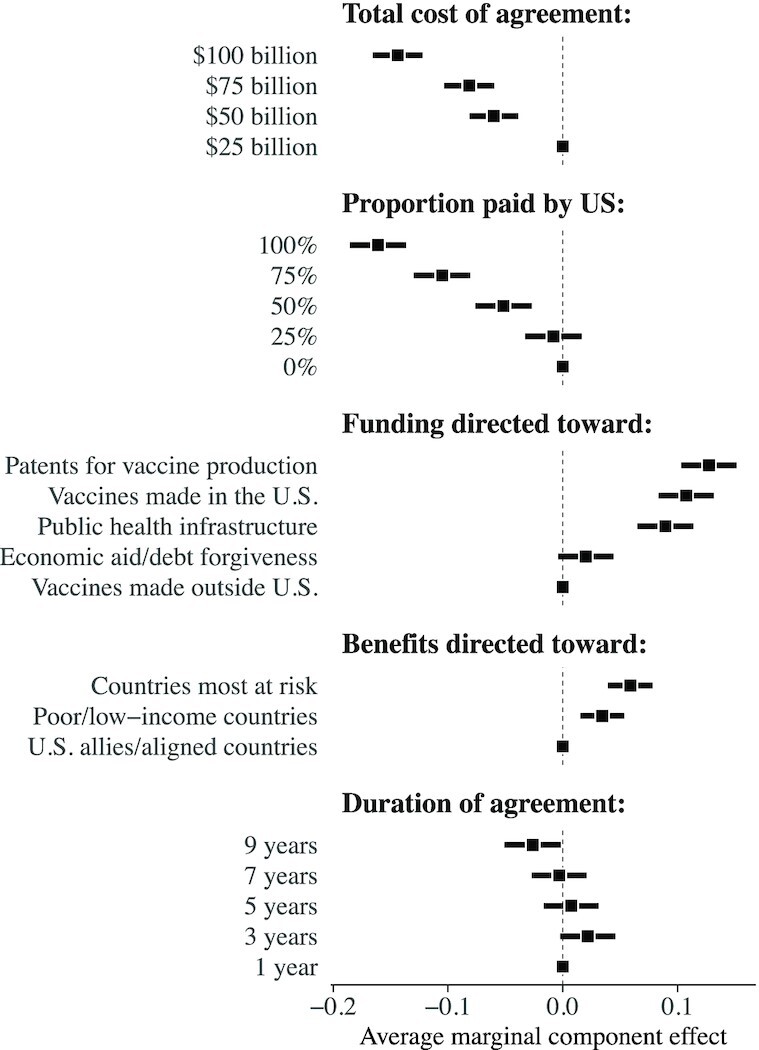
Average marginal component effects of randomly assigned design features on probability of selecting an agreement. Point estimates and 95% CIs estimated via OLS regression with robust SEs clustered at respondent level to correct for within-respondent clustering. Source: NORC/Amerispeak survey of US adults fielded in 2021 September/October (*N* = 4,214 respondents × 2 pairings × 2 agreements per pair = 16,856 observations). See Supplementary Material, Figures S7 and S8 for comparisons to estimates with survey weights.

While vaccines are a vital component in the fight against pandemics, international agreements may prioritize other types of redistribution, including vaccine production technology, intellectual property, and the financing of public health infrastructure (1, 37). These latter types of assistance may be just as important to global recovery efforts over the longer term. Our conjoint experiment embedded in Survey 2, therefore, broadened the focus to examine public support for potentially more expansive international agreements, which distribute different types of aid and range in duration from 1 to 9 y. Figure 3 shows the estimated effects relative to each reference category (dots without CIs).

Among these more expansive agreements, we find that total costs and burden-sharing with other countries have the strongest effects on public support. The estimated effect for agreements totalling ${\$}$50 billion—the approximate cost to vaccinate 70% of the world’s population—corresponds to a reduction in the probability of support by 0.06 ($\widehat{\text{SE}} = 0.01, P \lt 0.01$), relative to agreements that cost ${\$}$25 billion (baseline selection probability of 0.57). On the higher end, agreements that cost ${\$}$100 billion cause a decrease in the probability of support by 0.14 ($\widehat{\text{SE}} = 0.01, P \lt 0.01$). Importantly, the general public is broadly opposed to agreements in which the US funds the majority of global costs. Averaging across all pairwise comparisons, we find that agreements in which the United States paid 75% of the total costs were rejected 54% of the time and those in which the United States funded 100% were rejected 60% of the time.

Given the independent randomization across features, we can directly estimate how the implied burden to the United States (i.e. the causal interaction between total costs × proportion paid) affects domestic support for these agreements. On the low end, we find that agreements involving a ${\$}$6.25 billion burden (i.e. 25% of a ${\$}$25 billion total) are chosen with probability 0.61 ($\widehat{\text{SE}} = 0.02$), whereas those involving a ${\$}$100 billion burden are chosen with probability 0.32 ($\widehat{\text{SE}} = 0.02$). That is, all else equal, moving from ${\$}$6.25 billion to ${\$}$100 billion causes a decrease of nearly 30 percentage points ($0.61-0.32 = 0.29, \widehat{\text{SE}} = 0.02, P \lt 0.01$).

Figure 3 also demonstrates that the specific form that redistribution takes may be nearly as important as the total costs of global pandemic recovery efforts. International agreements that direct funding towards the purchase of patents for vaccine production have the largest effect, corresponding to a reduction in the probability of support by 0.13 ($\widehat{\text{SE}} = 0.01, P \lt 0.01$), relative to those that simply purchase and redistribute vaccines manufactured outside the United States, such as the widely distributed AstraZeneca and Sinovac vaccines. Overall, directing funds towards the purchase of vaccines made outside the United States (baseline selection probability of 0.43) or economic aid (0.45) are net unpopular. Relative to these options, respondents prefer agreements that direct funds towards financing public health infrastructure in low-income countries (0.52), purchasing vaccines made in the United States (0.54), or patent buyouts (0.56).

Although public support does not appear to be sensitive to the duration of potential agreements, attempts to use the COVID-19 crisis as an instrument of so-called “vaccine diplomacy” (38) can cause significant reductions in public support. On average, we find agreements that allocate benefits in proportion to need by prioritizing countries most at risk of outbreaks (also the most preferred distribution mechanism in Figure 2) are selected with probability 0.53. However, agreements that give preferential treatment to US allies and aligned countries are disfavored and, by comparison to the preferred need-based mechanism, selected with probability 0.47 ($\widehat{\text{AMCE}} = 0.06, \widehat{\text{SE}} = 0.01, P \lt 0.01$). These effects are largest among Democrats and those with below-median scores on measures of nationalism (see Supplementary Material Appendix S3.7 for analyses of effect heterogeneity by different measures of nationalism and “patriotism” (Figures S57 to S62), altruism (Figures S63 and S64), partisanship (Figures S65 and S66), and ideology (Figures S67 and S68)).

Overall, these results are consistent with prior research demonstrating that Americans prefer health-related assistance over other types of foreign aid (19,39). We also find strong evidence that agreements which prioritize innovative policies such as patent buyouts can generate large increases in public support, potentially enabling a bridge between fairness concerns and market incentives. On the other hand, a call for the United States to fund manufacturing facilities abroad is unlikely to win significant domestic support, despite the potential advantages of a globally diversified supply base.

Policymakers may therefore be able to ensure broad public support for international cooperation by relying more heavily on domestic production and incentives, which may also secure support from US interest groups. For example, consider a 5-y agreement in which the United States funds 50% of ${\$}$50 billion (implied burden of ${\$}$25 billion) to provide aid to countries most at risk for outbreaks. We find that such an agreement would win support in 62% of pairings if foreign aid is used to purchase patents for vaccine production. An agreement with the same cost features that instead directs aid toward the purchase of vaccines made in the United States wins support in 60% of pairings. However, when aid is instead used to fund the purchase of vaccines made outside the United States, we find that domestic support drops to 49%.

## Can persuasive messaging increase public support for global pandemic recovery efforts?

Although vaccine nationalism in the general population may be a constraint on the redistribution of vaccines under some circumstances, the results presented in the previous sections demonstrate that it is not an insurmountable barrier. That is, Americans’ preferences for vaccinating their own citizens before others does not imply domestic opposition to cooperative international agreements that redistribute scarce resources across national borders. It may therefore be feasible for decision-makers to increase support for international cooperation by emphasizing the potential benefits of the United States leading pandemic recovery efforts and becoming the world’s “arsenal of vaccines” (40, 41). Here, we examine the potential for persuasion to increase support for international cooperation using a randomized experiment, embedded in Survey 2, that tested five different messaging strategies on a large, nationally representative sample of US adults (*N* = 4,214; 2021 September/October).

These messages provided information on (1) the large economic benefits of increased growth and trade relative to the low costs of global vaccination efforts (“Economic Benefits” treatment); (2) the importance of global vaccine coverage in preventing the emergence of new virus variants (“Mutation Risk”); (3) the past success of US efforts in leading international efforts to combat infectious diseases like AIDS (“Past Success”); (4) the use of vaccine exports as a tool of strategic diplomacy by rivals such as China and Russia (“Vaccine Diplomacy”); or (5) inequality in access to vaccines between rich and poor countries (“Global Inequality”).

Each was selected to probe the influence of a theoretically distinct mechanism of persuasion. Variants of these appeals were also politically salient when the survey was fielded, and had appeared in US and international media outlets. The “Economic Benefits” message focuses on economic interests (42). “Mutation Risk” emphasizes threats to health (43). The “Global Inequality” message informs citizens about the need for vaccines abroad (44). “Past Success” seeks to ameliorate skepticism about the effectiveness of foreign and low trust in government (45). The “Vaccine Diplomacy” message informs citizens about the diplomatic and international relations dimensions of pandemic assistance by US rivals like China and Russia (38).

Each of the five treatments first provided relevant factual information about a different global challenge created by the pandemic, and then proposed US coordination of international efforts as a promising solution. For example, the “global inequality” treatment provided respondents with information about the unequal distribution of vaccines between rich and poor countries (i.e. less than 1% of the doses administered worldwide had been in poor countries). A control condition provided respondents with no information, and respondents were assigned to one of the six total conditions using simple random assignment (see Supplementary Material Appendix S2.3 for design details and the text used in each treatment arm; S7 for preregistration).

### Results

We measure overall support for global pandemic recovery efforts using a preregistered summary index that incorporates measures of both stated preferences and behavior: (1) respondents’ stated preferences about the share that the US government should contribute to the estimated ${\$}$50 billion in global vaccination costs; (2) willingness to engage in political action by signing a petition for Congress to increase spending on COVID-19 assistance abroad; and (3) willingness to make a charitable contribution to COVAX from a bonus payment of ${\$}$10. The first captures stated support for US spending on pandemic-specific foreign aid, the second willingness to engage in relatively low-cost political behavior, and the third altruistic behavior via charitable donations.

Among those assigned to the control group, the median respondent supported the United States contributing about 30% of the total funds required to vaccinate the world (implied burden of ${\$}$15 billion), 38% expressed a willingness to sign the petition, and the median respondent donated 50% of their bonus payment ($5) to COVAX.^h^ Overall, we find evidence of small persuasive effects, as measured by our summary index. Three of the five persuasive messages caused statistically significant increases in support for global vaccination efforts: Economic Benefits ($d = 0.16, \widehat{\text{SE}} = 0.04, P \lt 0.01$), Mutation Risk ($d = 0.12, \widehat{\text{SE}} = 0.04, P = 0.01$), and Global Inequality ($d = 0.11, \widehat{\text{SE}} = 0.04, P = 0.01$). The estimated effects of the other two strategies were even smaller, and not statistically distinguishable from zero at the conventional threshold: Vaccine Diplomacy ($d = 0.09, \widehat{\text{SE}} = 0.05, P = 0.05$) and Past Success ($d = 0.08, \widehat{\text{SE}} = 0.04, P = 0.07$).

**Fig. 4.**
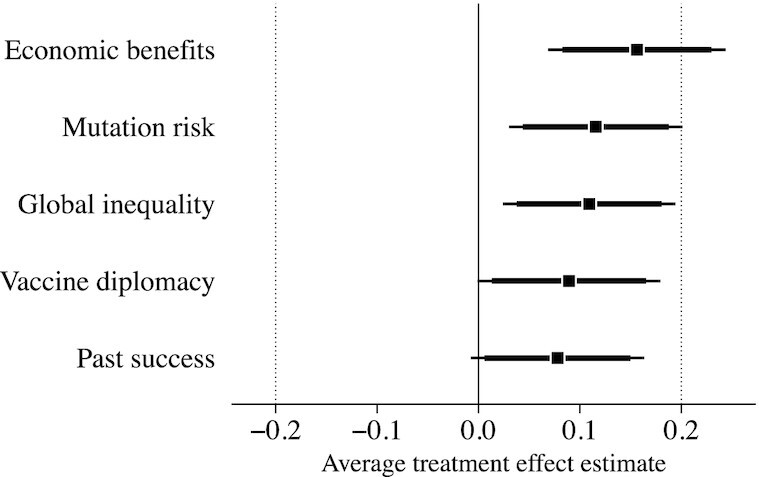
Estimated treatment effects of persuasive messaging strategies on support for global vaccination efforts. Thick horizontal lines denote 90% CIs and thin lines denote 95% CIs. Dotted vertical lines denote an MOE of ±0.20 standard units. All point estimates (and CIs) from covariate-adjusted linear regression estimator with CIs based on HC2 robust SEs. Pre-registered pretreatment covariates: age, sex, education level, race/ethnicity, region, employment status, household income, partisanship, conservatism, altruism, and nationalism/patriotism. See Supplementary Material Figure S9 for estimated effects on each index component, and Table S3 for point estimates and SEs with and without covariate adjustment.

To facilitate interpretation of effect sizes and comparisons, Figure 4 shows these estimates with both 90% and 95% CIs, as well as a margin of equivalence (MOE) bound of ±0.20 standard units. This MOE corresponds to one-fifth of 1 SD on the outcome index, and when the 90% CI for an estimated effect is contained inside the MOE, the null hypothesis of nonequivalence is rejected in favor of equivalence. We can, therefore, conclude that an estimated effect is distinguishable from zero, when the 95% CI excludes zero, but “minimal” (i.e. statistically equivalent to ±0.20 standard units), when the estimated 90% CI falls within the MOE (46–48). For substantive context, an effect of 0.20 standard units is about one-fifth the size of the baseline difference between Republicans and Democrats in the control group.

As Figure 4 demonstrates, four of the five messaging strategies had minimal persuasive effects. The one exception was the treatment emphasizing the potential economic benefits relative to the costs of global vaccination efforts, which caused a statistically significant increase in support of 0.16 standard units that cannot be declared minimal under the chosen MOE (the 90% interval includes 0.20). These results are consistent with prior work demonstrating that informing Americans of the relatively low costs, and the potential economic benefits, of foreign aid can increase support for international transfers in general (18,49). We examine effect heterogeneity as a function of preregistered background characteristics (including nationalism and partisanship) using a machine learning algorithm that automates the search for treatment–covariate interactions in Supplementary Material Appendix Section S3.8 (Figures S69 to S75). Consistent with prior work on political persuasion (50, 51), we do not find compelling evidence that the small effects identified here varied significantly across subgroups.

## Discussion

Mitigating the spread of COVID-19 will save lives, prevent the emergence of new variants, and accelerate trade and economic growth across the world. By increasing the supply of vaccines and related public health infrastructure in low-income countries, these benefits can be achieved at costs that are a fraction of domestic expenditures on pandemic response in wealthy nations. While these costs are trivial relative to the benefits, they are substantial in absolute terms, especially given the growing demand for booster shots and the need to develop new vaccines that provide protection against emerging variants of concern. This study used a series of experiments embedded in large-scale surveys of the US adult population to examine Americans’ willingness to support global pandemic recovery efforts. We sought to answer three salient questions. First, do Americans support the redistribution of vaccines to people living abroad? Second, does the design of policies and institutions matter in building mass support for costly international cooperation in the context of the pandemic, and which design features are most important? Third, what types of communication strategies are most effective in persuading citizens to back international efforts?

We found that “vaccine nationalism” in public policy is consistent with a broad consensus in the mass public: the vast majority of Americans prefer policies that prioritize US residents over non-US residents in the allocation of vaccines. But while Americans’ bias towards fellow citizens cannot be entirely eliminated, it shrinks when candidates for vaccines abroad face substantially higher health risks. Moreover, the mass public is willing to support the US government allocating significant expenditures towards cooperative international agreements that redistribute both COVID-19 vaccines and more generic forms of pandemic related foreign aid. We showed that decision-makers can bolster this support through policy choices and institutional design. One way of building mass support for international recovery efforts is to lower domestic costs by ensuring broader participation and burden-sharing on the part of other countries. Another is for decision-makers to focus recovery aid specifically on health-related interventions, such as patent buyouts and domestic vaccine manufacturing, that generate obvious benefits to the United States (while simultaneously eliciting the support of domestic interest groups). One of the novel theoretical insights that emerges from our analysis is that decision-makers can wield “economic nationalism” as a means of countering “vaccine nationalism” and concerns about the cost of contributions towards international cooperation. These policies may be second-best solutions from an economic and global justice perspective, but may prove more sustainable over the long term from a political standpoint and are relevant to both the present pandemic and future public health challenges. Finally, reframing US contributions to global pandemic recovery efforts from a purely humanitarian endeavor to one that serves the material and economic interests of the United States can reinforce domestic support for international cooperation.

## Supplementary Material

pgac123_Supplemental_File

## Data Availability

This research was approved by the Human Subjects Committee (HSC) at Harvard University (IRB Protocols #IRB21-0946 and #IRB20-2152). Consent to participate was obtained online at the beginning of each survey. All replication data and code have been deposited at Harvard Dataverse and are available for download at https://doi.org/10.7910/DVN/FYYJD9.

## Footnotes

a We are agnostic about whether this preference originates in individual self-interest or pro-social concerns; our claim is simply that national origin is an important predictor of distributive preferences.

b Nationalism is measured in this survey using respondents’ level of agreement with the statement “For the most part, the United States is better than any other country in the world.” Van Bavel et al. (33) distinguish between what they term “national identity (NI)” or national pride/patriotism and “national narcissism (NN)” or national superiority; the latter is more strongly associated with outgroup prejudice and may be better approximated by the measure used here.

c See Supplementary Material Appendix S1.1 and S2.1 for a description of pretreatment measures, and Figures S1 and S2 for correlations among these measures. See Supplementary Material Appendix S3.5 and Figures S21 to S38 for analyses of effect heterogeneity by vaccine nationalism, nationalism, cosmopolitanism, altruism, reciprocity, vaccination status, partisanship, and political ideology.

d While the forced-choice setup of the conjoint reflects an environment of scarcity and the distributive nature of this policy domain, additional ordinal measures of preferences also indicate no fundamental opposition to international transfers (see Supplementary Material Appendix S3.4, and Figures S15 to S20).

e In line with this work, we also find that the effect of burden sharing on agreement support is higher for individuals who score highly on measures of reciprocity (see Supplementary Material Figures S49 and S50).

f See Supplementary Material Appendix S1.1 fo a description of the different measures of nationalism and Figures S39 to S44 for analyses of effect heterogeneity across these measures.

g We find some evidence of heterogeneity by partisanship: Republicans prefer international agreements monitored by the US government or an independent commission over the United Nations or the World Health Organization (WHO), while Democrats favor monitoring by the WHO (see Supplementary Material Figures S53 and S54). Similar results hold for differences between self-identified liberals and conservatives (see Supplementary Material Figures S55 and S56). The Supplementary Material Appendix reports additional analyses of effect heterogeneity by cosmopolitanism (Supplementary Material Figures S45 and S46), altruism (Supplementary Material Figures S47 and S48), and reciprocity (Supplementary Material Figures S49 and S50).

h We find that 16.6% of those indicating a willingness to sign the petition ultimately followed a link provided at the end of the survey to actually send the petition. Approximately 1% of those that did not indicate a willingness to sign the petition also followed the link. This suggests one’s willingness to engage in political action is an imperfect proxy for actual political behavior. However, this lack of correspondence could reflect measurement error (e.g. respondents that paste the link into a new browser window are not captured). We do not interpret the COVAX donations as the respondents’ marginal propensity to give, but donations are a costly measure of changes in preferences across experimental conditions. In addition, donations are highly predictive of respondents’ willingness to support international agreements in the conjoint experiment, providing a behavioral validation of this measure of stated preferences (see Supplementary Material Section S3.3, Table S4, Figures S13 and S14)
