# Supplementary material for: Building mass support for global pandemic recovery efforts in the United States
Source: PNAS Nexus. 2022 Aug 16;1(4):pgac123. doi: 10.1093/pnasnexus/pgac123 (PMC9802409; doi:10.1093/pnasnexus/pgac123)
Supplement: pgac123_Supplemental_File [file pgac123_supplemental_file.pdf]

Supporting Information for 'Building mass support for global pandemic recovery efforts in the United States'

Version: June 2022

Contents

List of Figures

List of Tables

S1 Lucid sample (Survey 1) 1
- S1.1 Survey measures used for analyses of causal effect heterogeneity 2
- S1.2 Conjoint experiment on potential vaccine recipients 5
- S1.3 Conjoint experiment on international agreements 6

S2 NORC/Amerispeak sample (Survey 2) 7
- S2.1 Survey measures used for analyses of causal effect heterogeneity 8
- S2.2 Conjoint experiment on international agreements 9
- S2.3 Persuasive messaging experiment 10

S3 Supplementary analyses, tables and figures 14
- S3.1 Comparisons between weighted and un-weighted estimates 16
- S3.2 Heterogeneity in vaccine recipient conjoint by country of origin 24
- S3.3 Support for international agreements in NORC conjoint and donations to COVAX 27
- S3.4 Estimated marginal means and AMCEs on ordinal outcome measures 30
- S3.5 Effect heterogeneity in vaccine recipient conjoint by respondents' background characteristics 36
- S3.6 Effect heterogeneity in international agreement conjoint (Lucid) by respondents' background characteristics 54
- S3.7 Effect heterogeneity in international agreement conjoint (NORC) by respondents' background characteristics 72
- S3.8 Effect heterogeneity in persuasion experiment by respondents' background characteristics 84

References 92

S4 Pre-registration for vaccine recipient conjoint in Lucid survey 94

S5 Pre-registration for international agreement conjoint in Lucid survey 95

S6 Pre-registration for international agreement conjoint in NORC survey 96

S7 Pre-registration for persuasion experiment in NORC survey 97

S8 NORC/AmeriSpeak sampling methodology 99

List of Figures

S1 Correlations among survey items used for analyses of causal effect heterogeneity in Lucid sample . . . 5
S2 Correlations among survey items used for analyses of causal effect heterogeneity in NORC sample . . . 9
S3 Estimated marginal means in vaccine recipient conjoint with and without survey weights . . . 16
S4 Estimated AMCEs in vaccine recipient conjoint with and without survey weights 17
S5 Estimated marginal means in international agreement conjoint (Lucid) with and without survey weights . . . 18
S6 Estimated AMCEs in international agreement conjoint (Lucid) with and without survey weights . . . 19
S7 Estimated marginal means in international agreement conjoint (NORC) with and without survey weights . . . 20
S8 Estimated AMCEs in international agreement conjoint (NORC) with and without survey weights . . . 21
S9 Estimated ATEs in persuasion experiment with and without survey weights . . . 22
S10 Estimated conditional marginal means in vaccine recipient conjoint by non-U.S. v. U.S. origin . . . 24
S11 Estimated conditional AMCEs in vaccine recipient conjoint by non-U.S. v. U.S. origin . . . 25
S12 Estimated marginal means for paired comparisons between potential vaccine recipients from the U.S. and another country . . . 26
S13 Estimated marginal means in international agreement conjoint (NORC) by donation amount . . . 28
S14 Estimated AMCEs in international agreement conjoint (NORC) by donation amount 29
S15 Estimated marginal means on ordinal measure of support for potential vaccine recipients, with and without survey weights . . . 30
S16 Estimated AMCEs on ordinal measure of support for potential vaccine recipients, with and without survey weights . . . 31
S17 Estimated marginal means on likelihood of voting for international agreement in Lucid conjoint, with and without survey weights . . . 32
S18 Estimated AMCEs on likelihood of voting for international agreement in Lucid conjoint, with and without survey weights . . . 33
S19 Estimated marginal means on likelihood of voting for international agreement in NORC conjoint, with and without survey weights . . . 34
S20 Estimated AMCEs on likelihood of voting for international agreement in NORC conjoint, with and without survey weights . . . 35
S21 Estimated marginal means in vaccine recipient conjoint by vaccine nationalism indicator . . . 36
S22 Estimated AMCEs in vaccine recipient conjoint by vaccine nationalism indicator 37
S23 Estimated marginal means in vaccine recipient conjoint by vaccine nationalism index . . . 38
S24 Estimated AMCEs in vaccine recipient conjoint by vaccine nationalism index . . . 39
S25 Estimated marginal means in vaccine recipient conjoint by nationalism . . . 40
S26 Estimated AMCEs in vaccine recipient conjoint by nationalism . . . 41
S27 Estimated marginal means in vaccine recipient conjoint by cosmopolitanism . . . 42

S28 Estimated AMCEs in vaccine recipient conjoint by cosmopolitanism 43
S29 Estimated marginal means in vaccine recipient conjoint by altruism 44
S30 Estimated AMCEs in vaccine recipient conjoint by altruism 45
S31 Estimated marginal means in vaccine recipient conjoint by reciprocity 46
S32 Estimated AMCEs in vaccine recipient conjoint by reciprocity 47
S33 Estimated marginal means in vaccine recipient conjoint by vaccination status . . 48
S34 Estimated AMCEs in vaccine recipient conjoint by vaccination status 49
S35 Estimated marginal means in vaccine recipient conjoint by partisanship 50
S36 Estimated AMCEs in vaccine recipient conjoint by partisanship 51
S37 Estimated marginal means in vaccine recipient conjoint by political ideology . . . 52
S38 Estimated AMCEs in vaccine recipient conjoint by political ideology 53
S39 Estimated marginal means in international agreement conjoint (Lucid) by vaccine nationalism indicator 54
S40 Estimated AMCEs in international agreement conjoint (Lucid) by vaccine nationalism indicator 55
S41 Estimated marginal means in international agreement conjoint (Lucid) by vaccine nationalism index 56
S42 Estimated AMCEs in international agreement conjoint (Lucid) by vaccine nationalism index 57
S43 Estimated marginal means in international agreement conjoint (Lucid) by nationalism 58
S44 Estimated AMCEs in international agreement conjoint (Lucid) by nationalism . . 59
S45 Estimated marginal means in international agreement conjoint (Lucid) by cosmopolitanism 60
S46 Estimated AMCEs in international agreement conjoint (Lucid) by cosmopolitanism 61
S47 Estimated marginal means in international agreement conjoint (Lucid) by altruism 62
S48 Estimated AMCEs in international agreement conjoint (Lucid) by altruism 63
S49 Estimated marginal means in international agreement conjoint (Lucid) by reciprocity 64
S50 Estimated AMCEs in international agreement conjoint (Lucid) by reciprocity . . 65
S51 Estimated marginal means in international agreement conjoint (Lucid) by vaccination status 66
S52 Estimated AMCEs in international agreement conjoint (Lucid) by vaccination status 67
S53 Estimated marginal means in international agreement conjoint (Lucid) by partisanship 68
S54 Estimated AMCEs in international agreement conjoint (Lucid) by partisanship . 69
S55 Estimated marginal means in international agreement conjoint (Lucid) by political ideology 70
S56 Estimated AMCEs in international agreement conjoint (Lucid) by political ideology 71
S57 Estimated marginal means in international agreement conjoint (NORC) by vaccine nationalism 72
S58 Estimated AMCEs in international agreement conjoint (NORC) by vaccine nationalism 73
S59 Estimated marginal means in international agreement conjoint (NORC) by nationalism 74
S60 Estimated AMCEs in international agreement conjoint (NORC) by nationalism . 75
S61 Estimated marginal means in international agreement conjoint (NORC) by patriotism 76
S62 Estimated AMCEs in international agreement conjoint (NORC) by patriotism . . 77

S63 Estimated marginal means in international agreement conjoint (NORC) by altruism 78
S64 Estimated AMCEs in international agreement conjoint (NORC) by altruism . . . 79
S65 Estimated marginal means in international agreement conjoint (NORC) by partisanship 80
S66 Estimated AMCEs in international agreement conjoint (NORC) by partisanship . 81
S67 Estimated marginal means in international agreement conjoint (NORC) by political ideology 82
S68 Estimated AMCEs in international agreement conjoint (NORC) by political ideology 83
S69 Causal forest estimated treatment effects in persuasion experiment 85
S70 Causal forest estimated treatment effects in persuasion experiment by partisanship 86
S71 Causal forest estimated treatment effects in persuasion experiment by political ideology 87
S72 Causal forest estimated treatment effects in persuasion experiment by altruism . 88
S73 Causal forest estimated treatment effects in persuasion experiment by vaccine nationalism 89
S74 Causal forest estimated treatment effects in persuasion experiment by nationalism 90
S75 Causal forest estimated treatment effects in persuasion experiment by patriotism 91

List of Tables

S1 Group means (S.D.s) of covariates and pre-treatment attitudes by condition . . . 13
S2 Demographic characteristics in U.S. adult population and survey samples 15
S3 Estimated ATEs in persuasion experiment with and without survey weights . . . 23
S4 Likelihood of voting for agreement as a function of amount donated to COVAX and randomized features 27

S1 Lucid sample (Survey 1)

Data on 1,751 U.S. adults were collected between 12 April and 14 April 2021 via a survey fielded on the Lucid platform, which provides researchers with access to a sampling frame of over 11 million U.S. adults. Lucid has grown in popularity over the last five years and is now one of the most widely used online survey platforms for academic research; in 2020, Lucid sold over 2 million completed survey responses to academic clients [1]. Like other firms that source from opt-in online panels (e.g., YouGov, Qualtrics), Lucid provides researchers with quota samples of respondents that match U.S. census margins on demographic characteristics, and Lucid's quota samples compare favorably with nationally representative samples on both demographics and psychological traits [2].

Following best practices to ensure data quality in opt-in online panels, we restricted participation to respondents who read and passed an attention screener placed at the beginning of the survey [3, 1]. The screener was the first question displayed to respondents that consented to participate: "In this study, careful attention to survey questions is critical. To show that you are paying attention please select 'I have a question."' Responses options: "I understand", "I do not understand", and "I have a question" were displayed. Respondents that did not select "I have a question" were not eligible to complete the survey. Of the 2,259 respondents who consented to participate in the survey, 78% (N = 1,751) passed the attention check question and completed the survey.

This sample of 1,751 individuals was comparable to the U.S. adult population on sex, geographic region, age, and race/ethnicity, and at least as representative as the NORC/AmeriSpeak probability sample on demographic marginals (see Table S2). We also find that our descriptive estimates of vaccine nationalism (using the binary measure described in Section S1.1) closely match those obtained from probability samples conducted in the preceding months (Lucid: 67.44% without weights; 67.38% with weights. Pew survey from February 2021: 66%). Our measure of vaccination status (proportion of respondents who had received at least one dose) was also similar to available population benchmarks (Lucid: 56.83% without survey weights; 54.4% with weights. The CDC reported in April 2021 that 55% of adults had received at least one dose.[1]

We construct survey weights to adjust for potential differences in respondent demographics along the following characteristics: sex, region, race/ethnicity, household income, educational attainment, and age. Weights are constructed using the autumn package in R, which implements an iterative raking procedure used by the American National Election Study (ANES) survey [4, 5]. Applying the weights reduced the average difference between the sample and population proportions by 30% (from 0.02 to 0.006) for an effective sample size of 1,022 units from a nominal size of 1,751 (implying a design effect of 1.71). Table S2 compares the unweighted sample proportions, weighted sample proportions, and the target proportions for the U.S. adult population across demographic characteristics.

Each conjoint experiment was presented in randomized order. A second attention check

[1] See: https://www.usnews.com/news/national-news/articles/2021-04-29/cdc-30-of-us-population-fully-vaccinated-against-coronavirus).

question was added after the first randomly presented conjoint to measure respondent attention and 92% of respondents (1605/1751) passed.[2] The 146 respondents that did not pass were not excluded from any analyses presented here or in the manuscript. The median time to complete the entire survey was 12.5 minutes. Both experiments were pre-registered in advance of data collection. Anonymized versions of the pre-registrations for the vaccine recipient conjoint and the international agreement conjoint are provided in Appendix Sections S4-S5, and available for download from AsPredicted.org at `https://aspredicted.org/PVH_P8V` and `https://aspredicted.org/3GC_L3K`. Our goal was to recruit at least 1,500 participants, based on simulation studies that showed our experimental designs (described in more detail below) would be adequately powered (at least 0.80) to detect effects of 5 percentage points (fixing alpha = 0.05) at this sample size.

S1.1 Survey measures used for analyses of causal effect heterogeneity

In addition to the pre-treatment demographic characteristics provided by Lucid (see Table S2) respondents were also asked the questions detailed below. We pre-registered that these questions would be used in the exploratory analyses of causal effect heterogeneity presented in Sections S3.5 (vaccine recipient conjoint) and S3.6 (international agreement conjoint). All index measures here and in the manuscript are constructed by combining individual items using inverse covariance weighting [6]. Figure S1 shows the pairwise correlations between each individual item.

1. **Vaccine Nationalism Indicator** (`vnat_binary`). A binary question adopted from a Pew survey of U.S. adults conducted in February 2021[7]: "Thinking about developing countries around the world, which statement comes closer to your view about COVID-19 vaccines, even if neither is exactly right?" [0 = "The U.S. should help ensure that people in developing countries have access to vaccines, even if it means some people in the U.S. need to wait longer to get vaccines"; 1 = "The U.S. should ensure that there are enough vaccines for people in the U.S., even if it means people in developing countries need to wait longer to get vaccines"]

2. **Vaccine Nationalism Index** (`national_binary`). A binary recode of a 5-item index constructed from the following, coded 1 ("high") if a respondent scored higher than the median respondent and 0 ("low") otherwise [each measured on 7-point scales from "Strongly disagree" (1), ..., "Neither agree nor disagree" (4),...,"Strongly agree" (7)]. These questions – specific to the COVID-19 pandemic – were developed for this particular study and are not part of any established research literature that we are aware of.

   - `vax_nat_1`. "COVID-19 vaccines should be given to all Americans that want one before any are donated to people in other countries."
   - `vax_nat_2`. "Regardless of whether we have enough supply to vaccinate all Americans, vaccine doses should not be shared with other countries."
   - `vax_nat_3`. "The United States should cooperate with international efforts aimed at providing vaccines to people that need them in other countries" (reverse coded).

[2] Respondents were presented with the following question: "People are very busy these days and many do not have time to follow what goes on in the government. We are testing whether people read questions. To show that you've read this much, answer both 'extremely interested' and 'very interested."' Response options: "Extremely interested", "Very interested", "Moderately interested", "Slightly interested", "Not at all interested".

- `vax_nat_4`. "The United States should share the technology required to produce COVID-19 vaccines with other countries, even if comes at a cost to American companies" (reverse coded).
- `vax_nat_5`. "The United States should fund manufacturing facilities in developing countries to expand global vaccine supply" (reverse coded).

3. **Nationalism** (`national_binary`). A binary recode of the following 7-point scale, coded 1 ("high") if a respondent scored higher than the median respondent and 0 ("low") otherwise. This single-item measure, adopted from Pew surveys of U.S. adults, captures support for the U.S. over other countries [8, 9].

   - `national`. "For the most part, the U.S. is better than any other country in the world." [1 = "Strongly disagree",..., 4= "Neither agree nor disagree",...,7="Strongly agree"].

4. **Cosmopolitanism** (`cosmop_binary`). A binary recode of the following 7-point scale, coded 1 ("high") if a respondent scored higher than the median respondent and 0 ("low") otherwise. This measure has been widely used in prior studies to capture one's orientation towards the world rather than their local community, i.e. "the extent to which people have an interest in, and orientation towards, groups of individuals who are distant from them, geographically or culturally, as opposed to a local orientation that extends only to one's more immediate community" [10, 11, 12].

   - `cosmop`. "Although the media often reports about national and international events and developments, this news is rarely as interesting as the things that happen directly in our own community and neighborhood." [1 = "Strongly agree",..., 4= "Neither agree nor disagree",...,7="Strongly disagree"].

5. **Altruism** (`altruism_binary`). A binary recode of a 2-item index constructed from the following, coded 1 ("high") if a respondent scored higher than the median respondent and 0 ("low") otherwise. These measures are taken from recent work on survey-based measures of social preferences, and are prognostic of altruistic behavior as measured using experimental games [13].

   - `altruism_willing`. "In general, how willing or unwilling are you to give to good causes without expecting anything in return?" [1 = "Very unwilling",...,4= "Neither willing nor unwilling", ...,7="Very willing"]
   - `altruism_donate`. "Imagine the following situation: today you unexpectedly win $1,000. How much of this amount would you donate to a good cause? (Values between 0 and 1,000 are allowed)"

6. **Reciprocity** (`recip_gift_binary`). A binary recode of the following, coded 1 ("high") if a respondent scored higher than the median respondent and 0 ("low") otherwise. This measure is also taken from recent work on survey-based measures of social preferences, and is prognostic of conditional cooperation as measured using experimental games [13].

   - `recip_gift`. Please think about what you would do in the following situation. You are in an area you are not familiar with, and you realize that you lost your way. You ask a stranger for directions. The stranger offers to take you to your destination. Helping you costs the stranger about $20 in total. However, the stranger says they do not want any money from you. You have 6 presents with you. The cheapest is worth $5, and the most expensive one is worth $30. Do you give one of the presents to the stranger as a 'thank-you' gift? If so, which one?" [0 = "No present"; 5 = "The

present worth $5"; 10 = "The present worth $10"; 15 = "The present worth $15"; 20 = "The present worth $20"; 25 = "The present worth $25"; 30 = "The present worth $30"]

7. **Vaccination status** (`covid_vaxed_binary`). "Have you received at least one dose of any COVID-19 vaccine?" [0 = "No"; 1 = "Yes"]

   - `covid_fully_vaxed`. Displayed if `covid_vaxed_binary` = 1: "Have you been fully vaccinated?" [0 = "No, but I intend to receive the 2nd dose"; 0 = "No, and I do not intend to receive the 2nd dose"; 1 = "Yes, I have received 2 doses (e.g.,Pfizer/Moderna)"; 1 = "Yes, my vaccine requires a single dose (e.g., Johnson & Johnson)"]
   - `covid_antivax`. Displayed if `covid_vaxed_binary` = 0: "Which of the following best describes you?" [0 = "I am not yet eligible to receive a vaccine, but intend to take one"; 0 = "I am eligible but haven't been able to schedule an appointment"; 0 = "I am eligible and have a confirmed appointment"; 1 = "I am eligible, but I do not intend to take a vaccine"; 1 = "I am not yet eligible, and I do not intend to take a vaccine"]

8. **Partisanship** (`x_pid_7`). A 7-point scale based on the branching question used in the American National Election Studies (ANES) Survey: "Strong Democrat" (1), "Weak Democrat" (2), "Lean Democrat" (3), "Independent" (4), "Lean Republican" (5), "Weak Republican" (6), "Strong Republican" (7).

9. **Ideology** (`x_ideo_7`). A 7-point political ideology scale: "Extremely liberal" (1), "Liberal" (2), "Slightly Liberal" (3), "Moderate" (4), "Slightly Conservative" (5), "Conservative" (6), "Extremely Conservative" (7).

Figure S1: Correlations among survey items used for analyses of causal effect heterogeneity in Lucid sample

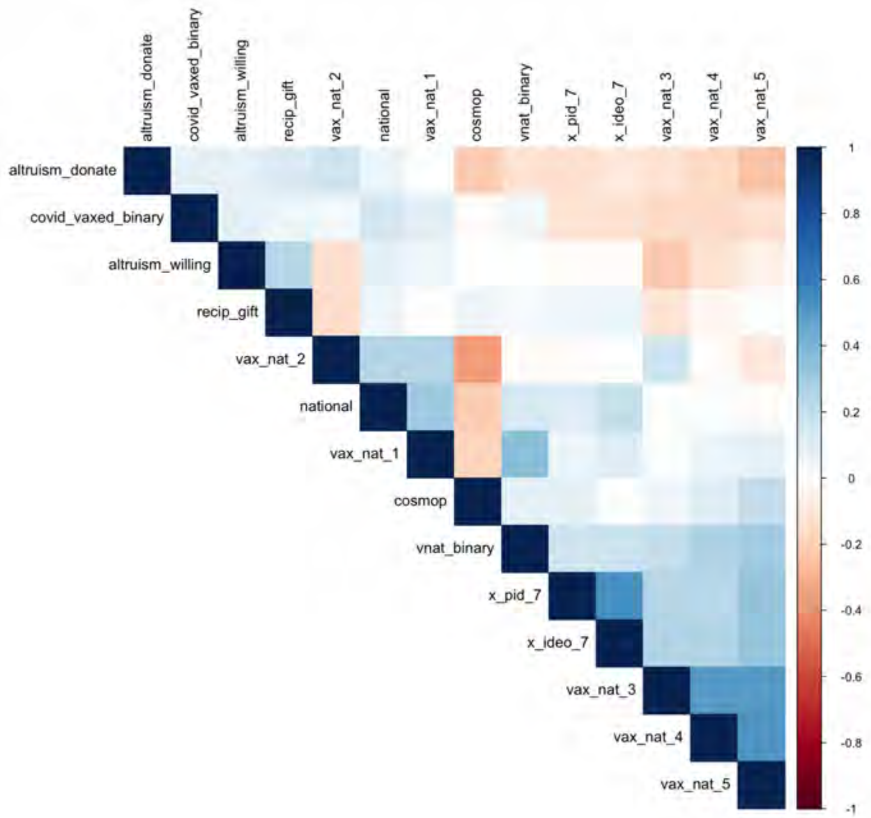

S1.2 Conjoint experiment on potential vaccine recipients

The next part of this survey examines COVID-19 vaccine allocation. We will provide you with information about different people waiting in line to receive a COVID-19 vaccination. For each pair of people, please indicate who you think should be given priority to receive a vaccination.

Although this exercise is purely hypothetical, the information we provide is based on real allocation rules and therefore provides a realistic portrait of the types of decisions that will have to be made.

Please remember that global demand for vaccines greatly exceeds supply. Even if you aren't entirely sure, please indicate which of the two people you think should be given priority. The information you will be provided about each person is shown below.

	Information that will be provided
**Sex**	Whether each person is male or female
**Age group**	The age group that each person belongs to
**Risk of exposure to COVID-19**	Each person's risk of exposure to COVID-19
**Risk of serious illness from COVID-19**	Each person's risk of serious illness from a COVID-19 infection due to underlying health issues
**Occupation group**	The broad occupational group that each person belongs to
**Can work from home?**	Whether each person can work from home or not
**Country of Origin**	The country where each person lives

Respondents then evaluated five pairs of hypothetical agreements (N = 1,751 respondents x 5 pairings x 2 agreements per pair = 17,510 observations) with the following randomly assigned features drawn for each attribute (in bold) with uniform distribution:

- **Sex:** Male; Female
- **Age group**: 18-24; 25-34; 35-44; 45-54; 55-64; 65-74; 75+
- **Risk of exposure to COVID-19**: Low; Moderate; High
- **Risk of serious illness from COVID-19**: Low; Moderate; High
- **Occupation group**: Non-essential workers; Healthcare workers; First responders (e.g., police/fire); Education and childcare; Public transit
- **Can work from home?**: Yes; No
- **Country of origin**: United States; Canada; Brazil; South Africa; Nigeria; India; Australia; Pakistan; China

For each agreement that a respondent evaluated, they were asked "If you had to choose between them, which of these two people should be given priority to receive the vaccine?" We constructed a binary measure of support for vaccinating each potential recipient coded 1 if a respondent chose that individual and 0 if they did not. In addition to this forced choice outcome, respondents were also asked: "Please rate each person on a scale from 1 to 7, where 1 indicates they should definitely not receive the vaccine and 7 indicates they should definitely receive the vaccine." This measure provides an assessment of the absolute support for a potential vaccine recipient.

S1.3 Conjoint experiment on international agreements

> Most countries around the world are currently developing new policies regarding access to COVID-19 vaccines. In the next section, we will provide you with several examples of what agreements between countries could look like. For each pair of agreements, please indicate which of the two you think the United States should join.
>
> You may like both alternatives similarly or may not like either of them at all. Regardless of your overall evaluation, please indicate which alternative you prefer over the other. The information you will be provided about each international agreement is shown below.

	Information that will be provided
**Number of participating countries**	The number of countries that will join each agreement
**Costs to average U.S. household**	How much implementing each agreement will cost a typical American household
**Distribution of costs**	How the costs of implementing each agreement will be distributed between countries
**Distribution of benefits**	How the vaccines obtained through each agreement will be distributed between countries
**External supply agreements allowed?**	Whether participating countries can make separate agreements to purchase vaccines directly from manufacturers
**Sharing of vaccine technology**	Whether sharing of vaccine research and manufacturing technologies between participating countries is compulsory or voluntary
**Monitoring**	How compliance with the rules of each agreement will be monitored

For each agreement that a respondent evaluated, they were asked "Which of these two agreements do you prefer?" We constructed a binary measure of support for each agreement coded 1 if an individual chose that agreement and 0 if they did not. In addition to this forced choice outcome, respondents were also asked: "If you could vote on each of these agreements, how likely is it that you would vote in favor or against each of the agreements? Please give your answer on the following scale from definitely against (1) to definitely in favor (7)." This measure provides an assessment of the absolute support for a given agreement.

S2 NORC/Amerispeak sample (Survey 2)

Data on 4,214 U.S. adults (aged 18 and older) were collected between 9 September and 15 October 2021 via a survey fielded on the NORC/AmeriSpeak panel, a probability based-panel of U.S. adults. For a given study, NORC selects samples from the panel using sampling strata based on age, race/ethnicity, education, and gender with sample size proportional to the population distribution in each stratum. In addition to these demographic characteristics, NORC's sample selection methodology adjusts for expected completion rates by demographic sub-groups so that the final set of individuals that ultimately complete the interview (here 4,214) are representative of the target population on demographic characteristics. Panelists were offered the cash equivalent of $2 for completing the study, and the median time to complete the module was 5 minutes.

This sample of 4,214 respondent was comparable to the U.S. adult population and our Lucid sample on sex, region, age, and race/ethnicity (see Table S2). NORC constructs survey weights using a combination of inverse probability weighting (to adjust for non-response from their sampling frame) and iterative raking (to adjust for deviations from the Current Population Survey on demographic characteristics). Applying the survey weights yields a margin of error

of 2.04% with an effective sample size of 2,254 units from a nominal sample of 4,421 (implying a design effect of 1.83). The weighted AAPOR RR3 recruitment rate was 19.1, the household retention rate was 75, the survey completion rate was 30.4, and the weighted AAPOR RR3 cumulative response rate was 4.4. A complete description of NORC's sampling methodology is provided in Section S8. Table S2 compares the unweighted sample proportions, weighted sample proportions, and the target proportions for the U.S. adult population across demographic characteristics.

Respondents first completed the conjoint experiment on international agreements (detailed in Section S2.2) and then completed the persuasion experiment (detailed in Section S2.3). Both experiments were pre-registered in advance of data collection. Anonymized version of the international agreement conjoint and the persuasion experiment are provided in Appendix Sections S6-S7, and available for download from AsPredicted.org at `https://aspredicted.org/PQV_PB4` and `https://aspredicted.org/RLW_1QR`.

S2.1 Survey measures used for analyses of causal effect heterogeneity

In addition to the pre-treatment demographic characteristics provided by NORC, respondents from the panel were also asked the questions detailed below. We pre-registered that these questions would be used in the exploratory analyses of causal effect heterogeneity presented in Sections S3.7 (international agreement conjoint) and S3.8 (persuasion experiment). Figure S2 shows the pairwise correlations between each of these measures.

1. **Vaccine nationalism** (`vax_nationalism`). "The US should cooperate with international efforts to provide COVID-19 vaccines to people in other countries." [5 = "Strongly disagree",..., 3= "Neither agree nor disagree",...,1="Strongly agree"].

2. **Nationalism** (`nationalism`). "I would rather be a citizen of the US than of any other country in the world." [1 = "Strongly disagree",..., 3= "Neither agree nor disagree",...,5="Strongly agree"].

3. **Patriotism** (`patriotism`). "I am proud to be American." [1 = "Strongly disagree",..., 3= "Neither agree nor disagree",...,5="Strongly agree"].

4. **Altruism** (`altruism`). "I am willing to give to good causes without expecting anything in return." [1 = "Strongly disagree",..., 3= "Neither agree nor disagree",...,5="Strongly agree"]. This measure (also included in our Lucid survey) is taken from recent work on survey-based measures of social preferences, and is prognostic of altruistic behavior as measured using experimental games [13].

5. **Partisanship** (`partisanship`). A 7-point scale based on the branching question used in the American National Election Studies (ANES) Survey: "Strong Democrat" (1), "Weak Democrat" (2), "Lean Democrat" (3), "Independent" (4), "Lean Republican" (5), "Weak Republican" (6), "Strong Republican" (7).

6. **Ideology** (`ideology`). A 7-point political ideology scale: "Extremely liberal" (1), "Liberal" (2), "Slightly Liberal" (3), "Moderate" (4), "Slightly Conservative" (5), "Conservative" (6), "Extremely Conservative" (7).

Figure S2: Correlations among survey items used for analyses of causal effect heterogeneity in NORC sample

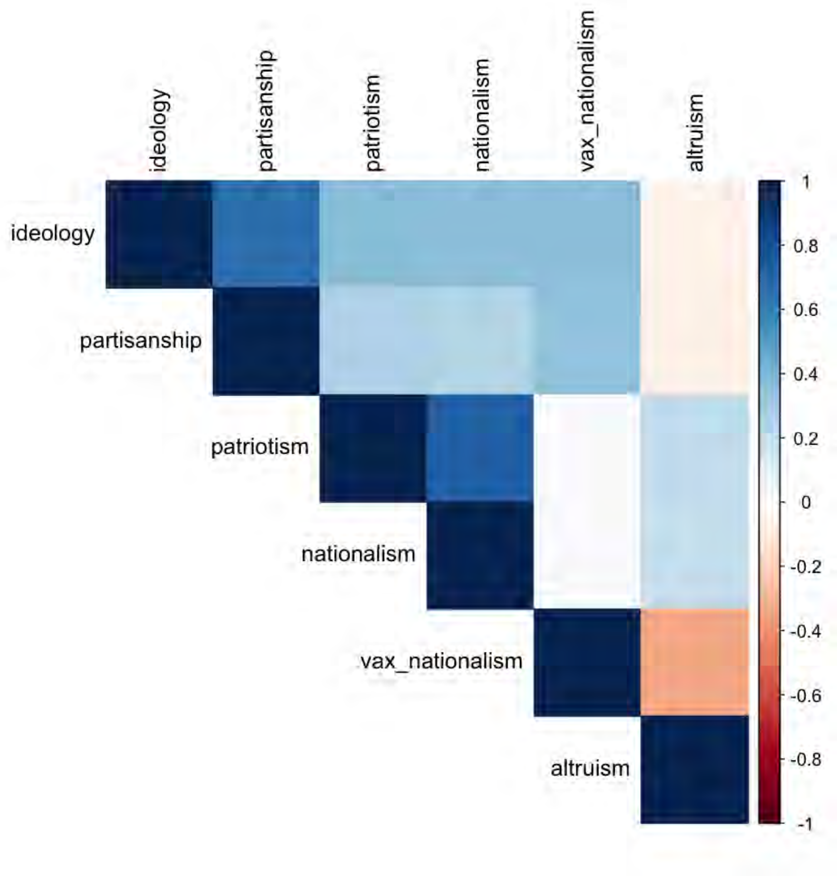

S2.2 Conjoint experiment on international agreements

After answering the questions enumerated in Section S2.1, and before the persuasion experiment described in the Section S2.3, respondents were presented with the following text describing the conjoint experiment that would appear on subsequent pages:

> Countries are attempting to reach an agreement on how to combat the COVID-19 global pandemic. In the next section, we will provide you with several examples of what agreements between countries could look like. For each pair of agreements, please indicate which of the two you think the US should support. The information you will be provided about each international agreement is shown below.

	Information that will be provided
**Overall cost of agreement**	How much implementing each agreement will cost the world.
**Proportion of costs paid by the U.S.**	Percentage of the total cost that will be paid by the United States.
**Funding will be used to**	How the funding in each agreement will be used.
**Benefits will be directed to**	How the benefits of each agreement will be distributed between countries.
**Duration of agreement**	How long each agreement will be in place.

Respondents then evaluated two pairs of hypothetical agreements (N = 4,214 respondents x 2 pairings x 2 agreements per pair = 16,856 observations) with the following randomly assigned features drawn for each attribute (in bold) with uniform distribution:

- **Overall cost of program:** \$100 billion; \$75 billion; \$50 billion; \$25 billion
- **Proportion of costs paid by the U.S.:** 100%; 75%; 50%; 25%; 0%
- **Funding will be used to:** Purchase vaccines made by companies in the US; Purchase vaccines made by companies outside the US; Purchase patents from vaccine companies to make production technology freely available to the world; Finance health infrastructure and health campaigns in poor countries; Provide economic aid and debt forgiveness to COVID-affected countries.
- **Benefits will be directed to:** US allies and US-aligned countries; countries most at risk of COVID-19 outbreaks; poor and low-income countries.
- **Duration of program:** 1 year; 2 years; 3 years; 4 years; 5 years.

For each agreement that a respondent evaluated, they were asked "If you had to choose, which agreement do you favor?" We constructed a binary measure of support for each agreement coded 1 if an individual chose that agreement and 0 if they did not. In addition to this forced choice outcome, respondents were also asked: "If you could vote on each of these agreements, how likely is it that you would vote in favor or against each of the agreements?" This measure provides an assessment of the absolute support for a given agreement on the following scale: "Definitely against" (1); "Probably against" (2); "Neither in favor nor against" (3); "Probably in favor" (4); "Definitely in favor" (5).

S2.3 Persuasive messaging experiment

At the end of the NORC/AmeriSpeak survey, following the conjoint experiment (described in previous section), respondents were first presented with the following text:

> In this final section, you will be provided with some factual information and asked some questions about global initiatives that seek to provide access to COVID-19 vaccines around the world. There are no "right" or "wrong" answers to these questions. This survey is anonymous, and we are interested in your honest opinions. Please answer to the best of your ability.

The purpose of this text was twofold. First, to emphasize that subsequent information would be factual rather than hypothetical (i.e, no deception). Second, following best practice recommendations for mitigating the potential of "social desirability biases", to provide additional assurances that responses are anonymous and remind participants that there are no "wrong answers" [14]. We note that such biases (also called "demand effects") have been difficult to find in the anonymous online research environment, and the search for substantively meaningful biases under this umbrella has proved quixotic across the many published studies, both observational and experimental, that have tried to find or induce them [15, 16, 17, 18, 19, 14]. Following the assurance text, respondents were then provided with one of the five randomly assigned messages – or nothing if assigned to control – enumerated below:

1. **Global inequality**. Rich countries have secured most of the world's COVID-19 vaccine supplies. Approximately 80% of the doses administered worldwide have been in rich countries, whereas less than 1% of doses have been administered in low-income countries. Many in Asia, Africa, and Latin America will not have access to a vaccine for the foreseeable future. The US can help to reduce these inequalities by coordinating a global effort to rapidly vaccinate the world against COVID-19.

2. **Past success**. In 2003, the US created the President's Emergency Plan for AIDS Relief (PEFPAR) to share medicines and technical support with partner countries in order to treat HIV/AIDS and other infectious diseases. PEPFAR is widely considered to be one of the most effective public health efforts in history and is estimated to have saved as many as 20 million lives. The US can build on successful past public health campaigns like PEFPAR to coordinate a global effort to rapidly vaccinate the world against COVID-19.

3. **Mutation risk**. As COVID-19 continues to spread in countries around the world, new variants are emerging. Some of these variants, such as those found in the United Kingdom, South Africa, and India, can be more contagious or deadly than strains for which vaccines have already been developed. The Delta variant, which first emerged in India, is now dominant in the US. The US can help to prevent the emergence of new strains by coordinating a global effort to rapidly vaccinate the world against COVID-19.

4. **Economic benefits**. A recent study found that if the poorest countries cannot access COVID-19 vaccines, the world could lose between $60 billion and $340 billion a year in income. The researchers concluded that for every $1 spent on supplying poorer countries with vaccines, high-income countries would get back about $4.80 through greater exports and other mechanisms. The US can help secure these benefits, which greatly exceed the costs, by coordinating a global effort to rapidly vaccinate the world against COVID-19.

5. **Vaccine diplomacy**. Countries like China and Russia are using the pandemic to increase their strategic influence. These countries are utilizing COVID-19 vaccines as a tool of diplomacy to win friends and allies. China has exported nearly 800 million doses of its Sinovac and Sinopharm vaccines to countries across Asia, Latin America, Africa, and Europe. The US, however, has only sent about 100 million doses to other countries. The US can counter China's rising influence by coordinating a global effort to rapidly vaccinate the world against COVID-19.

Next, the following outcome measures were displayed in randomized order:

1. "Recent studies by economists at the International Monetary Fund suggest that as much as $50 billion is required to quickly vaccinate everyone in all countries. In your view, what percentage of this amount should the U.S. fund? [0-100% slider]"

2. "Are you willing to support a petition* calling on Congress to increase US spending on COVID-19 assistance abroad? [Yes = 1; No = 0] *A link to the petition will be provided at the end of the survey."

3. "Approximately 100 participants in this survey will be randomly selected for a bonus payment of $10. If selected, you may keep the entire amount or donate a part of the payment to the COVID-19 Vaccines Global Access Initiative (COVAX), an international initiative to vaccinate people in low income countries. Your choice will not affect your chance of being selected for the bonus. Any amount you choose to donate will be deducted from your bonus payment before it is credited to you. What amount (if any) would you like to donate? [Donation amount: $(0-10 numeric entry); Keep amount: $(10 - Donation amount displayed)]"

To further examine the potential for demand effects, all participants that won the randomly assigned bonus were informed of this prior to responding to the outcome measures enumerated above. We find no evidence that learning about winning the bonus prior to answering these questions had a measurable effect on responses. The average effect on winning the bonus on the donation outcome was an increase in the amount donated of approximately 0.07 cents out of $10 USD (SE = 0.45, $P = 0.88$). The average effect on willingness to sign the petition (binary outcome) was 0 (SE = 0.05, $P = 0.94$), and the average effect on the proportion of total costs the U.S. should fund was 3.13 on a 100 point scale (SE = 2.6, $P = 0.23$).

Table S1 shows the number of subjects in each condition (P-value from Chi-squared test for equality of proportions is 0.82), as well as the group means (and S.D.s) across pre-treatment covariates, including the attitudinal measures described in Section S2.1. Given that we have multiple treatment arms and a large number of covariates, we conduct an omnibus "balance test" by regressing the treatment assignment on all pre-treatment measures listed in Table S1. We use the F-statistic from this regression to test the (sharp) null hypothesis that no covariates have any effect on the assigned treatment using randomization inference [20]. The P-value is therefore the proportion of simulated F-statistics as large (or larger) than the one observed under the null of covariate balance. The observed F-statistic is 1.09, and the P-value from 5,000 simulations of the experimental design is 0.36. Given that 36% of simulated F-statistics were at least as large as the observed F-statistic, we conclude that imbalance is not greater than one would expect by chance.

Table S1: Group means (S.D.s) of covariates and pre-treatment attitudes by condition

	**Control**	**Economic benefits**	**Mutation risk**	**Past success**	**Vaccine diplomacy**	**Global inequality**
	($n = 709$)	($n = 708$)	($n = 727$)	($n = 684$)	($n = 680$)	($n = 706$)
Sex:						
Female	0.52 (0.50)	0.49 (0.50)	0.52 (0.50)	0.50 (0.50)	0.52 (0.50)	0.51 (0.50)
Male	0.48 (0.50)	0.51 (0.50)	0.48 (0.50)	0.50 (0.50)	0.48 (0.50)	0.49 (0.50)
Region:						
South	0.33 (0.47)	0.32 (0.47)	0.32 (0.47)	0.35 (0.48)	0.31 (0.46)	0.32 (0.47)
West	0.26 (0.44)	0.26 (0.44)	0.26 (0.44)	0.25 (0.44)	0.25 (0.44)	0.27 (0.44)
Midwest	0.25 (0.44)	0.28 (0.45)	0.29 (0.45)	0.26 (0.44)	0.27 (0.44)	0.27 (0.44)
Northeast	0.15 (0.36)	0.15 (0.36)	0.12 (0.33)	0.14 (0.34)	0.17 (0.37)	0.14 (0.35)
Race/Ethnicity:						
White	0.63 (0.48)	0.61 (0.49)	0.66 (0.47)	0.63 (0.48)	0.61 (0.49)	0.66 (0.48)
Hispanic	0.14 (0.35)	0.17 (0.38)	0.16 (0.37)	0.17 (0.38)	0.20 (0.40)	0.16 (0.37)
Black	0.13 (0.34)	0.13 (0.33)	0.11 (0.32)	0.11 (0.32)	0.11 (0.31)	0.11 (0.31)
AAPI	0.04 (0.19)	0.03 (0.18)	0.03 (0.16)	0.03 (0.17)	0.03 (0.16)	0.03 (0.17)
Other	0.05 (0.22)	0.06 (0.23)	0.04 (0.20)	0.06 (0.23)	0.05 (0.21)	0.04 (0.20)
Household income:						
$15,000 or less	0.09 (0.28)	0.08 (0.27)	0.09 (0.28)	0.07 (0.26)	0.09 (0.28)	0.07 (0.25)
$15,000-$24,999	0.10 (0.31)	0.08 (0.28)	0.08 (0.28)	0.09 (0.28)	0.10 (0.29)	0.09 (0.29)
$25,000-$34,999	0.10 (0.30)	0.12 (0.32)	0.12 (0.32)	0.11 (0.31)	0.10 (0.30)	0.11 (0.31)
$35,000-$49,999	0.13 (0.34)	0.11 (0.32)	0.12 (0.32)	0.14 (0.34)	0.14 (0.35)	0.12 (0.33)
$50,000-$74,999	0.22 (0.41)	0.20 (0.40)	0.22 (0.42)	0.22 (0.41)	0.19 (0.39)	0.21 (0.41)
$75,000-$99,999	0.12 (0.32)	0.15 (0.36)	0.15 (0.35)	0.16 (0.37)	0.17 (0.37)	0.16 (0.36)
$100,000-$149,999	0.14 (0.34)	0.17 (0.38)	0.14 (0.34)	0.13 (0.34)	0.14 (0.35)	0.16 (0.36)
$150,000-$199,999	0.06 (0.24)	0.05 (0.22)	0.06 (0.23)	0.04 (0.19)	0.04 (0.20)	0.06 (0.23)
$200,000 and above	0.05 (0.21)	0.03 (0.18)	0.04 (0.19)	0.05 (0.21)	0.04 (0.20)	0.03 (0.17)
Educational attainment:						
No high school diploma	0.04 (0.19)	0.04 (0.19)	0.04 (0.19)	0.04 (0.18)	0.03 (0.17)	0.03 (0.17)
High school diploma	0.17 (0.38)	0.18 (0.38)	0.14 (0.35)	0.15 (0.36)	0.15 (0.36)	0.18 (0.38)
Associate's degree	0.44 (0.50)	0.44 (0.50)	0.47 (0.50)	0.48 (0.50)	0.46 (0.50)	0.44 (0.50)
Bachelor's degree	0.21 (0.41)	0.20 (0.40)	0.21 (0.41)	0.20 (0.40)	0.22 (0.41)	0.21 (0.41)
Graduate degree	0.14 (0.35)	0.15 (0.36)	0.14 (0.34)	0.13 (0.34)	0.14 (0.35)	0.14 (0.35)
Age:						
18-24	0.05 (0.22)	0.07 (0.26)	0.05 (0.22)	0.08 (0.27)	0.08 (0.27)	0.06 (0.24)
25-29	0.09 (0.29)	0.10 (0.30)	0.09 (0.29)	0.10 (0.30)	0.09 (0.29)	0.09 (0.29)
30-34	0.15 (0.35)	0.13 (0.34)	0.14 (0.35)	0.15 (0.35)	0.13 (0.34)	0.15 (0.36)
35-39	0.08 (0.27)	0.08 (0.27)	0.09 (0.28)	0.08 (0.28)	0.09 (0.29)	0.08 (0.28)
40-44	0.07 (0.26)	0.08 (0.27)	0.07 (0.25)	0.08 (0.28)	0.07 (0.25)	0.07 (0.25)
45-49	0.06 (0.24)	0.06 (0.24)	0.08 (0.27)	0.05 (0.23)	0.08 (0.27)	0.06 (0.24)
50-54	0.06 (0.25)	0.07 (0.26)	0.06 (0.24)	0.07 (0.25)	0.05 (0.21)	0.06 (0.24)
55-59	0.09 (0.28)	0.09 (0.28)	0.11 (0.31)	0.09 (0.29)	0.10 (0.30)	0.09 (0.29)
60-64	0.11 (0.31)	0.09 (0.29)	0.11 (0.31)	0.10 (0.29)	0.08 (0.27)	0.10 (0.31)
65-69	0.09 (0.28)	0.08 (0.27)	0.08 (0.27)	0.08 (0.28)	0.11 (0.31)	0.10 (0.30)
70-74	0.09 (0.28)	0.09 (0.29)	0.08 (0.28)	0.08 (0.27)	0.07 (0.26)	0.07 (0.26)
75+	0.06 (0.24)	0.05 (0.21)	0.04 (0.20)	0.04 (0.19)	0.05 (0.22)	0.06 (0.23)
Employment status:						
Employed	0.61 (0.49)	0.63 (0.48)	0.60 (0.49)	0.63 (0.48)	0.66 (0.48)	0.61 (0.49)
Pre-treatment attitudes:						
Political ideology	3.86 (1.58)	3.91 (1.62)	3.94 (1.60)	3.93 (1.60)	4.02 (1.59)	4.10 (1.57)
Partisanship	3.64 (2.02)	3.62 (2.04)	3.70 (2.06)	3.69 (2.13)	3.77 (2.11)	3.82 (2.08)
Vaccine nationalism	2.19 (1.02)	2.21 (1.08)	2.17 (1.04)	2.27 (1.08)	2.24 (1.08)	2.22 (1.03)
Nationalism	4.00 (1.07)	4.04 (1.08)	4.04 (1.06)	3.99 (1.08)	4.05 (1.04)	4.09 (1.04)
Patriotism	3.98 (1.04)	4.07 (1.03)	4.08 (0.99)	3.99 (1.01)	4.09 (1.00)	4.10 (1.01)
Altruism	4.16 (0.78)	4.20 (0.77)	4.16 (0.79)	4.19 (0.75)	4.10 (0.82)	4.17 (0.76)

S3 Supplementary analyses, tables and figures

Table S2: Demographic characteristics in U.S. adult population and survey samples

	**Population**	**Unweighted sample**		**Weighted sample**	
		Lucid	NORC	Lucid	NORC
Sex:					
Female	0.52	0.54	0.51	0.52	0.52
Male	0.48	0.46	0.49	0.48	0.48
Region:					
South	0.38	0.38	0.33	0.38	0.38
West	0.24	0.22	0.26	0.24	0.24
Midwest	0.21	0.19	0.27	0.21	0.21
Northeast	0.18	0.22	0.14	0.18	0.17
Race/ethnicity:					
White	0.64	0.62	0.63	0.64	0.62
Hispanic	0.17	0.16	0.17	0.17	0.17
Black	0.12	0.13	0.12	0.12	0.12
AAPI	0.06	0.06	0.03	0.06	0.06
Other	0.01	0.02	0.05	0.01	0.03
Household income:					
$15,000 or less	0.09	0.13	0.08	0.09	0.09
$15,000-$24,999	0.09	0.11	0.09	0.09	0.10
$25,000-$34,999	0.08	0.10	0.11	0.08	0.11
$35,000-$49,999	0.12	0.11	0.13	0.12	0.12
$50,000-$74,999	0.17	0.15	0.21	0.17	0.20
$75,000-$99,999	0.12	0.11	0.15	0.12	0.14
$100,000-$149,999	0.15	0.14	0.15	0.15	0.14
$150,000-$199,999	0.08	0.05	0.05	0.08	0.06
$200,000 and above	0.10	0.04	0.04	0.10	0.04
Educational attainment:					
No high school diploma	0.10	0.04	0.03	0.09	0.10
*High school diploma	0.45	0.39	-	0.45	-
*Associate's degree	0.10	0.08	-	0.10	-
Bachelor's degree	0.22	0.24	0.21	0.22	0.20
Graduate degree	0.13	0.25	0.14	0.13	0.14
Age:					
18-24	0.12	0.11	0.07	0.12	0.11
25-29	0.10	0.06	0.09	0.10	0.09
30-34	0.09	0.10	0.14	0.09	0.09
35-39	0.09	0.10	0.08	0.09	0.09
40-44	0.08	0.09	0.07	0.08	0.08
45-49	0.08	0.05	0.07	0.08	0.07
50-54	0.08	0.07	0.06	0.08	0.07
55-59	0.08	0.09	0.10	0.08	0.10
60-64	0.08	0.12	0.10	0.08	0.08
65-69	0.07	0.08	0.09	0.07	0.09
70+	0.14	0.12	0.13	0.14	0.13

Notes: Population proportions from 2020 U.S. Census and Current Population survey. *NORC combines HS graduates with "some college" and/or further vocational education/training together with Associate's degree holders; population benchmarks for the sub-groups created by NORC are unavailable.

S3.1 Comparisons between weighted and un-weighted estimates

Figure S3: Estimated marginal means in vaccine recipient conjoint with and without survey weights

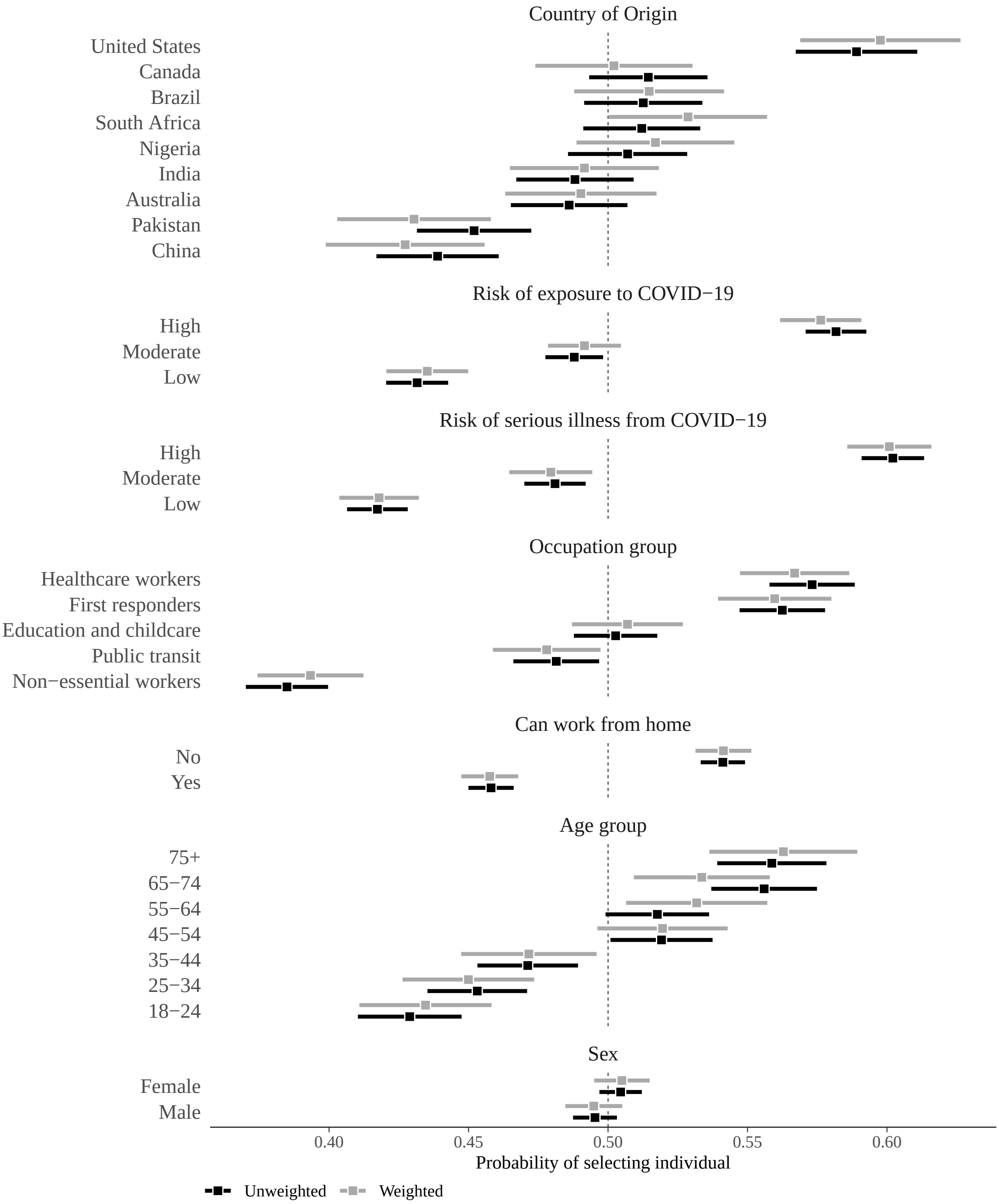

Notes: Marginal means showing mass preferences for COVID-19 vaccine distribution by characteristics of potential vaccine recipients. Point estimates and 95% confidence intervals estimated via OLS regression with robust standard errors clustered at respondent level to correct for within-respondent clustering. Lucid survey of U.S. adults fielded in April 2021 (N = 1,751 respondents x 5 pairings x 2 agreements per pair = 17,510 observations).

Figure S4: Estimated AMCEs in vaccine recipient conjoint with and without survey weights

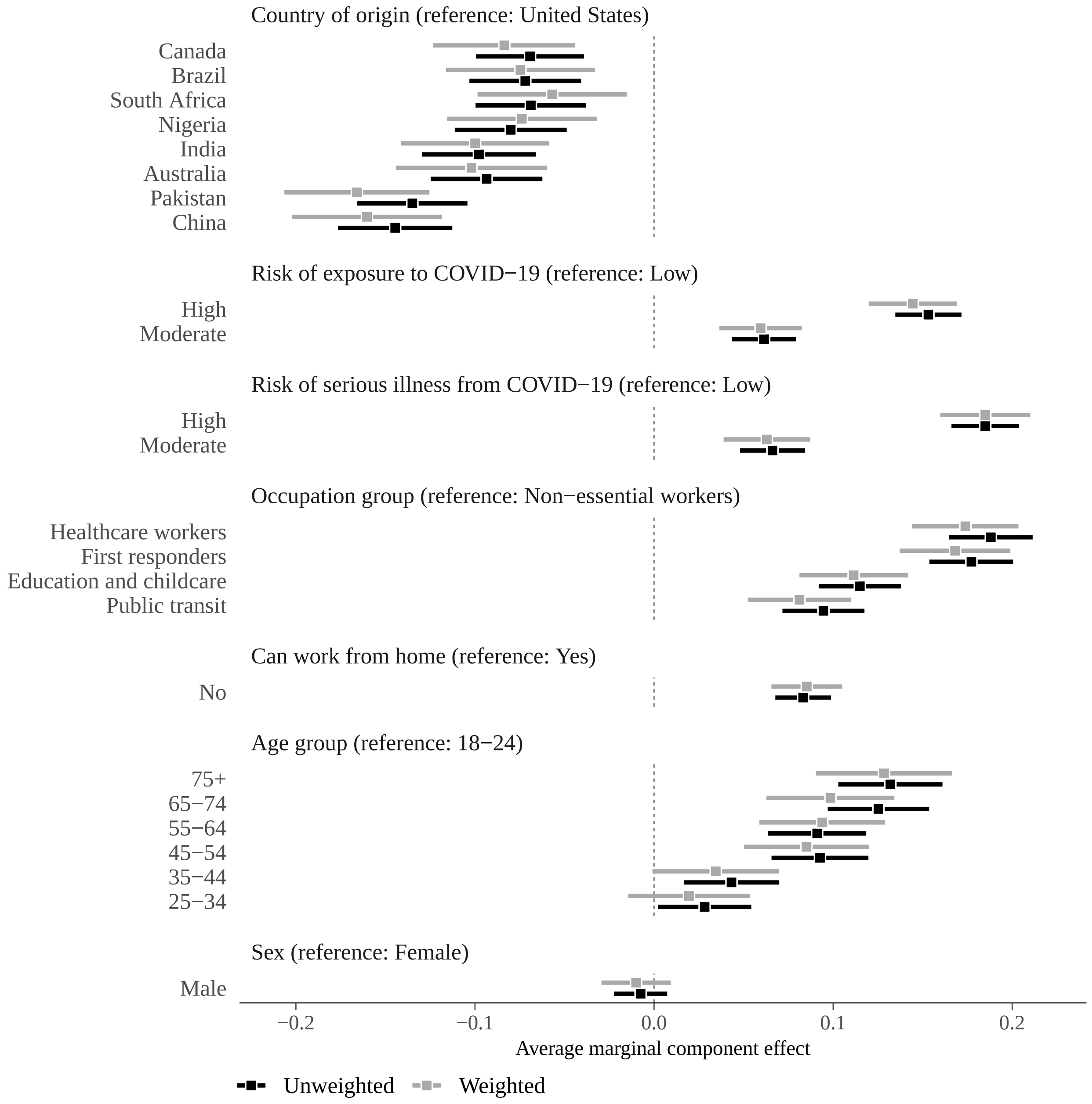

Notes: Effects of randomly assigned background characteristics on public support for distribution of COVID-19 vaccines to potential recipients. Point estimates and 95% confidence intervals estimated via OLS regression with robust standard errors clustered at respondent level to correct for within-respondent clustering. Lucid survey of U.S. adults fielded in April 2021 (N = 1,751 respondents x 5 pairings x 2 agreements per pair = 17,510 observations).

Figure S5: Estimated marginal means in international agreement conjoint (Lucid) with and without survey weights

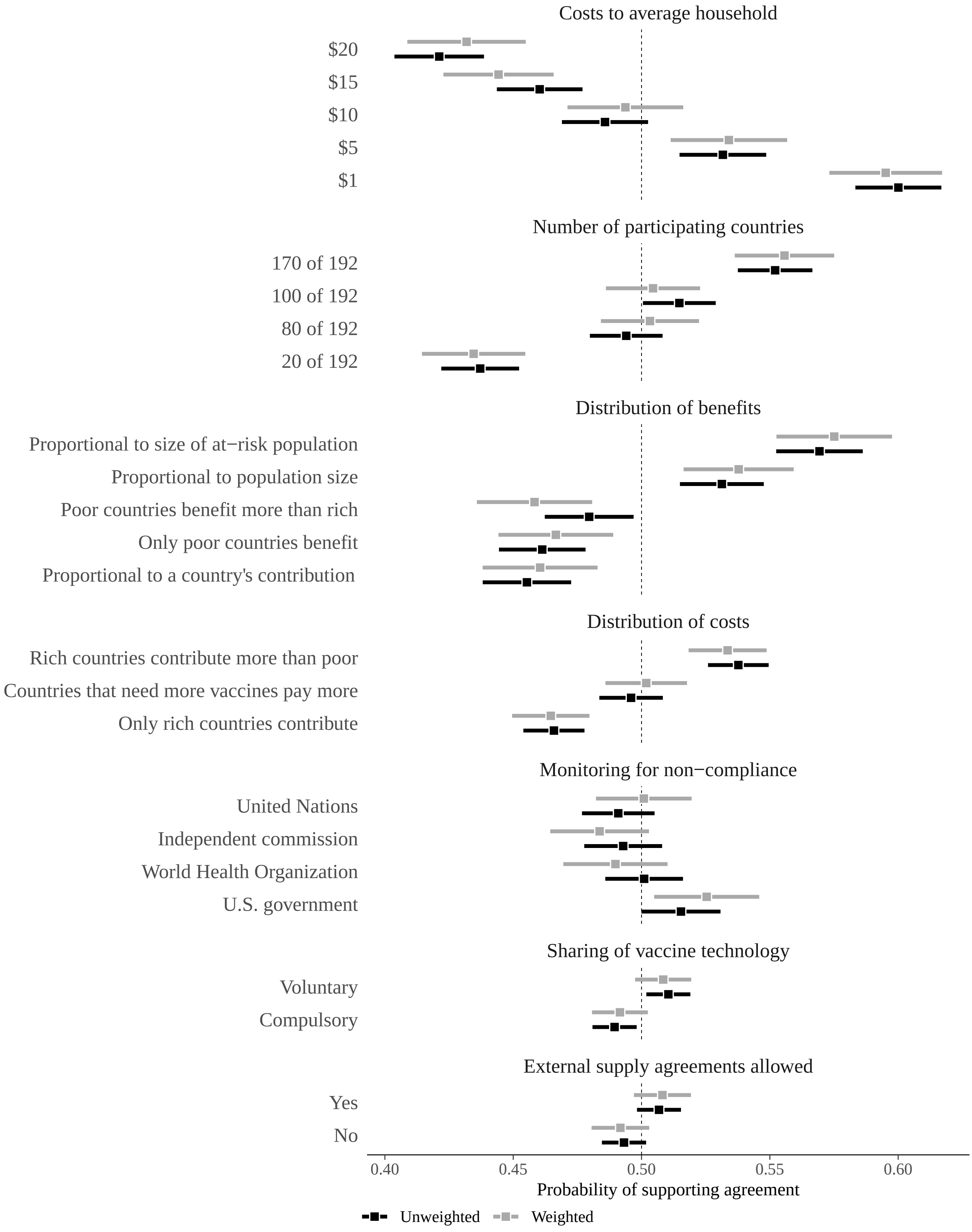

Notes: Marginal means showing public support for international cooperation on COVID-19 vaccine distribution across each randomly assigned institutional design feature. Point estimates and 95% confidence intervals estimated via OLS regression with robust standard errors clustered at respondent level to correct for within-respondent clustering. Lucid survey of U.S. adults fielded in April 2021 (N = 1,751 respondents x 4 pairings x 2 agreements per pair = 14,008 observations).

Figure S6: Estimated AMCEs in international agreement conjoint (Lucid) with and without survey weights

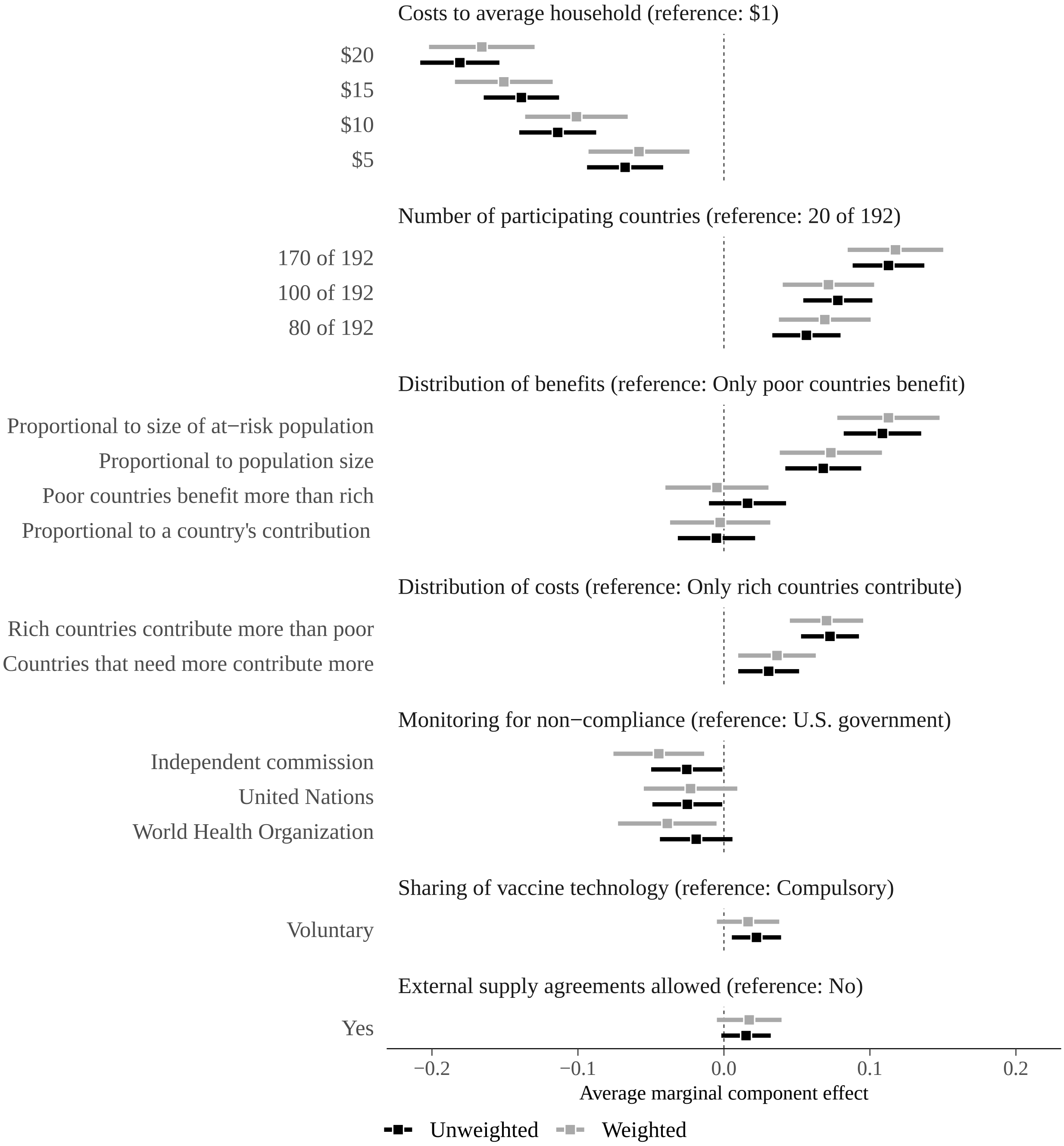

Notes: Effects of institutional design features on public support for international cooperation on COVID-19 vaccine distribution. Point estimates and 95% confidence intervals estimated via OLS regression with robust standard errors clustered at respondent level to correct for within-respondent clustering. Lucid survey of U.S. adults fielded in April 2021 (N = 1,751 respondents x 5 pairings x 2 agreements per pair = 17,510 observations).

Figure S7: Estimated marginal means in international agreement conjoint (NORC) with and without survey weights

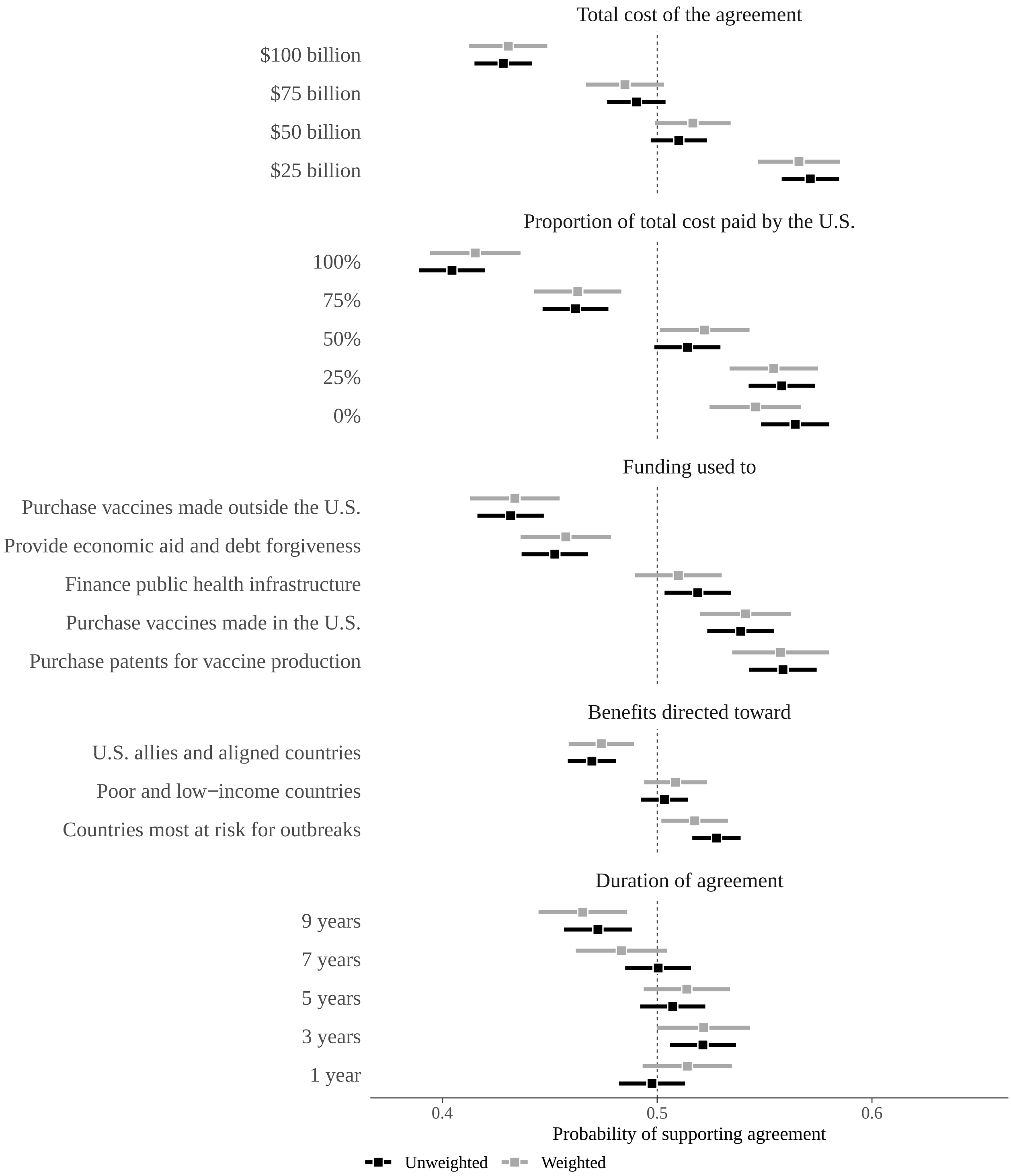

Notes: Marginal means showing public support for international cooperation on COVID-19 vaccine distribution across each randomly assigned institutional design feature. Point estimates and 95% confidence intervals estimated via OLS regression with robust standard errors clustered at respondent level to correct for within-respondent clustering. NORC/Amerispeak survey of U.S. adults fielded in September/October 2021 (N = 4,214 respondents x 2 pairings x 2 agreements per pair = 16,856 observations).

Figure S8: Estimated AMCEs in international agreement conjoint (NORC) with and without survey weights

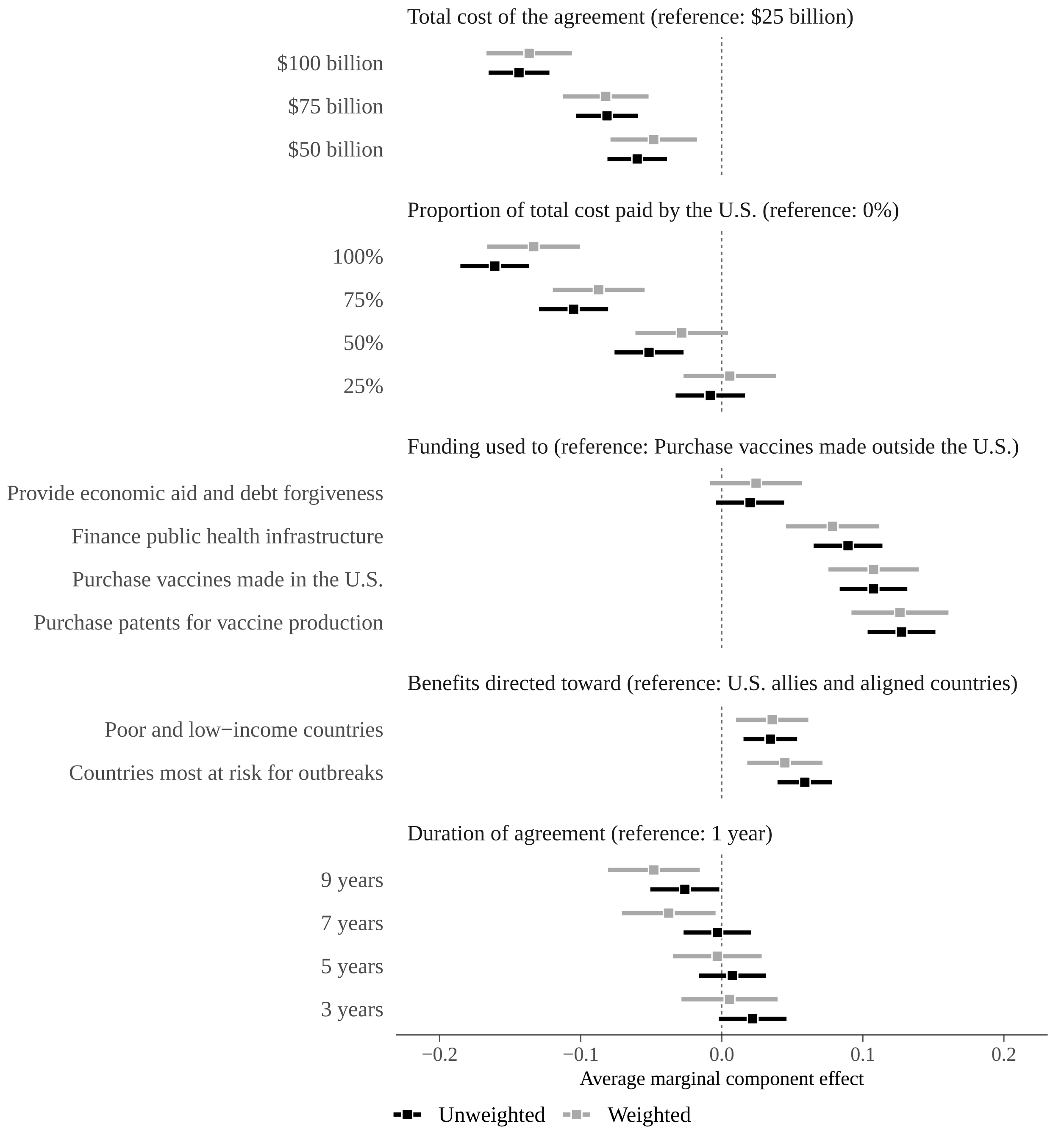

Notes: Effects of institutional design features on public support for international cooperation on COVID-19 vaccine distribution. Point estimates and 95% confidence intervals estimated via OLS regression with robust standard errors clustered at respondent level to correct for within-respondent clustering. NORC/Amerispeak survey of U.S. adults fielded in September/October 2021 (N = 4,214 respondents x 2 pairings x 2 agreements per pair = 16,856 observations).

Figure S9: Estimated ATEs in persuasion experiment with and without survey weights

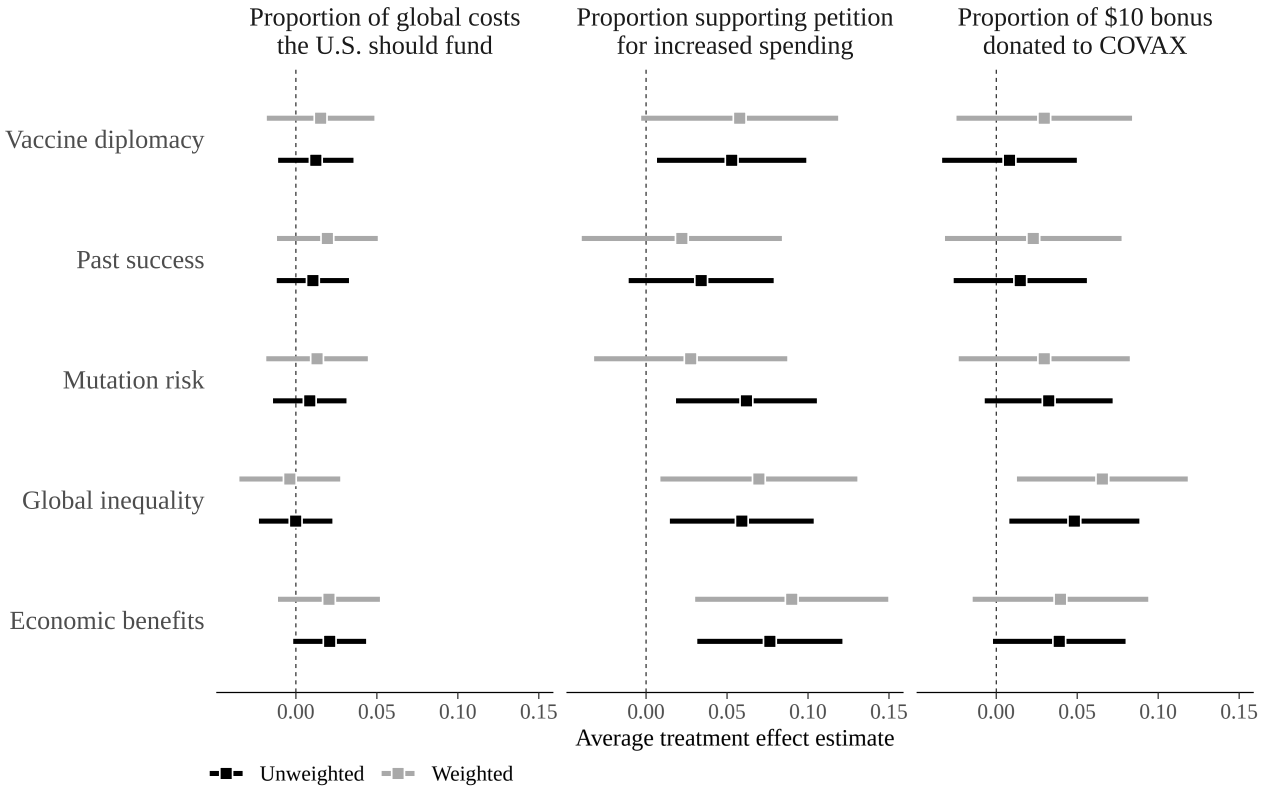

Notes: Effects of persuasive messaging strategies on support for government spending, willingness to petition U.S. Congress, and charitable contributions to the COVID-19 Vaccines Global Access Initiative (COVAX). Covariate-adjusted estimates (and 95% confidence intervals) from OLS regression with HC2 robust standard errors. Pre-treatment covariates: age, sex, education level, race/ethnicity, region, employment status, household income, partisanship, conservatism, altruism, vaccine nationalism, and nationalism/patriotism.

Table S3: Estimated ATEs in persuasion experiment with and without survey weights

	Proportion of global costs the U.S. should fund		Proportion supporting petition for increased spending		Proportion of $10 bonus donated to COVAX	
**Covariate-adjusted Estimator:**						
	Unweighted	*Weighted*	*Unweighted*	*Weighted*	*Unweighted*	*Weighted*
Economic benefits	0.02 (0.01)	0.02 (0.02)	0.08 (0.02)*	0.09 (0.03)*	0.04 (0.02)	0.04 (0.03)
Global inequality	-0.00 (0.01)	-0.00 (0.02)	0.06 (0.02)*	0.07 (0.03)	0.05 (0.02)	0.07 (0.03)
Mutation risk	0.01 (0.01)	0.01 (0.02)	0.06 (0.02)*	0.03 (0.03)	0.03 (0.02)	0.03 (0.03)
Past success	0.01 (0.01)	0.02 (0.02)	0.03 (0.02)	0.02 (0.03)	0.01 (0.02)	0.02 (0.03)
Vaccine diplomacy	0.01 (0.01)	0.02 (0.02)	0.05 (0.02)	0.06 (0.03)	0.01 (0.02)	0.03 (0.03)
**Difference-in-Means Estimator:**						
	Unweighted	*Weighted*	*Unweighted*	*Weighted*	*Unweighted*	*Weighted*
Economic benefits	0.02 (0.01)	0.02 (0.02)	0.06 (0.03)	0.08 (0.04)	0.02 (0.02)	0.05 (0.03)
Global inequality	-0.02 (0.01)	-0.02 (0.02)	0.02 (0.03)	0.03 (0.03)	0.04 (0.02)	0.06 (0.03)
Mutation risk	-0.00 (0.01)	0.01 (0.02)	0.05 (0.03)	0.03 (0.03)	0.02 (0.02)	0.05 (0.03)
Past success	0.00 (0.01)	0.02 (0.02)	0.02 (0.03)	0.03 (0.04)	-0.01 (0.02)	0.01 (0.03)
Vaccine diplomacy	0.00 (0.01)	0.02 (0.02)	0.03 (0.03)	0.07 (0.04)	-0.02 (0.02)	0.03 (0.03)

Notes: Effects of persuasive messaging strategies on support for government spending, willingness to petition U.S. Congress, and charitable contributions to the COVID-19 Vaccines Global Access Initiative (COVAX). Point estimates and standard errors with and without survey weights. All estimates from OLS regressions with HC2 robust standard errors. Covariate-adjusted estimator includes pre-treatment covariates: age, sex, education level, race/ethnicity, region, employment status, household income, partisanship, conservatism, altruism, vaccine nationalism, and nationalism/patriotism. *$P < 0.05$ after adjusting for multiple comparisons using the Benjamini and Hochberg (1995) method to control the false discovery rate.

S3.2 Heterogeneity in vaccine recipient conjoint by country of origin

Figure S10: Estimated conditional marginal means in vaccine recipient conjoint by non-U.S. v. U.S. origin

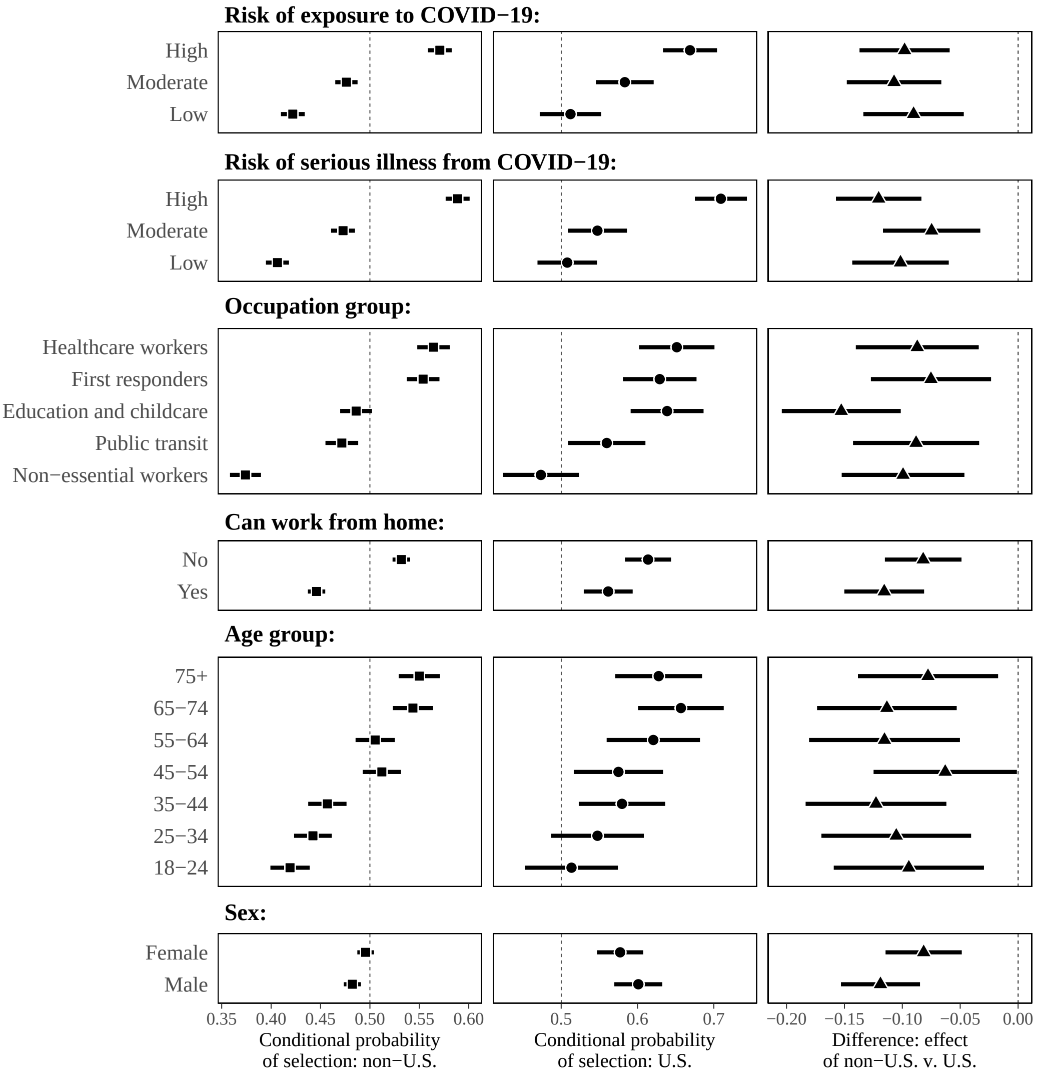

Notes: Sub-group estimates showing conditional marginal means when country of origin is non-U.S., U.S., and the differences. Point estimates and 95% confidence intervals estimated via OLS regression with robust standard errors clustered at respondent level to correct for within-respondent clustering. Lucid survey of U.S. adults fielded in April 2021 (N = 1,751 respondents x 5 pairings x 2 agreements per pair = 17,510 observations).

Figure S11: Estimated conditional AMCEs in vaccine recipient conjoint by non-U.S. v. U.S. origin

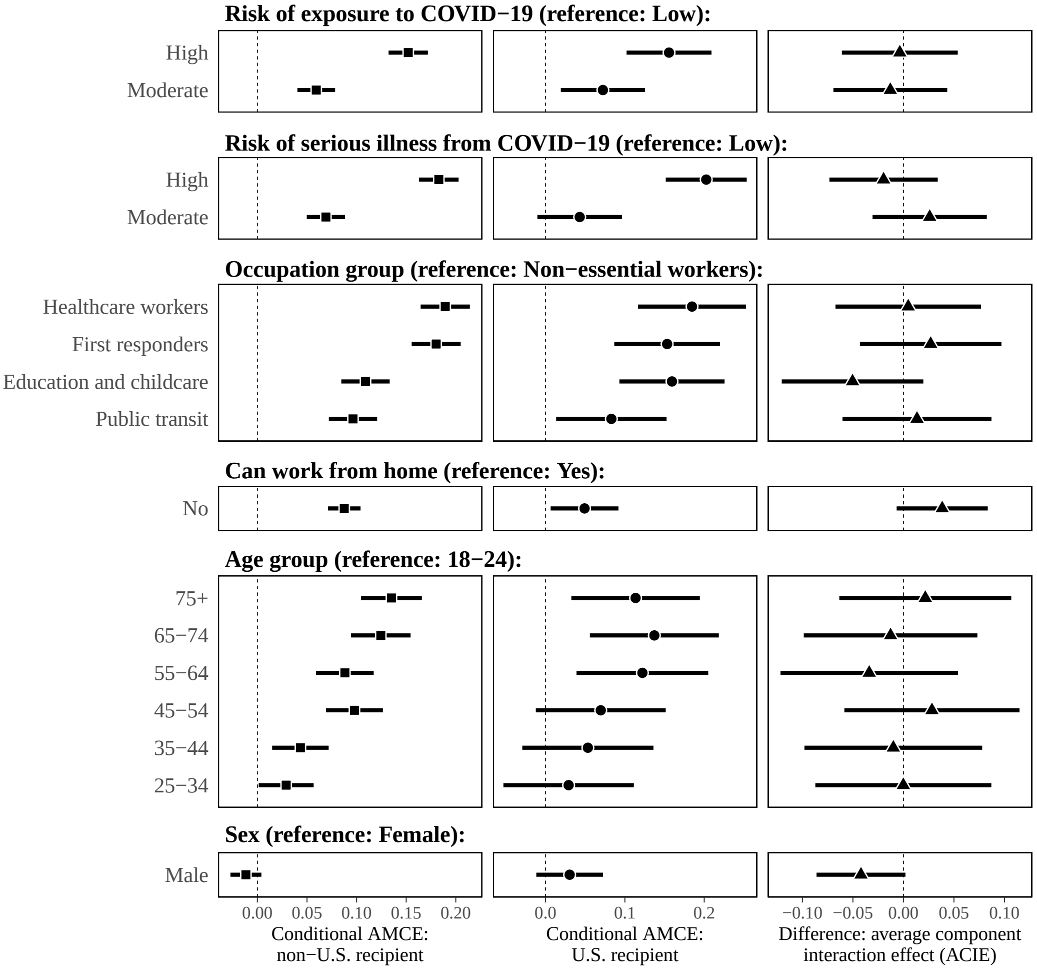

Notes: Sub-group estimates showing conditional AMCEs when country of origin is non-U.S., U.S., and the differences. Point estimates and 95% confidence intervals estimated via OLS regression with robust standard errors clustered at respondent level to correct for within-respondent clustering. Lucid survey of U.S. adults fielded in April 2021 (N = 1,751 respondents x 5 pairings x 2 agreements per pair = 17,510 observations).

Figure S12: Estimated marginal means for paired comparisons between potential vaccine recipients from the U.S. and another country

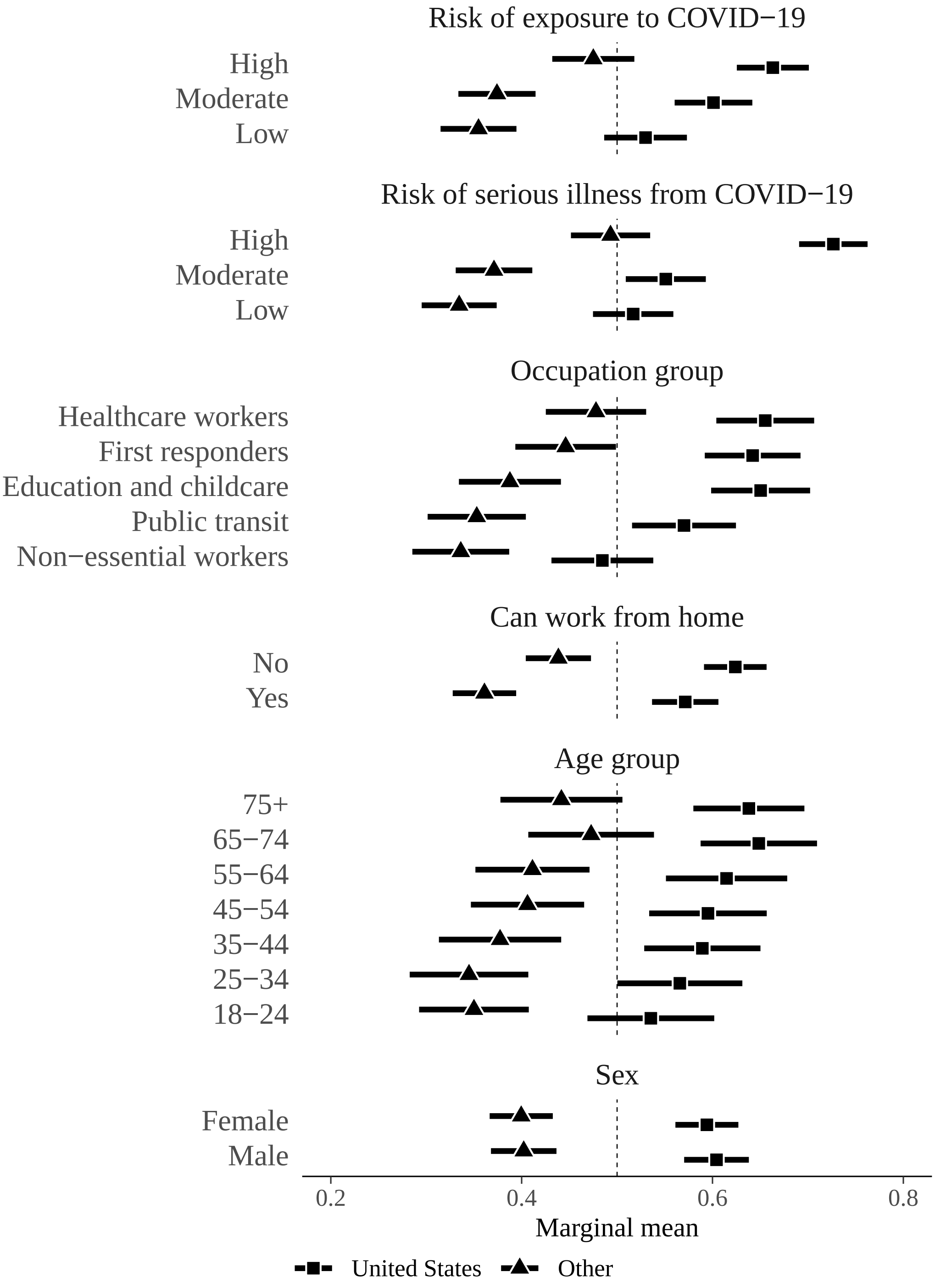

Notes: Marginal means showing mass preferences for COVID-19 vaccine distribution by characteristics of potential vaccine recipients. The sample is restricted to the subset of randomized profiles that forced respondents to make pairwise comparisons between the United States and another country (N = 3,418 observations). Point estimates and 95% confidence intervals estimated via OLS regression with robust standard errors clustered at respondent level to correct for within-respondent clustering.

S3.3 Support for international agreements in NORC conjoint and donations to COVAX

Table S4: Likelihood of voting for agreement as a function of amount donated to COVAX and randomized features

**Amount donated to COVAX (reference: ≤ $5.00)**	
I(Donated > $5.00)	0.10 (0.01)*
**Total cost of the agreement (reference: $25 billion)**	
$50 billion	-0.03 (0.01)*
$75 billion	-0.04 (0.01)*
$100 billion	-0.06 (0.01)*
**Proportion of total cost paid by the U.S. (reference: 0%)**	
25%	-0.02 (0.01)*
50%	-0.04 (0.01)*
75%	-0.07 (0.01)*
100%	-0.10 (0.01)*
**Funding used to (reference: Purchase vaccines made outside the U.S.)**	
Purchase vaccines made in the U.S.	0.06 (0.01)*
Provide economic aid and debt forgiveness	0.01 (0.01)
Finance public health infrastructure	0.04 (0.01)*
Purchase patents for vaccine production	0.06 (0.01)*
**Benefits directed toward (reference: U.S. allies and aligned countries)**	
Poor and low-income countries	0.02 (0.01)*
Countries most at risk for outbreaks	0.02 (0.01)*
**Duration of agreement (reference: 1 year)**	
3 years	0.02 (0.01)*
5 years	0.01 (0.01)
7 years	0.00 (0.01)
9 years	-0.01 (0.01)*

Notes: Estimates from OLS regression of likelihood of voting for agreement on indicator for respondents' that donated more than $5 to COVAX (the sample median) and randomized features, with robust standard errors clustered at respondent level to correct for within-respondent clustering. Outcome measure: 5-point scale standardized to $[0, 1]$ interval (0 = "Definitely vote against",...,0.5 = "Neither in favor nor against", ...,1 = "Definitely vote in favor"). NORC/Amerispeak survey of U.S. adults fielded in September/October 2021 (N = 4,214 respondents x 2 pairings x 2 agreements per pair = 16,856 observations). $*P < 0.05$

Figure S13: Estimated marginal means in international agreement conjoint (NORC) by donation amount

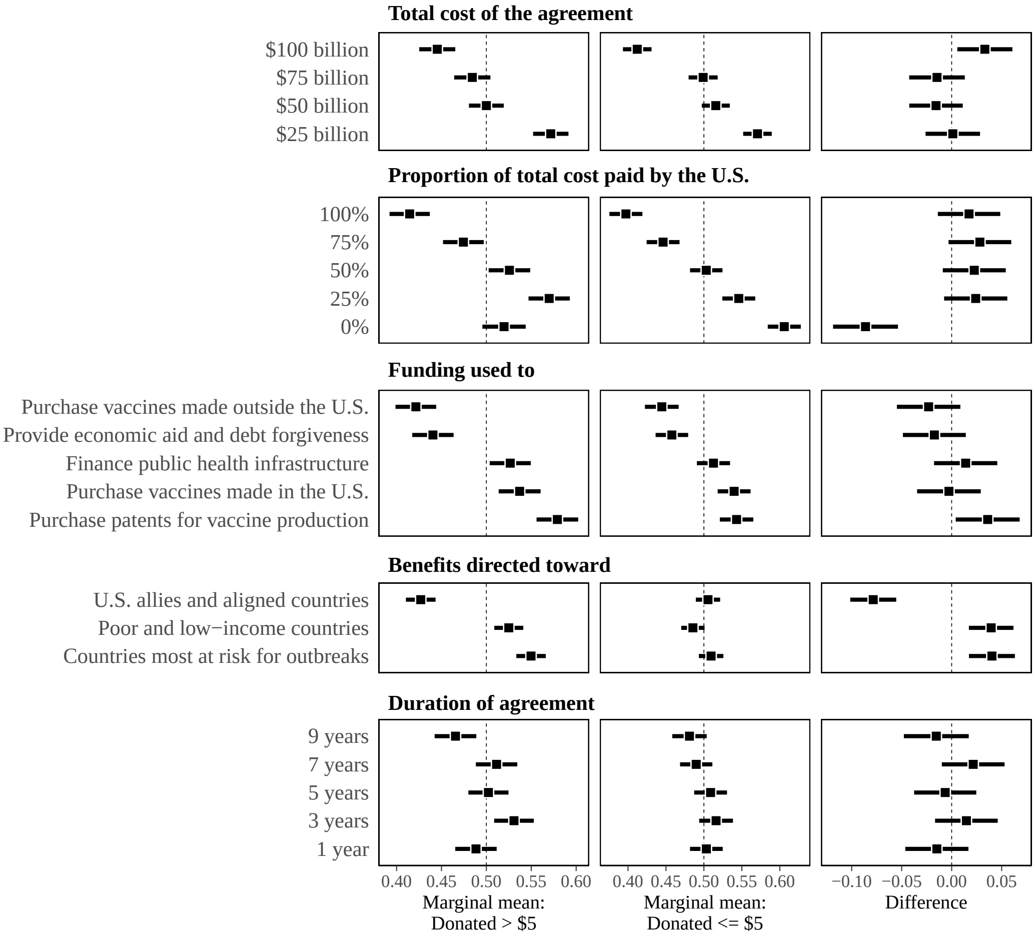

Notes: Sub-group estimates showing marginal means among those donating more than $5 to COVAX (sample median), $5 or less, and the differences. Point estimates and 95% confidence intervals estimated via OLS regression with robust standard errors clustered at respondent level to correct for within-respondent clustering. NORC/Amerispeak survey of U.S. adults fielded in September/October 2021 (N = 4,214 respondents x 2 pairings x 2 agreements per pair = 16,856 observations).

Figure S14: Estimated AMCEs in international agreement conjoint (NORC) by donation amount

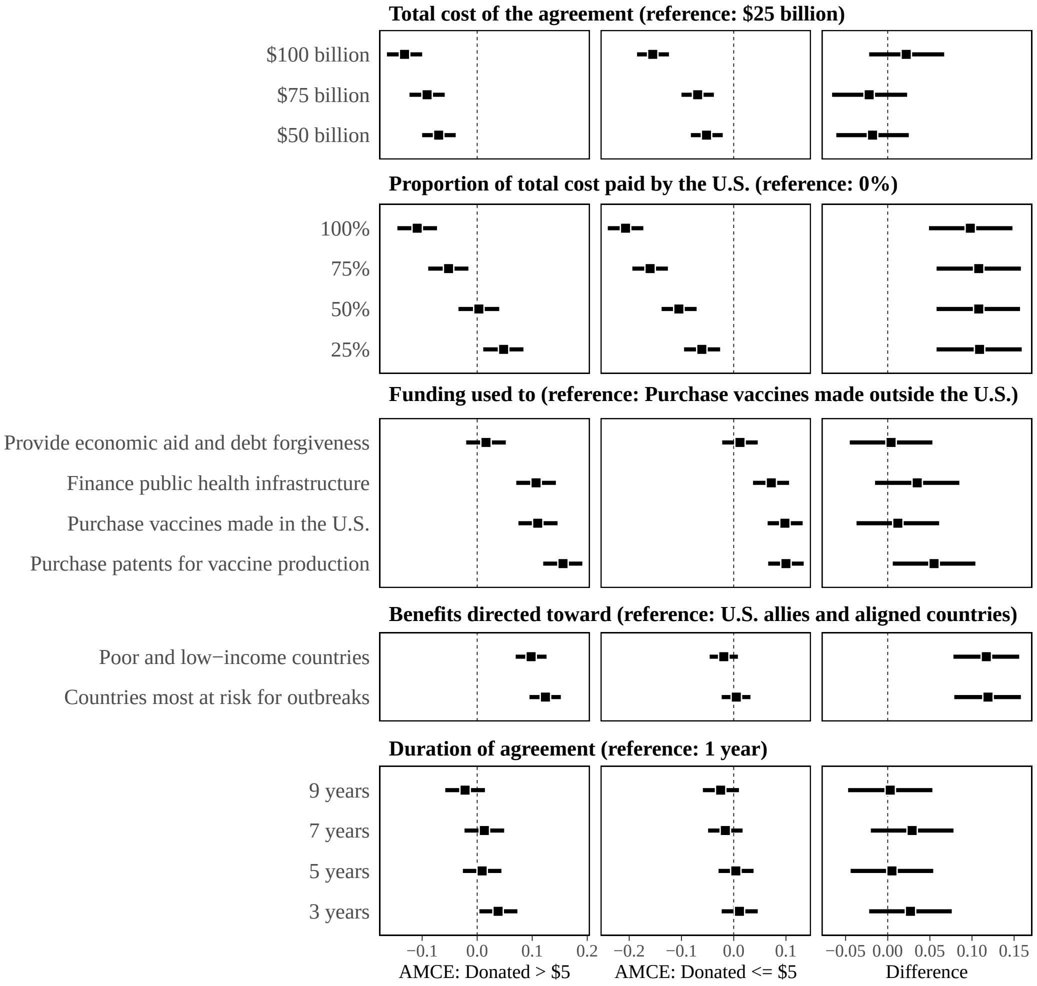

Notes: Sub-group estimates showing AMCEs among those donating more than $5 to COVAX (sample median), $5 or less, and the differences. Point estimates and 95% confidence intervals estimated via OLS regression with robust standard errors clustered at respondent level to correct for within-respondent clustering. NORC/Amerispeak survey of U.S. adults fielded in September/October 2021 (N = 4,214 respondents x 2 pairings x 2 agreements per pair = 16,856 observations).

S3.4 Estimated marginal means and AMCEs on ordinal outcome measures

Figure S15: Estimated marginal means on ordinal measure of support for potential vaccine recipients, with and without survey weights

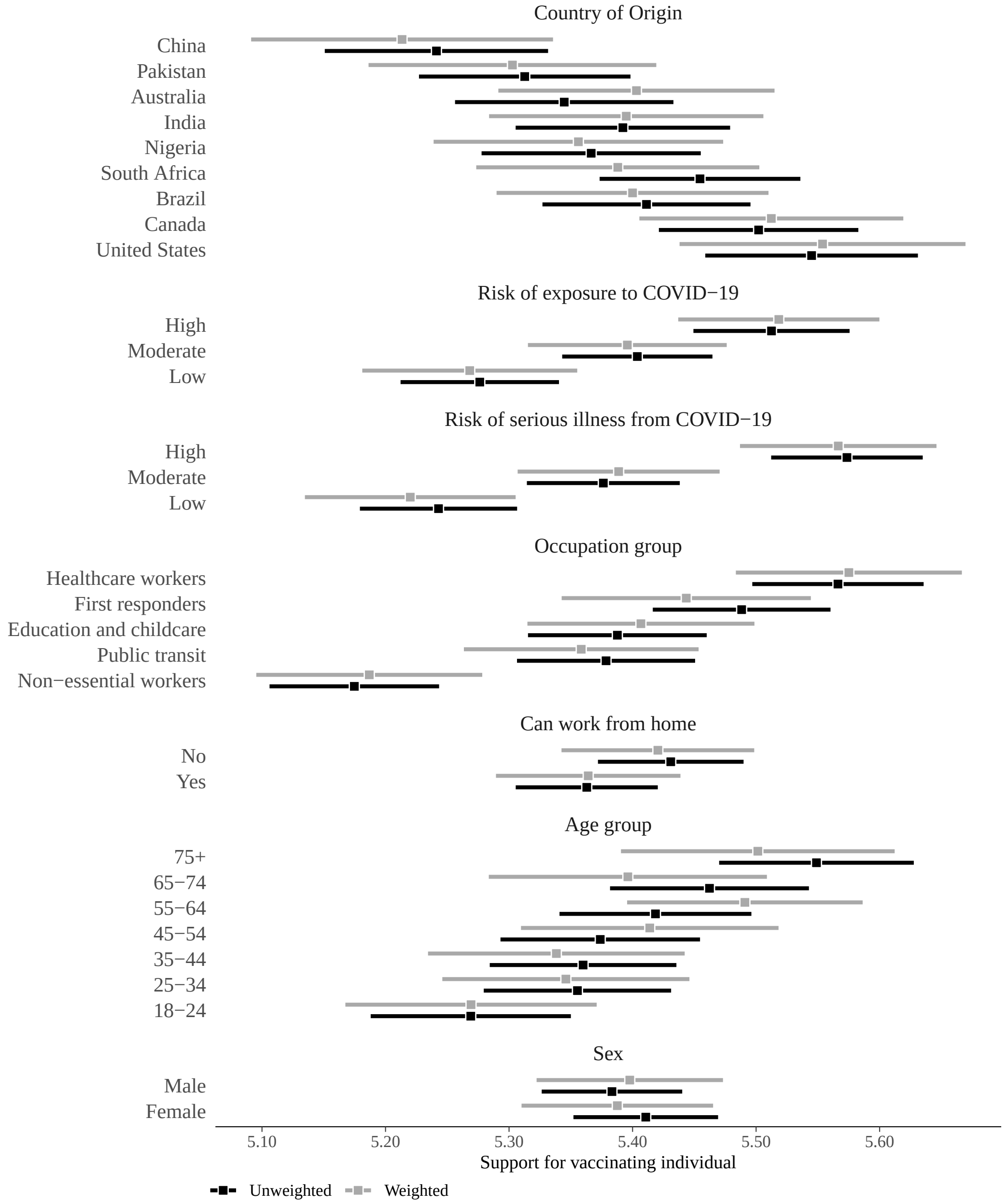

Notes: Marginal means showing mass preferences for COVID-19 vaccine distribution by characteristics of potential vaccine recipients. Outcome measure: 1 = "Definitely should not receive vaccine", ...,4 = "Neutral", ..., 7="Definitely should receive vaccine". Point estimates and 95% confidence intervals estimated via OLS regression with robust standard errors clustered at respondent level to correct for within-respondent clustering. Lucid survey of U.S. adults fielded in April 2021 (N = 1,751 respondents x 5 pairings x 2 agreements per pair = 17,510 observations).

Figure S16: Estimated AMCEs on ordinal measure of support for potential vaccine recipients, with and without survey weights

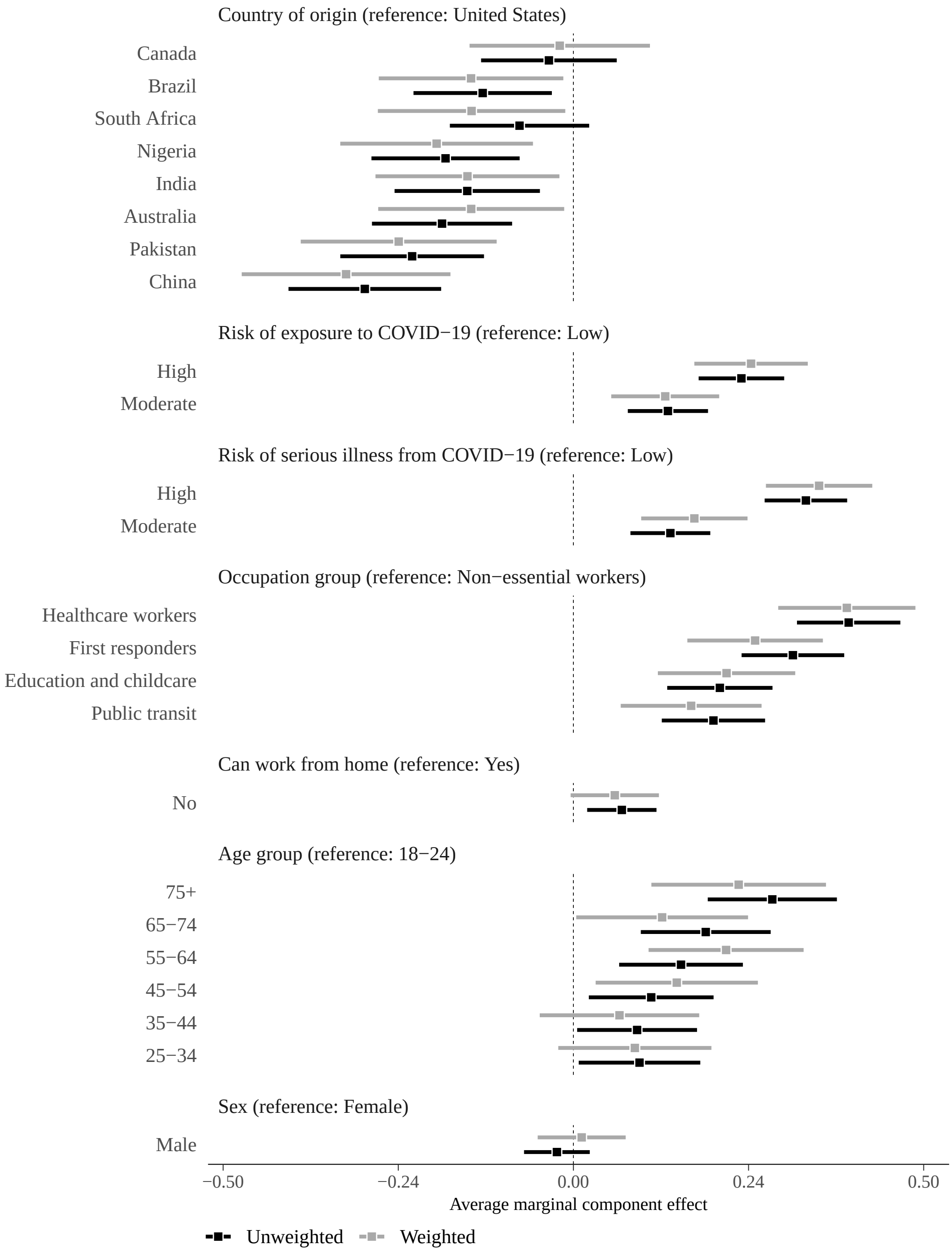

Notes: Effects of randomly assigned background characteristics on public support for distribution of COVID-19 vaccines to potential recipients. Outcome measure: 7-point scales from 1 = "Definitely should not receive vaccine", ...,4 = "Neutral", ..., 7="Definitely should receive vaccine". Point estimates and 95% confidence intervals estimated via OLS regression with robust standard errors clustered at respondent level to correct for within-respondent clustering. Lucid survey of U.S. adults fielded in April 2021 (N = 1,751 respondents x 5 pairings x 2 agreements per pair = 17,510 observations).

Figure S17: Estimated marginal means on likelihood of voting for international agreement in Lucid conjoint, with and without survey weights

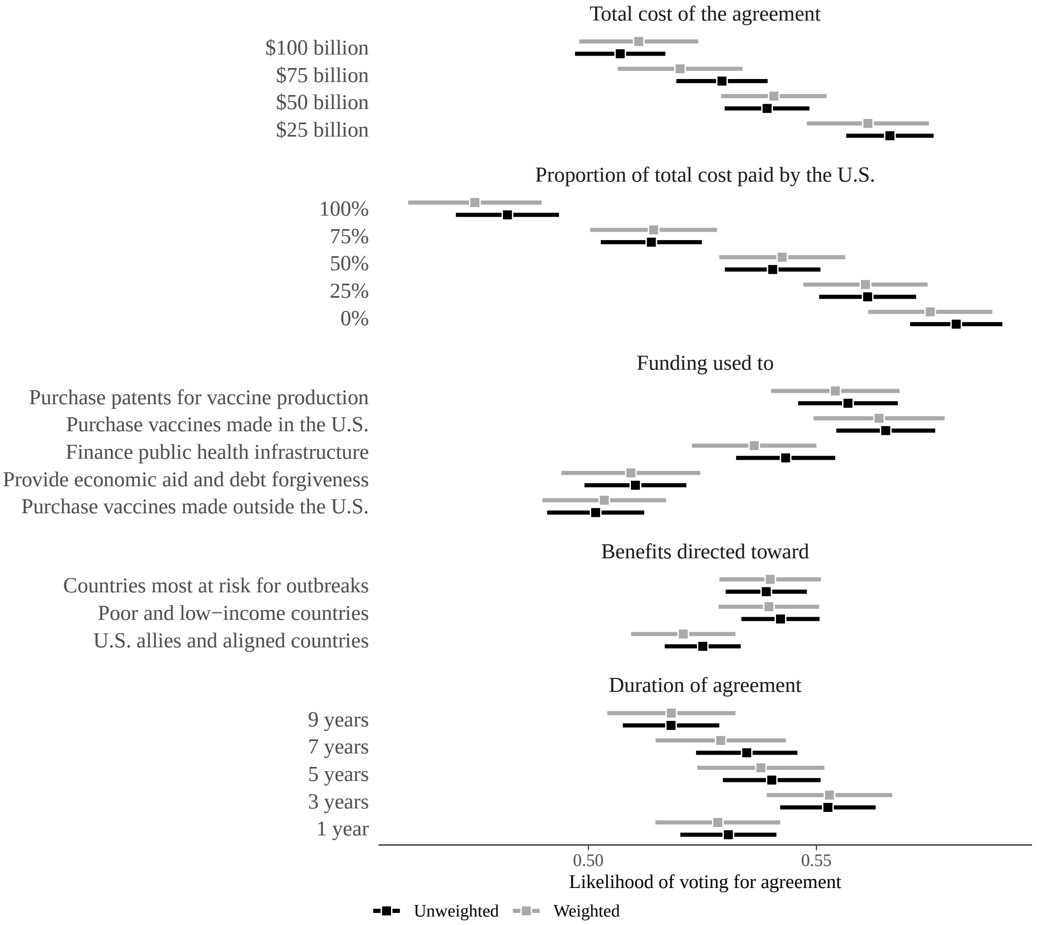

Notes: Marginal means showing public support for international cooperation on COVID-19 vaccine distribution across each randomly assigned institutional design feature. Outcome measure: 7-point scale standardized to $[0, 1]$ interval (0 = "Definitely vote against",...,0.5 = "Neither in favor nor against", ...,1 = "Definitely vote in favor"). Point estimates and 95% confidence intervals estimated via OLS regression with robust standard errors clustered at respondent level to correct for within-respondent clustering. Lucid survey of U.S. adults fielded in April 2021 (N = 1,751 respondents x 4 pairings x 2 agreements per pair = 14,008 observations).

Figure S18: Estimated AMCEs on likelihood of voting for international agreement in Lucid conjoint, with and without survey weights

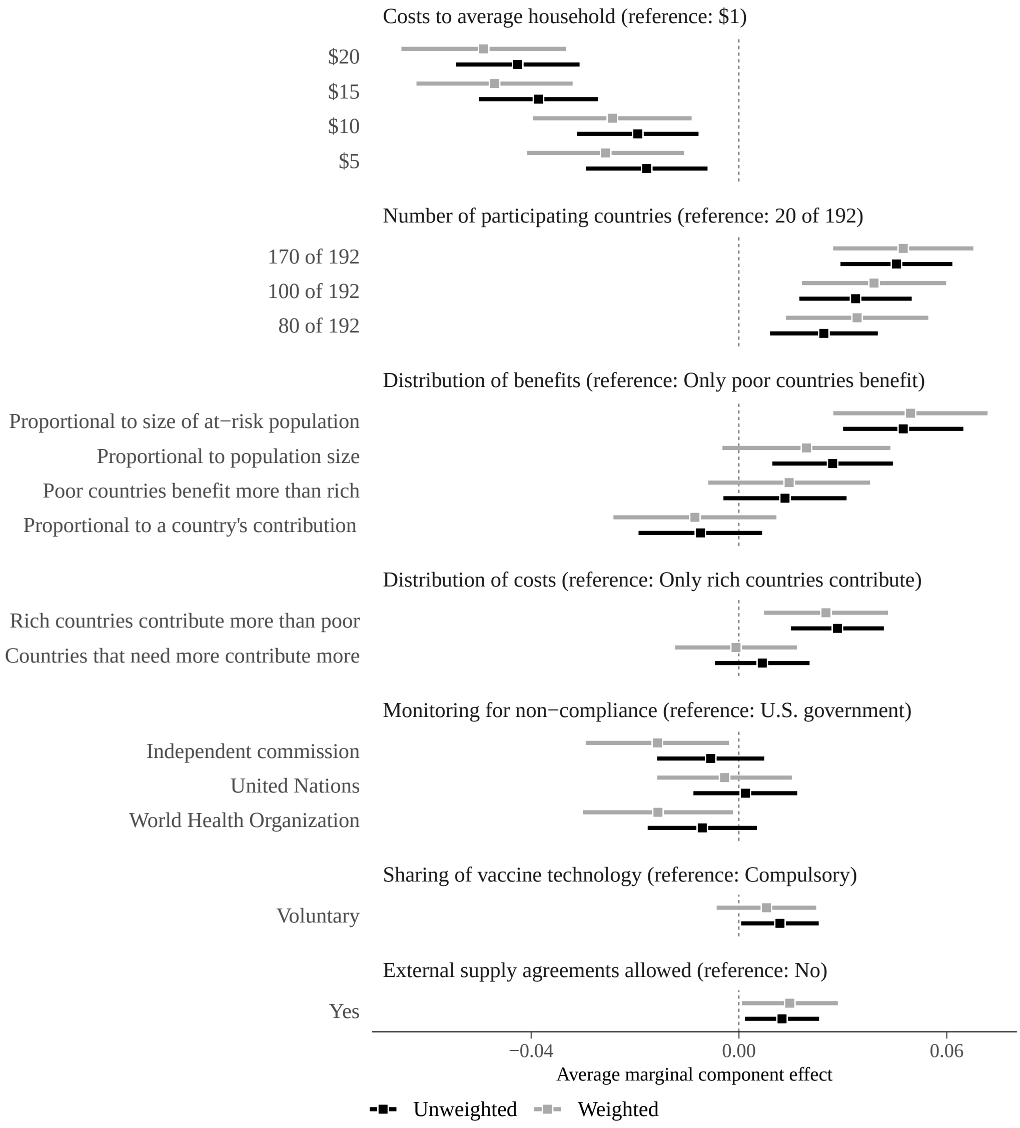

*Notes:*Effects of institutional design features on public support for international cooperation on COVID-19 vaccine distribution. Outcome measure: 7-point scale standardized to $[0, 1]$ interval (0 = “Definitely vote against”,...,0.5 = “Neither in favor nor against”, ...,1 = “Definitely vote in favor”). Point estimates and 95% confidence intervals estimated via OLS regression with robust standard errors clustered at respondent level to correct for within-respondent clustering. Lucid survey of U.S. adults fielded in April 2021 (N = 1,751 respondents x 5 pairings x 2 agreements per pair = 17,510 observations).

Figure S19: Estimated marginal means on likelihood of voting for international agreement in NORC conjoint, with and without survey weights

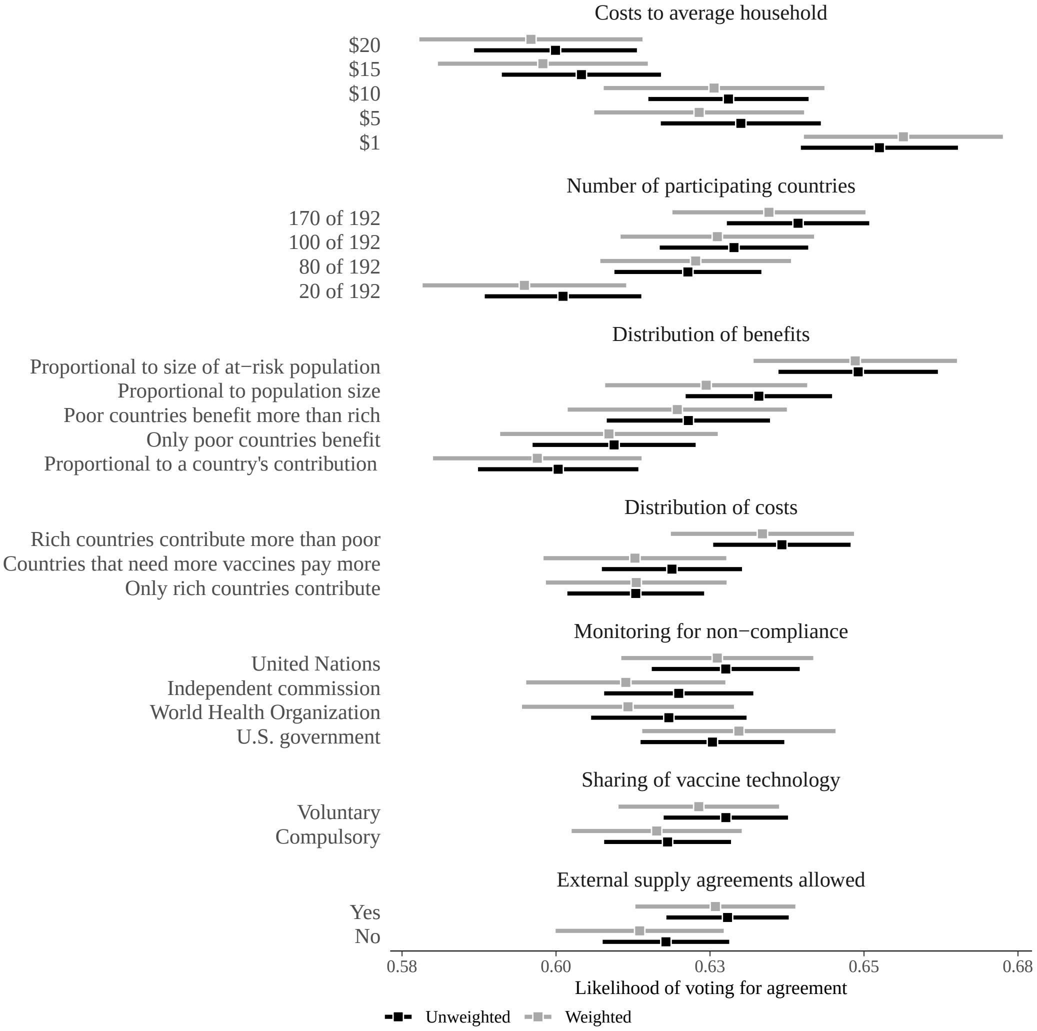

Notes: Marginal means showing public support for international cooperation on COVID-19 vaccine distribution across each randomly assigned institutional design feature. Outcome measure: 5-point scale standardized to $[0, 1]$ interval (0 = "Definitely vote against",...,0.5 = "Neither in favor nor against", ...,1 = "Definitely vote in favor"). Point estimates and 95% confidence intervals estimated via OLS regression with robust standard errors clustered at respondent level to correct for within-respondent clustering. NORC/Amerispeak survey of U.S. adults fielded in September/October 2021 (N = 4,214 respondents x 2 pairings x 2 agreements per pair = 16,856 observations).

Figure S20: Estimated AMCEs on likelihood of voting for international agreement in NORC conjoint, with and without survey weights

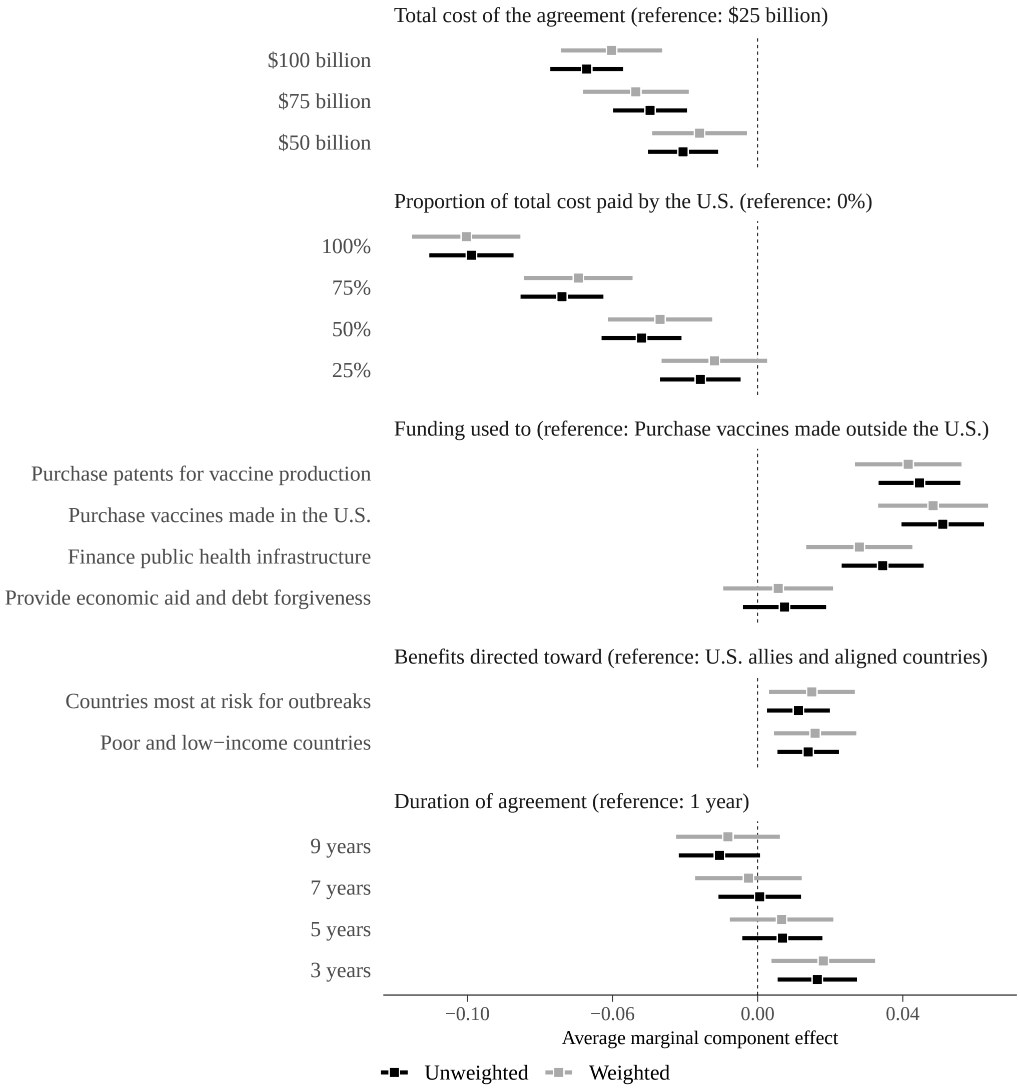

Notes: Effects of institutional design features on public support for international cooperation on COVID-19 vaccine distribution. Outcome measure: 5-point scale standardized to $[0, 1]$ interval (0 = "Definitely vote against",...,0.5 = "Neither in favor nor against", ...,1 = "Definitely vote in favor"). Point estimates and 95% confidence intervals estimated via OLS regression with robust standard errors clustered at respondent level to correct for within-respondent clustering. NORC/Amerispeak survey of U.S. adults fielded in September/October 2021 (N = 4,214 respondents x 2 pairings x 2 agreements per pair = 16,856 observations).

S3.5 Effect heterogeneity in vaccine recipient conjoint by respondents' background characteristics

Figure S21: Estimated marginal means in vaccine recipient conjoint by vaccine nationalism indicator

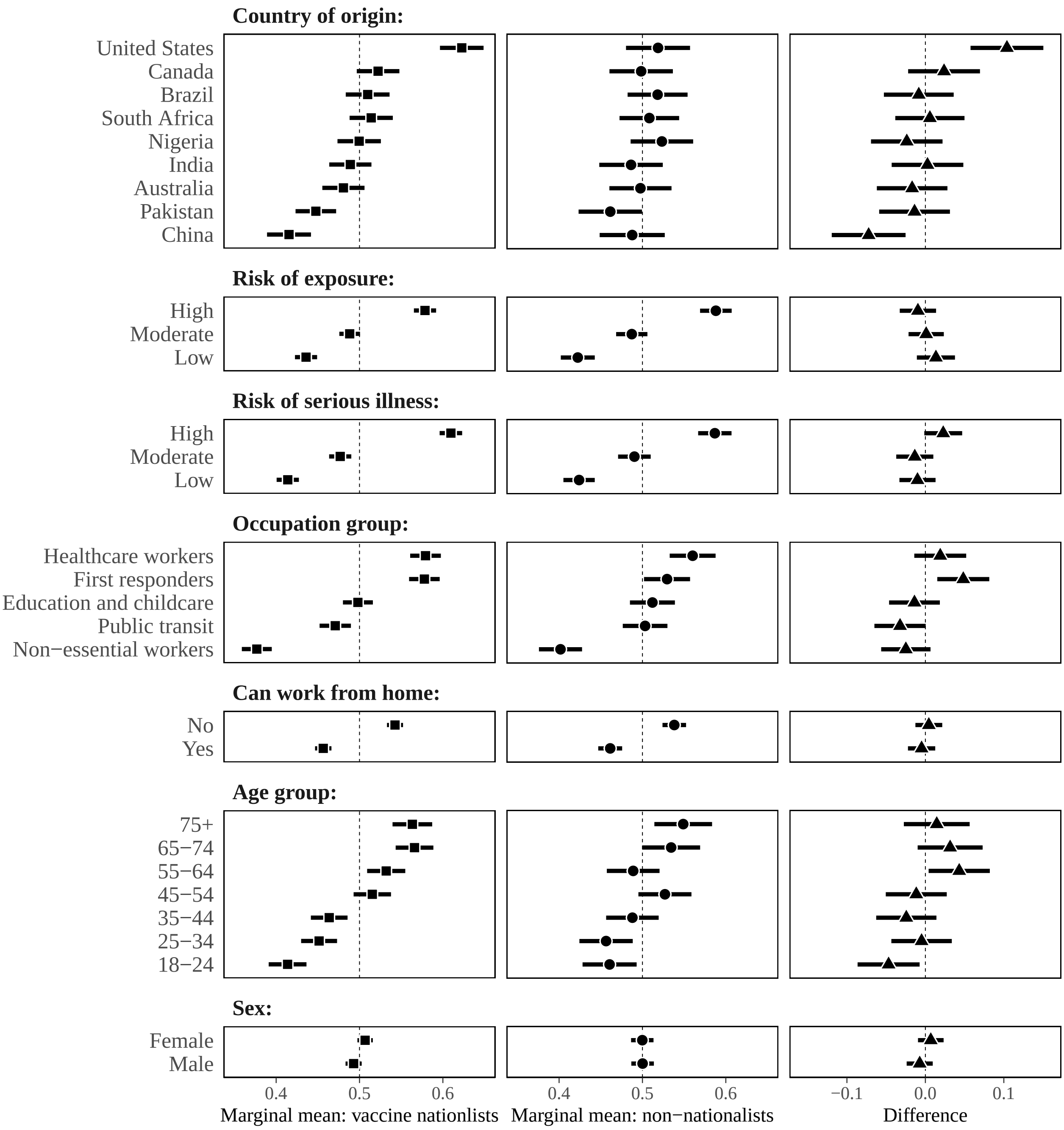

Notes: Sub-group estimates showing marginal means among vaccine nationalists, non-nationalists, and the differences. Sub-group classifications are based on the binary vaccine nationalism indicator. Point estimates and 95% confidence intervals estimated via OLS regression with robust standard errors clustered at respondent level to correct for within-respondent clustering. Lucid survey of U.S. adults fielded in April 2021 (N = 1,751 respondents x 5 pairings x 2 agreements per pair = 17,510 observations).

Figure S22: Estimated AMCEs in vaccine recipient conjoint by vaccine nationalism indicator

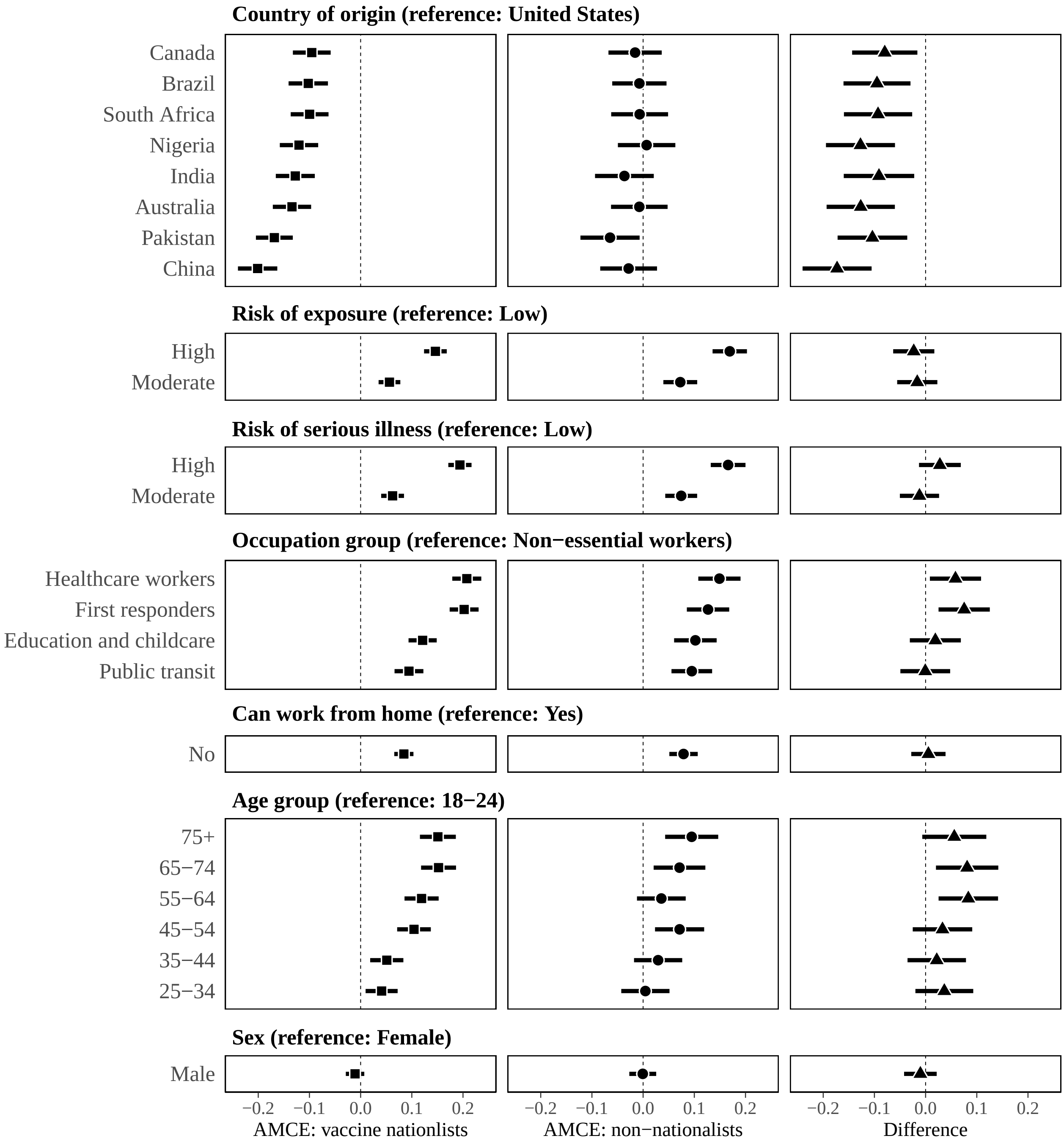

Notes: Sub-group estimates showing average marginal component effects (AMCEs) among vaccine nationalists, non-nationalists, and the differences. Sub-group classifications are based on the binary vaccine nationalism indicator. Point estimates and 95% confidence intervals estimated via OLS regression with robust standard errors clustered at respondent level to correct for within-respondent clustering. Lucid survey of U.S. adults fielded in April 2021 (N = 1,751 respondents x 5 pairings x 2 agreements per pair = 17,510 observations).

Figure S23: Estimated marginal means in vaccine recipient conjoint by vaccine nationalism index

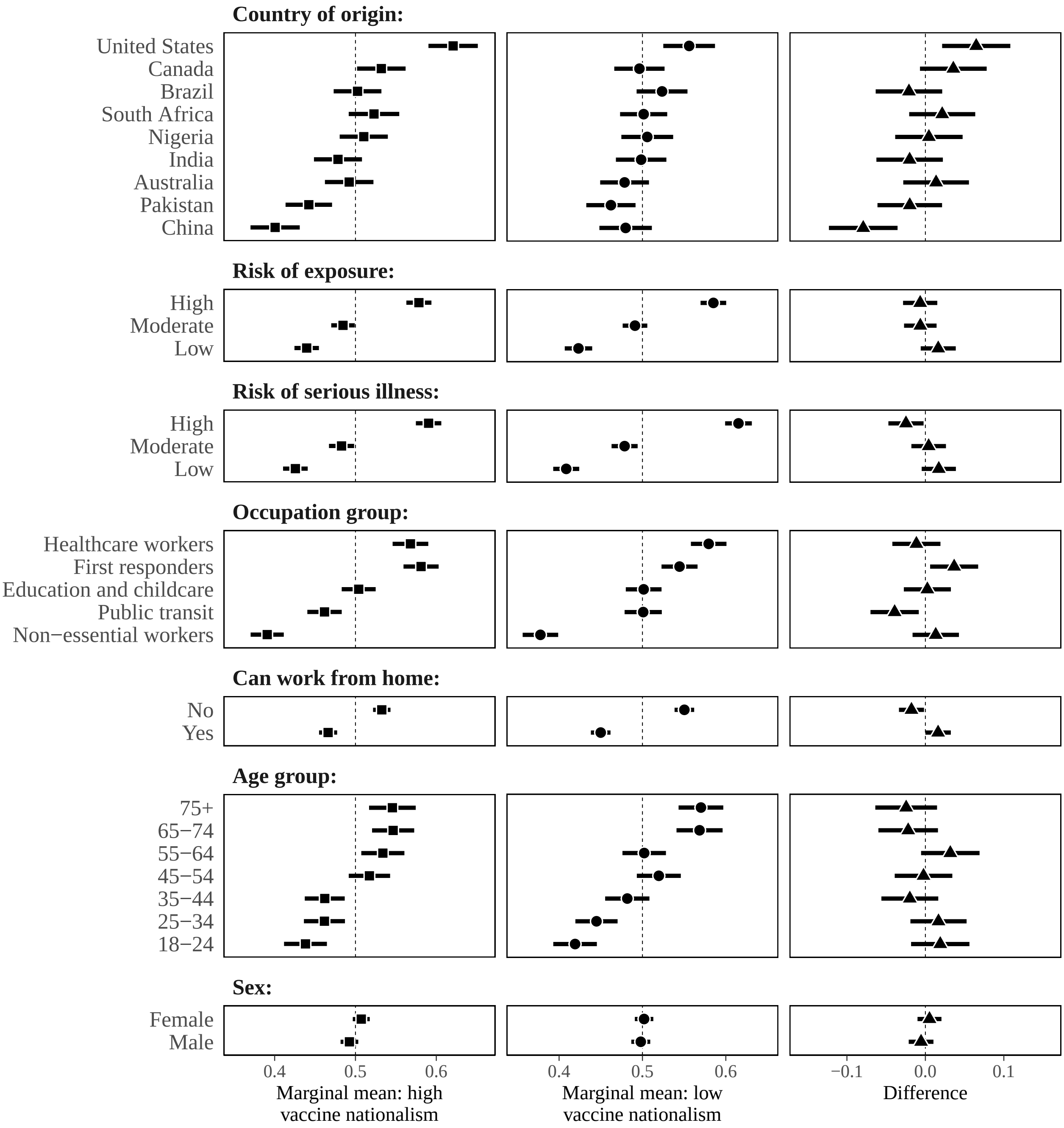

Notes: Sub-group estimates showing marginal means among vaccine nationalists, non-nationalists, and the differences. Sub-group classifications are based on scores derived from the 5-item vaccine nationalism index; vaccine nationalism is coded as high if a respondent scored higher than the median respondent. Point estimates and 95% confidence intervals estimated via OLS regression with robust standard errors clustered at respondent level to correct for within-respondent clustering. Lucid survey of U.S. adults fielded in April 2021 (N = 1,751 respondents x 5 pairings x 2 agreements per pair = 17,510 observations).

Figure S24: Estimated AMCEs in vaccine recipient conjoint by vaccine nationalism index

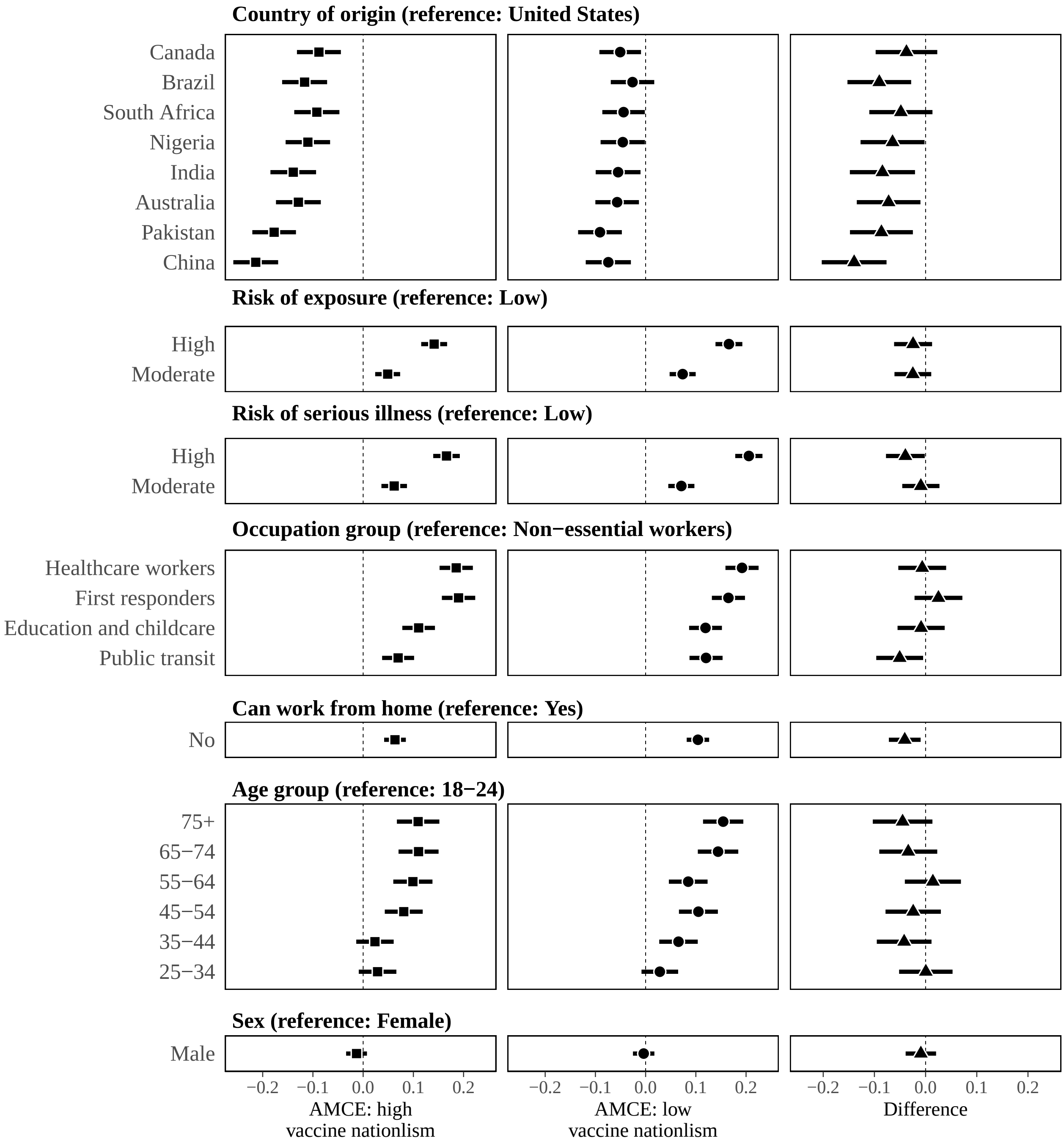

Notes: Sub-group estimates showing average marginal component effects (AMCEs) among vaccine nationalists, non-nationalists, and the differences. Sub-group classifications are based on scores derived from the 5-item vaccine nationalism index; vaccine nationalism is coded as high if a respondent scored higher than the median respondent. Point estimates and 95% confidence intervals estimated via OLS regression with robust standard errors clustered at respondent level to correct for within-respondent clustering. Lucid survey of U.S. adults fielded in April 2021 (N = 1,751 respondents x 5 pairings x 2 agreements per pair = 17,510 observations).

Figure S25: Estimated marginal means in vaccine recipient conjoint by nationalism

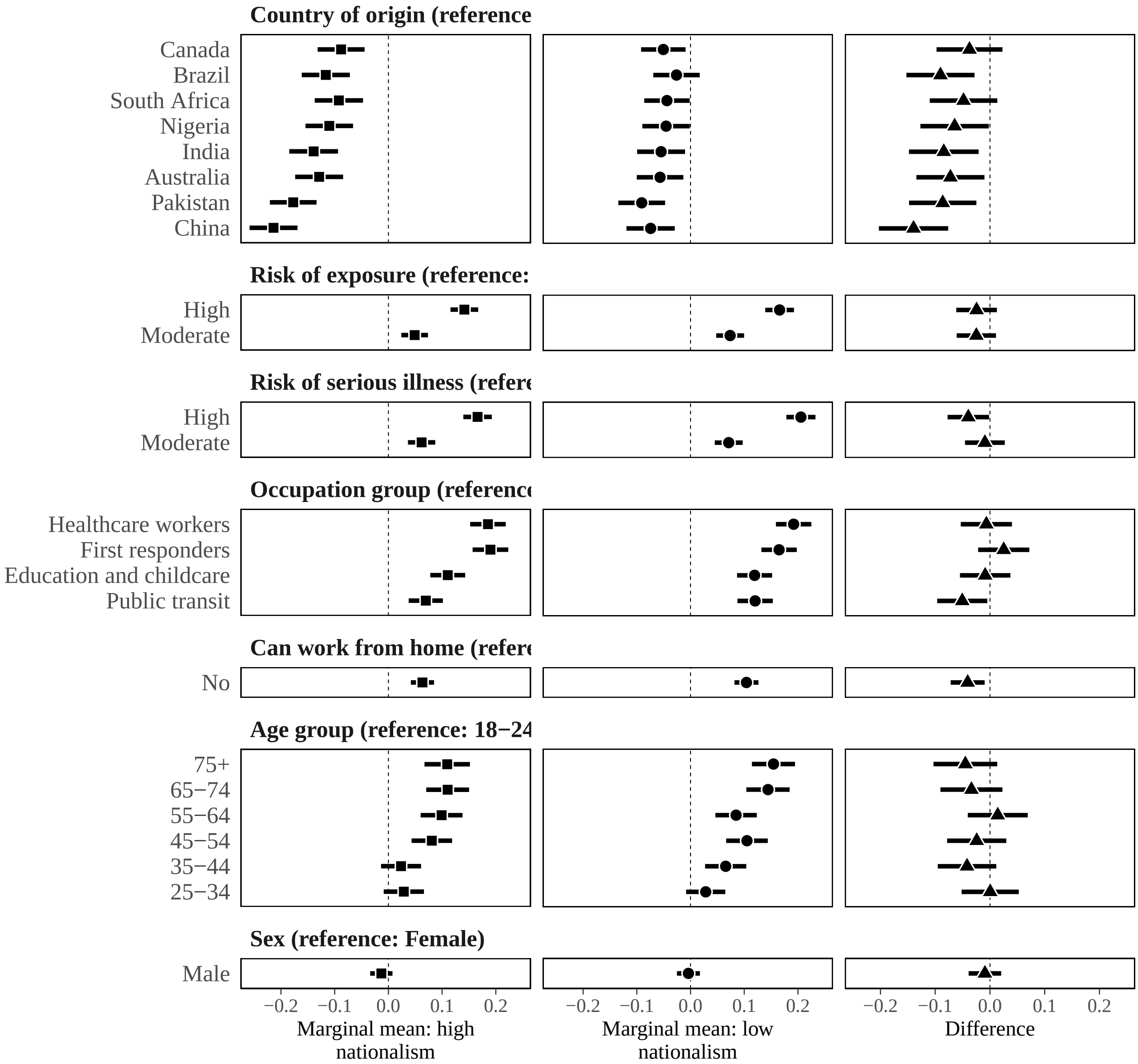

Notes: Sub-group estimates showing marginal means among nnationalists, non-nationalists, and the differences. Sub-group classifications are based on scores derived from a 7-point nationalism scale; nationalism is coded as high if a respondent scored higher than the median respondent. Point estimates and 95% confidence intervals estimated via OLS regression with robust standard errors clustered at respondent level to correct for within-respondent clustering. Lucid survey of U.S. adults fielded in April 2021 (N = 1,751 respondents x 5 pairings x 2 agreements per pair = 17,510 observations).

Figure S26: Estimated AMCEs in vaccine recipient conjoint by nationalism

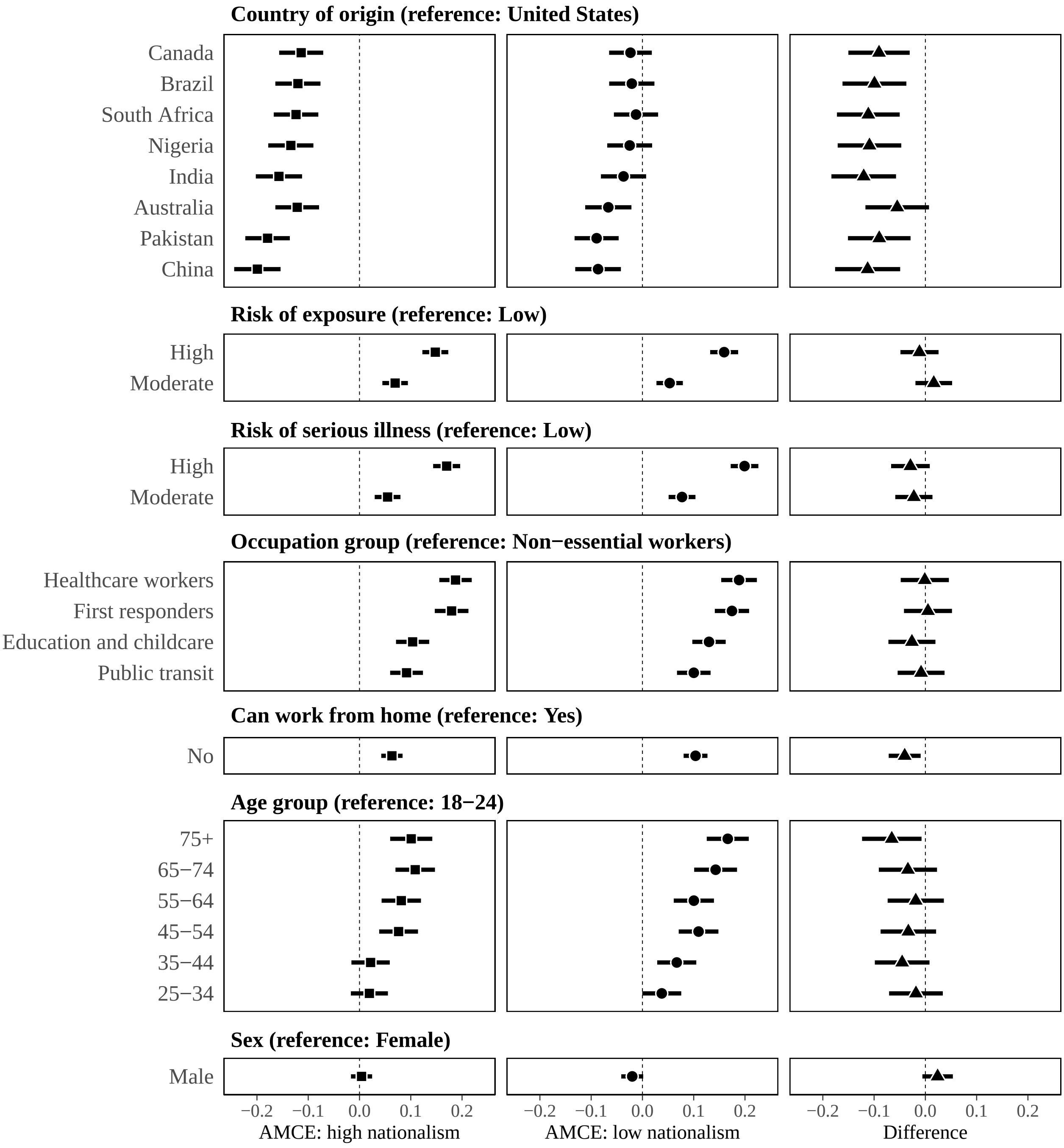

Notes: Sub-group estimates showing average marginal component effects (AMCEs) among nationalists, non-nationalists, and the differences. Sub-group classifications are based on scores derived from a 7-point nationalism scale; nationalism is coded as high if a respondent scored higher than the median respondent.Point estimates and 95% confidence intervals estimated via OLS regression with robust standard errors clustered at respondent level to correct for within-respondent clustering. Lucid survey of U.S. adults fielded in April 2021 (N = 1,751 respondents x 5 pairings x 2 agreements per pair = 17,510 observations).

Figure S27: Estimated marginal means in vaccine recipient conjoint by cosmopolitanism

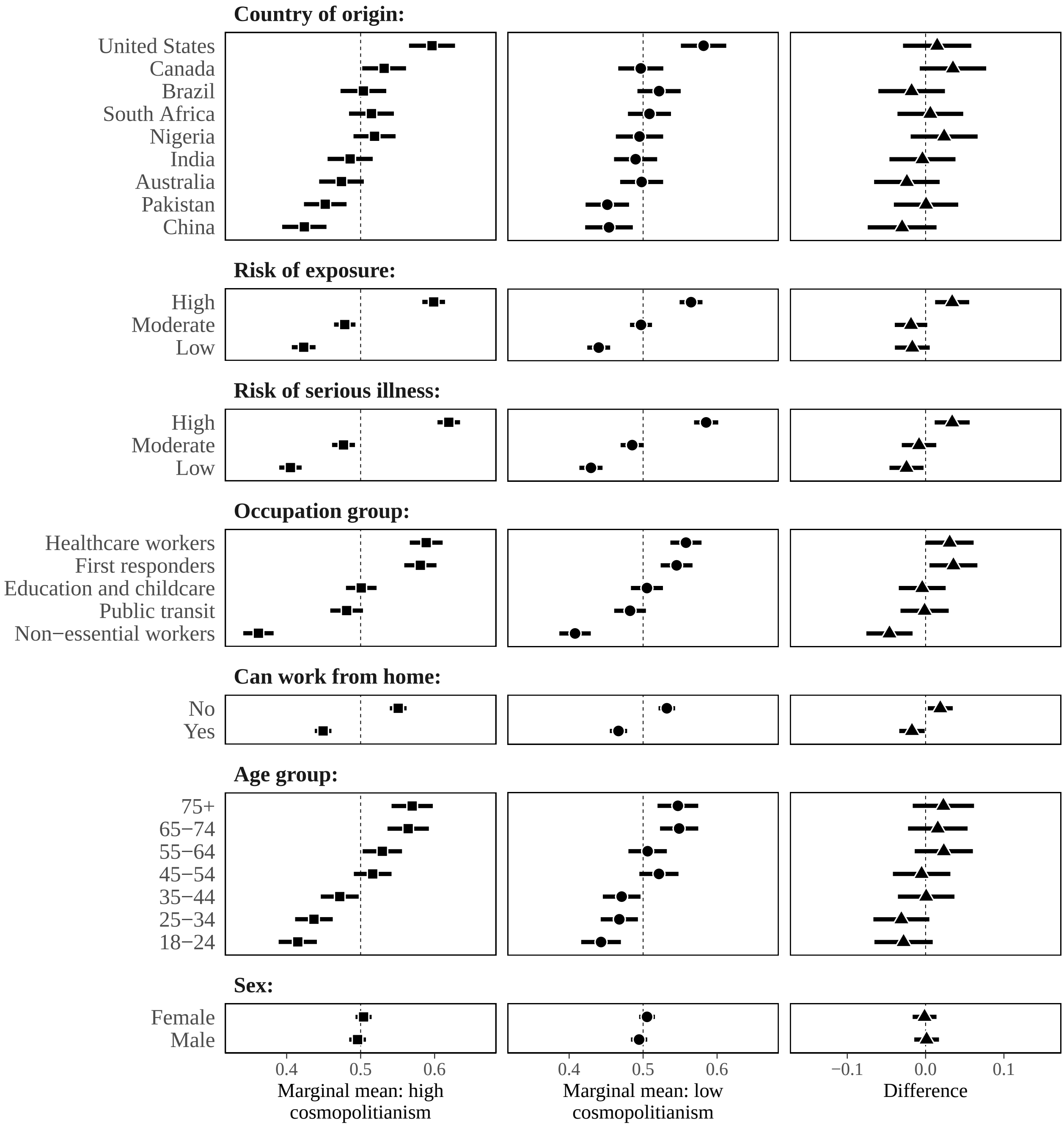

Notes: Sub-group estimates showing marginal means among those high in cosmopolitanism, low in cosmopolitanism, and the differences. Sub-group classifications are based on scores derived from a 7-point cosmopolitanism scale; cosmopolitanism is coded as high if a respondent scored higher than the median respondent. Point estimates and 95% confidence intervals estimated via OLS regression with robust standard errors clustered at respondent level to correct for within-respondent clustering. Lucid survey of U.S. adults fielded in April 2021 (N = 1,751 respondents x 5 pairings x 2 agreements per pair = 17,510 observations).

Figure S28: Estimated AMCEs in vaccine recipient conjoint by cosmopolitanism

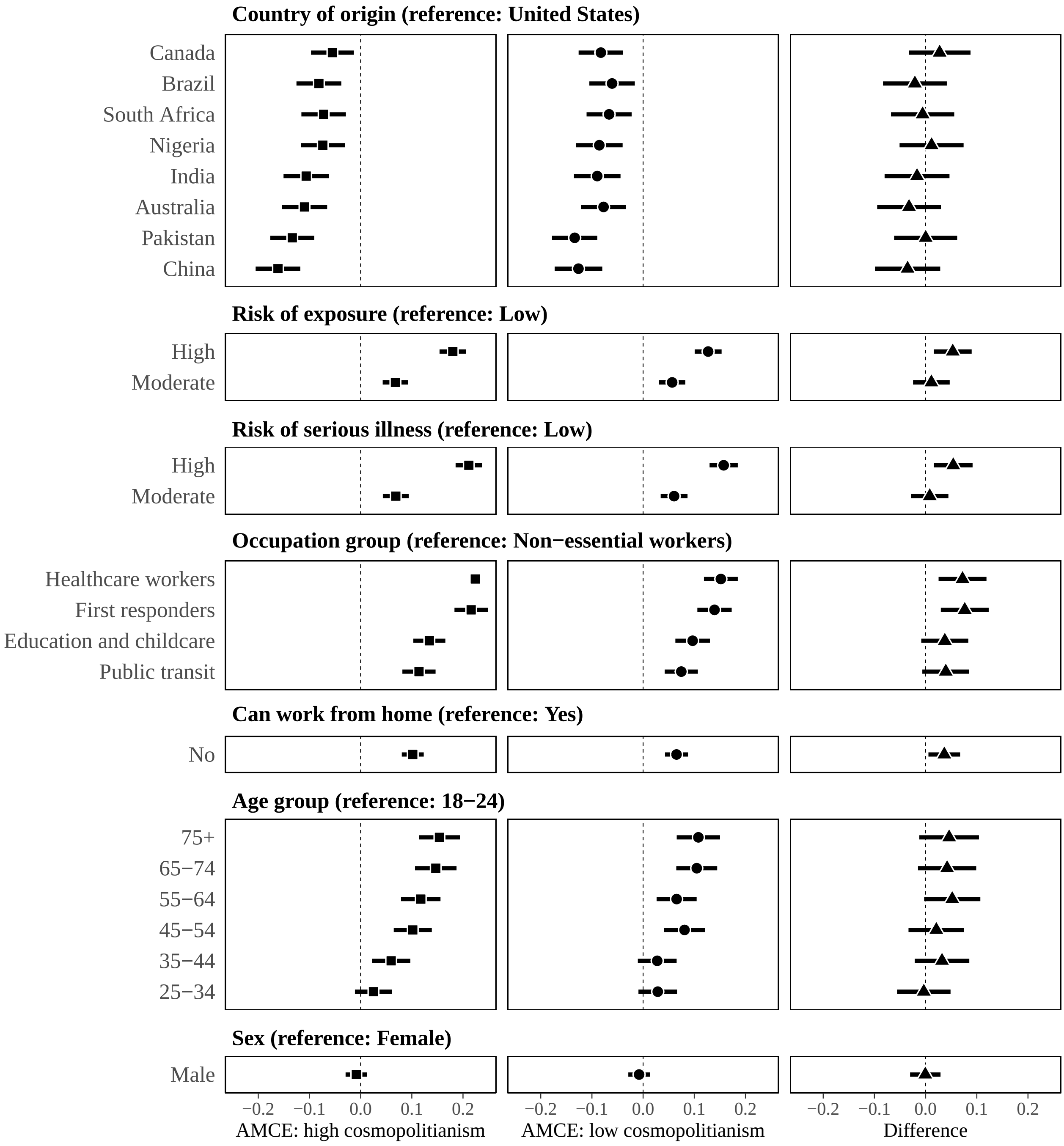

Notes: Sub-group estimates showing average marginal component effects (AMCEs) among those high in cosmopolitanism, low in cosmopolitanism and the differences. Sub-group classifications are based on scores derived from a 7-point cosmopolitanism scale; cosmopolitanism is coded as high if a respondent scored higher than the median respondent. Point estimates and 95% confidence intervals estimated via OLS regression with robust standard errors clustered at respondent level to correct for within-respondent clustering. Lucid survey of U.S. adults fielded in April 2021 (N = 1,751 respondents x 5 pairings x 2 agreements per pair = 17,510 observations).

Figure S29: Estimated marginal means in vaccine recipient conjoint by altruism

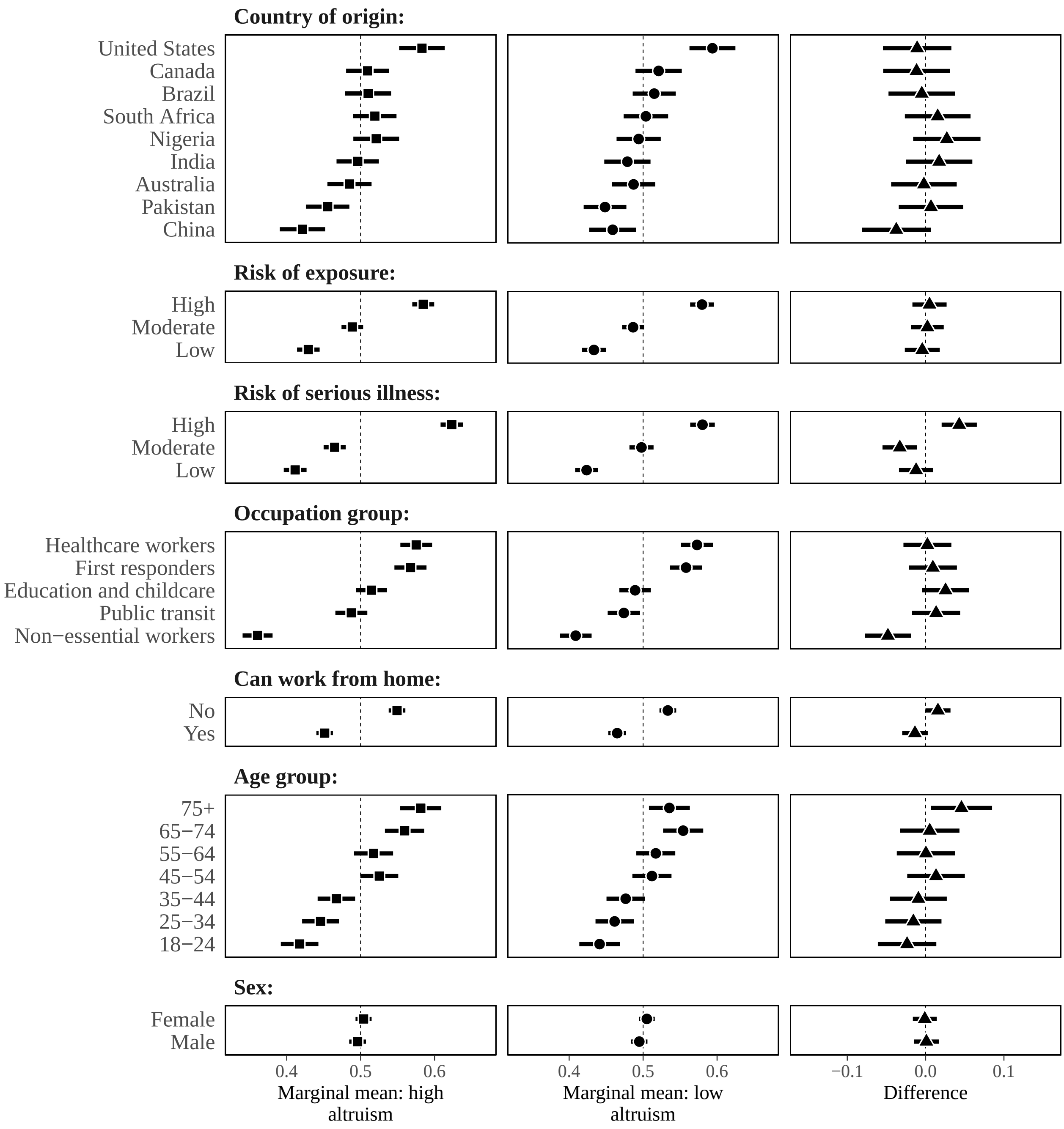

Notes: Sub-group estimates showing marginal means among those high in altruism, low in altruism, and the differences. Sub-group classifications are based on scores derived from a 2-item altruism index; altruism is coded as high if a respondent scored higher than the median respondent. Point estimates and 95% confidence intervals estimated via OLS regression with robust standard errors clustered at respondent level to correct for within-respondent clustering. Lucid survey of U.S. adults fielded in April 2021 (N = 1,751 respondents x 5 pairings x 2 agreements per pair = 17,510 observations).

Figure S30: Estimated AMCEs in vaccine recipient conjoint by altruism

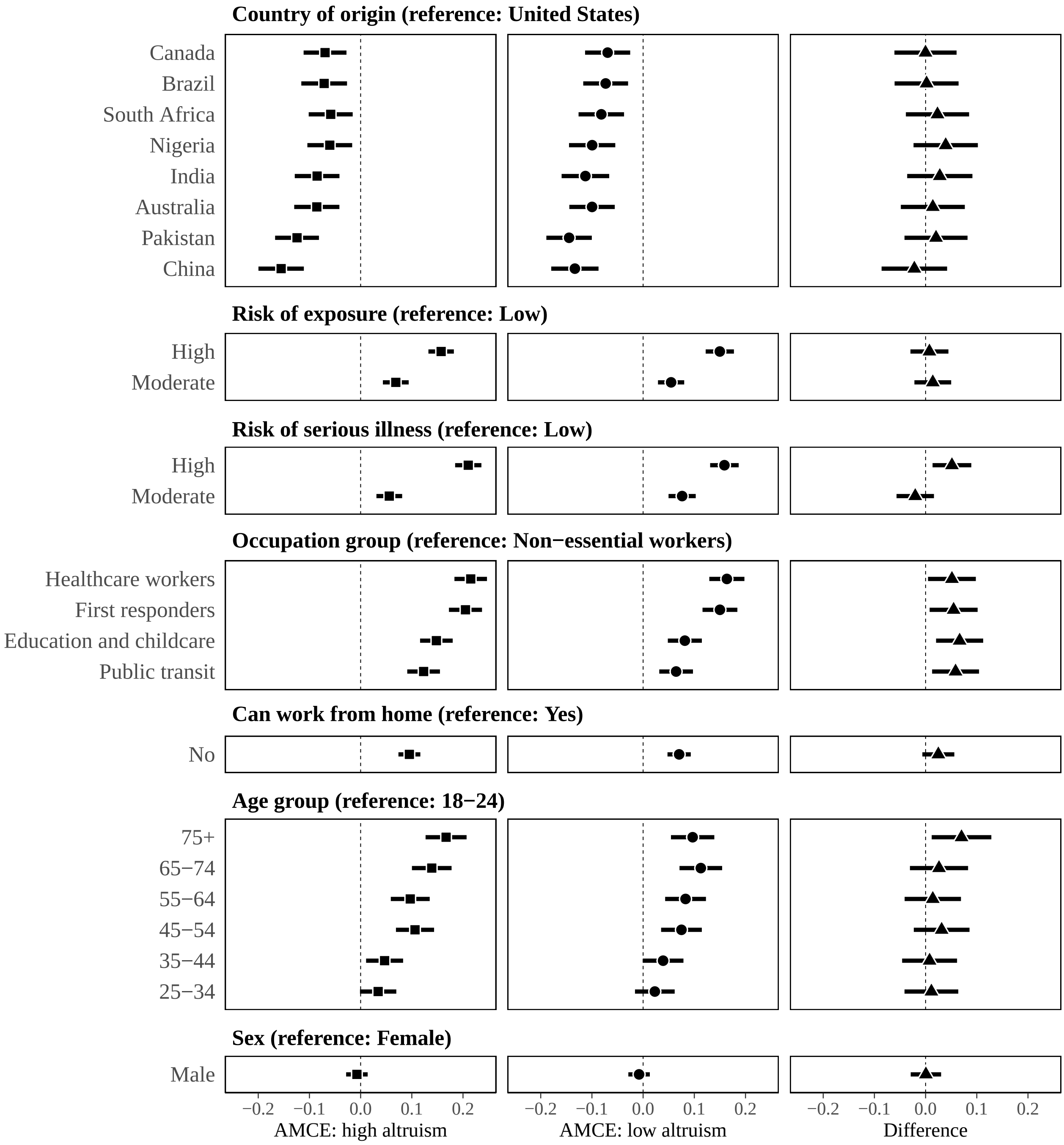

Notes: Sub-group estimates showing average marginal component effects (AMCEs) among those high in altruism, low in altruism, and the differences. Sub-group classifications are based on scores derived from a 2-item altruism index; altruism is coded as high if a respondent scored higher than the median respondent. Point estimates and 95% confidence intervals estimated via OLS regression with robust standard errors clustered at respondent level to correct for within-respondent clustering. Lucid survey of U.S. adults fielded in April 2021 (N = 1,751 respondents x 5 pairings x 2 agreements per pair = 17,510 observations).

Figure S31: Estimated marginal means in vaccine recipient conjoint by reciprocity

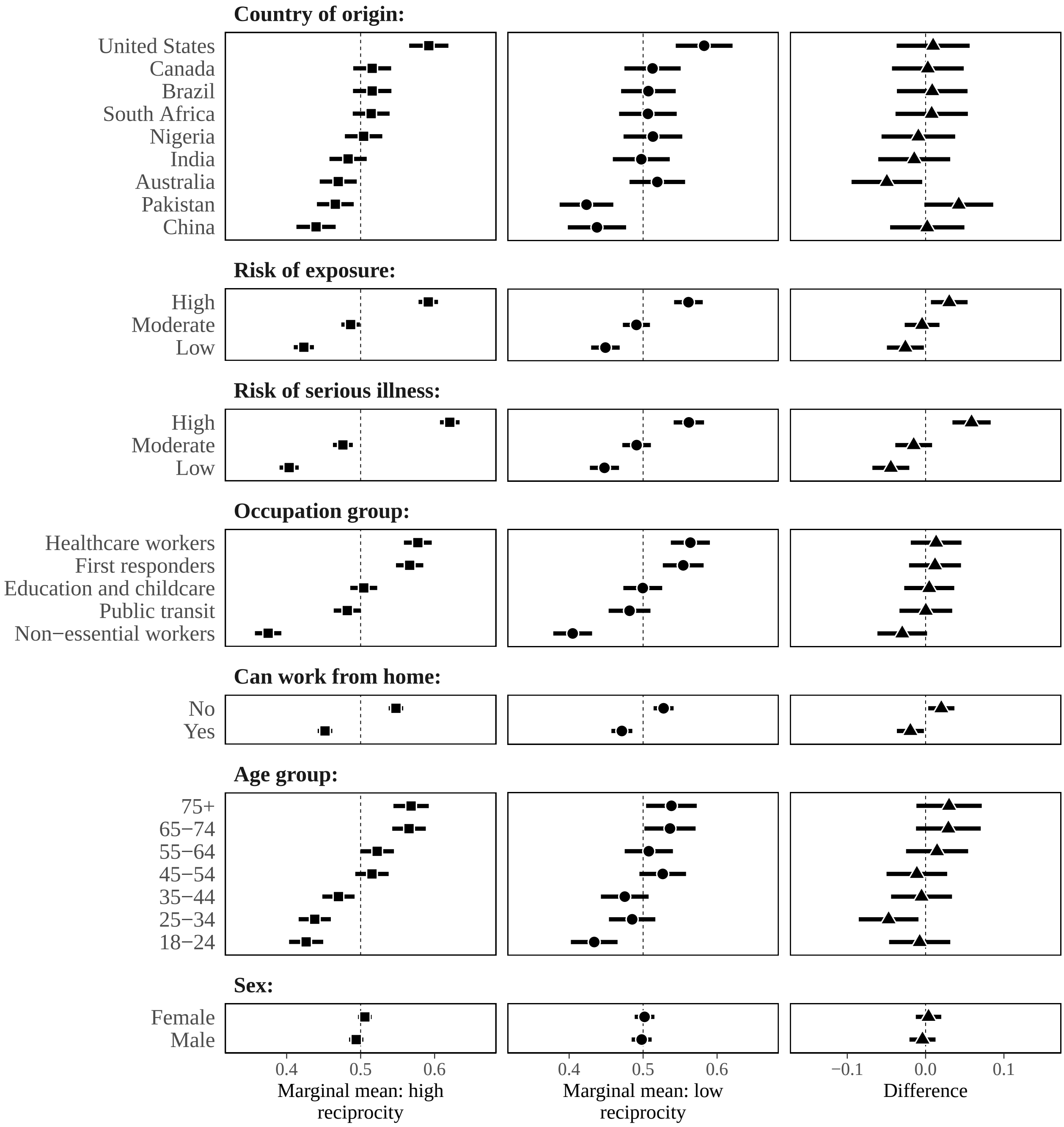

Notes: Sub-group estimates showing marginal means among those high in reciprocity, low in reciprocity, and the differences. Sub-group classifications are based on scores derived from the hypothetical gift-giving scenario; reciprocity is coded as high if a respondent scored higher than the median respondent. Point estimates and 95% confidence intervals estimated via OLS regression with robust standard errors clustered at respondent level to correct for within-respondent clustering. Lucid survey of U.S. adults fielded in April 2021 (N = 1,751 respondents x 5 pairings x 2 agreements per pair = 17,510 observations).

Figure S32: Estimated AMCEs in vaccine recipient conjoint by reciprocity

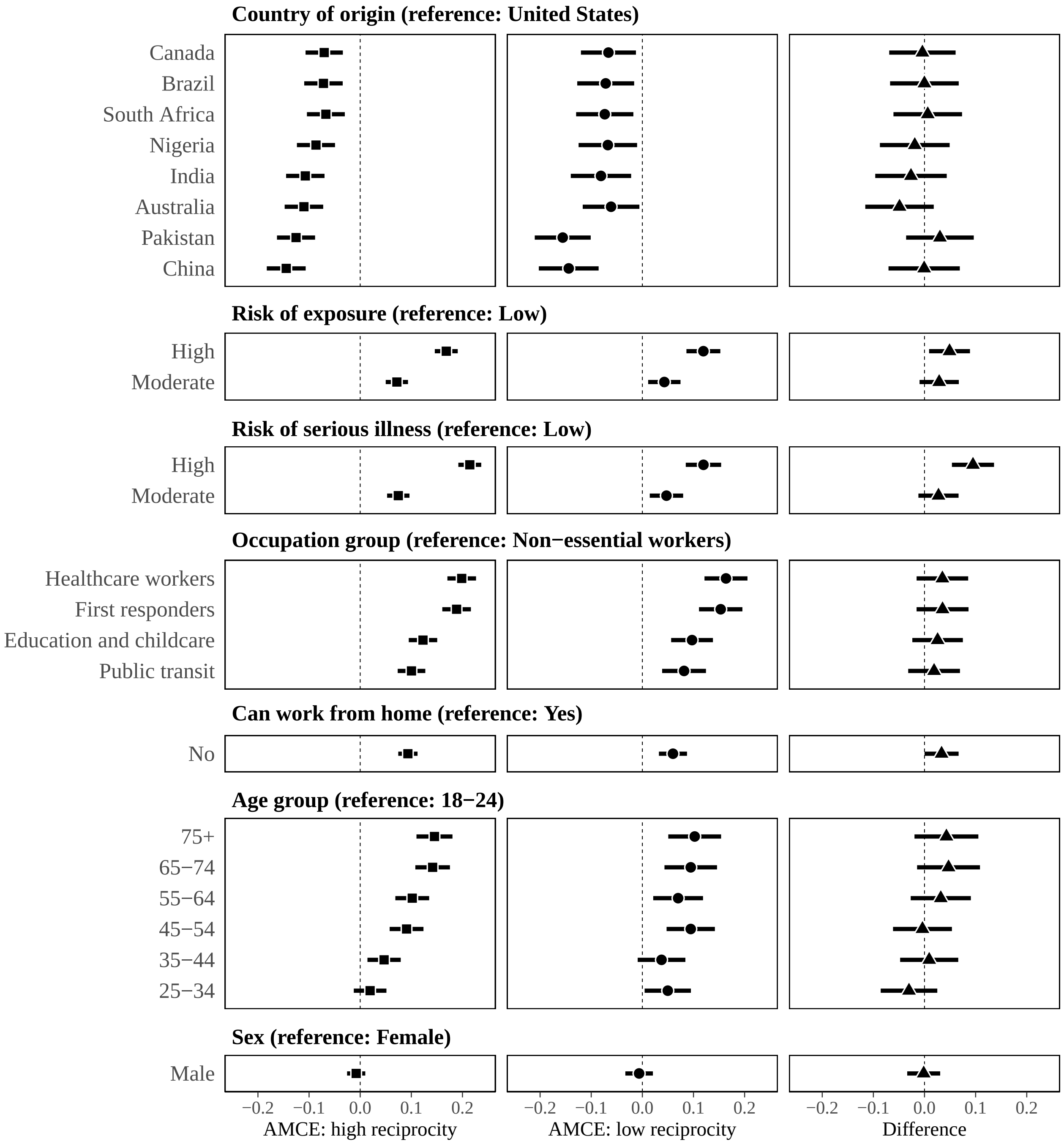

Notes: Sub-group estimates showing average marginal component effects (AMCEs) among those high in reciprocity, low in reciprocity, and the differences. Sub-group classifications are based on scores derived from the hypothetical gift-giving scenario; reciprocity is coded as high if a respondent scored higher than the median respondent. Point estimates and 95% confidence intervals estimated via OLS regression with robust standard errors clustered at respondent level to correct for within-respondent clustering. Lucid survey of U.S. adults fielded in April 2021 (N = 1,751 respondents x 5 pairings x 2 agreements per pair = 17,510 observations).

Figure S33: Estimated marginal means in vaccine recipient conjoint by vaccination status

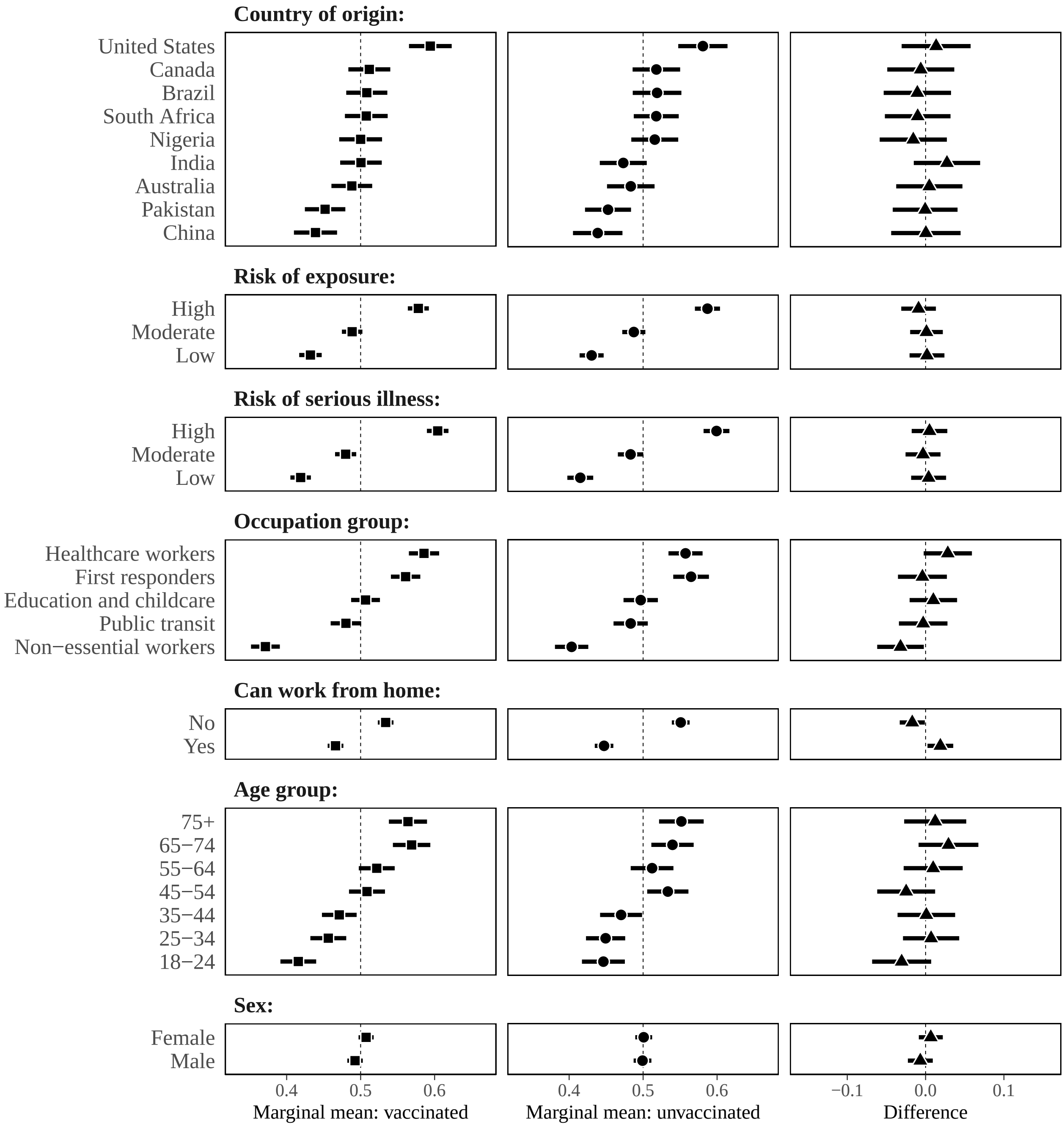

Notes: Sub-group estimates showing marginal means among those who had received a vaccination, had not received a vaccinated, and the differences. Individuals that reported receiving at least one dose of any COVID-19 vaccine at the time of the survey were classified as having received a vaccination. Point estimates and 95% confidence intervals estimated via OLS regression with robust standard errors clustered at respondent level to correct for within-respondent clustering. Lucid survey of U.S. adults fielded in April 2021 (N = 1,751 respondents x 5 pairings x 2 agreements per pair = 17,510 observations).

Figure S34: Estimated AMCEs in vaccine recipient conjoint by vaccination status

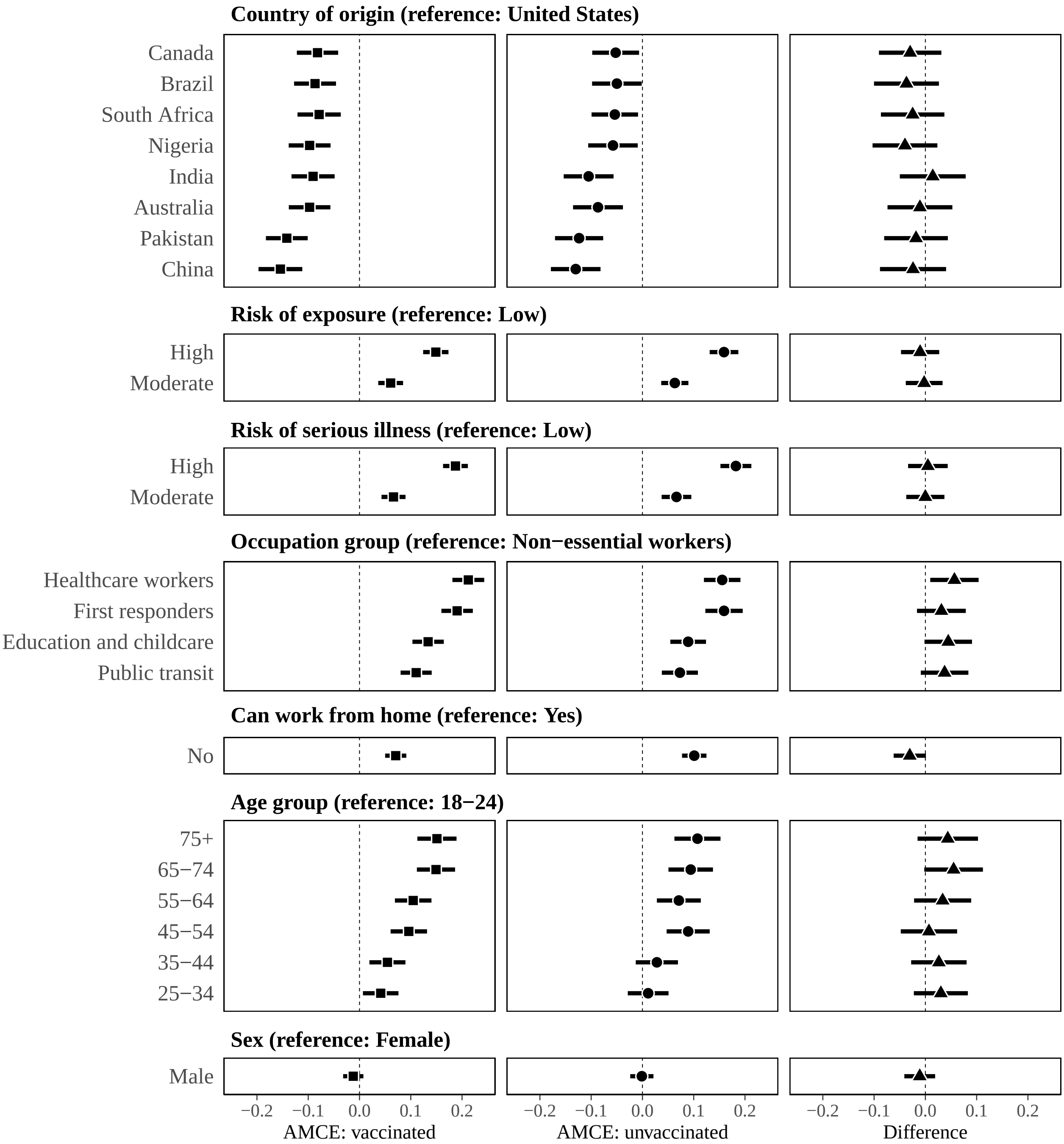

Notes: Sub-group estimates showing average marginal component effects (AMCEs) aamong those who had received a vaccination, had not received a vaccinated, and the differences. Individuals that reported receiving at least one dose of any COVID-19 vaccine at the time of the survey were classified as having received a vaccination. Point estimates and 95% confidence intervals estimated via OLS regression with robust standard errors clustered at respondent level to correct for within-respondent clustering. Lucid survey of U.S. adults fielded in April 2021 (N = 1,751 respondents x 5 pairings x 2 agreements per pair = 17,510 observations).

Figure S35: Estimated marginal means in vaccine recipient conjoint by partisanship

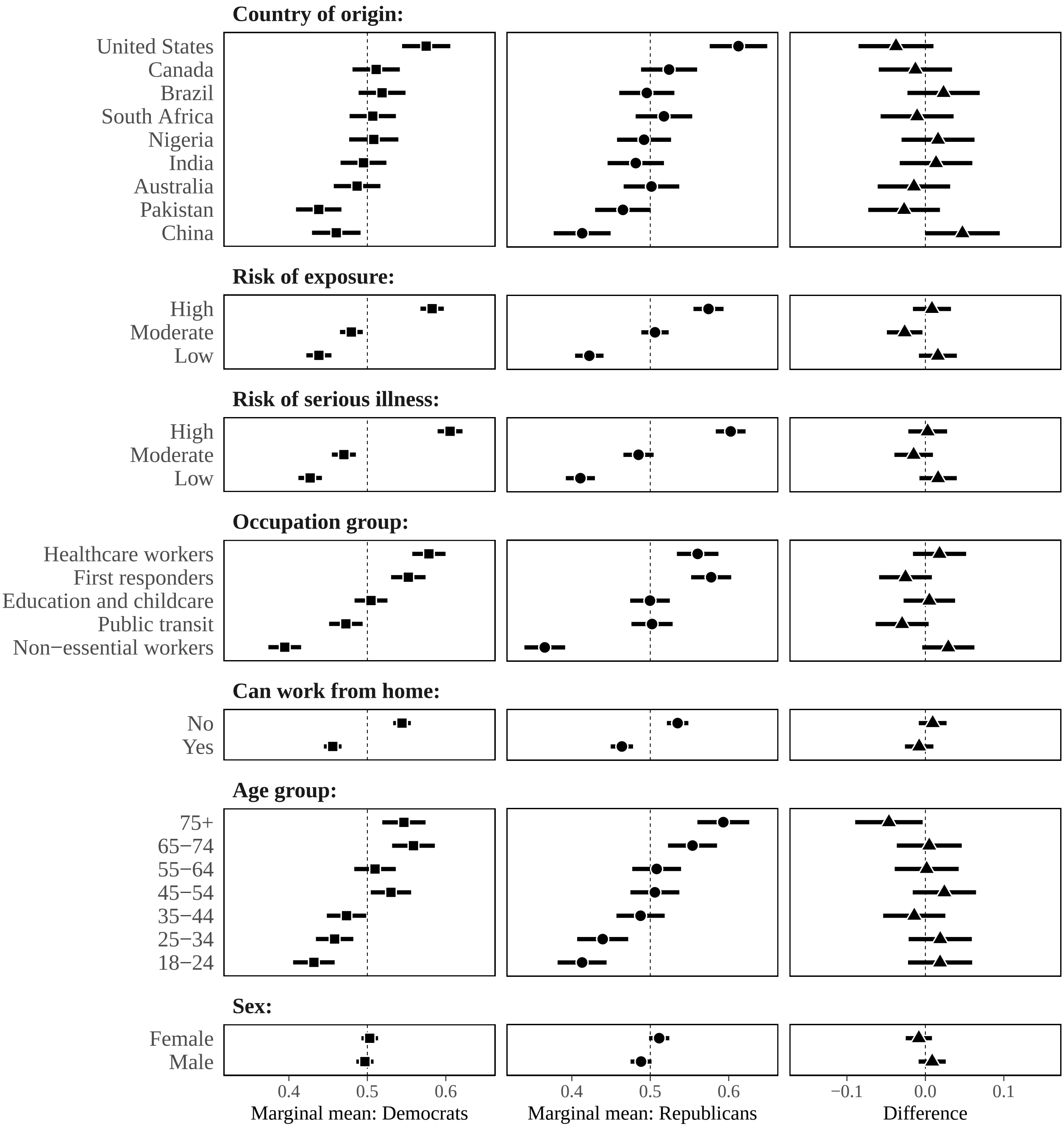

Notes: Sub-group estimates showing marginal means among Democrats, Republicans, and the differences. Partisanship is measured with the standard 7-point branching question used in the American National Election Studies (ANES) Survey. Therefore, Democrat and Republican sub-groups include weak supporters, strong supporters, and those that lean toward one party or another, whereas Independents do not lean one way or the other and are excluded. Point estimates and 95% confidence intervals estimated via OLS regression with robust standard errors clustered at respondent level to correct for within-respondent clustering. Lucid survey of U.S. adults fielded in April 2021 (N = 1,751 respondents x 5 pairings x 2 agreements per pair = 17,510 observations).

Figure S36: Estimated AMCEs in vaccine recipient conjoint by partisanship

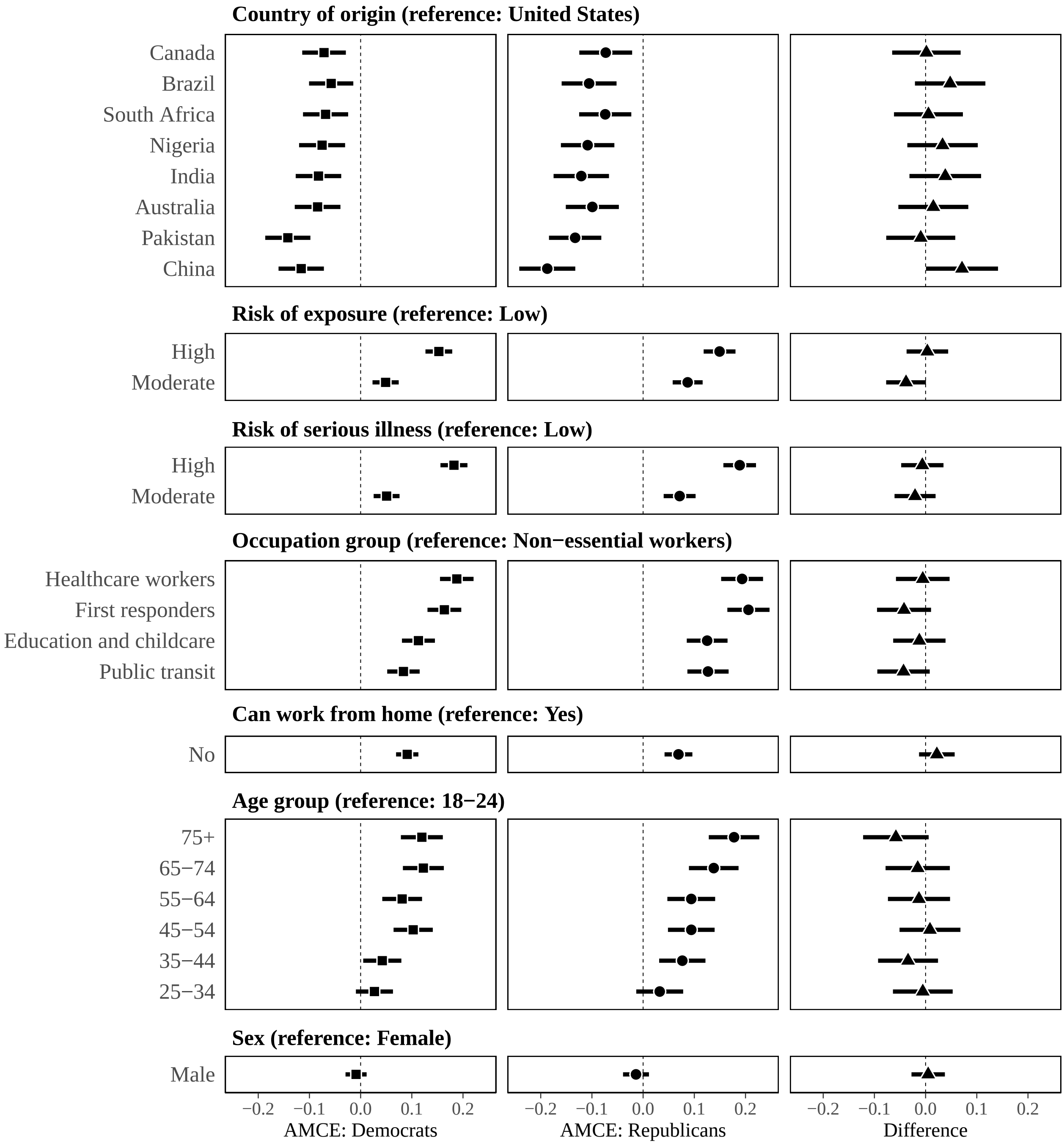

Notes: Sub-group estimates showing average marginal component effects (AMCEs) among Democrats, Republicans, and the differences. Partisanship is measured with the standard 7-point branching question used in the American National Election Studies (ANES) Survey. Therefore, Democrat and Republican sub-groups include weak supporters, strong supporters, and those that lean toward one party or another, whereas Independents do not lean one way or the other and are excluded. Point estimates and 95% confidence intervals estimated via OLS regression with robust standard errors clustered at respondent level to correct for within-respondent clustering. Lucid survey of U.S. adults fielded in April 2021 (N = 1,751 respondents x 5 pairings x 2 agreements per pair = 17,510 observations).

Figure S37: Estimated marginal means in vaccine recipient conjoint by political ideology

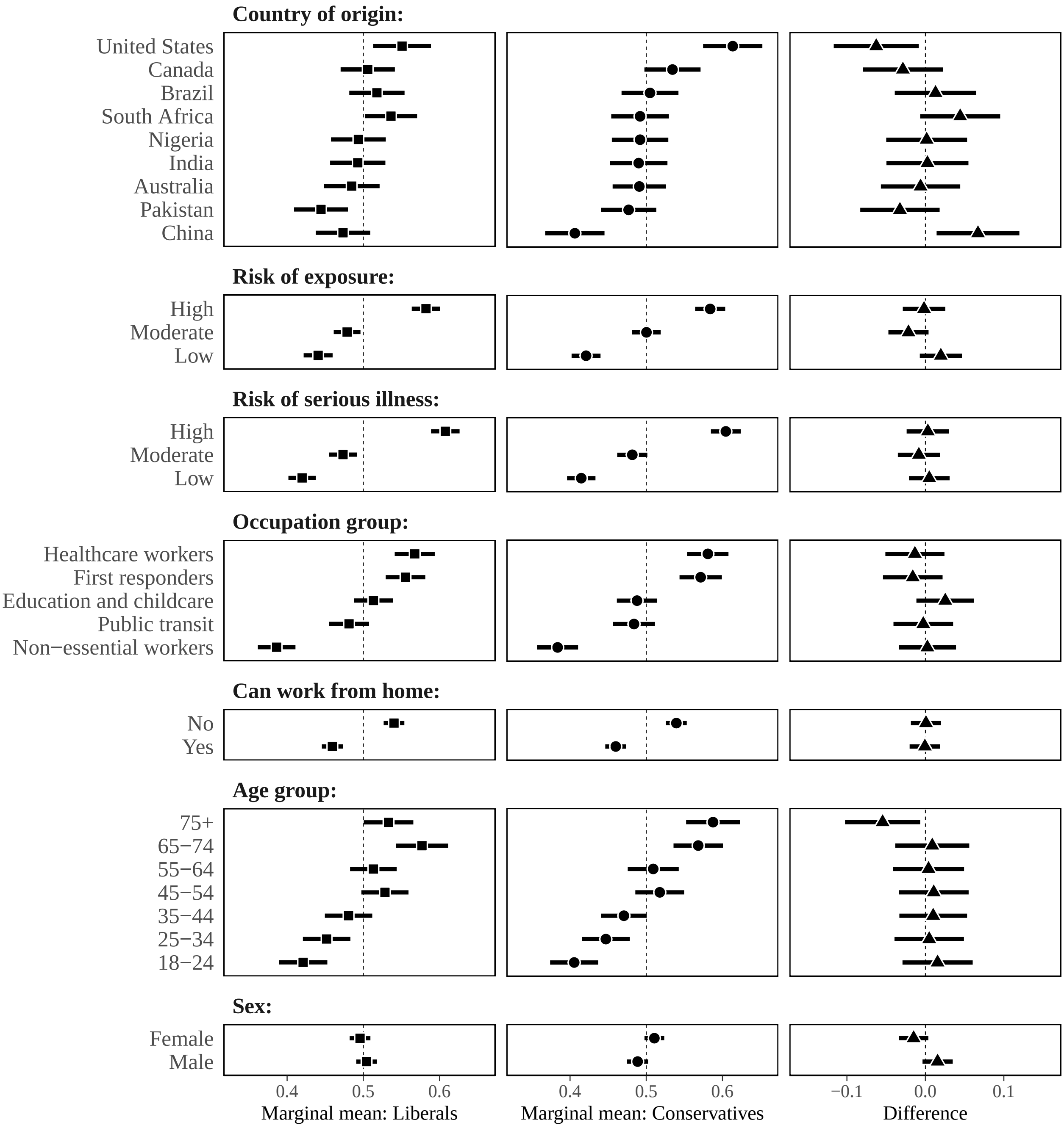

Notes: Sub-group estimates showing marginal means among Liberals, Conservatives, and the differences. Ideology is measured with the standard 7-point scale from "Extremely Liberal" to "Extremely Conservative", and moderates (those at the midpoint of the scale) are excluded. Point estimates and 95% confidence intervals estimated via OLS regression with robust standard errors clustered at respondent level to correct for within-respondent clustering. Lucid survey of U.S. adults fielded in April 2021 (N = 1,751 respondents x 5 pairings x 2 agreements per pair = 17,510 observations).

Figure S38: Estimated AMCEs in vaccine recipient conjoint by political ideology

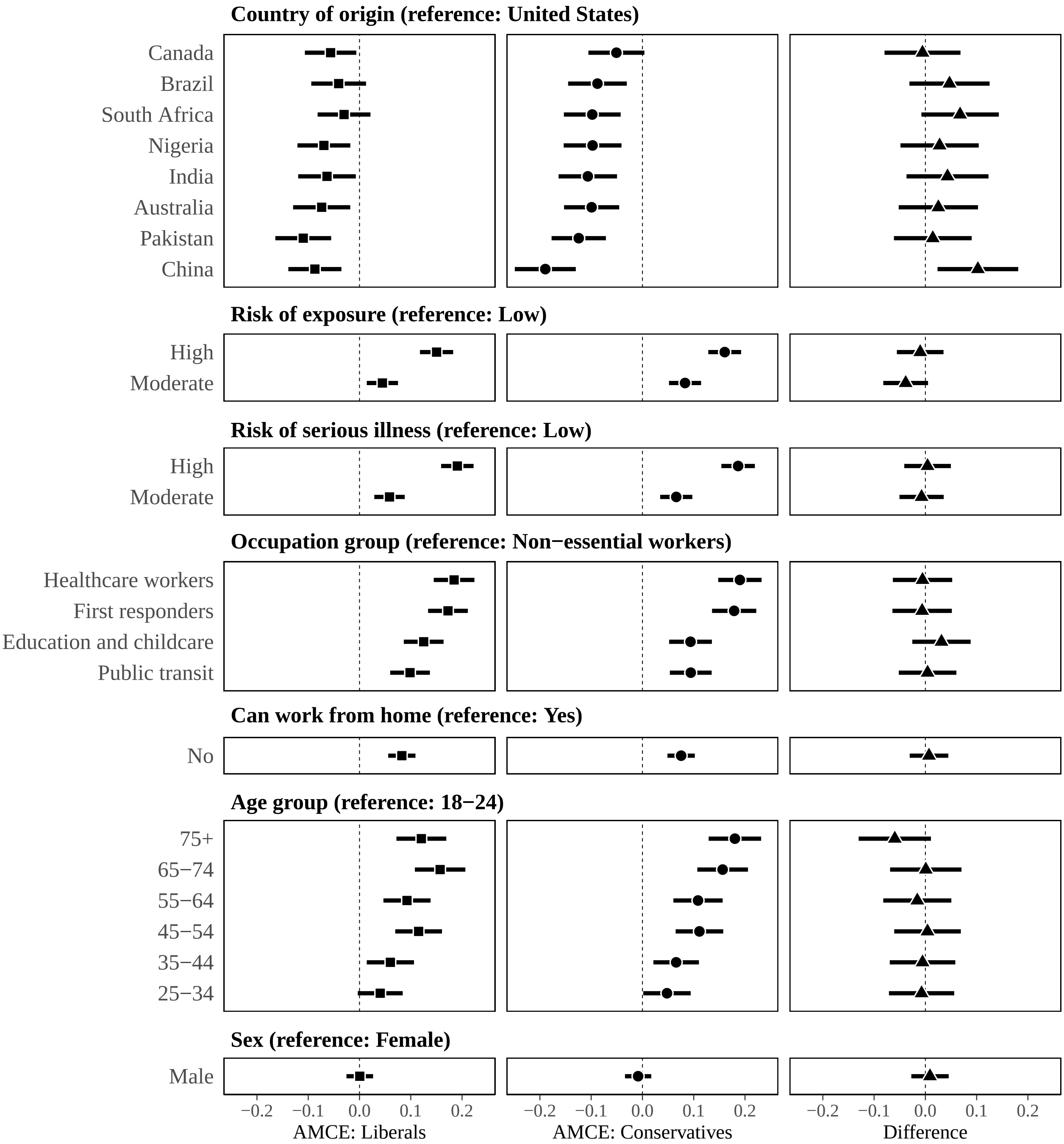

Notes: Sub-group estimates showing average marginal component effects (AMCEs) among Liberals, Conservatives, and the differences. Ideology is measured with the standard 7-point scale from "Extremely Liberal" to "Extremely Conservative", and moderates (those at the midpoint of the scale) are excluded. Point estimates and 95% confidence intervals estimated via OLS regression with robust standard errors clustered at respondent level to correct for within-respondent clustering. Lucid survey of U.S. adults fielded in April 2021 (N = 1,751 respondents x 5 pairings x 2 agreements per pair = 17,510 observations).

S3.6 Effect heterogeneity in international agreement conjoint (Lucid) by respondents' background characteristics

Figure S39: Estimated marginal means in international agreement conjoint (Lucid) by vaccine nationalism indicator

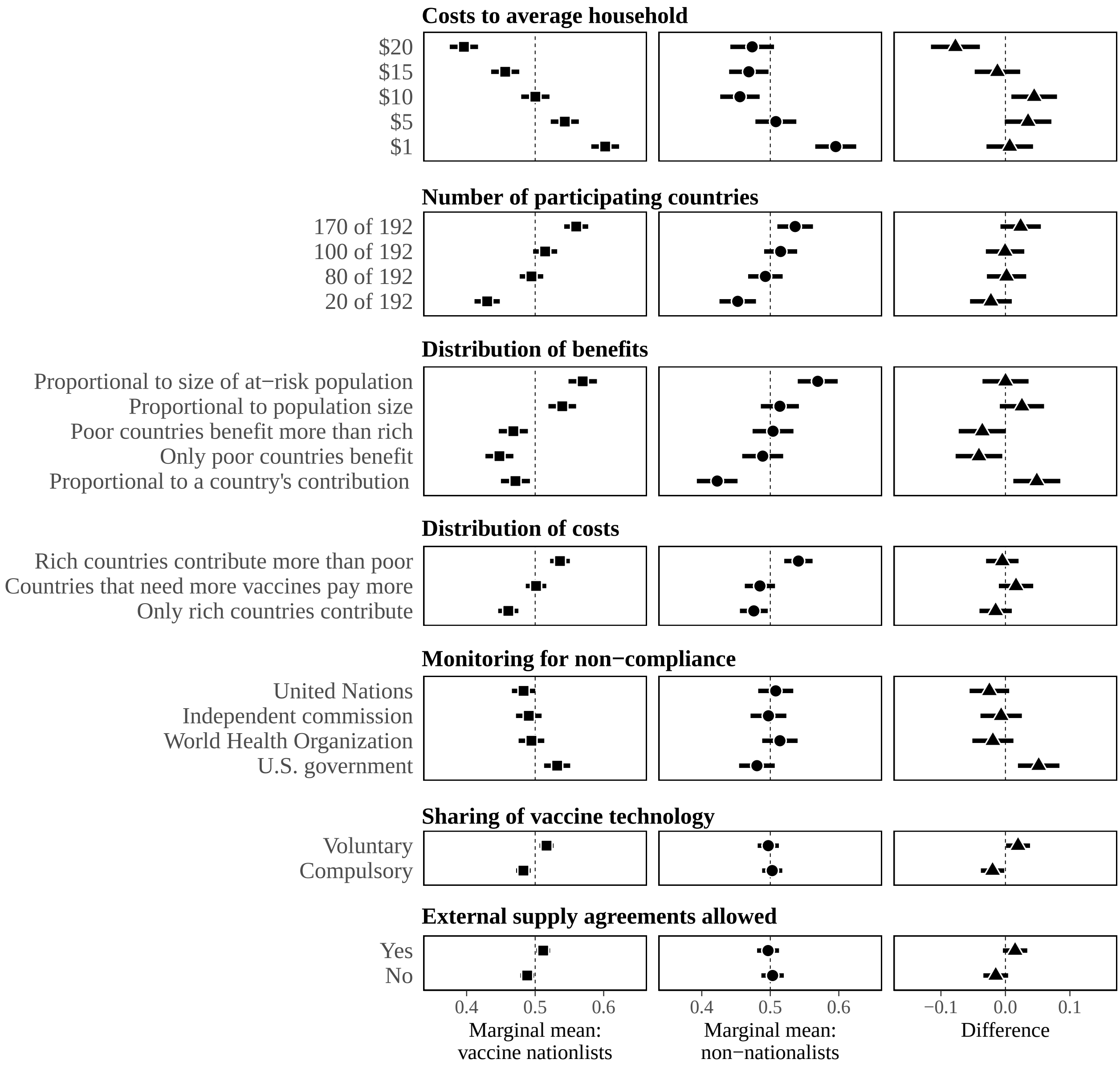

Notes: Sub-group estimates showing marginal means among vaccine nationalists, non-nationalists, and the differences. Sub-group classifications are based on the binary vaccine nationalism indicator. Point estimates and 95% confidence intervals estimated via OLS regression with robust standard errors clustered at respondent level to correct for within-respondent clustering. Lucid survey of U.S. adults fielded in April 2021 (N = 1,751 respondents x 4 pairings x 2 agreements per pair = 14,008 observations).

Figure S40: Estimated AMCEs in international agreement conjoint (Lucid) by vaccine nationalism indicator

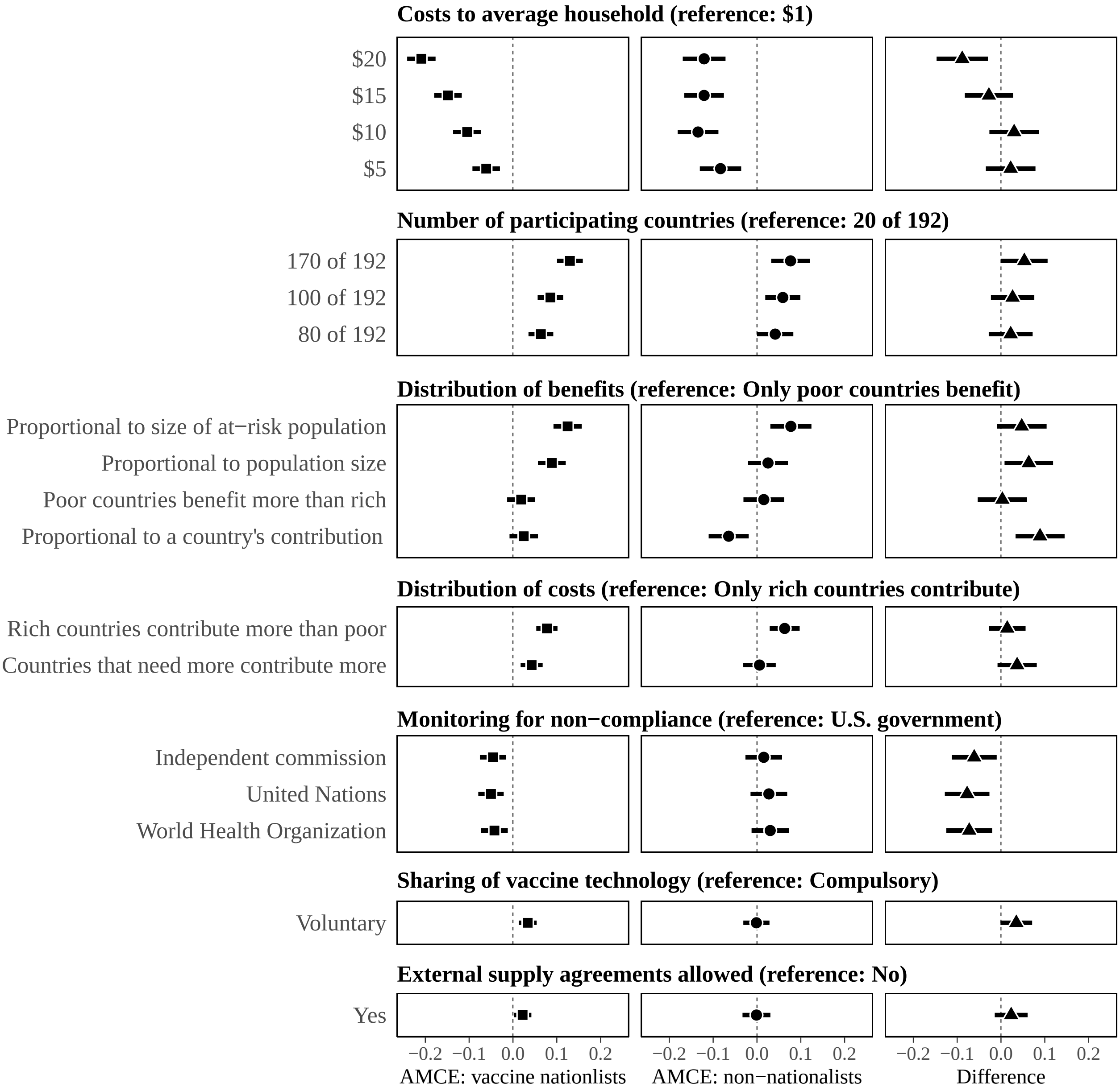

Notes: Sub-group estimates showing average marginal component effects (AMCEs) among vaccine nationalists, non-nationalists, and the differences. Sub-group classifications are based on the binary vaccine nationalism indicator. Point estimates and 95% confidence intervals estimated via OLS regression with robust standard errors clustered at respondent level to correct for within-respondent clustering. Lucid survey of U.S. adults fielded in April 2021 (N = 1,751 respondents x 4 pairings x 2 agreements per pair = 14,008 observations).

Figure S41: Estimated marginal means in international agreement conjoint (Lucid) by vaccine nationalism index

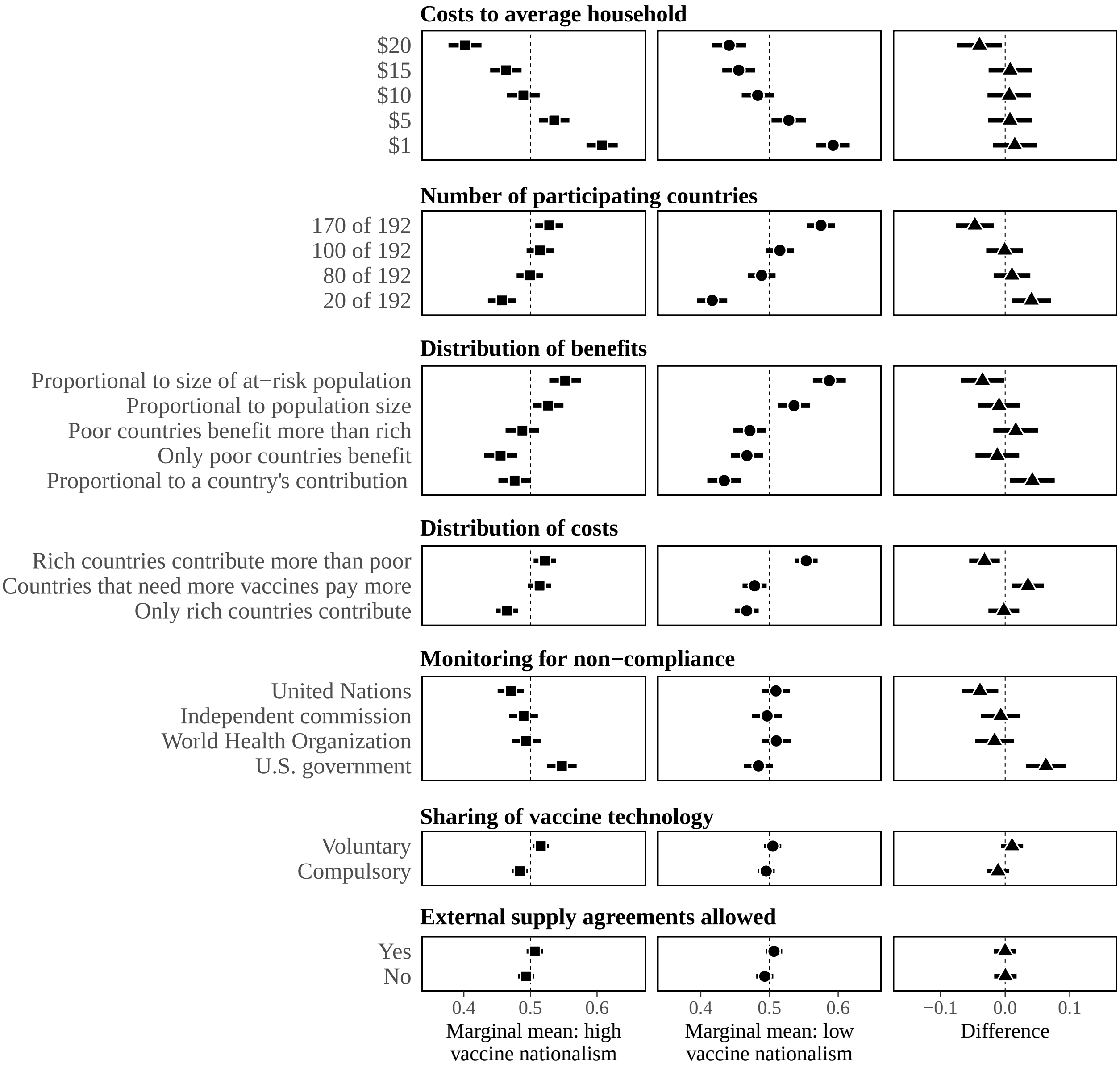

Notes: Sub-group estimates showing marginal means among vaccine nationalists, non-nationalists, and the differences. Sub-group classifications are based on scores derived from the 5-item vaccine nationalism index; vaccine nationalism is coded as high if a respondent scored higher than the median respondent. Point estimates and 95% confidence intervals estimated via OLS regression with robust standard errors clustered at respondent level to correct for within-respondent clustering. Lucid survey of U.S. adults fielded in April 2021 (N = 1,751 respondents x 4 pairings x 2 agreements per pair = 14,008 observations).

Figure S42: Estimated AMCEs in international agreement conjoint (Lucid) by vaccine nationalism index

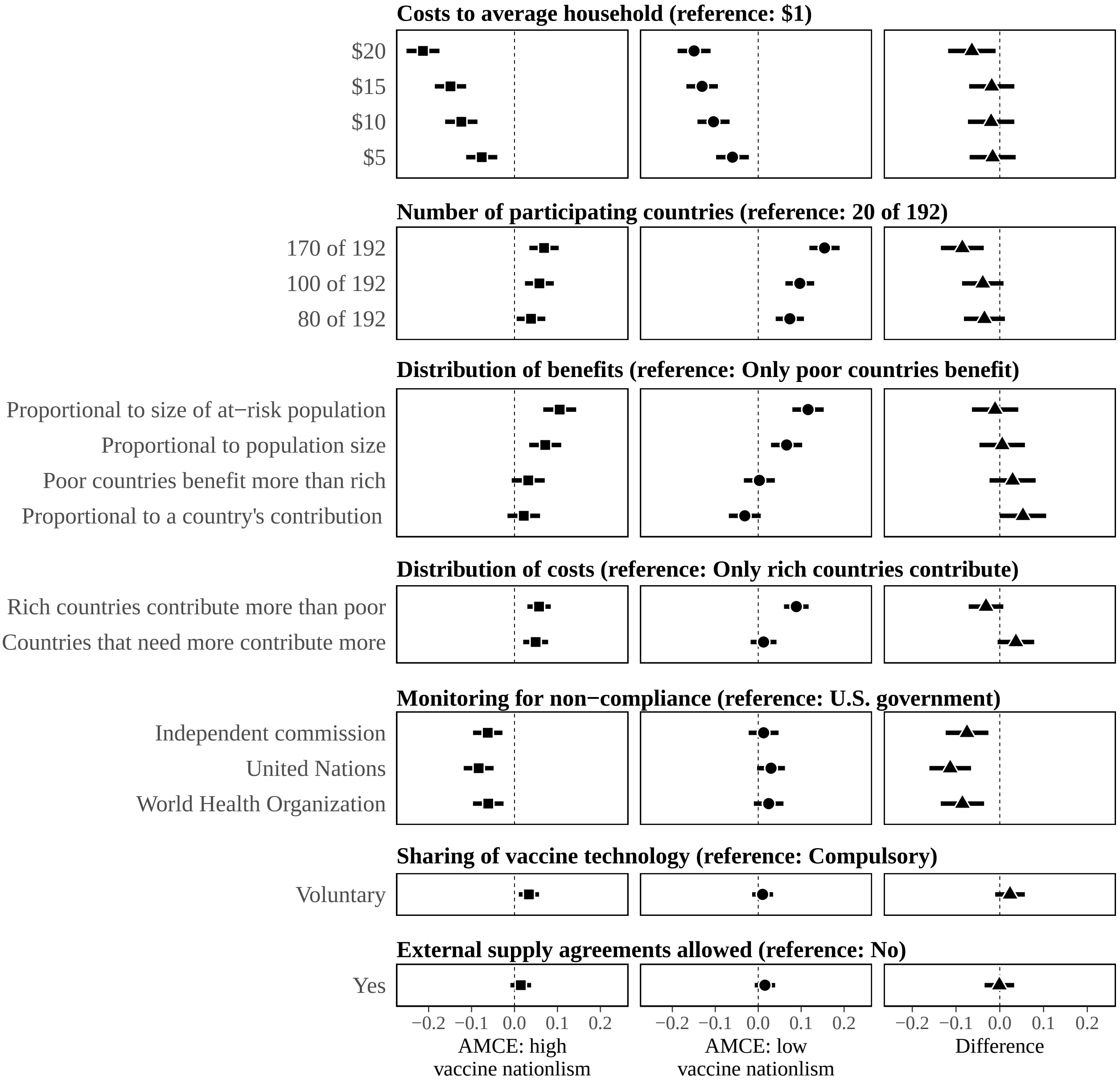

Notes: Sub-group estimates showing average marginal component effects (AMCEs) among vaccine nationalists, non-nationalists, and the differences. Sub-group classifications are based on scores derived from the 5-item vaccine nationalism index; vaccine nationalism is coded as high if a respondent scored higher than the median respondent. Point estimates and 95% confidence intervals estimated via OLS regression with robust standard errors clustered at respondent level to correct for within-respondent clustering. Lucid survey of U.S. adults fielded in April 2021 (N = 1,751 respondents x 4 pairings x 2 agreements per pair = 14,008 observations).

Figure S43: Estimated marginal means in international agreement conjoint (Lucid) by nationalism

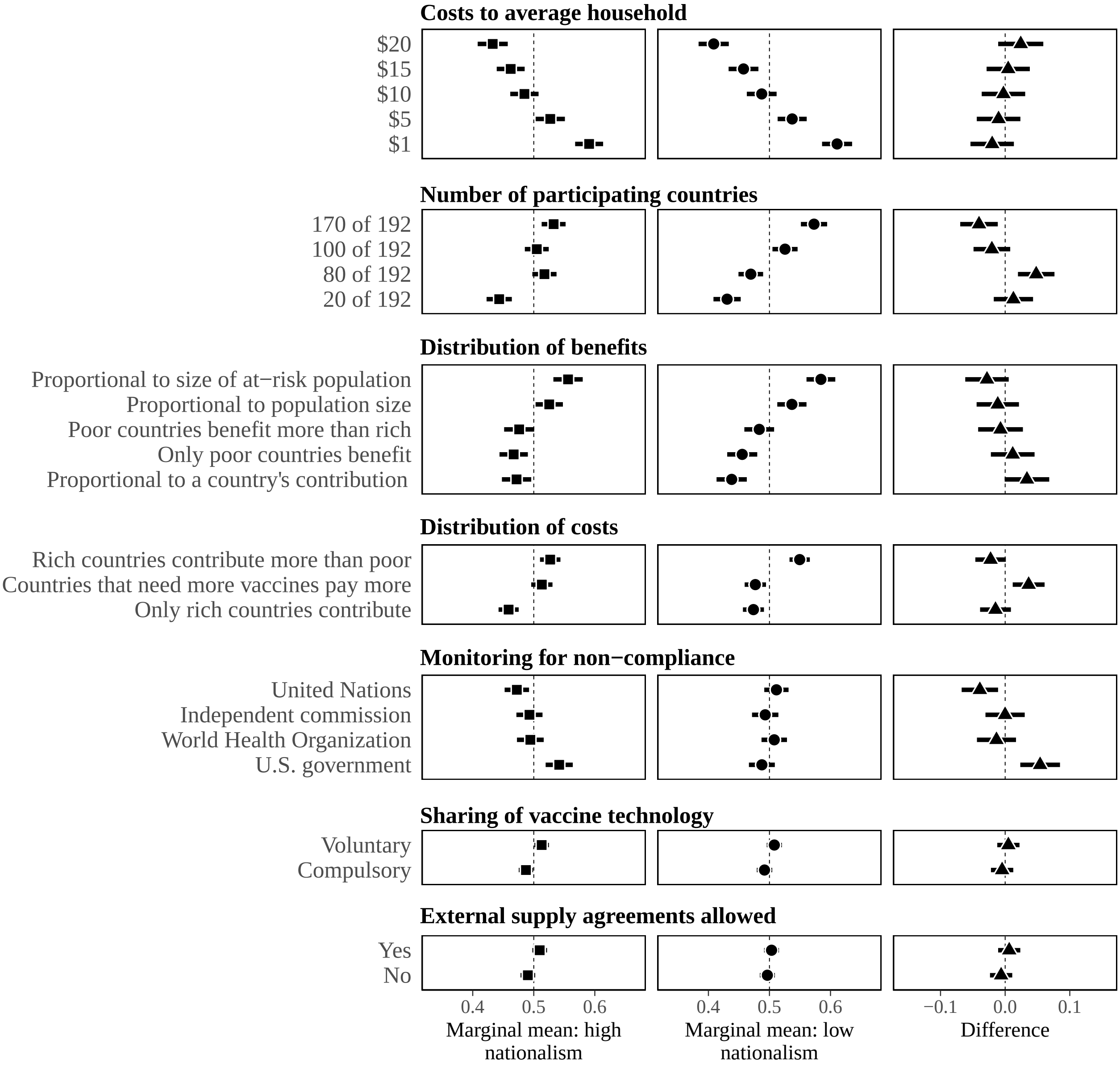

Notes: Sub-group estimates showing marginal means among nnationalists, non-nationalists, and the differences. Sub-group classifications are based on scores derived from a 7-point nationalism scale; nationalism is coded as high if a respondent scored higher than the median respondent. Point estimates and 95% confidence intervals estimated via OLS regression with robust standard errors clustered at respondent level to correct for within-respondent clustering. Lucid survey of U.S. adults fielded in April 2021 (N = 1,751 respondents x 4 pairings x 2 agreements per pair = 14,008 observations).

Figure S44: Estimated AMCEs in international agreement conjoint (Lucid) by nationalism

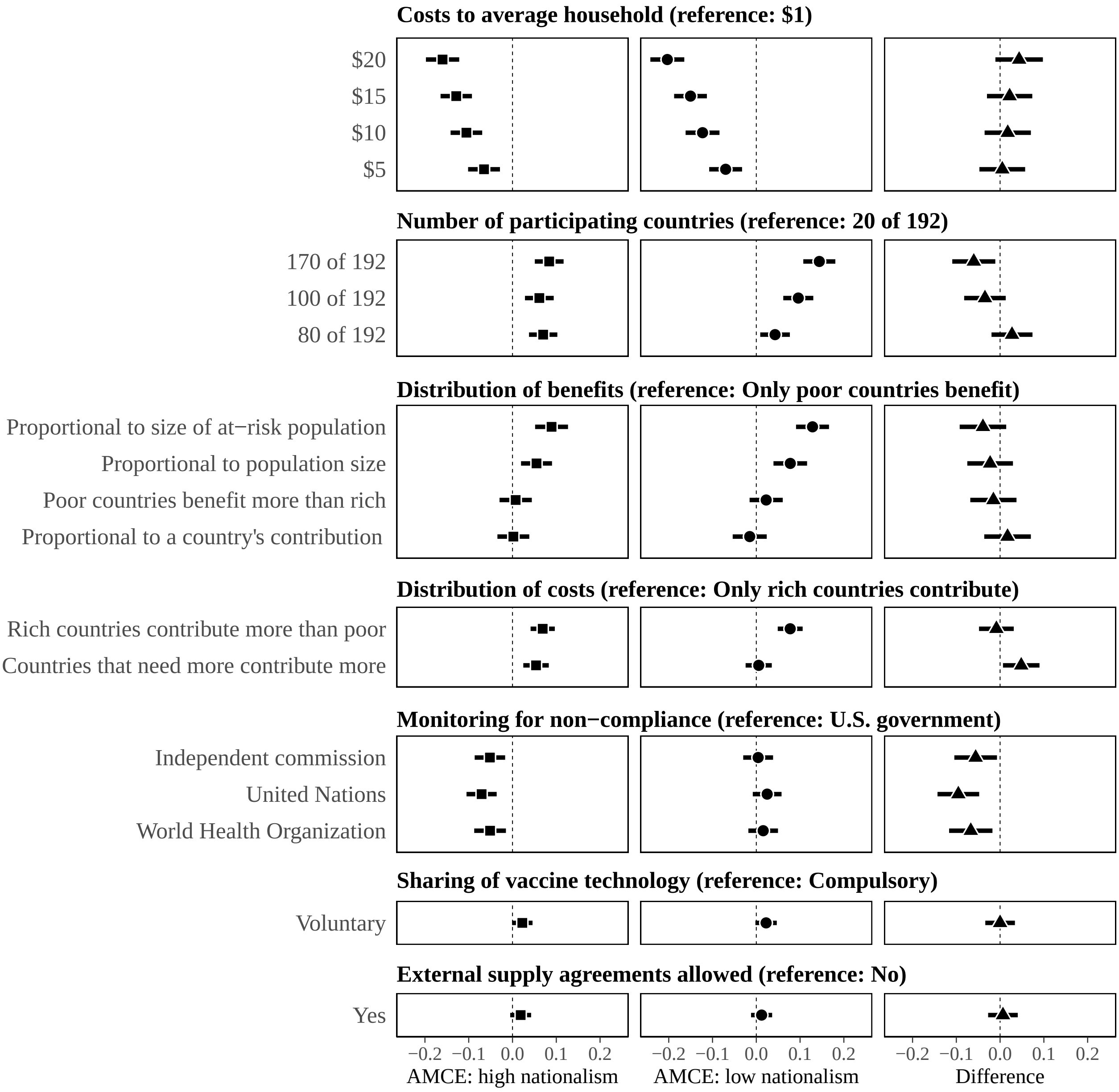

Notes: Sub-group estimates showing average marginal component effects (AMCEs) among nationalists, non-nationalists, and the differences. Sub-group classifications are based on scores derived from a 7-point nationalism scale; nationalism is coded as high if a respondent scored higher than the median respondent.Point estimates and 95% confidence intervals estimated via OLS regression with robust standard errors clustered at respondent level to correct for within-respondent clustering. Lucid survey of U.S. adults fielded in April 2021 (N = 1,751 respondents x 4 pairings x 2 agreements per pair = 14,008 observations).

Figure S45: Estimated marginal means in international agreement conjoint (Lucid) by cosmopolitanism

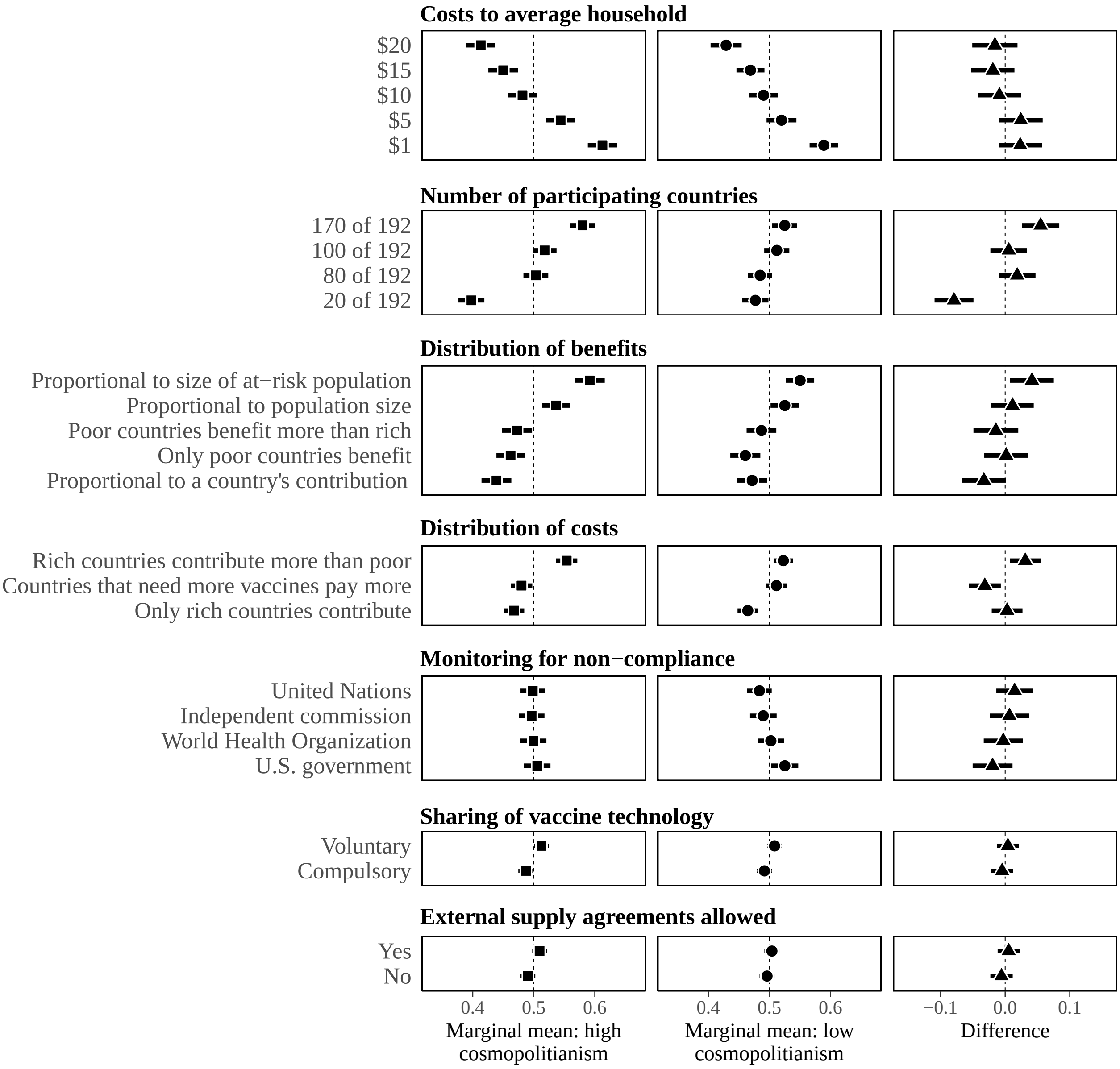

Notes: Sub-group estimates showing marginal means among those high in cosmopolitanism, low in cosmopolitanism, and the differences. Sub-group classifications are based on scores derived from a 7-point cosmopolitanism scale; cosmopolitanism is coded as high if a respondent scored higher than the median respondent. Point estimates and 95% confidence intervals estimated via OLS regression with robust standard errors clustered at respondent level to correct for within-respondent clustering. Lucid survey of U.S. adults fielded in April 2021 (N = 1,751 respondents x 4 pairings x 2 agreements per pair = 14,008 observations).

Figure S46: Estimated AMCEs in international agreement conjoint (Lucid) by cosmopolitanism

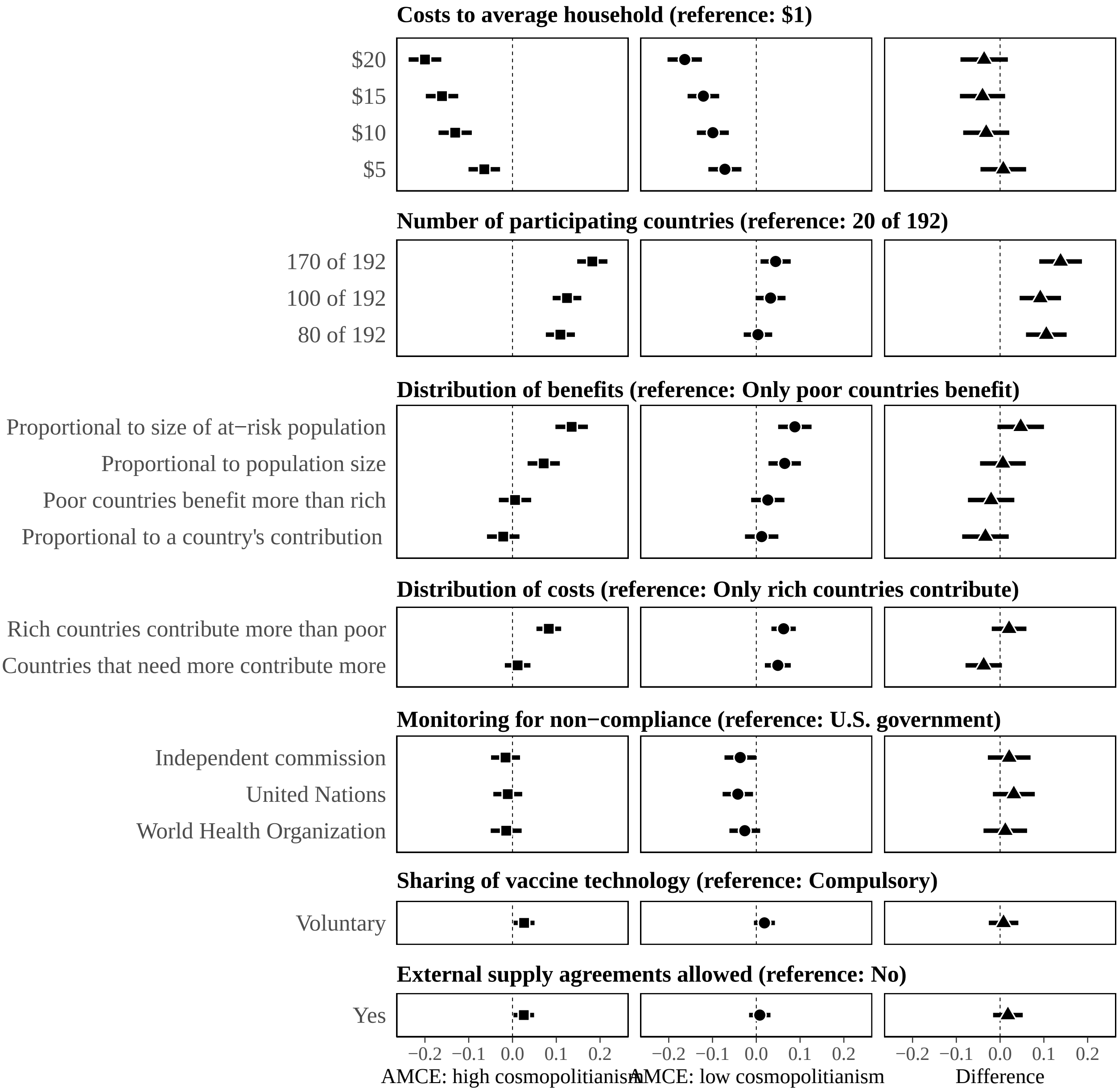

Notes: Sub-group estimates showing average marginal component effects (AMCEs) among those high in cosmopolitanism, low in cosmopolitanism and the differences. Sub-group classifications are based on scores derived from a 7-point cosmopolitanism scale; cosmopolitanism is coded as high if a respondent scored higher than the median respondent. Point estimates and 95% confidence intervals estimated via OLS regression with robust standard errors clustered at respondent level to correct for within-respondent clustering. Lucid survey of U.S. adults fielded in April 2021 (N = 1,751 respondents x 4 pairings x 2 agreements per pair = 14,008 observations).

Figure S47: Estimated marginal means in international agreement conjoint (Lucid) by altruism

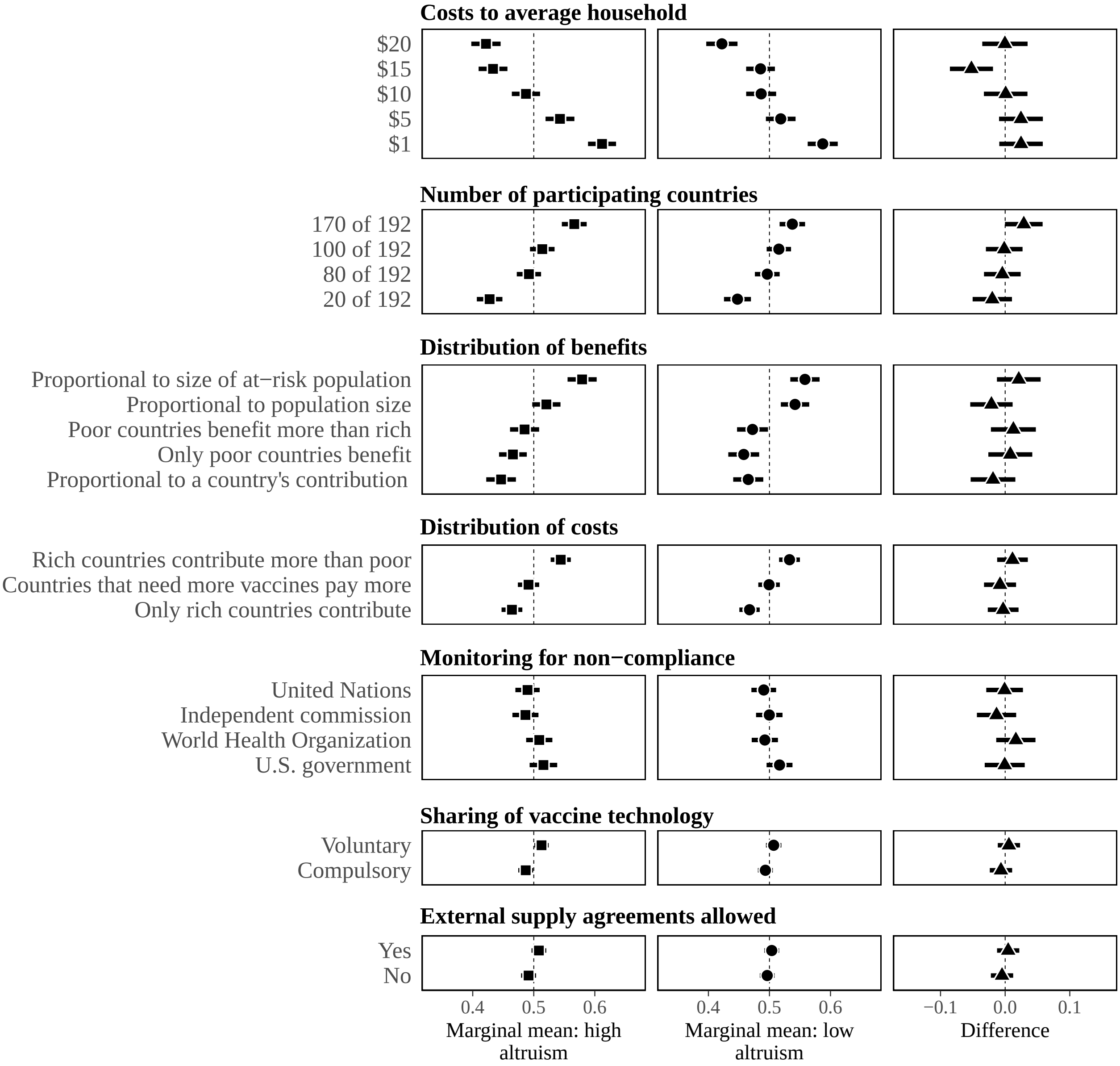

Notes: Sub-group estimates showing marginal means among those high in altruism, low in altruism, and the differences. Sub-group classifications are based on scores derived from a 2-item altruism index; altruism is coded as high if a respondent scored higher than the median respondent. Point estimates and 95% confidence intervals estimated via OLS regression with robust standard errors clustered at respondent level to correct for within-respondent clustering. Lucid survey of U.S. adults fielded in April 2021 (N = 1,751 respondents x 4 pairings x 2 agreements per pair = 14,008 observations).

Figure S48: Estimated AMCEs in international agreement conjoint (Lucid) by altruism

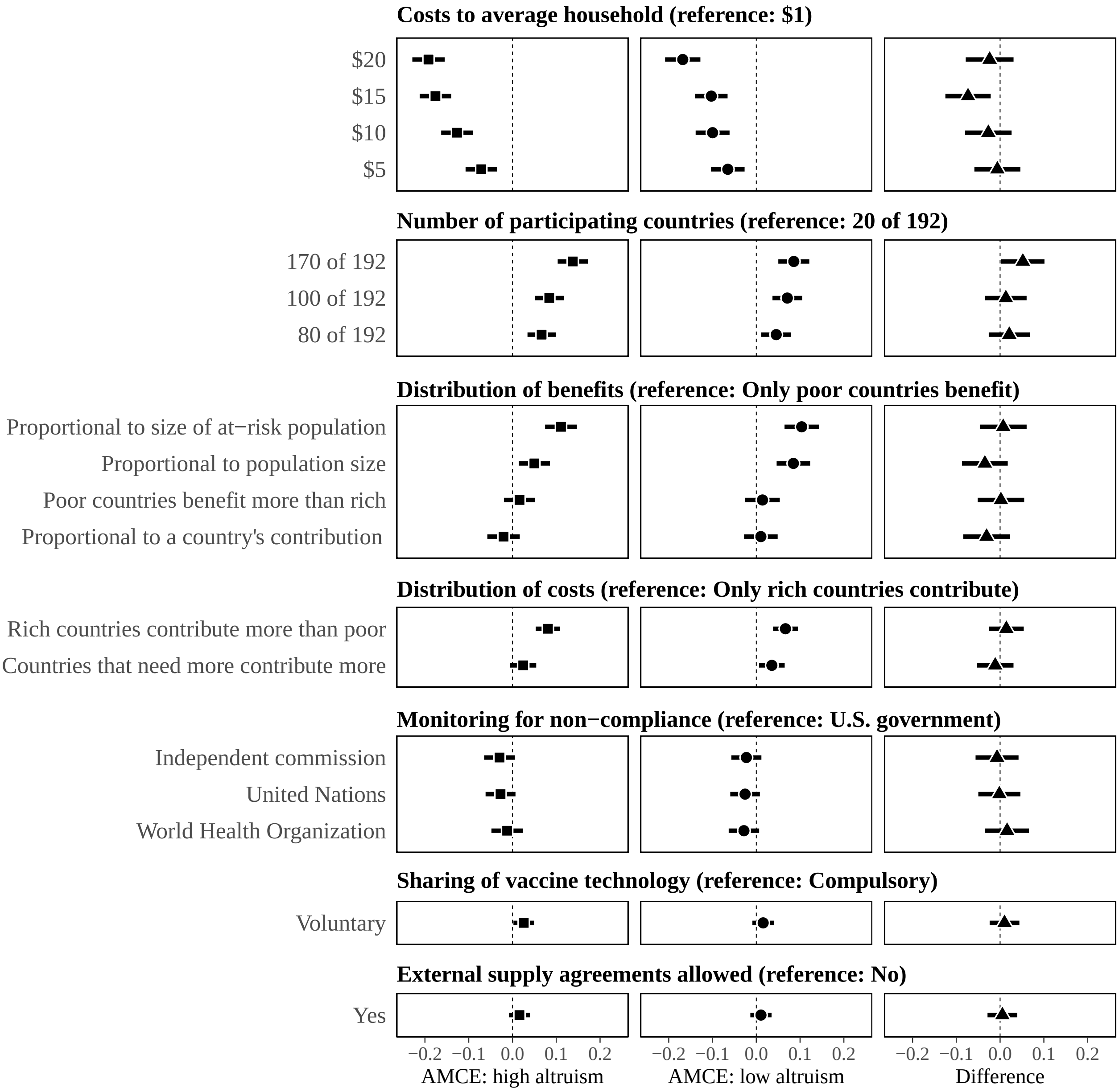

Notes: Sub-group estimates showing average marginal component effects (AMCEs) among those high in altruism, low in altruism, and the differences. Sub-group classifications are based on scores derived from a 2-item altruism index; altruism is coded as high if a respondent scored higher than the median respondent. Point estimates and 95% confidence intervals estimated via OLS regression with robust standard errors clustered at respondent level to correct for within-respondent clustering. Lucid survey of U.S. adults fielded in April 2021 (N = 1,751 respondents x 4 pairings x 2 agreements per pair = 14,008 observations).

Figure S49: Estimated marginal means in international agreement conjoint (Lucid) by reciprocity

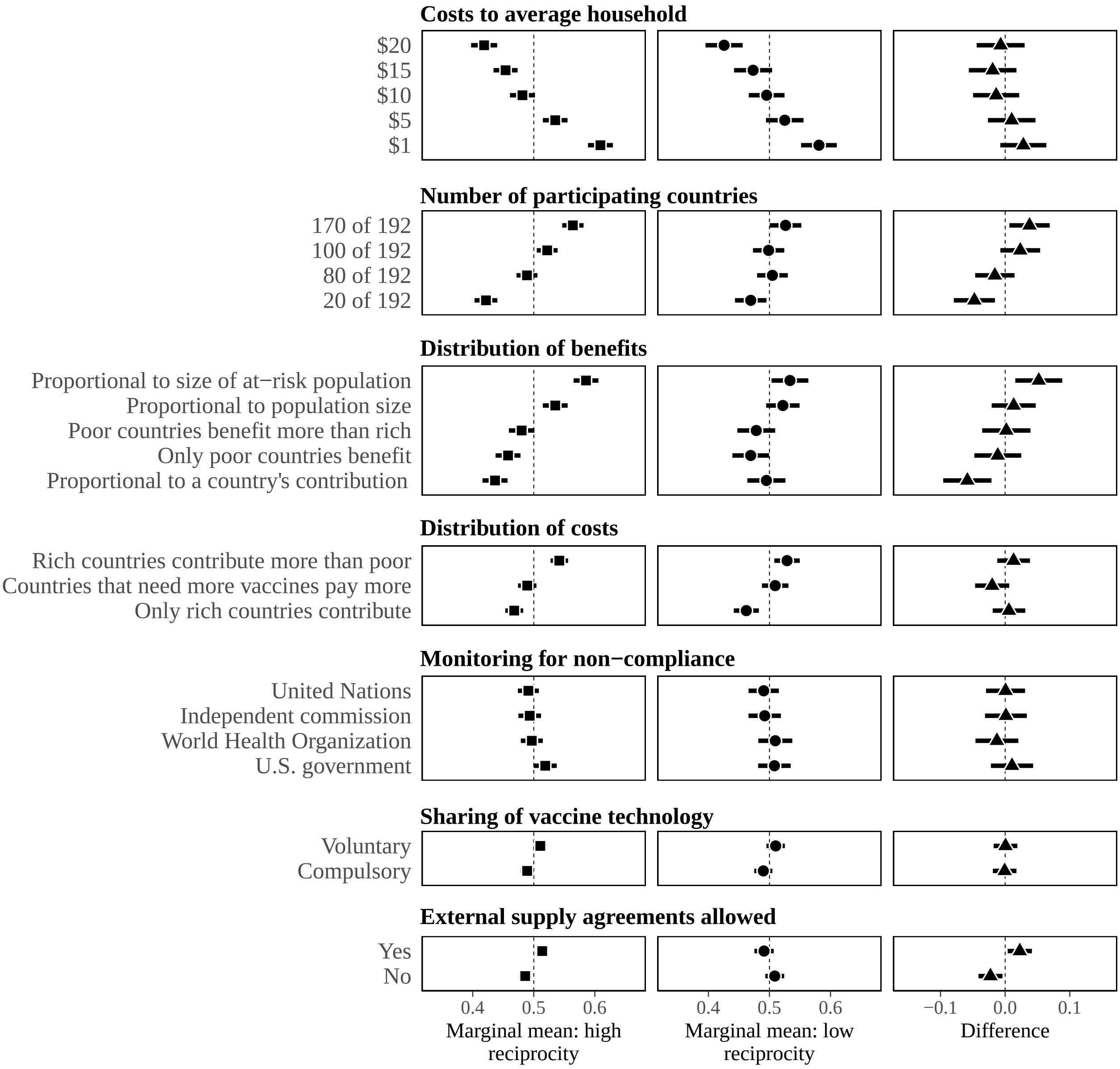

Notes: Sub-group estimates showing marginal means among those high in reciprocity, low in reciprocity, and the differences. Sub-group classifications are based on scores derived from the hypothetical gift-giving scenario; reciprocity is coded as high if a respondent scored higher than the median respondent. Point estimates and 95% confidence intervals estimated via OLS regression with robust standard errors clustered at respondent level to correct for within-respondent clustering. Lucid survey of U.S. adults fielded in April 2021 (N = 1,751 respondents x 4 pairings x 2 agreements per pair = 14,008 observations).

Figure S50: Estimated AMCEs in international agreement conjoint (Lucid) by reciprocity

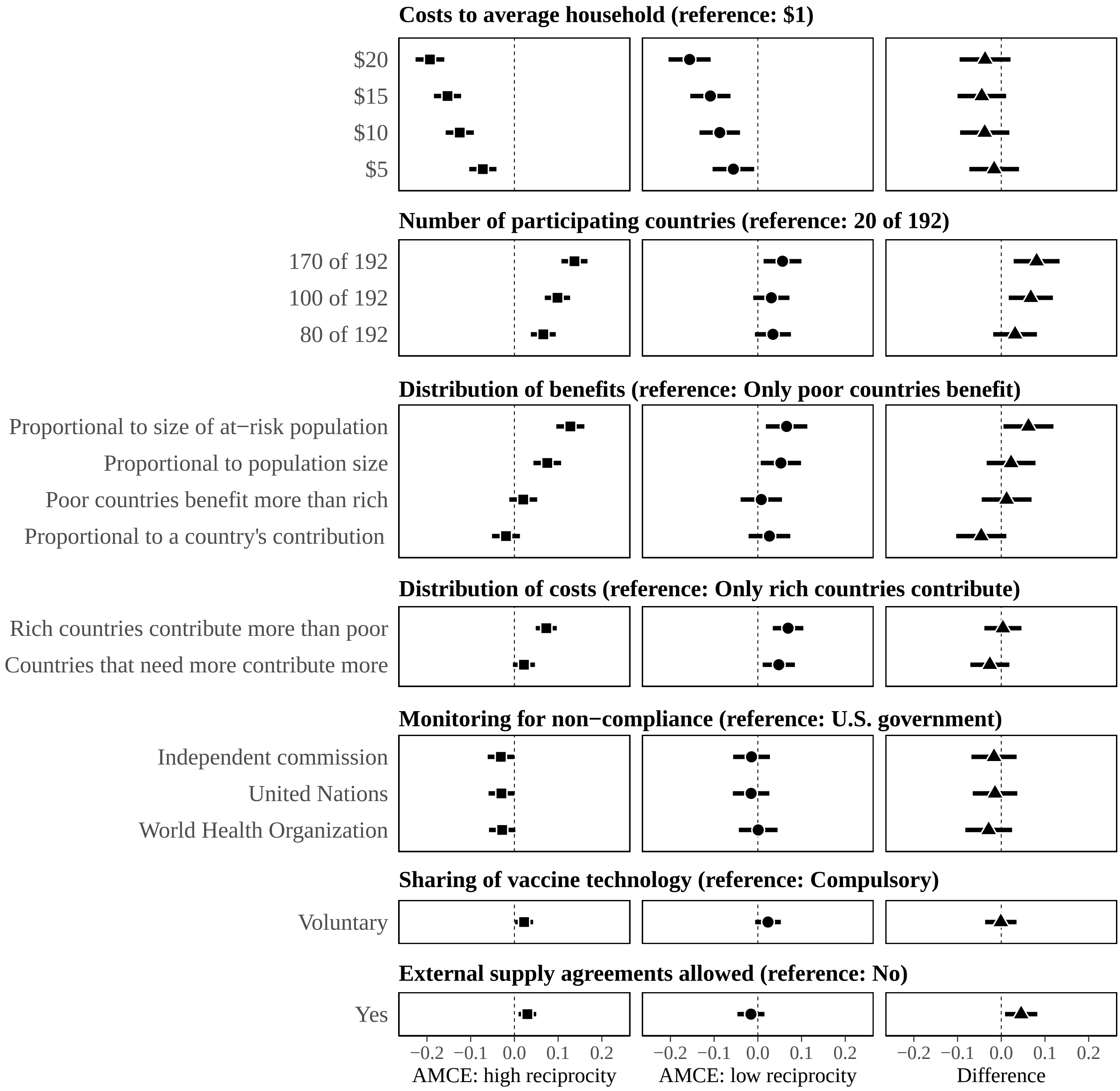

Notes: Sub-group estimates showing average marginal component effects (AMCEs) among those high in reciprocity, low in reciprocity, and the differences. Sub-group classifications are based on scores derived from the hypothetical gift-giving scenario; reciprocity is coded as high if a respondent scored higher than the median respondent. Point estimates and 95% confidence intervals estimated via OLS regression with robust standard errors clustered at respondent level to correct for within-respondent clustering. Lucid survey of U.S. adults fielded in April 2021 (N = 1,751 respondents x 4 pairings x 2 agreements per pair = 14,008 observations).

Figure S51: Estimated marginal means in international agreement conjoint (Lucid) by vaccination status

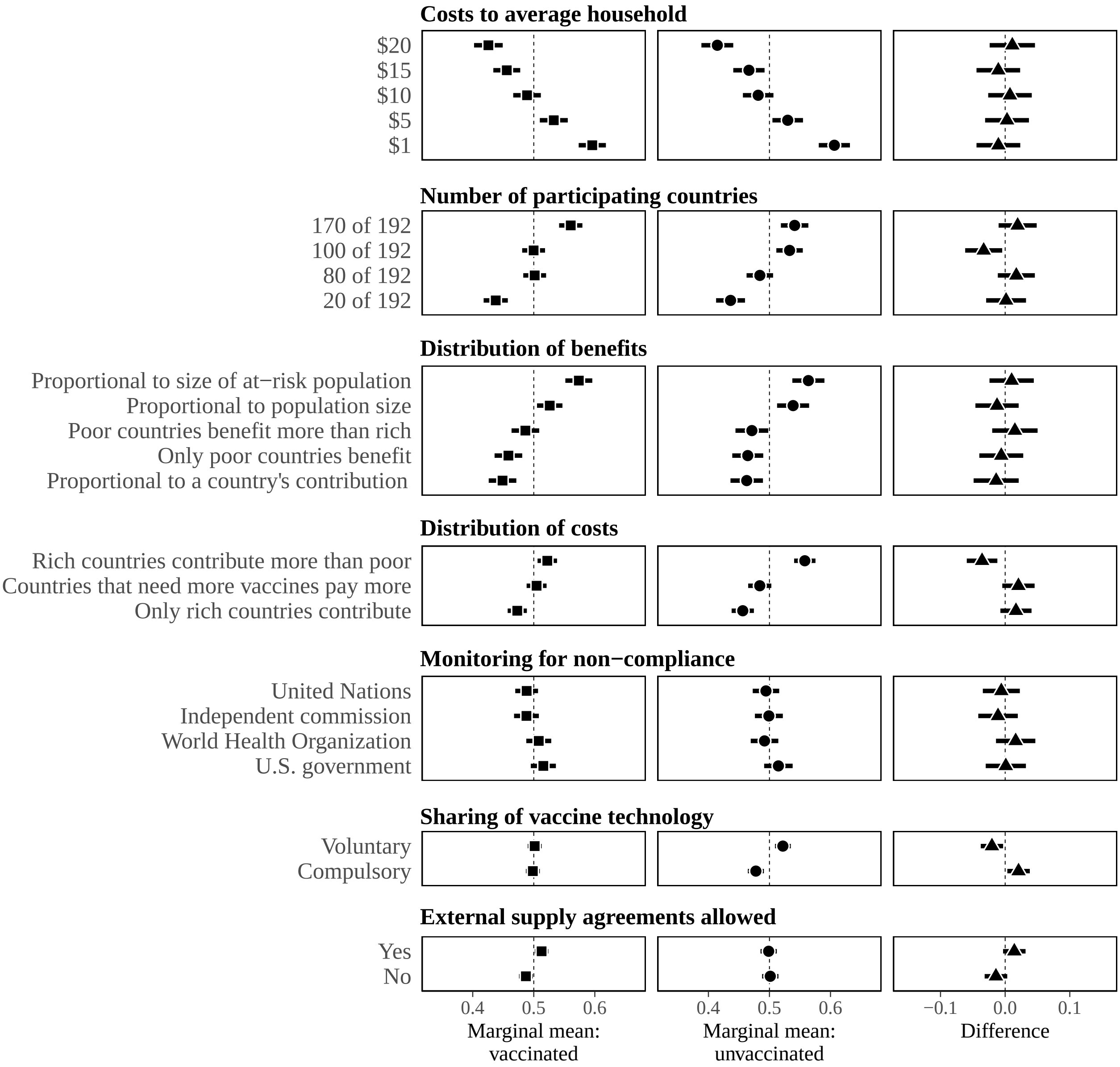

Notes: Sub-group estimates showing marginal means among those who had received a vaccination, had not received a vaccinated, and the differences. Individuals that reported receiving at least one dose of any COVID-19 vaccine at the time of the survey were classified as having received a vaccination. Point estimates and 95% confidence intervals estimated via OLS regression with robust standard errors clustered at respondent level to correct for within-respondent clustering. Lucid survey of U.S. adults fielded in April 2021 (N = 1,751 respondents x 4 pairings x 2 agreements per pair = 14,008 observations).

Figure S52: Estimated AMCEs in international agreement conjoint (Lucid) by vaccination status

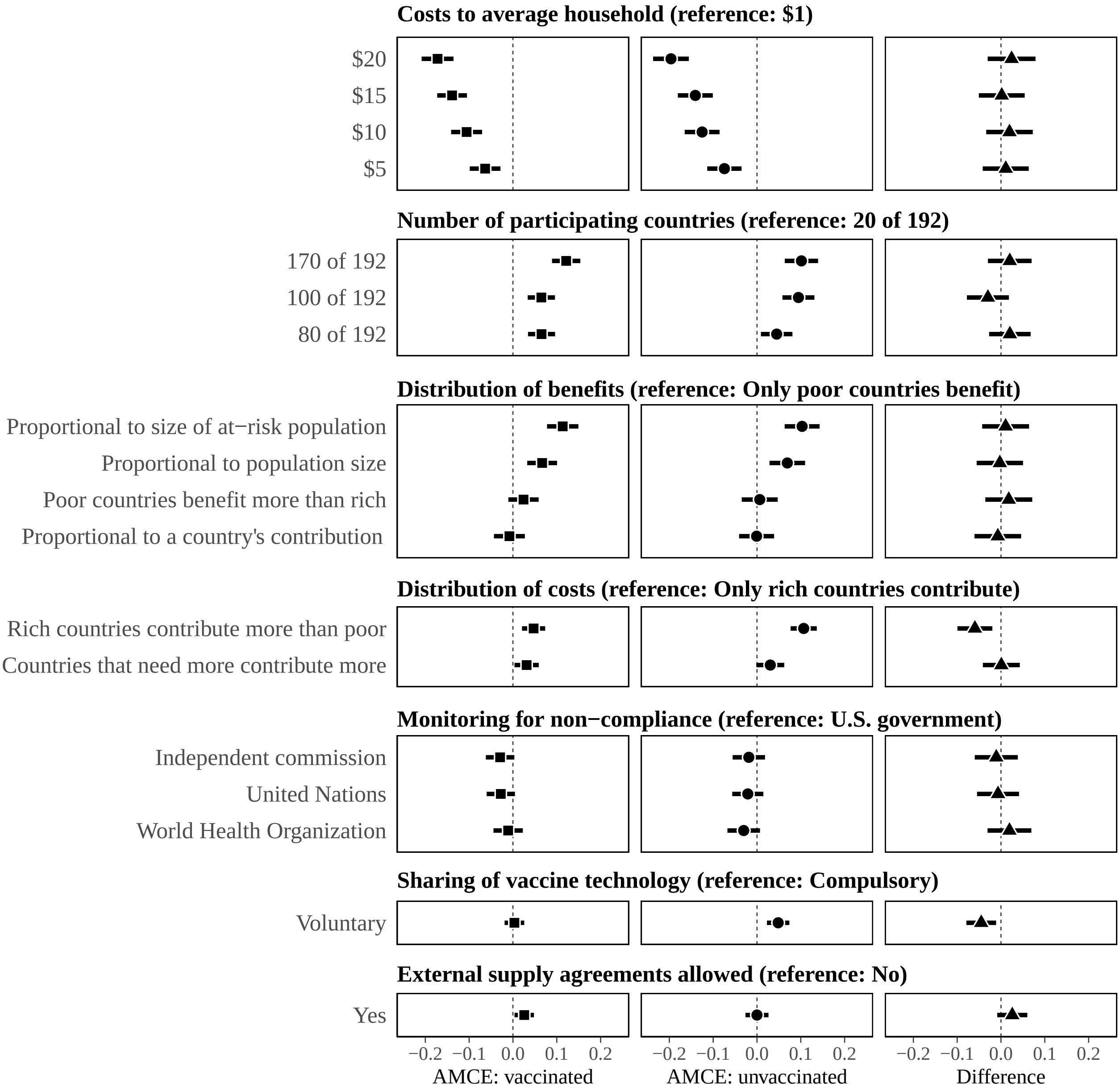

Notes: Sub-group estimates showing average marginal component effects (AMCEs) aamong those who had received a vaccination, had not received a vaccinated, and the differences. Individuals that reported receiving at least one dose of any COVID-19 vaccine at the time of the survey were classified as having received a vaccination. Point estimates and 95% confidence intervals estimated via OLS regression with robust standard errors clustered at respondent level to correct for within-respondent clustering. Lucid survey of U.S. adults fielded in April 2021 (N = 1,751 respondents x 4 pairings x 2 agreements per pair = 14,008 observations).

Figure S53: Estimated marginal means in international agreement conjoint (Lucid) by partisanship

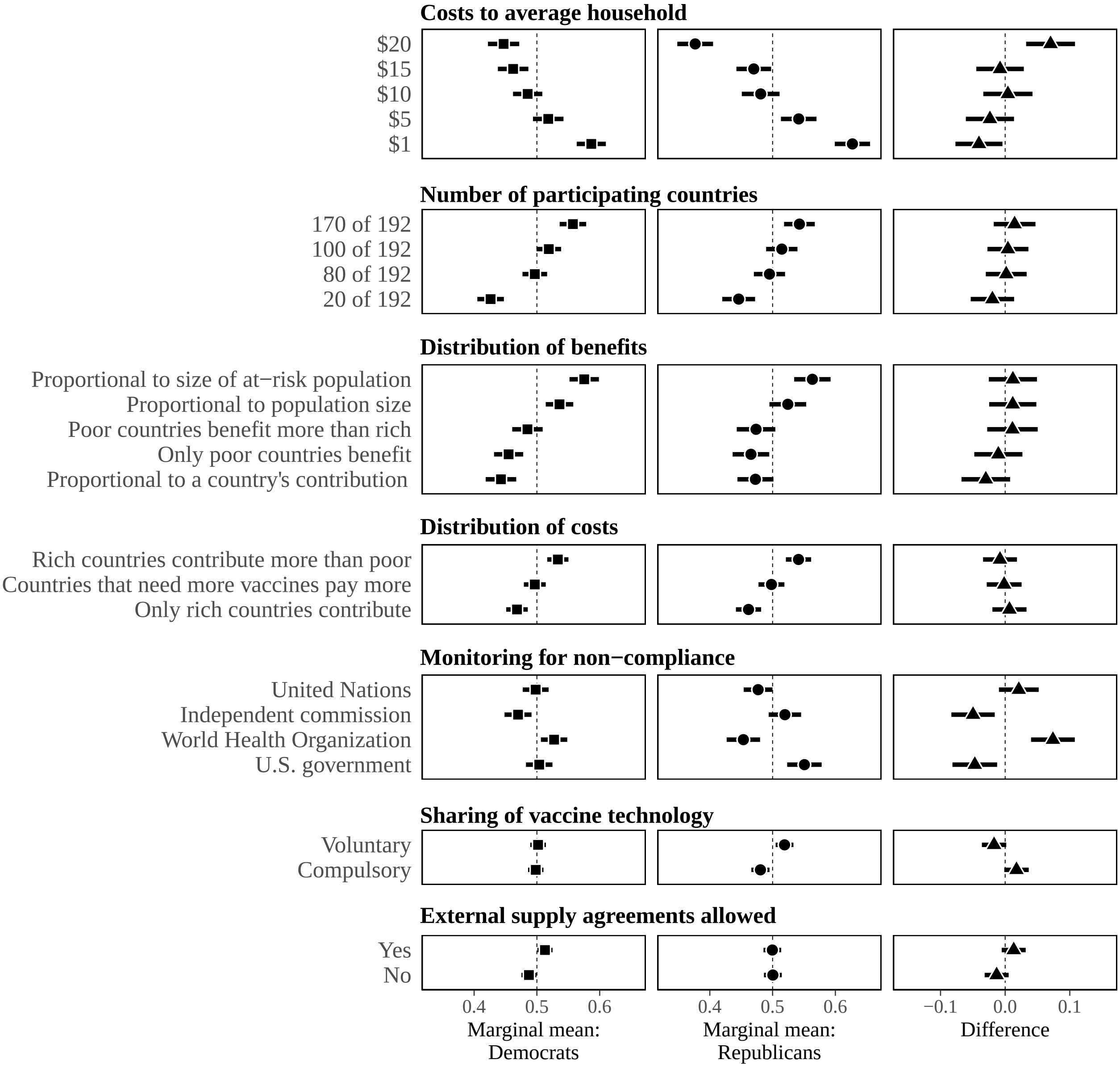

Notes: Sub-group estimates showing marginal means among Democrats, Republicans, and the differences. Partisanship is measured with the standard 7-point branching question used in the American National Election Studies (ANES) Survey. Therefore, Democrat and Republican sub-groups include weak supporters, strong supporters, and those that lean toward one party or another, whereas Independents do not lean one way or the other and are excluded. Point estimates and 95% confidence intervals estimated via OLS regression with robust standard errors clustered at respondent level to correct for within-respondent clustering. Lucid survey of U.S. adults fielded in April 2021 (N = 1,751 respondents x 4 pairings x 2 agreements per pair = 14,008 observations).

Figure S54: Estimated AMCEs in international agreement conjoint (Lucid) by partisanship

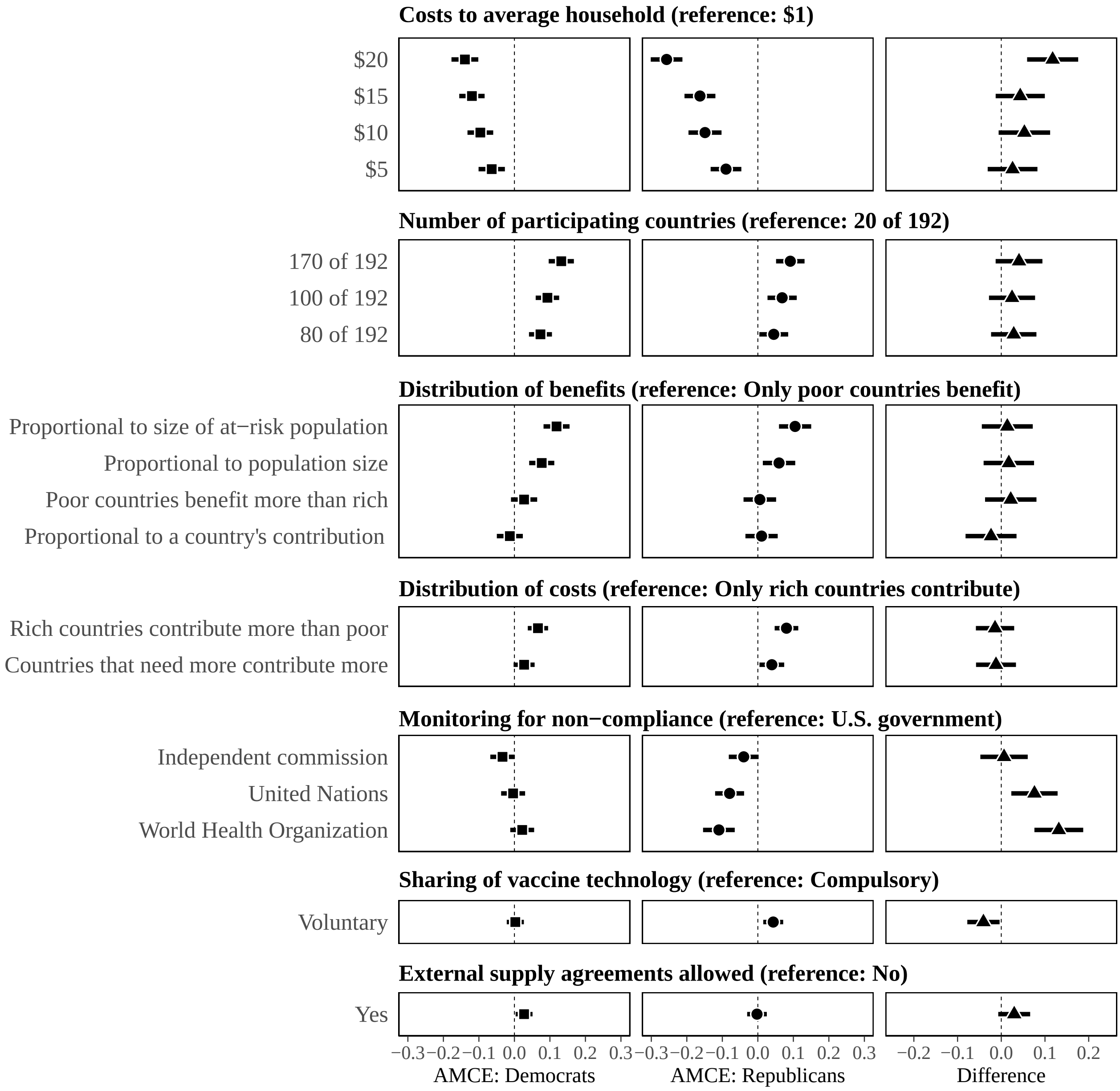

Notes: Sub-group estimates showing average marginal component effects (AMCEs) among Democrats, Republicans, and the differences. Partisanship is measured with the standard 7-point branching question used in the American National Election Studies (ANES) Survey. Therefore, Democrat and Republican sub-groups include weak supporters, strong supporters, and those that lean toward one party or another, whereas Independents do not lean one way or the other and are excluded. Point estimates and 95% confidence intervals estimated via OLS regression with robust standard errors clustered at respondent level to correct for within-respondent clustering. Lucid survey of U.S. adults fielded in April 2021 (N = 1,751 respondents x 4 pairings x 2 agreements per pair = 14,008 observations).

Figure S55: Estimated marginal means in international agreement conjoint (Lucid) by political ideology

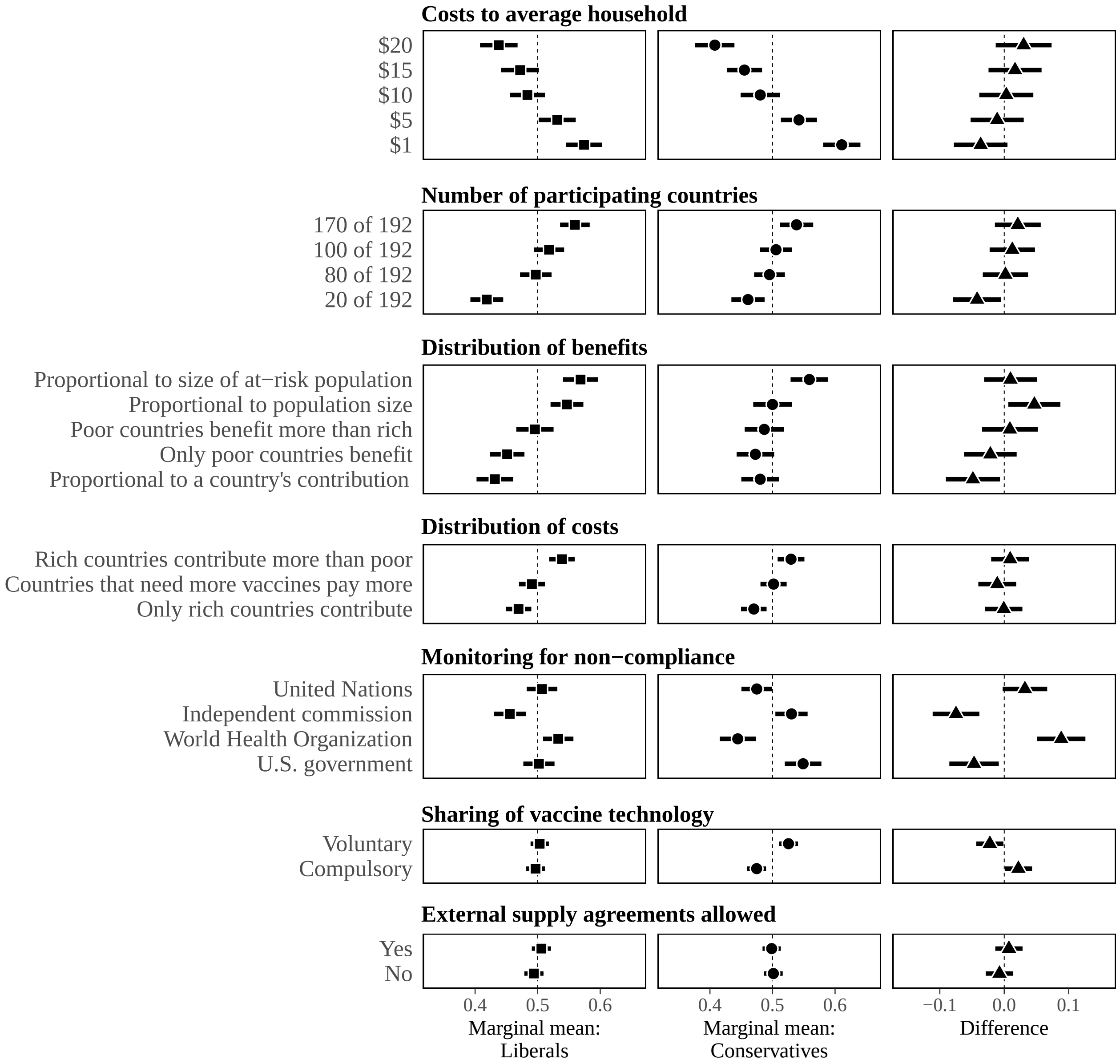

Notes: Sub-group estimates showing marginal means among Liberals, Conservatives, and the differences. Ideology is measured with the standard 7-point scale from "Extremely Liberal" to "Extremely Conservative", and moderates (those at the midpoint of the scale) are excluded. Point estimates and 95% confidence intervals estimated via OLS regression with robust standard errors clustered at respondent level to correct for within-respondent clustering. Lucid survey of U.S. adults fielded in April 2021 (N = 1,751 respondents x 4 pairings x 2 agreements per pair = 14,008 observations).

Figure S56: Estimated AMCEs in international agreement conjoint (Lucid) by political ideology

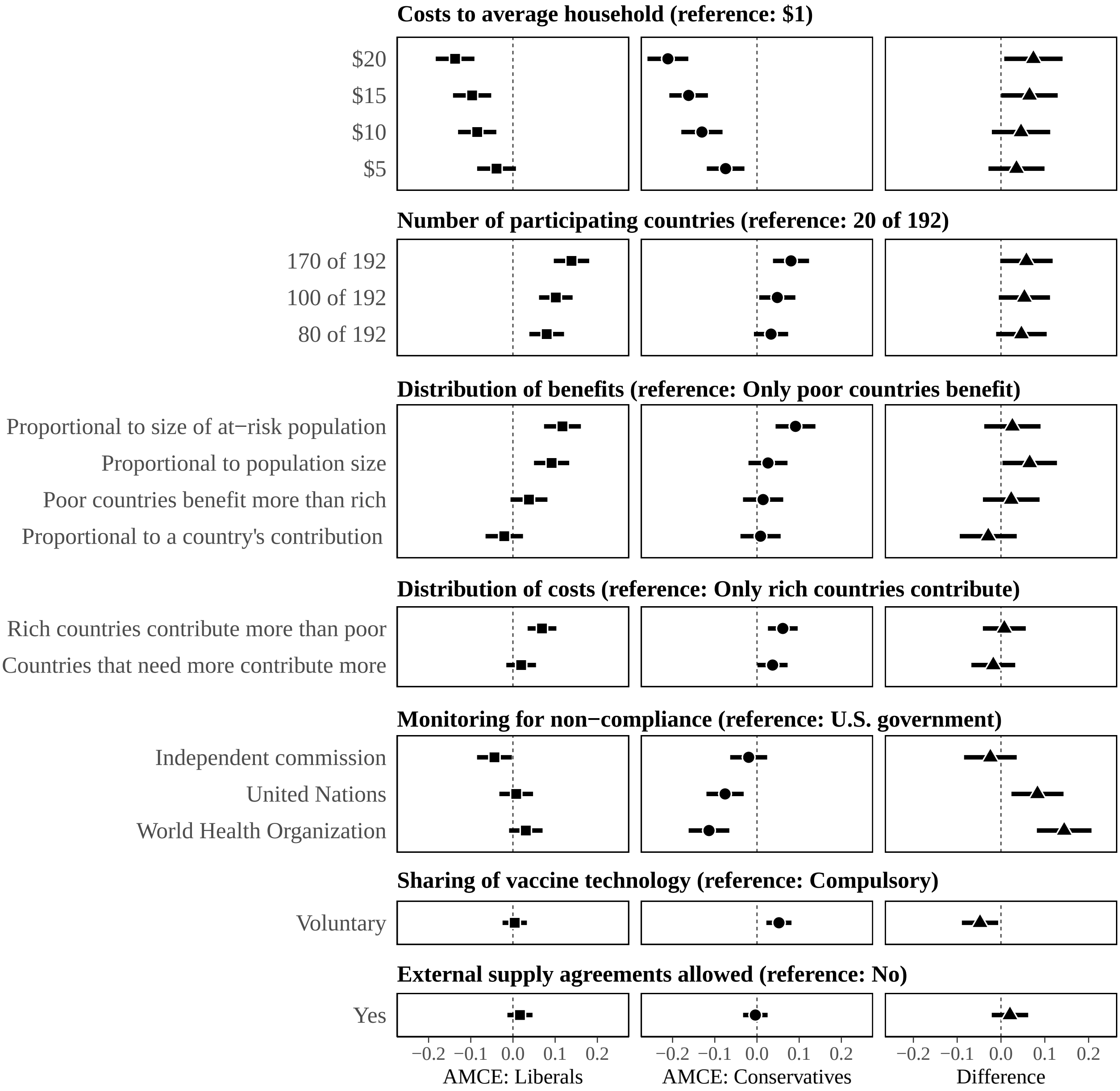

Notes: Sub-group estimates showing average marginal component effects (AMCEs) among Liberals, Conservatives, and the differences. Ideology is measured with the standard 7-point scale from "Extremely Liberal" to "Extremely Conservative", and moderates (those at the midpoint of the scale) are excluded. Point estimates and 95% confidence intervals estimated via OLS regression with robust standard errors clustered at respondent level to correct for within-respondent clustering. Lucid survey of U.S. adults fielded in April 2021 (N = 1,751 respondents x 4 pairings x 2 agreements per pair = 14,008 observations).

S3.7 Effect heterogeneity in international agreement conjoint (NORC) by respondents' background characteristics

Figure S57: Estimated marginal means in international agreement conjoint (NORC) by vaccine nationalism

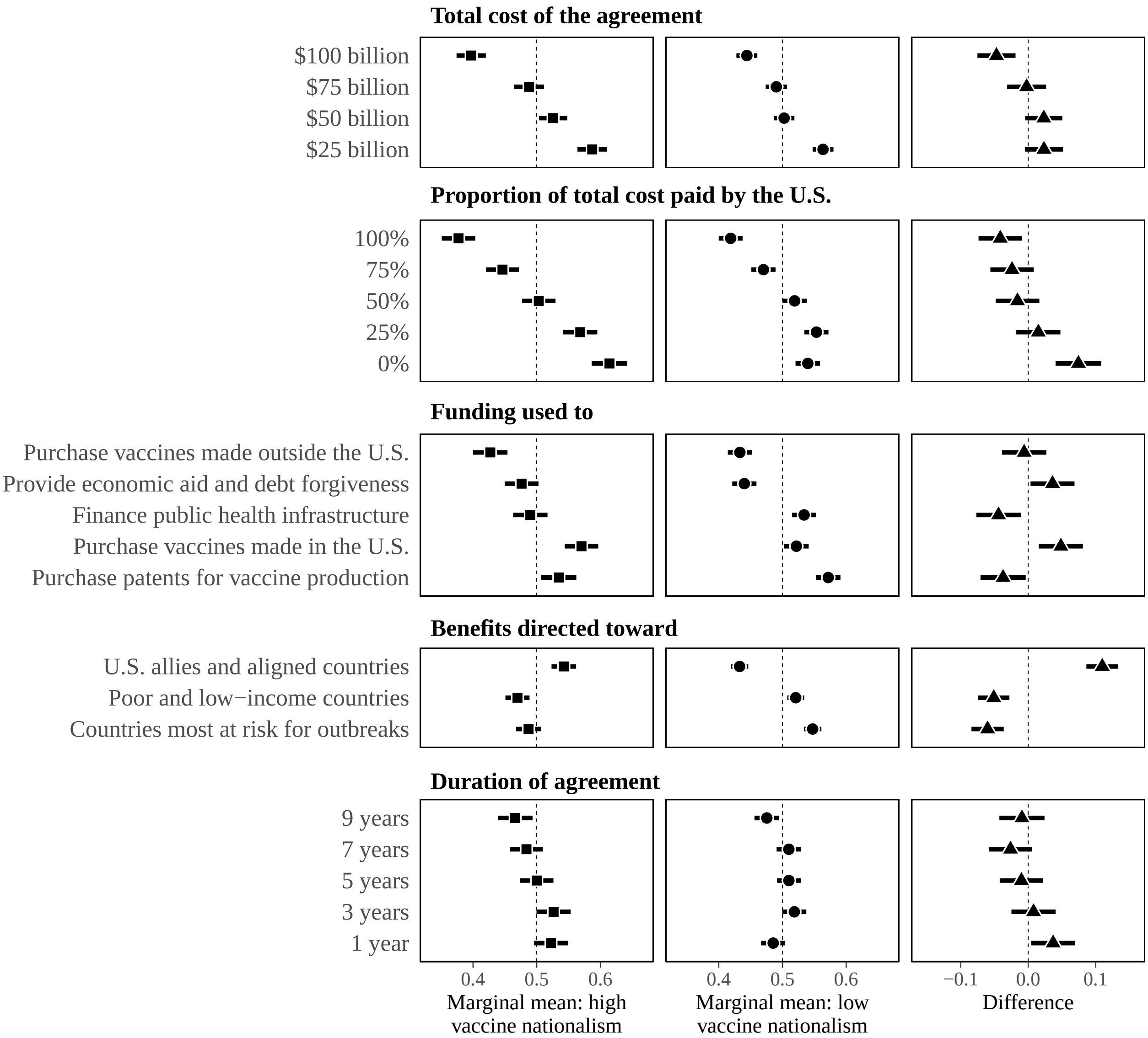

Notes: Sub-group estimates showing marginal means among those high in vaccine nationalism, low in vaccine nationalism, and the differences. Sub-group classifications are based on scores derived from the vaccine nationalism scale described in Section S2.1; vaccine nationalism is coded as high if a respondent scored higher than the median respondent. Point estimates and 95% confidence intervals estimated via OLS regression with robust standard errors clustered at respondent level to correct for within-respondent clustering. NORC/Amerispeak survey of U.S. adults fielded in September/October 2021 (N = 4,214 respondents x 2 pairings x 2 agreements per pair = 16,856 observations).

Figure S58: Estimated AMCEs in international agreement conjoint (NORC) by vaccine nationalism

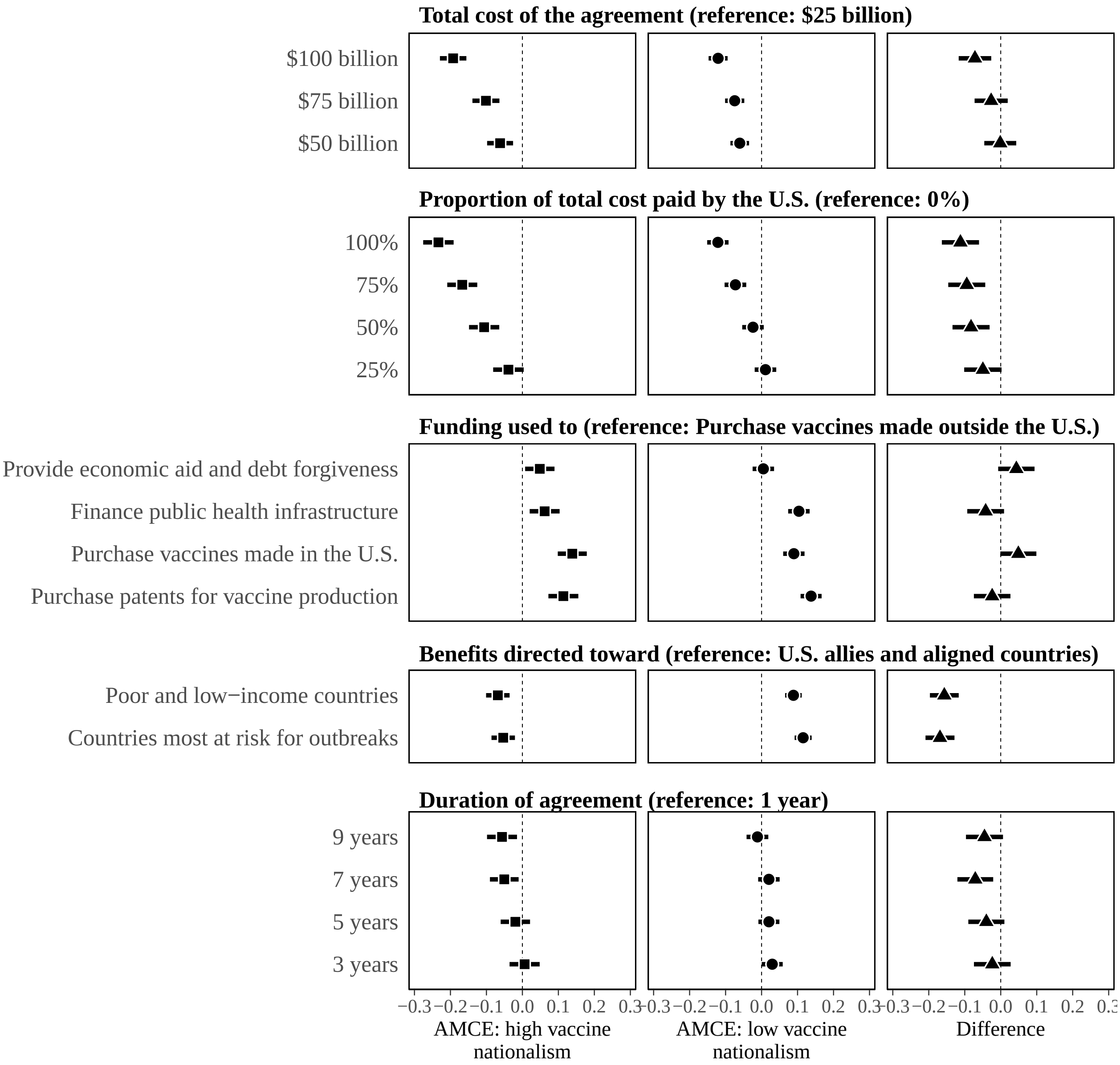

Notes: Sub-group estimates showing average marginal component effects (AMCEs) among those high in vaccine nationalism, low in vaccine nationalism, and the differences. Sub-group classifications are based on scores derived from the vaccine nationalism scale described in Section S2.1; vaccine nationalism is coded as high if a respondent scored higher than the median respondent. Point estimates and 95% confidence intervals estimated via OLS regression with robust standard errors clustered at respondent level to correct for within-respondent clustering. NORC/Amerispeak survey of U.S. adults fielded in September/October 2021 (N = 4,214 respondents x 2 pairings x 2 agreements per pair = 16,856 observations).

Figure S59: Estimated marginal means in international agreement conjoint (NORC) by nationalism

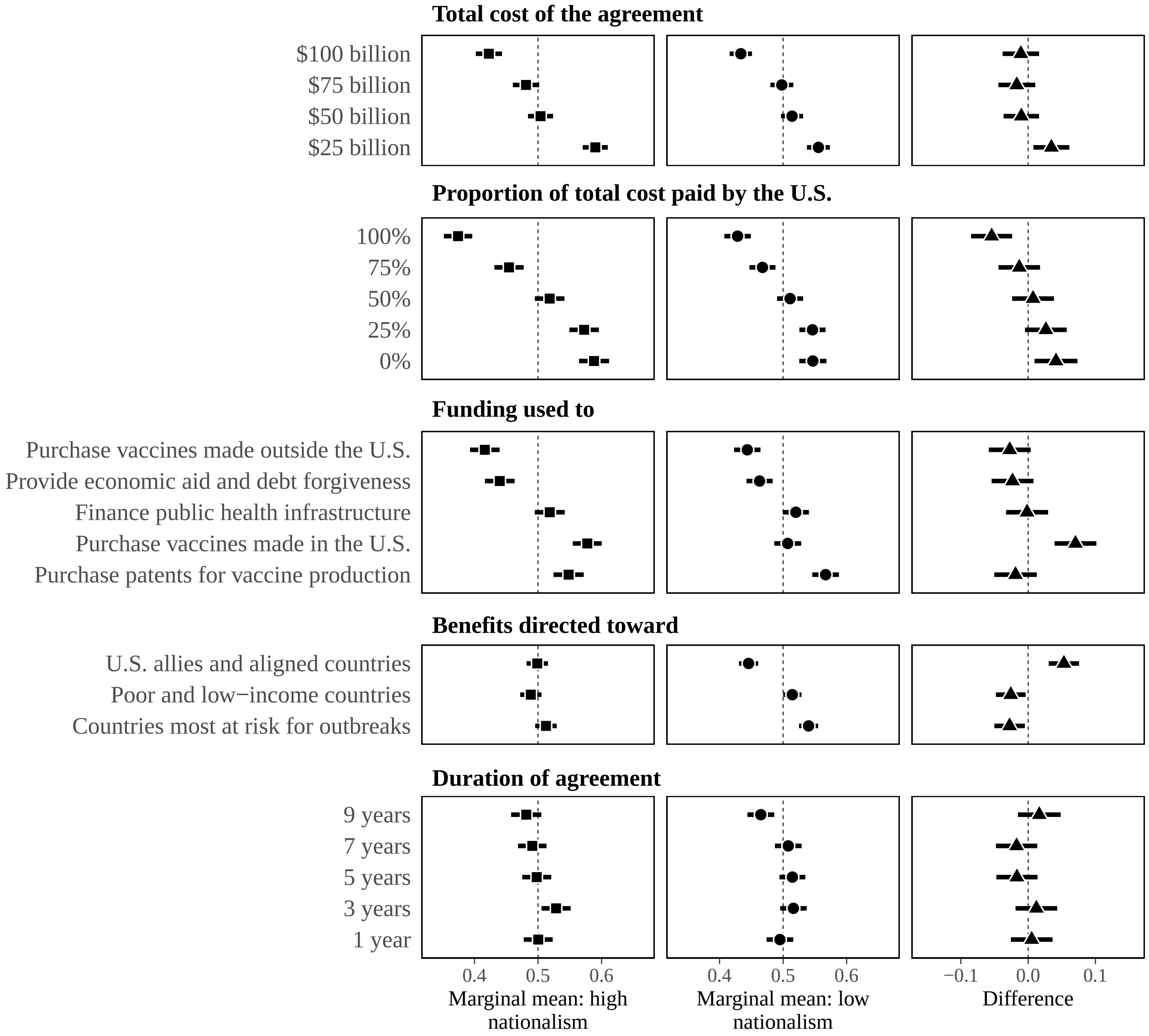

Notes: Sub-group estimates showing marginal means among those high in nationalism, low in nationalism, and the differences. Sub-group classifications are based on scores derived from the nationalism scale described in Section S2.1; nationalism is coded as high if a respondent scored higher than the median respondent. Point estimates and 95% confidence intervals estimated via OLS regression with robust standard errors clustered at respondent level to correct for within-respondent clustering. NORC/Amerispeak survey of U.S. adults fielded in September/October 2021 (N = 4,214 respondents x 2 pairings x 2 agreements per pair = 16,856 observations).

Figure S60: Estimated AMCEs in international agreement conjoint (NORC) by nationalism

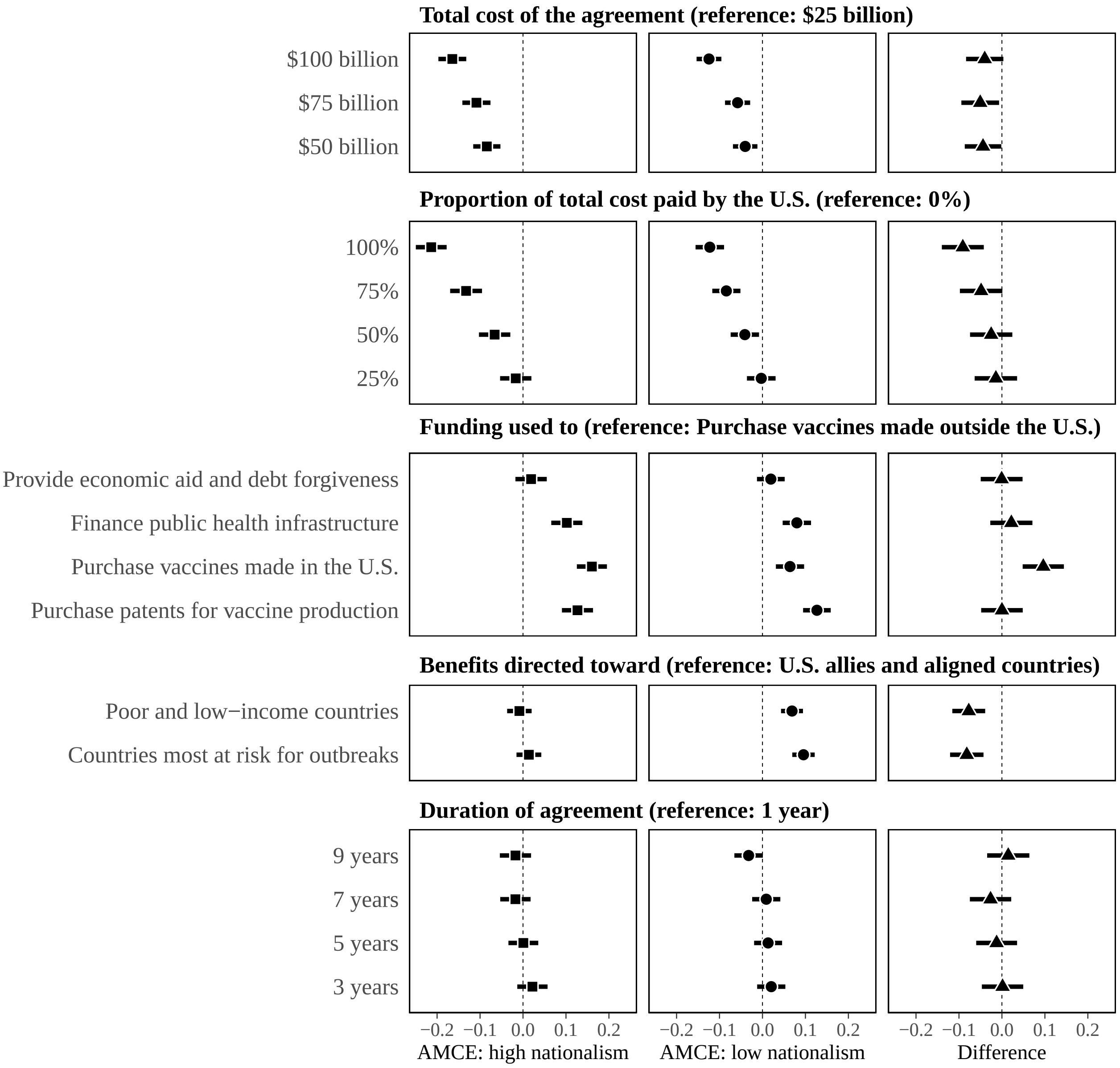

Notes: Sub-group estimates showing average marginal component effects (AMCEs) a among those high in nationalism, low in nationalism, and the differences. Sub-group classifications are based on scores derived from the nationalism scale described in Section S2.1; nationalism is coded as high if a respondent scored higher than the median respondent. Point estimates and 95% confidence intervals estimated via OLS regression with robust standard errors clustered at respondent level to correct for within-respondent clustering. NORC/Amerispeak survey of U.S. adults fielded in September/October 2021 (N = 4,214 respondents x 2 pairings x 2 agreements per pair = 16,856 observations).

Figure S61: Estimated marginal means in international agreement conjoint (NORC) by patriotism

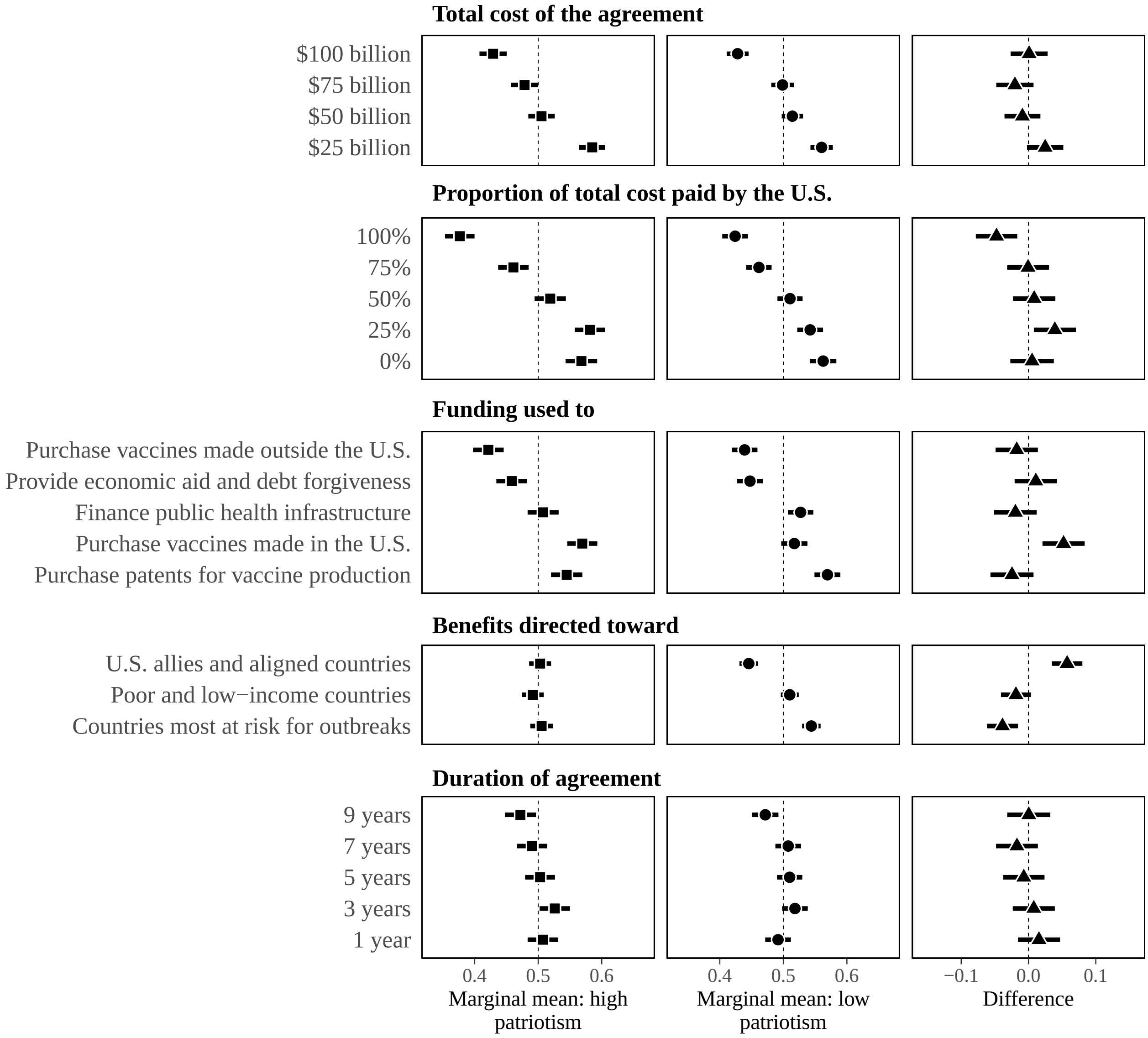

Notes: Sub-group estimates showing marginal means among those high in patriotism, low in patriotism, and the differences. Sub-group classifications are based on scores derived from the patriotism scale described in Section S2.1; patriotism is coded as high if a respondent scored higher than the median respondent. Point estimates and 95% confidence intervals estimated via OLS regression with robust standard errors clustered at respondent level to correct for within-respondent clustering. NORC/Amerispeak survey of U.S. adults fielded in September/October 2021 (N = 4,214 respondents x 2 pairings x 2 agreements per pair = 16,856 observations).

Figure S62: Estimated AMCEs in international agreement conjoint (NORC) by patriotism

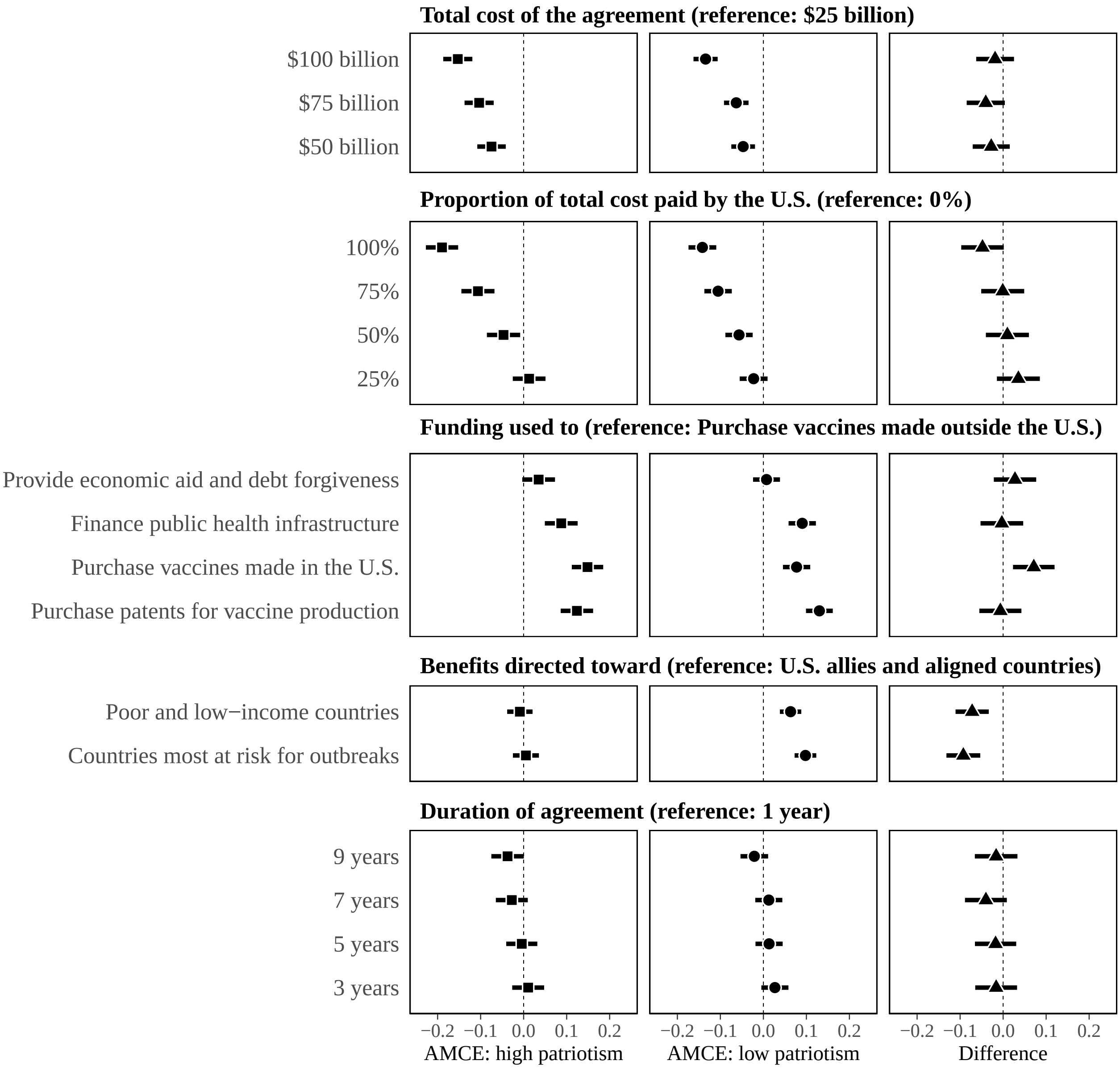

Notes: Sub-group estimates showing average marginal component effects (AMCEs) a among those high in patriotism, low in patriotism, and the differences. Sub-group classifications are based on scores derived from the patriotism scale described in Section S2.1; patriotism is coded as high if a respondent scored higher than the median respondent. Point estimates and 95% confidence intervals estimated via OLS regression with robust standard errors clustered at respondent level to correct for within-respondent clustering. NORC/Amerispeak survey of U.S. adults fielded in September/October 2021 (N = 4,214 respondents x 2 pairings x 2 agreements per pair = 16,856 observations).

Figure S63: Estimated marginal means in international agreement conjoint (NORC) by altruism

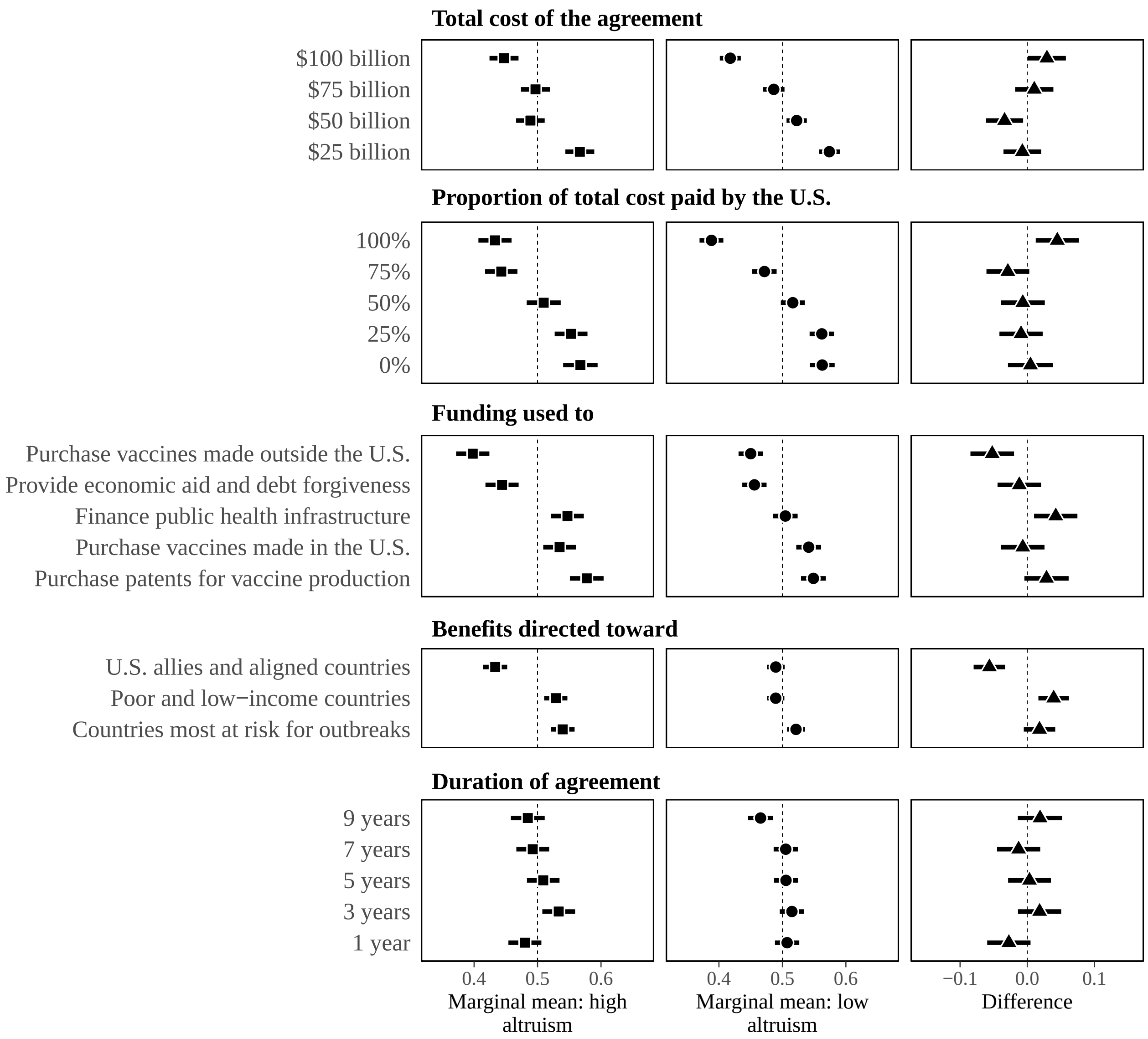

Notes: Sub-group estimates showing marginal means among those high in altruism, low in altruism, and the differences. Sub-group classifications are based on scores derived from the altruism scale described in Section S2.1; altruism is coded as high if a respondent scored higher than the median respondent. Point estimates and 95% confidence intervals estimated via OLS regression with robust standard errors clustered at respondent level to correct for within-respondent clustering. NORC/Amerispeak survey of U.S. adults fielded in September/October 2021 (N = 4,214 respondents x 2 pairings x 2 agreements per pair = 16,856 observations).

Figure S64: Estimated AMCEs in international agreement conjoint (NORC) by altruism

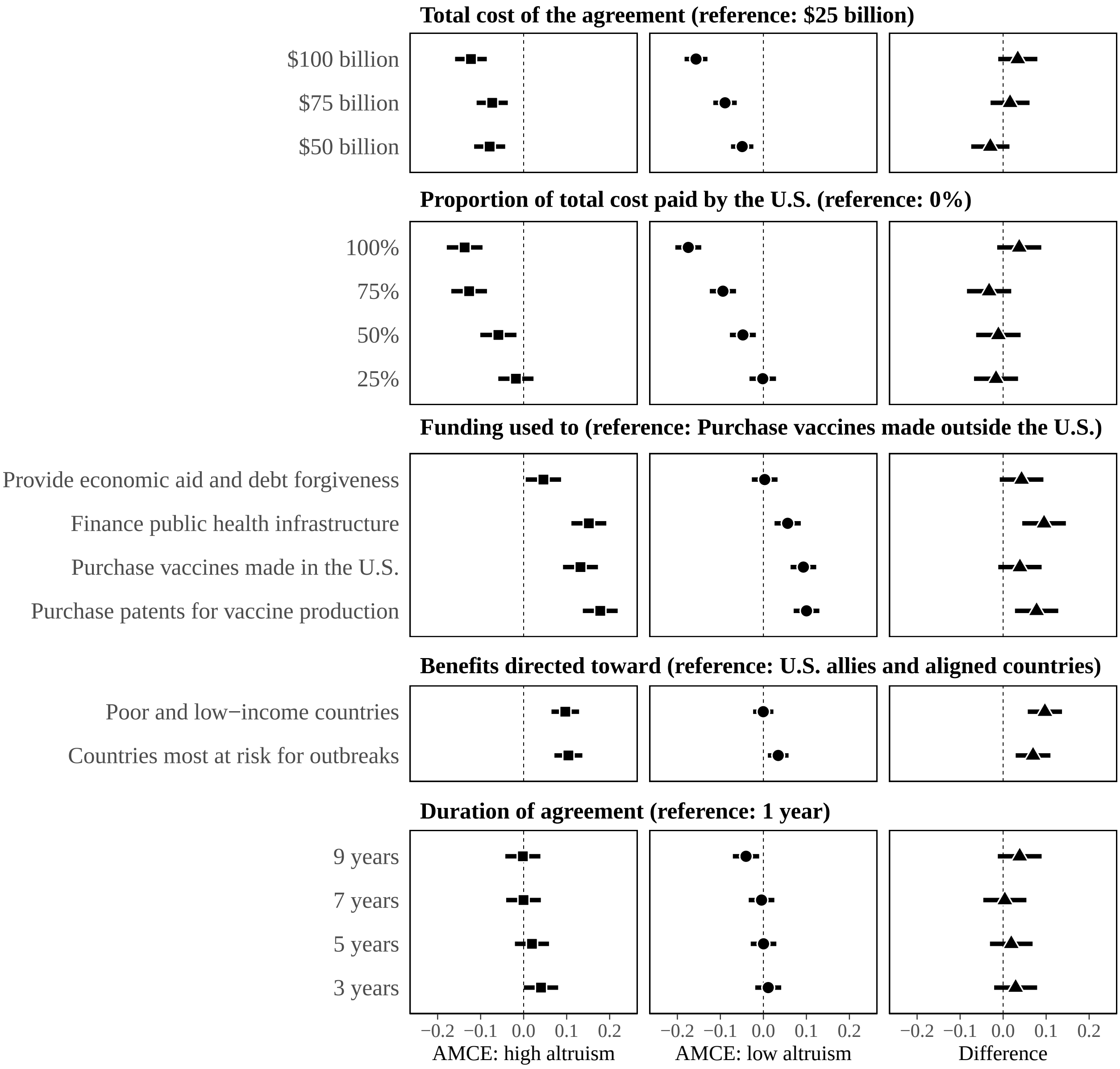

Notes: Sub-group estimates showing average marginal component effects (AMCEs) among those high in altruism, low in altruism, and the differences. Sub-group classifications are based on scores derived from the altruism scale described in Section S2.1; altruism is coded as high if a respondent scored higher than the median respondent. Point estimates and 95% confidence intervals estimated via OLS regression with robust standard errors clustered at respondent level to correct for within-respondent clustering. NORC/Amerispeak survey of U.S. adults fielded in September/October 2021 (N = 4,214 respondents x 2 pairings x 2 agreements per pair = 16,856 observations)

Figure S65: Estimated marginal means in international agreement conjoint (NORC) by partisanship

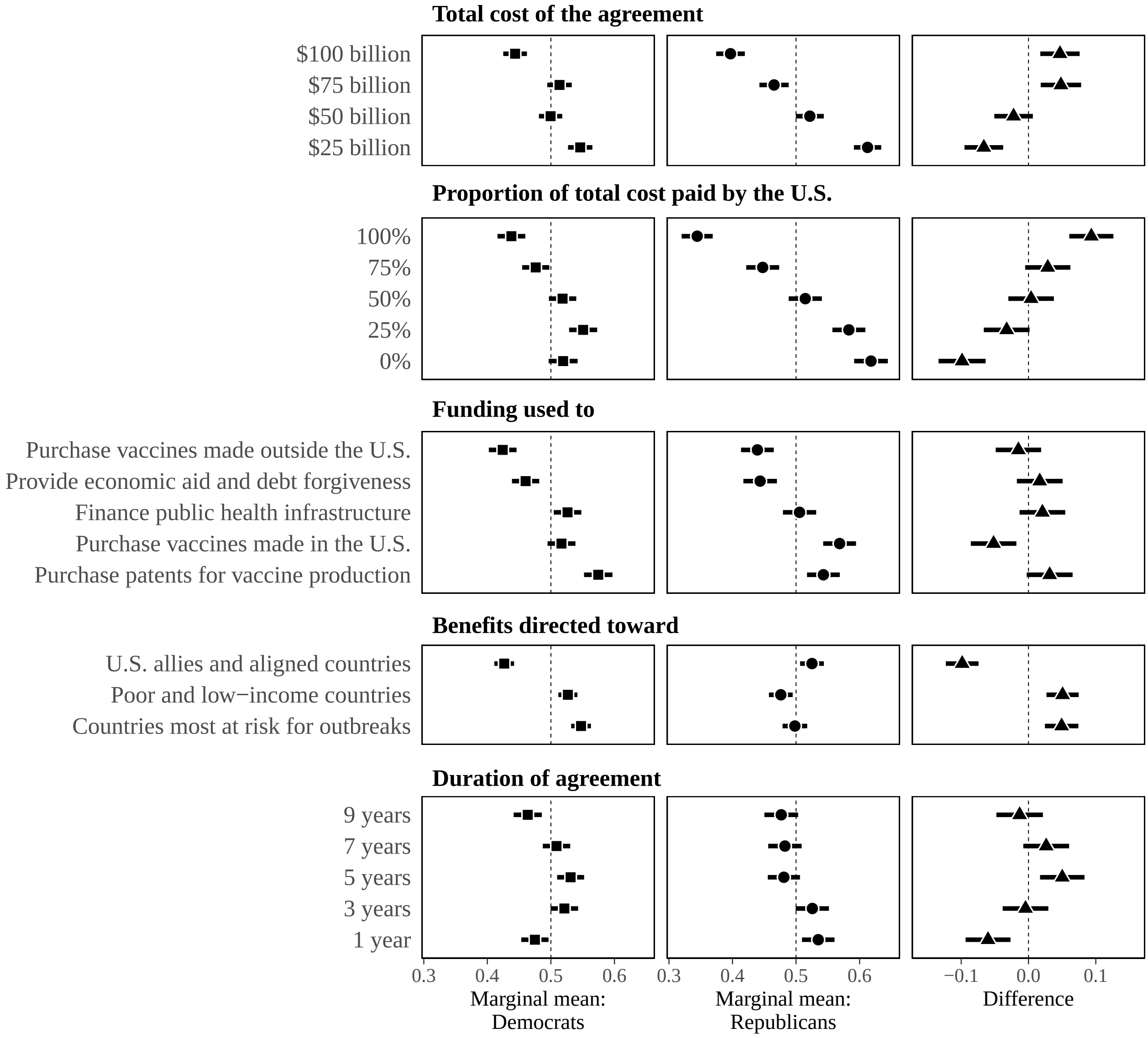

Notes: Sub-group estimates showing marginal means among Democrats, Republicans, and the differences. Partisanship is measured with the standard 7-point branching question used in the American National Election Studies (ANES) Survey. Therefore, Democrat and Republican sub-groups include weak supporters, strong supporters, and those that lean toward one party or another, whereas Independents do not lean one way or the other and are excluded. Point estimates and 95% confidence intervals estimated via OLS regression with robust standard errors clustered at respondent level to correct for within-respondent clustering. NORC/Amerispeak survey of U.S. adults fielded in September/October 2021 (N = 4,214 respondents x 2 pairings x 2 agreements per pair = 16,856 observations)

Figure S66: Estimated AMCEs in international agreement conjoint (NORC) by partisanship

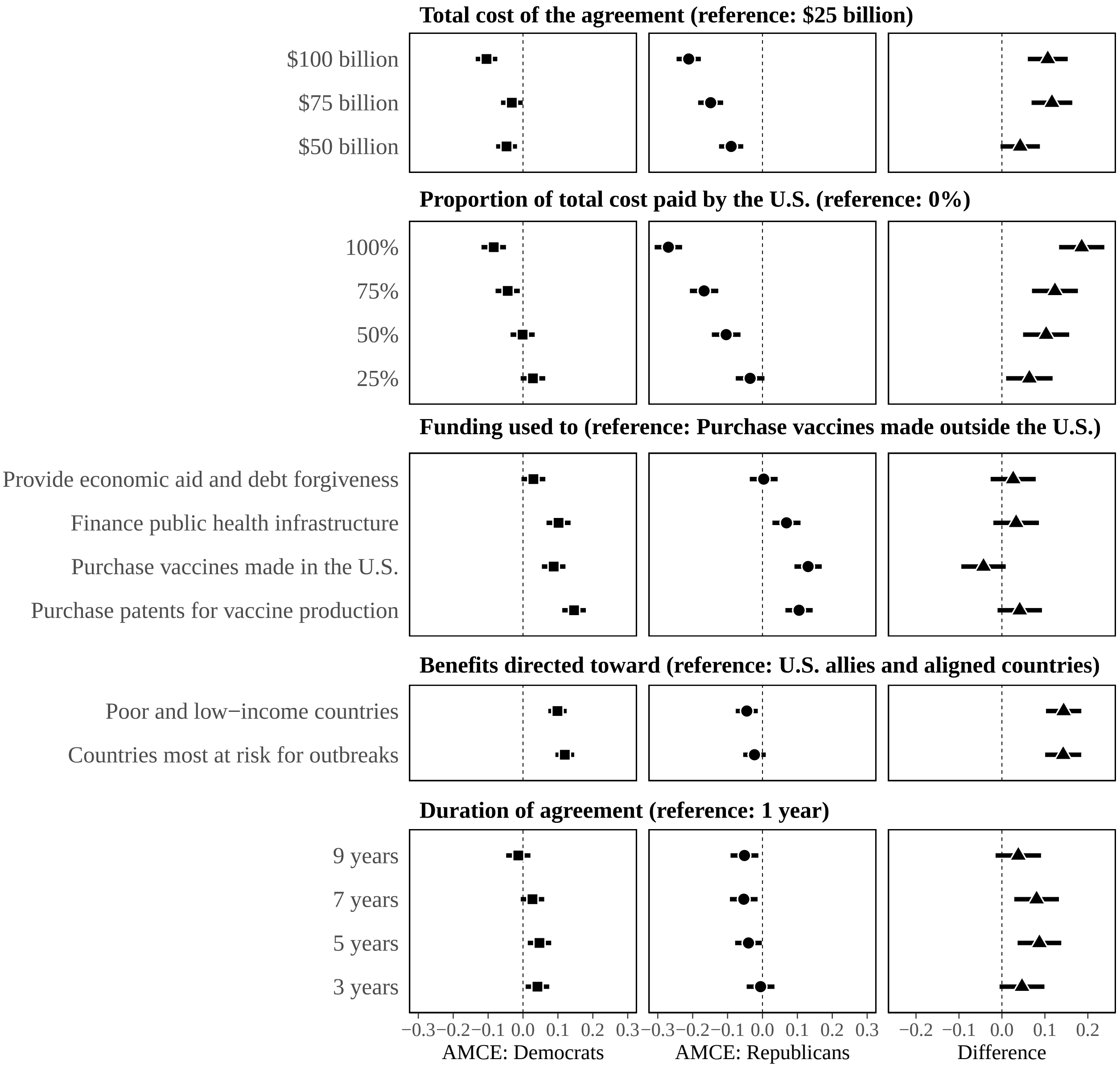

Notes: Sub-group estimates showing average marginal component effects (AMCEs) among Democrats, Republicans, and the differences. Partisanship is measured with the standard 7-point branching question used in the American National Election Studies (ANES) Survey. Therefore, Democrat and Republican sub-groups include weak supporters, strong supporters, and those that lean toward one party or another, whereas Independents do not lean one way or the other and are excluded. Point estimates and 95% confidence intervals estimated via OLS regression with robust standard errors clustered at respondent level to correct for within-respondent clustering. NORC/Amerispeak survey of U.S. adults fielded in September/October 2021 (N = 4,214 respondents x 2 pairings x 2 agreements per pair = 16,856 observations)

Figure S67: Estimated marginal means in international agreement conjoint (NORC) by political ideology

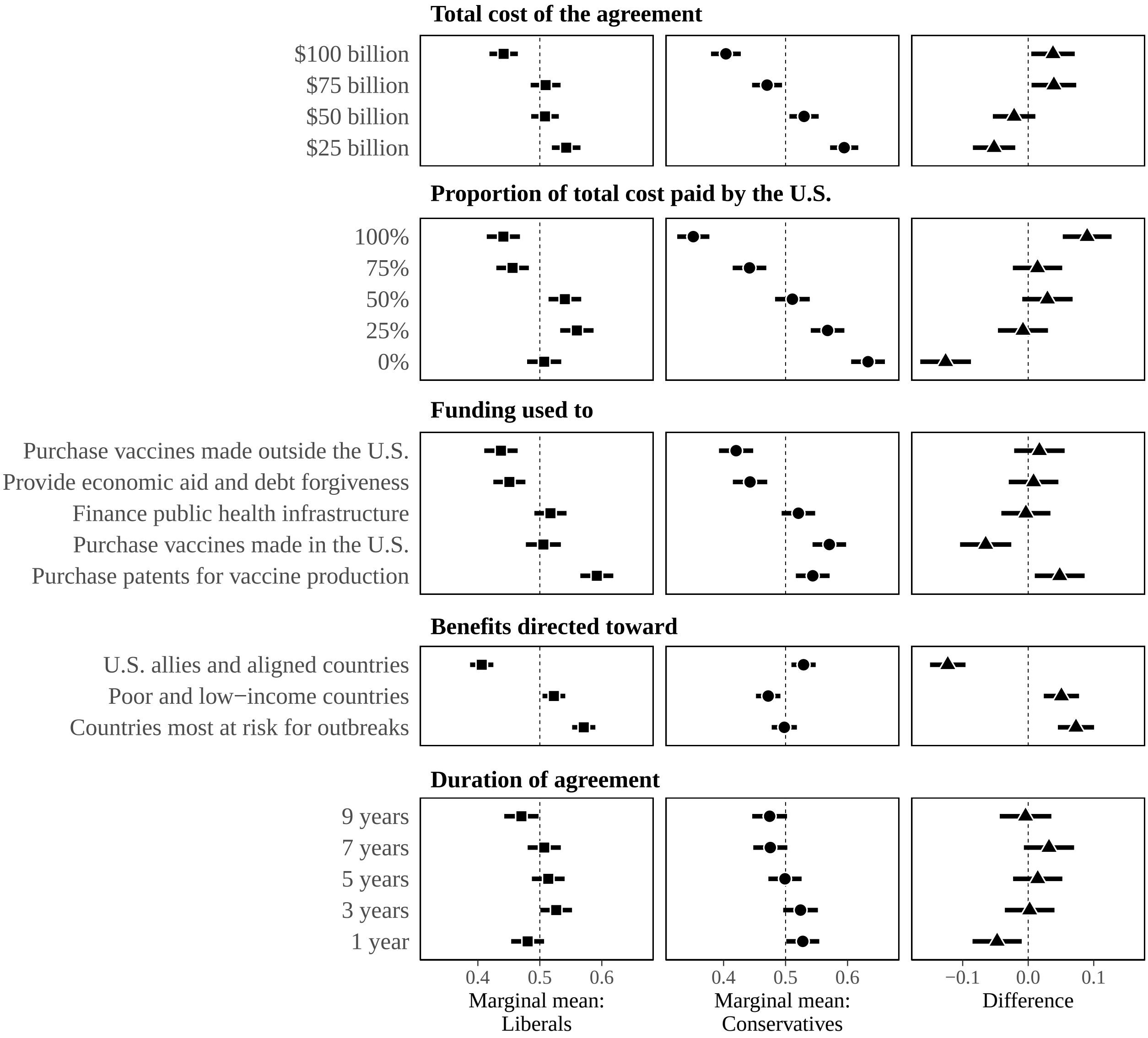

Notes: Sub-group estimates showing marginal means among Liberals, Conservatives, and the differences. Ideology is measured with the standard 7-point scale from "Extremely Liberal" to "Extremely Conservative", and moderates (those at the midpoint of the scale) are excluded. Point estimates and 95% confidence intervals estimated via OLS regression with robust standard errors clustered at respondent level to correct for within-respondent clustering. NORC/Amerispeak survey of U.S. adults fielded in September/October 2021 (N = 4,214 respondents x 2 pairings x 2 agreements per pair = 16,856 observations)

Figure S68: Estimated AMCEs in international agreement conjoint (NORC) by political ideology

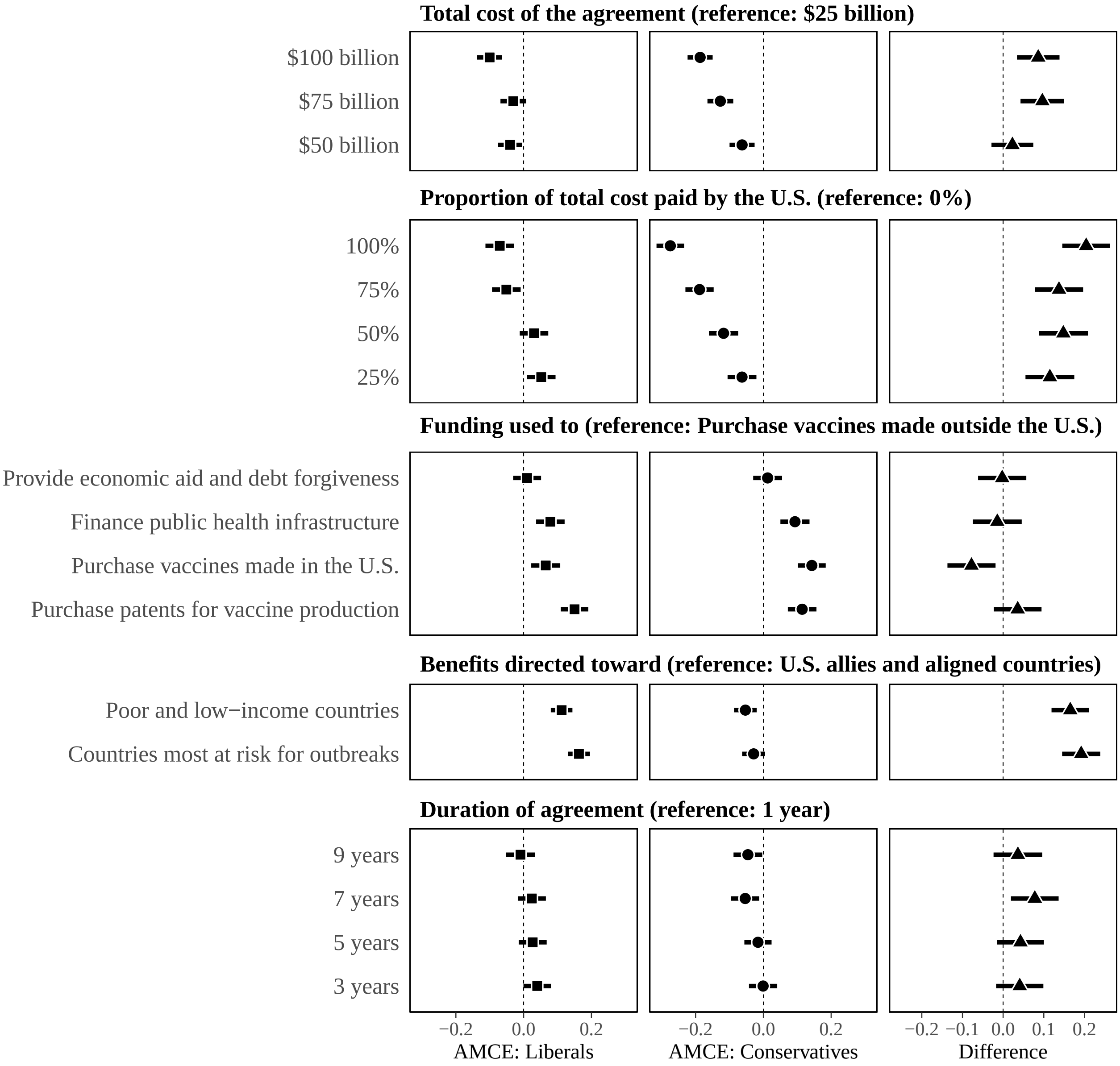

Notes: Sub-group estimates showing average marginal component effects (AMCEs) among Liberals, Conservatives, and the differences. Ideology is measured with the standard 7-point scale from “Extremely Liberal” to “Extremely Conservative”, and moderates (those at the midpoint of the scale) are excluded. Point estimates and 95% confidence intervals estimated via OLS regression with robust standard errors clustered at respondent level to correct for within-respondent clustering. NORC/Amerispeak survey of U.S. adults fielded in September/October 2021 (N = 4,214 respondents x 2 pairings x 2 agreements per pair = 16,856 observations)

S3.8 Effect heterogeneity in persuasion experiment by respondents' background characteristics

We conducted exploratory analyses to investigate treatment effect heterogeneity by background covariates (and the survey items enumerated in Section S2.1) in the persuasion experiment using Generalized Random Forests (GRF), a machine learning algorithm that automates the search for treatment-covariate interactions [21]. GRF estimates heterogeneity as a function of subject-level covariates, predicting individual level treatment effects for all subjects. We apply this approach to the index of all three primary outcomes (as presented in the manuscript). Following graphical presentations in prior work [22, 23], Figure S69 plots predicted treatment effects estimated as a function of individuals' covariate profiles, along with 95% CIs, to provide an overall summary. According to this analysis, 34% of predictions were positive and statistically distinguishable from zero for the Economic Benefits treatment, 14% for Mutation Risk, 17% for Global Inequality, 7% for Vaccine Diplomacy, and 4% for Past Success. Figures S70-S75 plot these predicted treatment effects (jittered and without CIs to avoid over-plotting) as a function of partisanship, ideology, altruism, nationalism, and patriotism. Overall, these analyses suggests a remarkable degree of causal effect homogeneity across subjects.

Figure S69: Causal forest estimated treatment effects in persuasion experiment

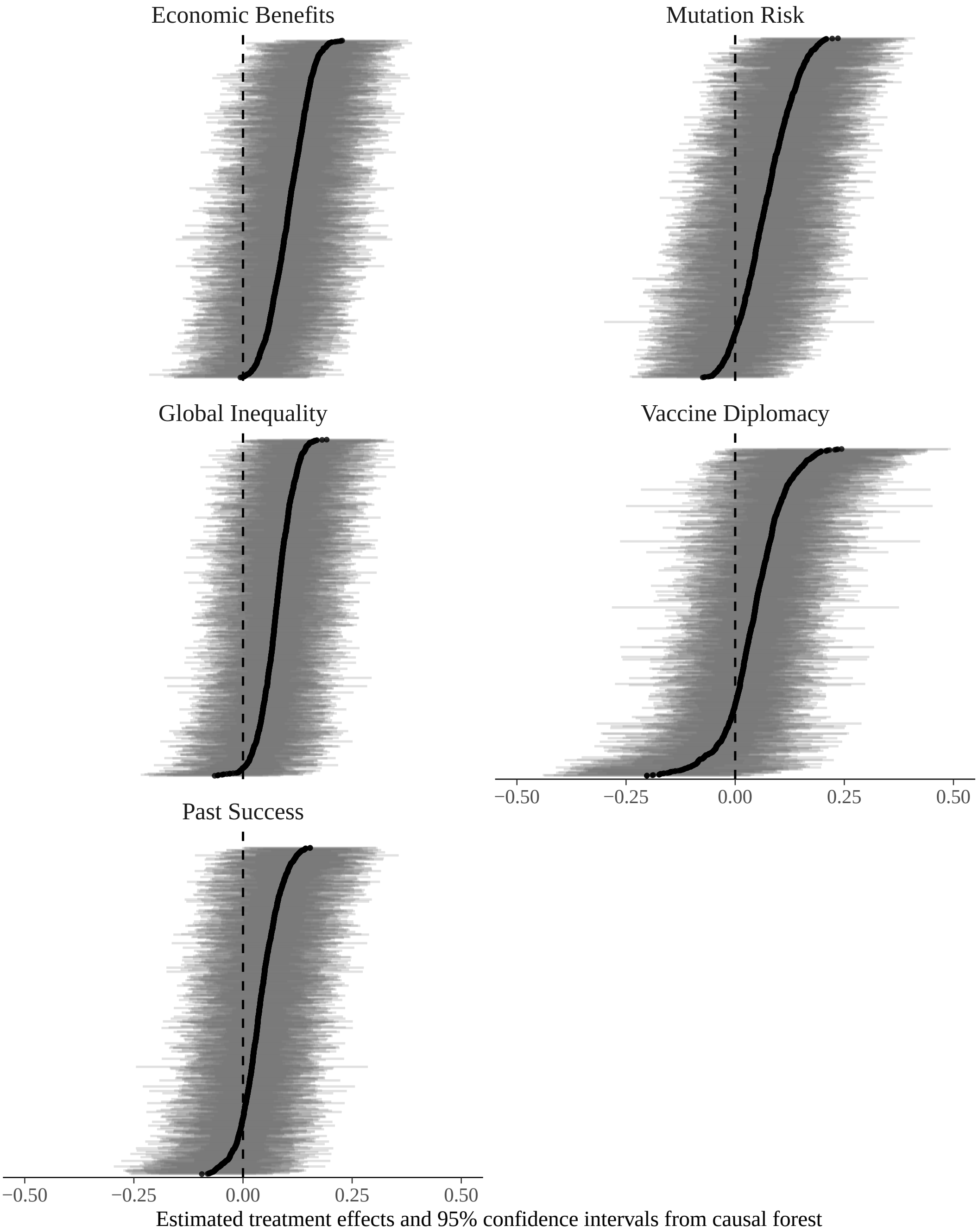

Notes: Estimated treatment effects and 95% confidence intervals from causal forest. Black dots indicate estimated treatment effects for each individual as a function of their covariate profile, ordered by effect size. Gray horizontal lines indicate 95% CIs. Covariates: age, sex, education level, race/ethnicity, region, employment status, household income, partisanship, conservatism, altruism, vaccine nationalism, and nationalism/patriotism.

Figure S70: Causal forest estimated treatment effects in persuasion experiment by partisanship

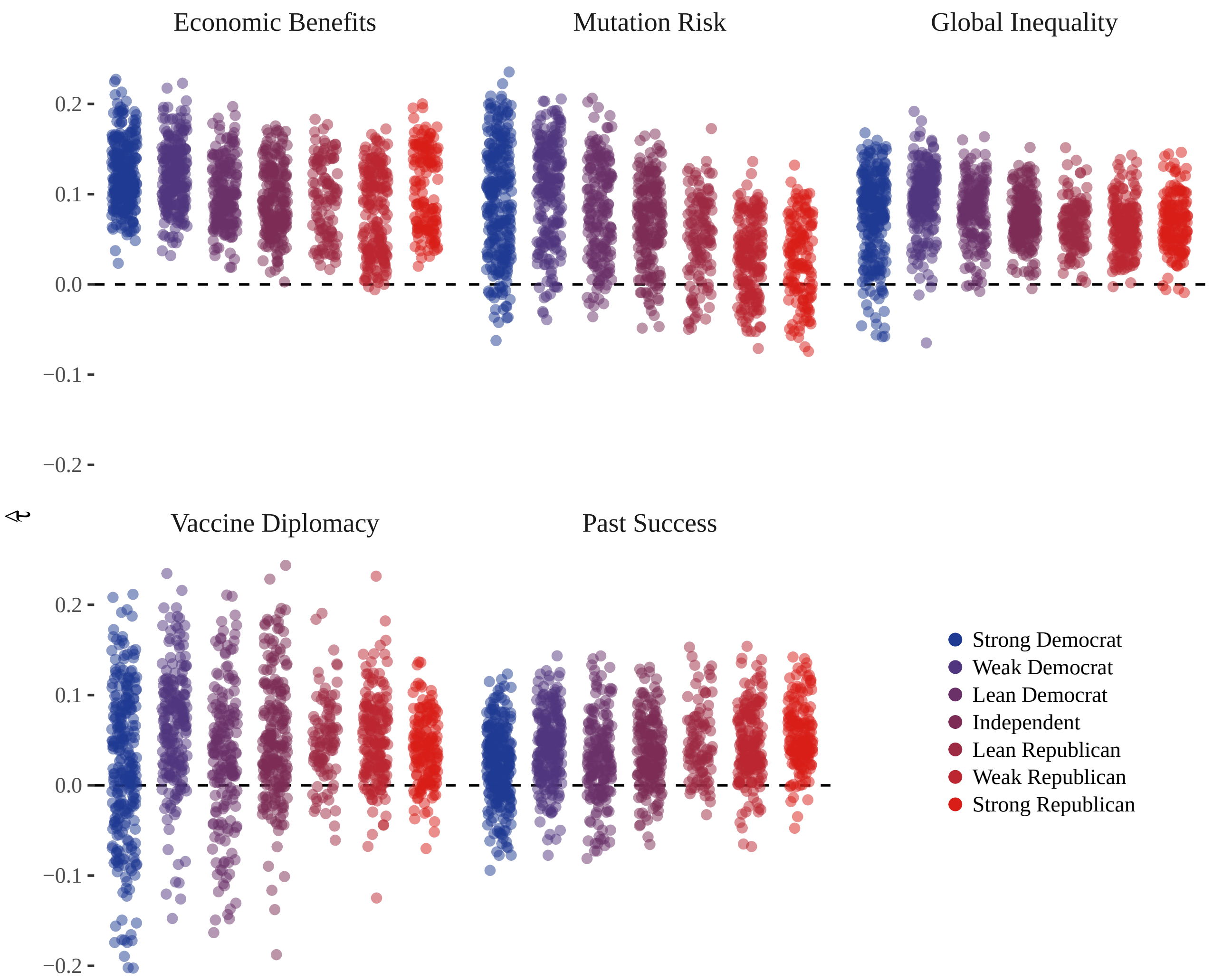

Figure S71: Causal forest estimated treatment effects in persuasion experiment by political ideology

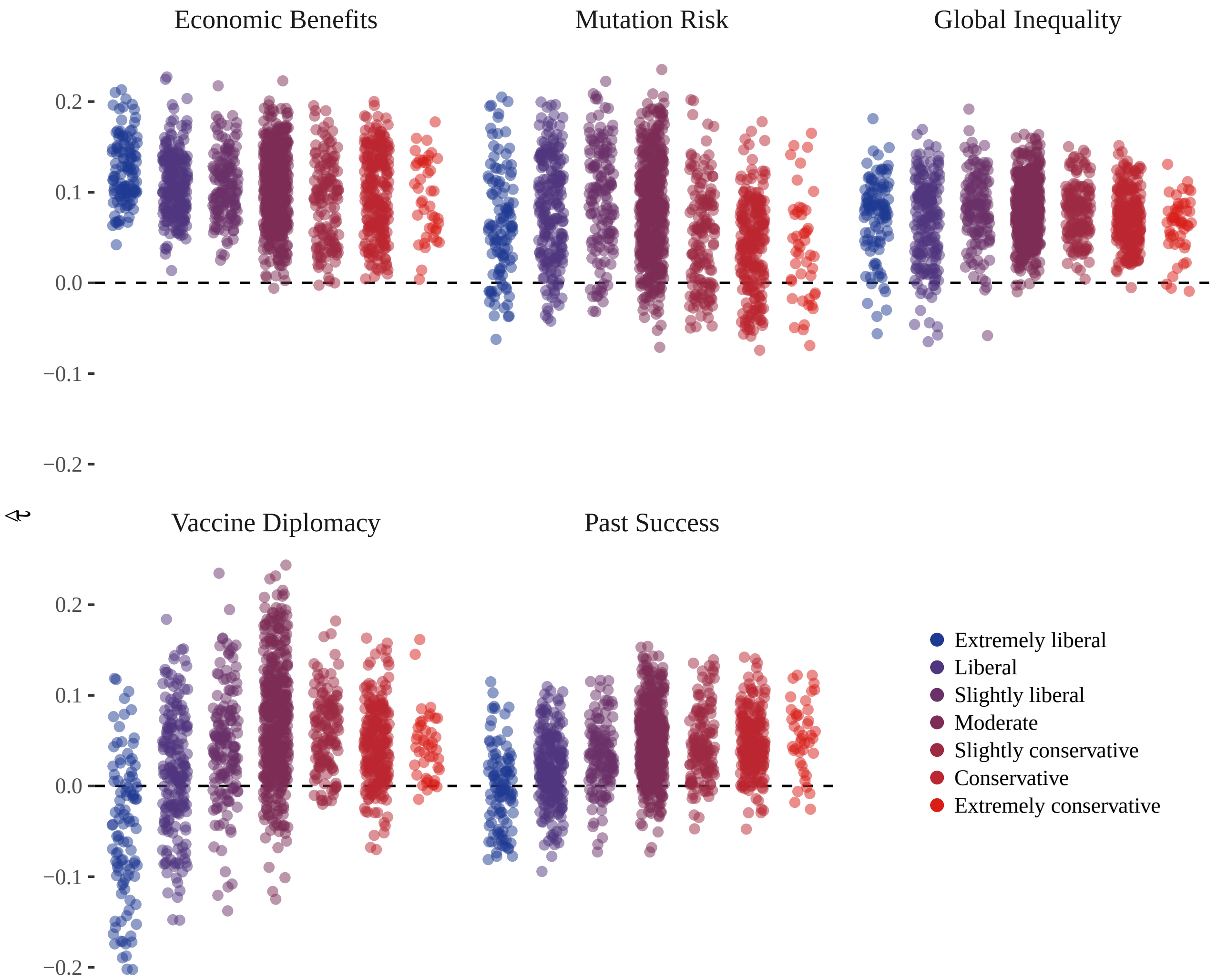

Figure S72: Causal forest estimated treatment effects in persuasion experiment by altruism

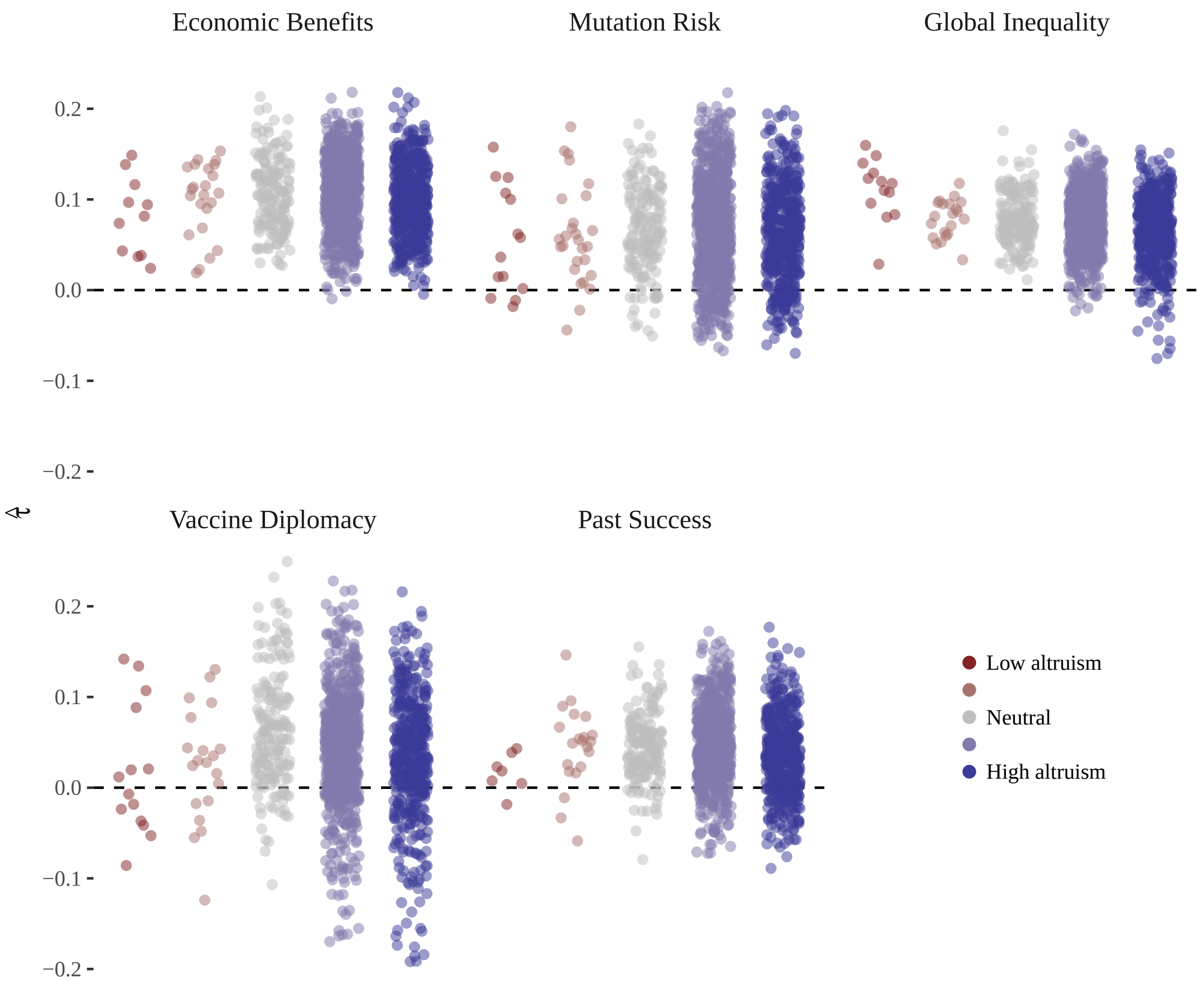

Figure S73: Causal forest estimated treatment effects in persuasion experiment by vaccine nationalism

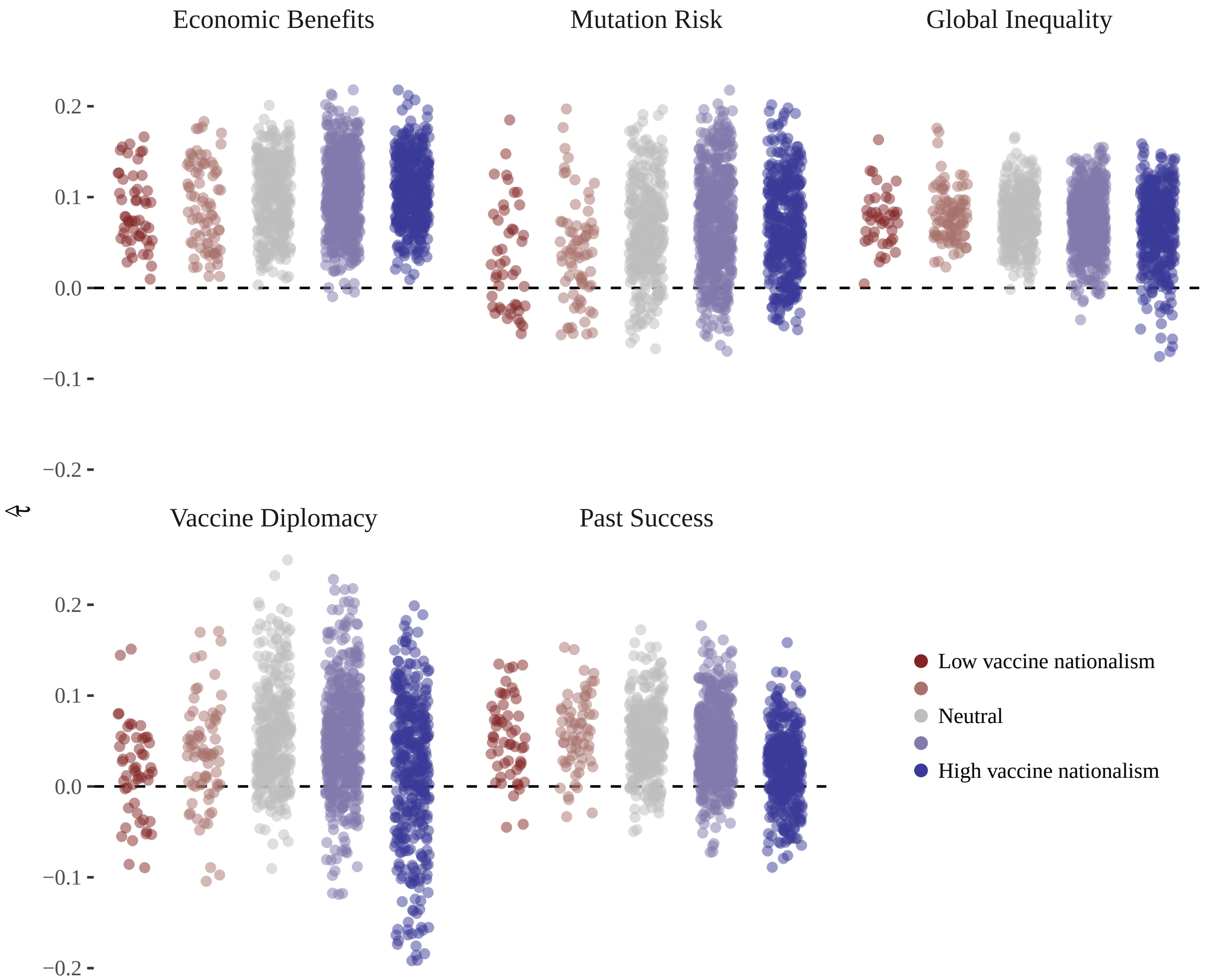

Figure S74: Causal forest estimated treatment effects in persuasion experiment by nationalism

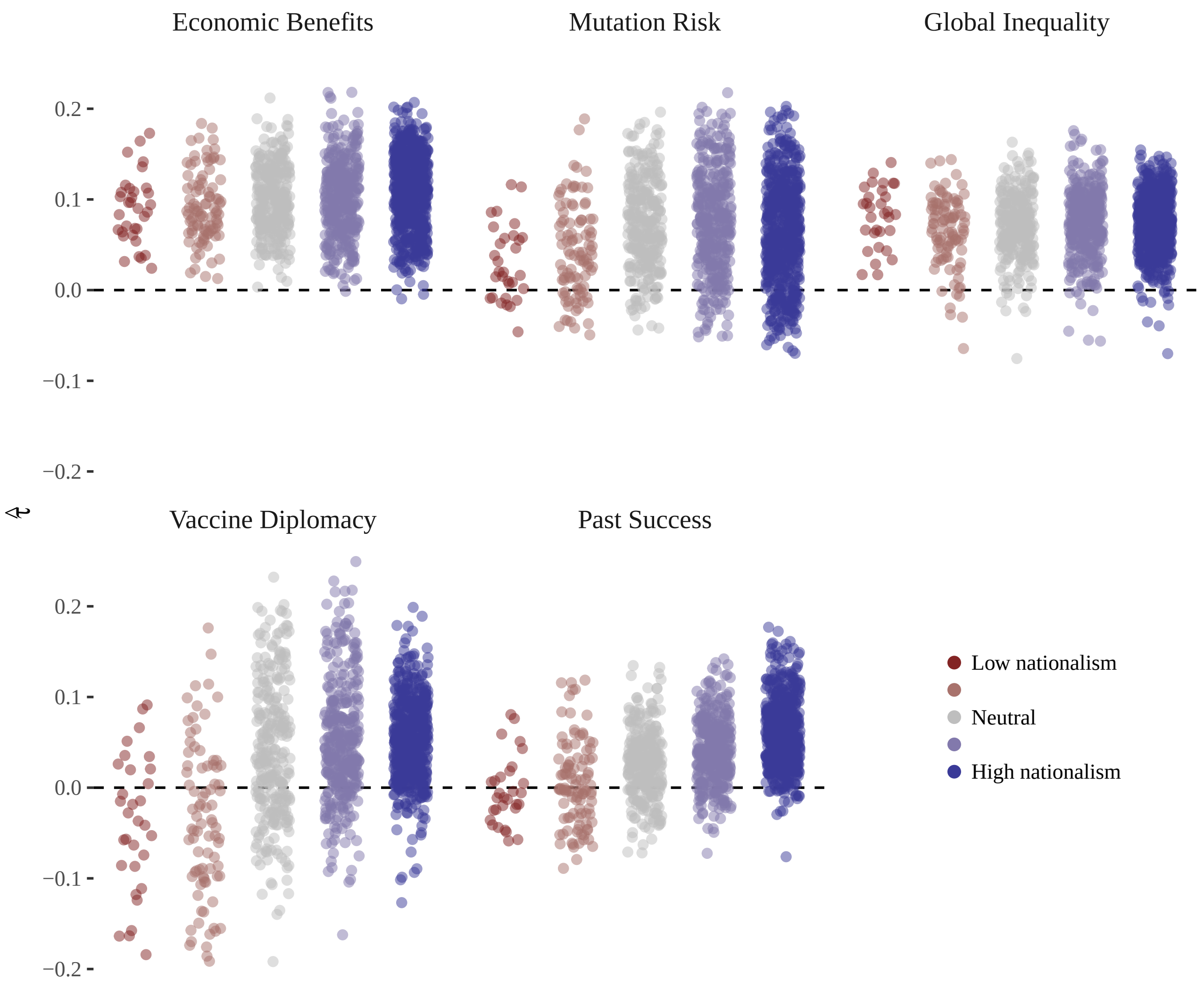

Figure S75: Causal forest estimated treatment effects in persuasion experiment by patriotism

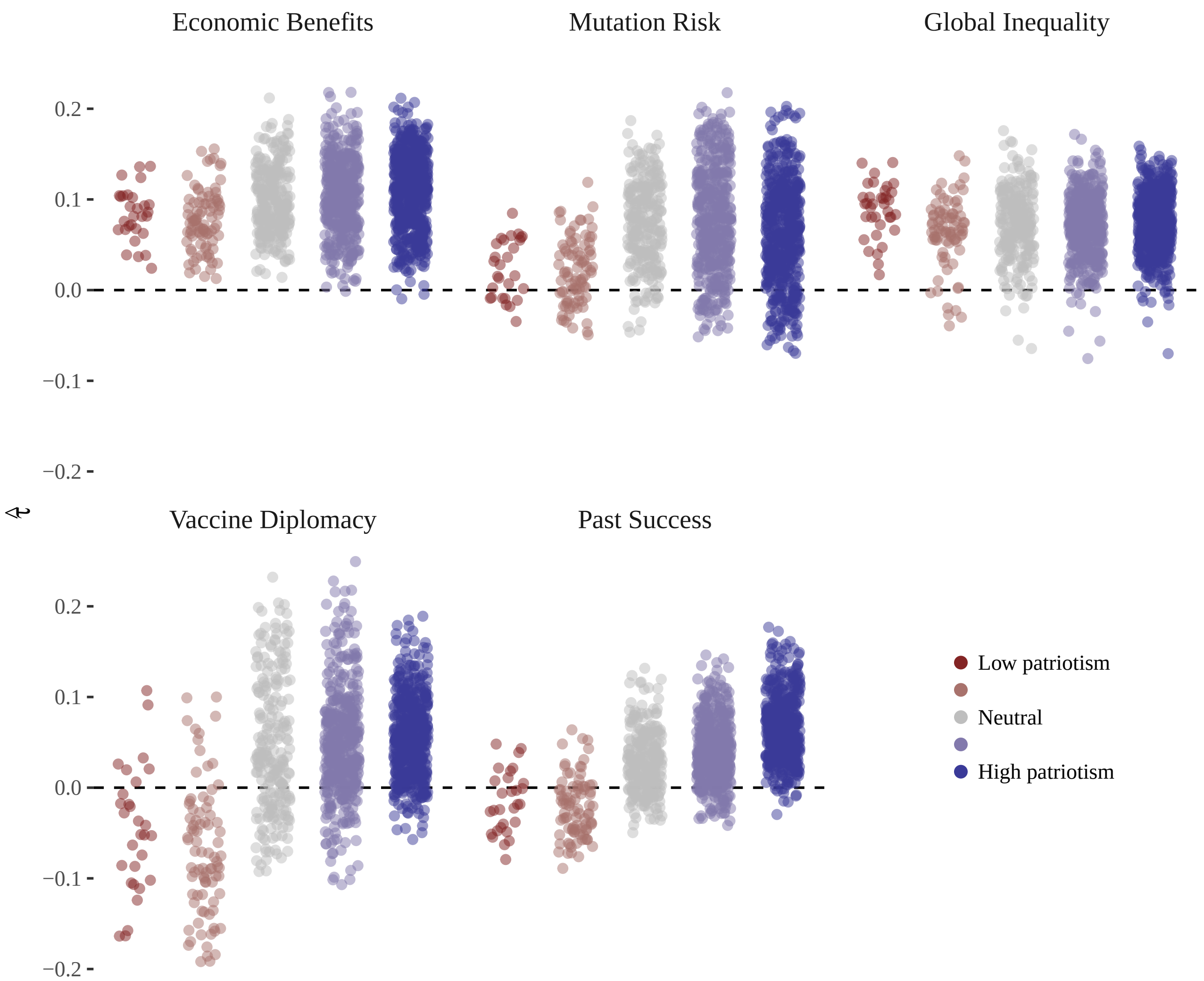

References

[1] Peyton K, Huber GA, Coppock A (2021) The generalizability of online experiments conducted during the covid-19 pandemic. *Journal of Experimental Political Science* p. 1–16.

[2] Coppock A, McClellan OA (2019) Validating the demographic, political, psychological, and experimental results obtained from a new source of online survey respondents. *Research & Politics* 6(1):2053168018822174.

[3] Aronow PM, Kalla J, Orr L, Ternovski J (2020) Evidence of rising rates of inattentiveness on lucid in 2020. *SocArXiv.*

[4] Rudkin A (2020) autumn: Fast, modern, and tidy-friendly iterative raking. *R package version 0.1.*

[5] DeBell M, Krosnick JA (2009) Computing weights for american national election study survey data. *ANES Technical Report Series: nes012427.*

[6] Anderson ML (2008) Multiple inference and gender differences in the effects of early intervention: A reevaluation of the abecedarian, perry preschool, and early training projects. *Journal of the American statistical Association* 103(484):1481–1495.

[7] Funk C, Tyson A (2021) Growing share of americans say they plan to get a covid-19 vaccine–or already have. *Pew Research Center Science & Society.[(accessed on 2 April 2021)].*

[8] Thorsett L, Kiley J (2017) Most americans say the u.s. is among the greatest countries in the world. *Pew Research Center Science & Society.[(accessed on 2 April 2021)].*

[9] Hartig H, Gilberstadt H (2019) Younger americans more likely than older adults to say there are other countries that are better than the u.s. *Pew Research Center Science & Society.[(accessed on 2 April 2021)].*

[10] Bechtel MM, Hainmueller J, Margalit Y (2014) Preferences for international redistribution: The divide over the eurozone bailouts. *American Journal of Political Science* 58(4):835–856.

[11] Dye TR (1963) The local-cosmopolitan dimension and the study of urban politics. *Social Forces* 41(3):239–246.

[12] Merton RK, Merton RC (1968) *Social theory and social structure.* (Simon and Schuster).

[13] Falk A, Becker A, Dohmen TJ, Huffman D, Sunde U (forthcoming) The preference survey module: A validated instrument for measuring risk, time, and social preferences. *Management Science.*

[14] Blair G, Coppock A, Moor M (2020) When to worry about sensitivity bias: A social reference theory and evidence from 30 years of list experiments. *American Political Science Review* 114(4):1297–1315.

[15] Bischoff I, Frank B, , et al. (2011) Good news for experimenters: Subjects are hard to influence by instructors' cues. *Economics Bulletin* 31(4):3221–3225.

[16] White A, Strezhnev A, Lucas C, Kruszewska D, Huff C (2018) Investigator characteristics and respondent behavior in online surveys. *Journal of Experimental Political Science* 5(1):56–67.

[17] De Quidt J, Haushofer J, Roth C (2018) Measuring and bounding experimenter demand. *American Economic Review* 108(11):3266–3302.

[18] De Quidt J, Vesterlund L, Wilson AJ (2019) Experimenter demand effects in *Handbook of research methods and applications in experimental economics.* (Edward Elgar Publishing).

[19] Mummolo J, Peterson E (2019) Demand effects in survey experiments: An empirical assessment. *American Political Science Review* 113(2):517–529.

[20] Coppock A (2019) ri2: Randomization inference for randomized experiments. *R package version 0.1* 2.

[21] Athey S, Tibshirani J, Wager S (2019) Generalized random forests. *The Annals of Statistics* 47(2):1148–1178.

[22] Guess A, Coppock A (2020) Does counter-attitudinal information cause backlash? results from three large survey experiments. *British Journal of Political Science* 50(4):1497–1515.

[23] Peyton K (2020) Does trust in government increase support for redistribution? evidence from randomized survey experiments. *American Political Science Review* 114(2):596–602.

S4 Pre-registration for vaccine recipient conjoint in Lucid survey

ASPREDICTED

Wharton CREDIBILITY LAB UNIVERSITY of PENNSYLVANIA

CONFIDENTIAL - FOR PEER-REVIEW ONLY

American vaccine conjoint for individuals (#63202)

Created: 04/12/2021 07:03 AM (PT)

This is an anonymized copy (without author names) of the pre-registration. It was created by the author(s) to use during peer-review.
A non-anonymized version (containing author names) should be made available by the authors when the work it supports is made public.

1) Have any data been collected for this study already?
No, no data have been collected for this study yet.

2) What's the main question being asked or hypothesis being tested in this study?
This study uses a conjoint experiment to quantify how the attributes enumerated below affect Americans' preferences for distributing COVID-19 vaccines between individuals. The primary question of interest is whether, all else equal, Americans prioritize vaccinating U.S. residents over individuals in need from other countries.

3) Describe the key dependent variable(s) specifying how they will be measured.
Respondents will choose between two hypothetical persons to receive a vaccine. The primary outcome is a binary choice between the persons presented during each conjoint task: "If you had to choose between them, which of these two people should be given priority to receive the vaccine?". The secondary outcome is a 7-point scale: "Please rate each person on a scale from 1 to 7, where 1 indicates they should definitely not receive the vaccine and 7 indicates they should definitely receive the vaccine."

4) How many and which conditions will participants be assigned to?
Each respondent will evaluate five conjoint tasks with seven attributes. The attributes (and levels): 1) Sex ("Male", "Female"); 2) Age group ("18-24","25-34","35-44","45-54","55-64","65-74","75+"); 3) Risk of serious illness from COVID-19 ("Low","Moderate","High"); 4) Risk of exposure to COVID-19 ("Low","Moderate","High"); 5) Country of origin ("United States","Canada","Brazil","Nigeria","India","Pakistan","China","South Africa","Australia"); 6) Occupation group ("Non-essential worker","Healthcare workers","First responders (e.g., police/fire)","Education and childcare","Public transit"); 7) Can work from home? ("Yes", "No").

5) Specify exactly which analyses you will conduct to examine the main question/hypothesis.
The estimands of interest in this conjoint are the AMCEs. We will use linear regression on the randomized attributes, with robust standard errors clustered at the respondent level. We will also report the marginal means for the levels within each attribute.

6) Describe exactly how outliers will be defined and handled, and your precise rule(s) for excluding observations.
Not applicable.

7) How many observations will be collected or what will determine sample size? No need to justify decision, but be precise about exactly how the number will be determined.
1500 participants. Determined from simulation studies that showed our estimation procedure would detect effects of 5 percentage points with at least 0.80 power (fixing alpha = 0.05) at this sample size.

8) Anything else you would like to pre-register? (e.g., secondary analyses, variables collected for exploratory purposes, unusual analyses planned?)
We will conduct secondary analyses to examine effect heterogeneity across sub-groups of respondents along the following dimensions: 1) nationalism/cosmopolitanism; 2) altruism; 3) reciprocity; 4) vaccination status of respondent at time of survey. Nationalism/cosmopolitanism, altruism and reciprocity scores will be based on existing scales that have been behaviorally validated in prior studies.

IMPORTANT
This pre-registration is part of a set of similar and related pre-registrations sharing at least one author. When one of these pre-registrations was shared by an author, the rest were shared automatically. Links to all of them, including this one, appear below:

https://aspredicted.org/PVH_P8V
https://aspredicted.org/3GC_L3K

Version of AsPredicted Questions: 2.00

Available at https://aspredicted.org/PVH_P8V

S5 Pre-registration for international agreement conjoint in Lucid survey

AS PREDICTED

Wharton CREDIBILITY LAB UNIVERSITY of PENNSYLVANIA

CONFIDENTIAL - FOR PEER-REVIEW ONLY

American vaccine conjoint for international agreements (#63201)

Created: 04/12/2021 07:02 AM (PT)

This is an anonymized copy (without author names) of the pre-registration. It was created by the author(s) to use during peer-review.
A non-anonymized version (containing author names) should be made available by the authors when the work it supports is made public.

---

1) Have any data been collected for this study already?
No, no data have been collected for this study yet.

2) What's the main question being asked or hypothesis being tested in this study?
This study uses a conjoint experiment to quantify how the attributes enumerated below affect Americans' preferences for international agreements to supply COVID-19 vaccines to countries in need. The primary question of interest is whether, all else equal, the cost of implementing such agreements outweighs institutional design features.

3) Describe the key dependent variable(s) specifying how they will be measured.
Respondents will choose between two hypothetical agreements to supply COVID-19 vaccines internationally. The primary outcome is a binary choice between the persons presented during each conjoint task: "Which of these two agreements do you prefer?" The secondary outcome is a 7-point scale: "If you could vote on each of these agreements, how likely is it that you would vote in favor or against each of the agreements? Please give your answer on the following scale from definitely against (1) to definitely in favor (7)."

4) How many and which conditions will participants be assigned to?
Each respondent will evaluate four conjoint tasks with seven attributes. The attributes (and levels): 1) Cost to average household ("$1","$5","$10","$15","$20"); 2) Number of participating countries ("20 of 192","80 of 192","100 of 192","170 of 192"); 3) Distribution of costs ("Only rich countries contribute","Rich countries contribute more than poor countries", "Countries that need more vaccines pay more"); 4) Distribution of benefits ("Only poor countries","Poor countries get more than rich countries","Proportional to a country's population size","Countries that pay more get more in return","Countries with more people at risk of severe illness get more"); 5) External supply agreements allowed? ("Yes", "No"); 6) Sharing of vaccine technology ("Compulsory", "Voluntary"); 7) Monitoring ("U.S. government","World Health Organization","Independent commission","United Nations")

5) Specify exactly which analyses you will conduct to examine the main question/hypothesis.
The estimands of interest in this conjoint are the AMCEs. We will use linear regression on the randomized attributes, with robust standard errors clustered at the respondent level. We will also report the marginal means for the levels within each attribute.

6) Describe exactly how outliers will be defined and handled, and your precise rule(s) for excluding observations.
Not applicable.

7) How many observations will be collected or what will determine sample size? No need to justify decision, but be precise about exactly how the number will be determined.
1500 participants. Determined from simulation studies that showed our estimation procedure would detect effects of 5 percentage points with at least 0.80 power (fixing alpha = 0.05) at this sample size.

8) Anything else you would like to pre-register? (e.g., secondary analyses, variables collected for exploratory purposes, unusual analyses planned?)
We will conduct secondary analyses to examine effect heterogeneity across sub-groups of respondents along the following dimensions: 1) nationalism/cosmopolitanism; 2) altruism; 3) reciprocity; 4) vaccination status of respondent at time of survey. Nationalism/cosmopolitanism, altruism and reciprocity scores will be based on existing scales that have been behaviorally validated in prior studies.

IMPORTANT
This pre-registration is part of a set of similar and related pre-registrations sharing at least one author. When one of these pre-registrations was shared by an author, the rest were shared automatically. Links to all of them, including this one, appear below:

https://aspredicted.org/PVH_P8V
https://aspredicted.org/3GC_L3K

S6 Pre-registration for international agreement conjoint in NORC survey

AS PREDICTED

Wharton CREDIBILITY LAB UNIVERSITY of PENNSYLVANIA

CONFIDENTIAL - FOR PEER-REVIEW ONLY

NORC/AmeriSpeak COVID conjoint (#74270)

Created: 09/09/2021 07:56 AM (PT)

This is an anonymized copy (without author names) of the pre-registration. It was created by the author(s) to use during peer-review.
A non-anonymized version (containing author names) should be made available by the authors when the work it supports is made public.

---

1) Have any data been collected for this study already?
No, no data have been collected for this study yet.

2) What's the main question being asked or hypothesis being tested in this study?
This study uses a conjoint experiment to quantify how the attributes enumerated below affect Americans' preferences for international agreements to supply COVID-19 vaccines and related assistance to countries in need. The primary question of interest is how, all else equal, the implementation costs and proportion paid by the United States affects program support relative to other institutional design features such as the form of transfer, duration of the program, and method of benefit distribution.

3) Describe the key dependent variable(s) specifying how they will be measured.
Respondents will choose between two hypothetical agreements to supply COVID-19 vaccines internationally. The primary outcome is a binary choice between the persons presented during each conjoint task: "Which of these two agreements do you prefer?" The secondary outcome is a 7-point scale: "If you could vote on each of these agreements, how likely is it that you would vote in favor or against each of the agreements? Please give your answer on the following scale from definitely against (1) to definitely in favor (7)."

4) How many and which conditions will participants be assigned to?
Each respondent will evaluate four conjoint tasks with seven attributes. The attributes (and levels) are: 1. Overall cost: "$100 billion","$75 billion","$50 billion", or "$25 billion"; 2. Proportion of total paid by US: "100%", "75%", "50%", "25%", or "0%"; 3. Funding will be used to: "Purchase vaccines made by companies in the U.S.", "Purchase vaccines made by companies outside the U.S.", "Purchase patents from vaccine companies to make production technology freely available to the world", "Finance health infrastructure and health campaigns in poor countries", or "Provide economic aid and debt forgiveness to COVID-affected countries"; 4. Benefits will be directed to: "U.S. allies and U.S. aligned countries", "Countries most at risk of COVID-19 outbreaks", or "Poor and low-income countries"; 5. Duration of program: "1 year", "3 year", "5 years", "7 years", or "9 years".

5) Specify exactly which analyses you will conduct to examine the main question/hypothesis.
The estimands of interest in this conjoint are the Average Marginal Component Effects (AMCEs). We will use linear regression of the outcome on the randomized attributes, with robust standard errors clustered at the respondent level. We will also report the marginal means for the levels within each attribute.

6) Describe exactly how outliers will be defined and handled, and your precise rule(s) for excluding observations.
Not applicable.

7) How many observations will be collected or what will determine sample size? No need to justify decision, but be precise about exactly how the number will be determined.
The study will be administered as part of a nationally representative survey on the NORC AmeriSpeak panel. This survey consists of approximately 4,000 U.S. adults (aged 18+).

8) Anything else you would like to pre-register? (e.g., secondary analyses, variables collected for exploratory purposes, unusual analyses planned?)
We will conduct secondary analyses to examine effect heterogeneity across sub-groups of respondents along the following dimensions: 1) nationalism/patriotism; 2) partisanship; 3) altruism; 4) ideology. These pre-treatment survey measures are based on existing scales that have been validated in prior studies.

Version of AsPredicted Questions: 2.00

Available at https://aspredicted.org/PQV_PB4

S7 Pre-registration for persuasion experiment in NORC survey

CONFIDENTIAL - FOR PEER-REVIEW ONLY

NORC/AmeriSpeak COVID persuasion experiment (#74271)

Created: 09/09/2021 08:02 AM (PT)

This is an anonymized copy (without author names) of the pre-registration. It was created by the author(s) to use during peer-review.
A non-anonymized version (containing author names) should be made available by the authors when the work it supports is made public.

---

1) Have any data been collected for this study already?

No, no data have been collected for this study yet.

2) What's the main question being asked or hypothesis being tested in this study?

This study uses a randomized experiment with six-arms to quantify how different persuasive messaging strategies affect public support for funding international initiatives to supply COVID-19 vaccines and related aid to countries abroad. The primary question of interest is whether, compared to a control condition with no information, providing different persuasive appeals (as detailed below) affects Americans' support for spending on international vaccination efforts, as measured across various outcomes.

3) Describe the key dependent variable(s) specifying how they will be measured.

There are three outcome measures (enumerated below). We will also compute an index of support that combines all three outcome measures.

1. "Recent studies by economists at the International Monetary Fund suggest that as much as $50 billion is required to quickly vaccinate everyone in all countries. In your view, what percentage of this amount should the US fund?" (0-100%).
2. Whether the respondent supports a real petition calling on Congress to "increase US spending on COVID-19 assistance abroad" (Yes = 1; No = 0).
3. Whether the respondent is willing to donate real money to support international vaccination efforts: "Approximately 100 participants in this survey will be randomly selected for a bonus payment of $10. If selected, you may keep the entire amount or donate a part of the payment to the COVID-19 Vaccines Global Access Initiative (COVAX), an international initiative to vaccinate people in low-income countries. Your choice will not affect your chance of being selected for the bonus. Any amount you choose to donate will be deducted from your bonus payment before it is credited to you. What amount (if any) would you like to donate?" (0-$10).

4) How many and which conditions will participants be assigned to?

There are six treatment arms, with 1/6 of the sample randomly assigned to each:

1. Control: no information.
2. Inequality: "Rich countries have secured most of the world's COVID-19 vaccine supplies. 85% of shots administered worldwide have been in high-and upper-middle-income countries. Less than 1% of doses have been administered in low-income countries, and many in Asia, Africa, and Latin America will not have access to a vaccine for the foreseeable future. The US can help to reduce these inequalities by coordinating a global effort to rapidly vaccinate the world against COVID-19."
3. Past success: "In 2003, the US created the President's Emergency Plan for AIDS Relief (PEFPAR) to share medicines and technical support with partner countries in order to treat HIV/AIDS and other infectious diseases. PEPFAR is widely considered to be one of the most effective public health efforts in history and is estimated to have saved as many as 20 million lives. The US can build on successful past public health campaigns like PEFPAR to coordinate a global effort to rapidly vaccinate the world against COVID-19."
4. Mutation risk: "As COVID-19 continues to spread in countries around the world, new variants are emerging. Some of these variants, such as those found in the United Kingdom, South Africa, and India, can be more contagious or deadly than strains for which vaccines have already been developed. The Delta variant, which first emerged in India, is now dominant in the US. The US can help to prevent the emergence of new strains by coordinating a global effort to rapidly vaccinate the world against COVID-19."
5. Economic benefits: "A recent study found that if the poorest countries cannot access COVID-19 vaccines, the world could lose between $60 billion and $340 billion a year in income. The researchers concluded that for every $1 spent on supplying poorer countries with vaccines, high-income countries would get back about $4.80 through greater exports and other mechanisms. The US can help secure these benefits, which greatly exceed the costs, by coordinating a global effort to rapidly vaccinate the world against COVID-19."
6. Vaccine diplomacy: "Countries like China and Russia are using the pandemic to increase their strategic influence. These countries are utilizing COVID-19 vaccines as a tool of diplomacy to win friends and allies. China has exported nearly 350 million doses of its Sinovac and Sinopharm vaccines to countries across Asia, Latin America, Africa, and Europe. The US has shipped fewer than 24 million doses to 10 recipient countries. The US can counter China's rising influence by coordinating a global effort to rapidly vaccinate the world against COVID-19."

5) Specify exactly which analyses you will conduct to examine the main question/hypothesis.

The primary estimands of interest in this study are the average treatment effects for each of the five persuasive appeals, relative to the control condition. We will use linear regression of each outcome on treatment assignment, with robust standard errors, to estimate treatment effects. In addition to estimating unadjusted differences in means we will also estimate covariate-adjusted differences in means (using pre-treatment covariates to increase precision).

6) Describe exactly how outliers will be defined and handled, and your precise rule(s) for excluding observations.
Not applicable.

7) How many observations will be collected or what will determine sample size? No need to justify decision, but be precise about exactly how the number will be determined.
The study will be administered as part of a nationally representative survey on the NORC AmeriSpeak panel. This survey consists of approximately 4,000 U.S. adults (aged 18+).

8) Anything else you would like to pre-register? (e.g., secondary analyses, variables collected for exploratory purposes, unusual analyses planned?)
We will conduct secondary analyses to examine effect heterogeneity across sub-groups of respondents along the following dimensions: 1) nationalism/patriotism; 2) partisanship; 3) altruism; 4) ideology. These pre-treatment survey measures are based on existing scales that have been validated in prior studies.

S8 NORC/AmeriSpeak sampling methodology

TECHNICAL OVERVIEW OF THE AMERISPEAK® PANEL: NORC'S PROBABILITY-BASED HOUSEHOLD PANEL

Updated January 26, 2021

This technical overview provides the basic information about AmeriSpeak®, a large probability-based panel funded and operated by NORC at the University of Chicago. AmeriSpeak is designed to be representative of the U.S. household population, including all 50 states and the District of Columbia. U.S. households are randomly selected with a known, non-zero probability from the NORC National Frame as well as address-based sample (ABS) frames, and then recruited by mail, telephone, and by field interviewers face to face. AmeriSpeak panelists participate in NORC studies or studies conducted by NORC on behalf of governmental agencies, academic institutions, the media, and commercial organizations.

The construction of the AmeriSpeak panel started in 2014 with pilot samples. In 2015, about 7,000 households were recruited from a sample of around 60,000 addresses. In 2016, about 128,000 addresses were sampled to expand the panel to around 20,000 recruited households. About 51,000 addresses were selected for the 2017 recruitment, which led to the expansion of the regular AmeriSpeak panel to 23,000 recruited households. The AmeriSpeak Panel expanded to approximately 30,000 households in 2018 and 35,000 households in 2019 through further recruitment efforts. The 2020 recruitment is expected to expand the panel by another 5,000 households. The current panel size is 48,900 panel members age 13 and over residing in over 40,000 households.

In addition to the regular panel for general population studies, AmeriSpeak also contains sub-panels to support studies of special populations, including AmeriSpeak Latino, AmeriSpeak Teen, and AmeriSpeak Young Adult 18-34 (which features an oversample of African Americans, Hispanics, and Asians). AmeriSpeak is also the probability sample source for TrueNorth®, the NORC calibration solution for combining probability and non-probability samples for estimation through small area modeling that leverages data from AmeriSpeak, the American Community Survey, Current Population Survey, and other data sources for improved statistical efficiency.[1]

Panel Sample Frame

The primary sampling frame for AmeriSpeak is the 2010 NORC National Frame, a multistage probability sample that fully represents the U.S. household population. We provide a brief description of how the National Frame was constructed after the 2010 Census.

The primary sampling units (PSUs) in the first stage sample selection are 1,917 National Frame Areas (NFAs), each of which is an entire metropolitan area (made up of one or more counties), a county, or a group of counties with a minimum population of 10,000. A total of 126 NFAs are selected in the first stage, including 38 certainty NAFs, 60 urban NFAs, and 28 non-urban NFAs. The largest 38 NFAs, those with a population of at least 1,543,728 (0.5 percent of the 2010 Census U.S. population), were selected into the National Frame with certainty. The certainty NFAs consist of areas with high population density and are

[1] For more information about TrueNorth, see http://amerispeak.norc.org/our-capabilities/Pages/TrueNorth.aspx.

dominated by census tracts with city-style mailing addresses. These areas contain 56 percent of the U.S. household population while only accounting for about 8 percent of the geographic area of the United States. The remaining 1,879 NFAs are stratified into urban areas where city-style addresses predominate, and rural areas that are less likely to have city-style addresses. The latter stratum comprises 81 percent of the geographic area, but only 14 percent of the population. A sample of 60 and 28 NFAs is selected systematically from the urban and rural stratum, respectively, with selection probability proportional to size (PPS) where the measure of size is the number of housing units per NFA.

Within the 126 selected NFAs, the secondary sampling units (SSUs) are segments defined from Census tracts or block groups, where each segment contains at least 300 housing units according to the 2010 Census. Within the certainty NFAs, a sample of 896 segments was selected using systematic PPS sampling, where the size of a segment is the number of housing units. Implicit stratification was achieved by sorting the segments by location (NFA, state, and county), by principal city indicator, by ethnic and income indicators. From each urban and rural NFA, a sample of 8 and 5 segments was selected, respectively, using systematic PPS sampling where the measure of size is the number of housing units per segment. A total of 618 segments are selected from the non-certainty NFAs[2]. Overall, a stratified probability sample of 1,514 segments was selected into the National Frame in the second stage sampling.

Within the selected segments, all housing units are listed using the U.S. Postal Service Delivery Sequence File (DSF). In the 123 segments where the DSF coverage is deemed inadequate, the DSF address list is enhanced with in-person field listing to improve coverage. The final National Frame, consisting of all listed households in the sample segments, is estimated to provide over 97 percent coverage of the U.S. household population. It contains almost 3 million households, including over 80,000 rural households that are added through in-person listing. In addition to NORC's National Frame, the DSF is used as a supplemental sample frame in four states. Although nationally representative, the National Frame does not include households from Alaska, Iowa, North Dakota, and Wyoming. Since 2016, the annual panel recruitment sample has included a small address-based sample (ABS) from these four states to assure AmeriSpeak presence in in all U.S. States and Washington, D.C.

In 2017, an enhanced DSF frame was also used to develop a new Latino Panel with adequate representation of Spanish-language-dominant Hispanics. Census tracts with high incidence (at least 30%) of Spanish-dominant Hispanics were targeted for this recruitment. Furthermore, within these Census tracts, households that were flagged as Hispanic based on consumer vendor data (that are typically used for direct-mail marketing) were oversampled. This new AmeriSpeak Latino Panel contains approximately 5,400 Hispanic panelists with 24% of those panelists being Spanish-language dominant. As of February 2020, 63.4% of the recruited adults in the AmeriSpeak Panel were sourced from the National Frame, 27.1% from targeted ABS frames, and 9.5% from voter registration files[3]. Proper weights allow the full use of the combined sample.

Panel Sample Selection

For panel sample selection between 2014 and 2018, National Frame segments are stratified into six sampling strata based on the race/ethnicity and age composition of each segment, as below:

- Hispanic, high youth segments
- Hispanic, not high youth segments
- Non-Hispanic Black, high youth segments
- Non-Hispanic Black, not high youth segments
- Other, high youth segments

[2] A sample of 5 segments was selected from each of the 28 non-urban NFAs. However, 2 sample segments were later subsampled out in Montana due to cost.

[3] Voter registration files were used as supplemental sample source for the AmeriSpeak Young Adult Panel.

- Other, not high youth segments

Hispanic segments are those where Hispanics make up at least a third of the population and the Hispanic share in the population is greater than that of non-Hispanic Black. Similarly, non-Hispanic Black segments are those where non-Hispanic Black make up at least a third of the population and the non-Hispanic Black share in the population is greater than that of Hispanic. Finally, High Youth refers to segments in which 18-24 year old adults are at least 12% of the total adult population. The above stratification is used to oversample housing units in areas with higher concentration of young adults, Hispanics, and non-Hispanic African-Americans. The resulting household sample is referred to as the initial AmeriSpeak sample or sample for initial panel recruitment.

To support the second stage of panel recruitment, initially sampled but nonresponding housing units are subsampled for a nonresponse follow-up (NRFU)[4]. At this stage, consumer vendor data are matched to the pending housing units, and housing units that are flagged as having a young adult[5] (18-34 years of age) or minority (Hispanic[6], non-Hispanic Black[7]) are oversampled for the NRFU sample. Overall, approximately one in five initially nonresponding housing units are subsampled for NRFU using the same six sampling strata defined above. Due to NRFU, these initially nonresponding housing units have a much higher selection probability compared to the housing units that were recruited during the first stage of panel recruitment.

A 2-phase state-based ABS sample design was used for the 2019 AmeriSpeak recruitment. NORC's National Frame is designed to represent the U.S. household population nationally. At the state level, however, the panel may have more significant clustering effects from the use of the National Frame, especially for states with a small population. The primary objective of the 2019 design is to improve state-level representation by selecting the recruitment sample mostly from areas that are outside the National Frame. A stratified systematic sample was selected in the first phase, where each state constitutes a sampling stratum and the sample was allocated to the strata proportional to the square root of the state population. In the second phase, young adults, Hispanic, non-Hispanic Black, and conservatives are oversampled based on commercial data sources to improve their representation in the panel. Because the 2019 design did not use NRFU face-to-face recruitment, the 2019 design did not involve geographic clustering.

The overall AmeriSpeak Panel sample design reflects the cumulative design features of the NORC National Frame and the annual recruitment samples thus far. These design features are captured in the final panel weight for each recruited household and each panelist. There are mainly two reasons why the sampling design for AmeriSpeak Panel recruitment deviates from EPSEM (Equal Probability of Selection Method) sampling: (a) oversampling of housing units in segments with a higher concentration of young adults and minorities results in the sample selection probabilities being higher for housing units in these segments; and (b) NRFU subsampling results in initially nonresponding housing units having a much higher selection probability. Furthermore, within the NRFU samples, selection probabilities vary for housing units depending on the appended commercial data flags to target specific demographics for improved efficiency. The initial and NRFU sampling procedures are examined and possibly modified each year to more efficiently recruit subpopulations who are less likely to respond to the recruitment survey.

[4] A small fraction of initially nonresponding housing units are not eligible for NRFU, including "hard refusals" and those with an appointment for a call back from NORC.

[5] A young adult flagged household refers to a household where MSG or TargetSmart indicated there was an 18-24 year old adult in the household. In 2016 and 2017, a slightly different definition was used, and a young adult flagged household was defined as having an 18-34 year old adult in the household by MSG or 18-30 year old adult by TargetSmart.

[6] A Hispanic flagged household refers to a household where MSG or TargetSmart indicated the presence of a Hispanic adult in the household.

[7] A non-Hispanic Black flagged household refers to a household where MSG or TargetSmart indicated the presence of a non-Hispanic Black adult in the household.

Panel Recruitment Procedures

The highly efficient recruitment procedures set AmeriSpeak apart from other national panels. AmeriSpeak Panel recruitment is a two-stage process: (i) an initial recruitment using USPS mailings, telephone contact, and modest incentives, and (ii) a more elaborate NRFU recruitment using FedEx mailings, enhanced incentives, and in-person visits by NORC field interviewers.

For the initial recruitment, sample households are invited to join AmeriSpeak online by visiting the panel website AmeriSpeak.org or by calling a toll-free telephone line (in-bound/outbound supported). Both English and Spanish languages are supported for online and telephone recruitment. The initial recruitment data collection protocol features the following: an over-sized pre-notification postcard, a USPS recruitment package in a 9”x12” envelope (containing a cover letter, a summary of the privacy policy, FAQs, and a study brochure), two follow-up post cards, and contact by NORC’s telephone research center for sample units with a matched telephone number.

For the second-stage NRFU recruitment, a stratified random sample is selected from the nonrespondents of the initial recruitment. Units sampled for NRFU are sent a new recruitment package by Federal Express with an enhanced incentive offer. Meanwhile, NORC field interviewers make personal, face-to-face visits to the pending cases to encourage participation. Once the households are located, the field interviewers administer the recruitment survey in-person using CAPI or else encourage the respondents to register online or by telephone.

Panel Recruitment Response Rate and Other Panel Statistics

A sample household is considered recruited or responded if at least one adult in the household joins the panel. The weighted household response rate (AAPOR RR3) is about 6% for initial recruitment and 28% for NRFU recruitment. We report two recruitment response rates: one for all the panel recruitment years (2014-2019) and one for the recruitment years with NRFU (2014-2018). For all recruitment years, the cumulative weighted household response rate is 24.1%; for recruitment years with NRFU, and the cumulative weighted household response rate is 34.2%. For client studies requiring a panel recruitment response rate exceeding 30%, the sampling frame may be restricted to the panelists recruited in the NRFU years. The panel recruitment response rate calculation methodology is consistent with AAPOR guidelines and fully documented.[8] The annual panel retention rate is about 85%.

For individual client surveys based on the AmeriSpeak Panel, the AAPOR RR3 response rate is between 10% to 20% depending on specific study parameters such as target population, survey length, time in the field, salience of subject, and the like. This response rate takes into account panel recruitment rate, panel retention rate, and survey participation rate.[9]

Other important panel statistics with respect to the 2014-2019 recruited households are as follows: 62% are recruited in the initial stage and 38% are recruited via NRFU; 85% of the active panelists prefer to do web or online surveys, while 15% prefer to participate in telephone surveys; 21% of the recruited households are

---

[8] See http://amerispeak.norc.org/research/Pages/WhitePaper_ResponseRateCalculation_AmeriSpeak_2016.pdf

[9] A properly calculated cumulative AAPOR response rate for panel-based research takes into account all sources of non-response at each stage of the panel recruitment, management, and survey administration process (see https://www.aapor.org/AAPOR_Main/media/publications/Standard-Definitions20169theditionfinal.pdf, page 48-9). A common misapplication of the term “response rate” in online panel surveys is to represent the survey-specific cooperation rate as the “cumulative survey response rate.” *See* “Response Rate Calculation Methodology for Recruitment of a Two-Phase Probability-Based Panel: The Case of AmeriSpeak” authored by Robert Montgomery, J. Michael Dennis, N. Ganesh. The paper is available at https://amerispeak.norc.org/research/.

non-Internet[10]; 82% are cell-phone only or cell-phone mostly; 19% are African-American and 20% Hispanic; and 33% have household income below $30,000 (compared to CPS benchmark of 26%).[11]

Impact of Non-Response Follow-Up

NRFU is instrumental in producing the industry-leading response rate for AmeriSpeak Panel recruitment. Moreover, due to the more intensive effort, NRFU recruitments better represent hard to reach groups and are therefore more representative of the target population. For example, initial recruitment tends to under-represent young adults 18-34 years of age. NRFU recruitment corrects for this bias by bringing the age distribution of the panel closer to ACS benchmarks.

Overall, NRFU recruitment significantly improves the representation of the panel with respect to demographic segments that are under-represented among the respondents to the initial recruitment, including young adults (persons 18 to 34 years of age), African Americans, Hispanics, lower income households, renters, cell-phone only households, and persons with lower educational attainment (e.g., no college degree). To the extent that these demographic characteristics are correlated with substantive survey variables, NRFU helps to reduce potential non-response bias in the sample estimates. NORC's research indicates that NRFU respondents are indeed somewhat different from initial respondents for many common survey variables. For example, compared to the panelists recruited during the initial stage, NRFU panelists tend to be more conservative politically, more likely to attend church, less interested in current events or topics in the news report, less knowledgeable about science, less likely to be in favor of gun control policies, less likely to read a print newspaper (more likely to read the news online and use social media), more likely to eat at fast food restaurants and so on[12]. These observations illustrate that NRFU recruitment is critical for achieving a more balanced panel and for making the substantive estimates in AmeriSpeak studies more accurate. Even though NRFU panelists are more reluctant to complete surveys, the addition of NRFU panelists reduced total absolute bias on average 5 to21 percentage points when compared to the initial stage recruits (among examined surveys).[13]

Mixed-Mode Data Collection

The AmeriSpeak Panel supports mixed-mode data collection to improve response rate and the representativeness of the complete surveys. During the recruitment survey, AmeriSpeak panelists are offered an opportunity to choose their preferred mode—web or phone—for future participation in AmeriSpeak surveys. A recruited household can consist of both web- and phone-mode panelists residing in the same household. Panelists predominantly prefer web over phone mode. As of February 2020, 85% of the active panelists prefer to do web or online surveys, while 15% prefer to participate in telephone surveys. The telephone mode encompasses panelists without internet access, panelists whose only internet access is via a smartphone, and panelists with internet access but are unwilling to share an email address.

---

[10] The non-internet households (HHs) are those that do not select "High-speed, broadband internet at home (such as cable or DSL)" or "Dial-up internet at home" response options when they are asked "What kind of internet access do you have? Please select all that apply" item in the recruitment survey. The non-internet HHs include those that only use internet on a cell connection or mobile phone.

[11] For transparency purposes, unweighted percentages are presented in this section. Hence, these results do not take into account selection probabilities. The base weighted distributions that take into account selection probabilities can be provided upon request.

[12] *See* "The Undercounted: Measuring the Impact of 'Nonresponse Follow-up' on Research Data and Outcome Measures" authored by Ipek Bilgen, J. Michael Dennis, N. Ganesh. The paper will be soon available at https://amerispeak.norc.org/research/.

[13] *See* "Nonresponse Follow-up Impact on AmeriSpeak Panel Sample Composition and Representativeness" authored by Ipek Bilgen, J. Michael Dennis, N. Ganesh. The paper is available at https://amerispeak.norc.org/research/.

To the extent that non-internet households or "net averse" persons are different from the rest of the population, mixed-mode surveys have better population coverage and produce more accurate population estimates. NORC's telephone interviewers administer the telephone surveys using a data collection system supporting both the phone and web modes, providing an integrated sample management and data collection platform. For panelists using smartphones for web-mode surveys, the NORC survey system renders an optimized presentation of the survey questions for these mobile users.

Panel Management and Maintenance

Panel management and maintenance are crucial for panel health and efficiency. NORC maintains strict panel management rules to limit respondent burden, reduce panel attrition, and minimize the risk of panel fatigue. On average, AmeriSpeak panelists are invited to participate in client studies two to three times a month. AmeriSpeak works with NORC clients to create surveys that provide an appropriate user experience for AmeriSpeak panelists. AmeriSpeak will not field surveys that in our professional judgment will result in a poor user experience for our panelists. AmeriSpeak also has a designated website and a telephone number for panelist communications.

Panel maintenance is a dynamic process because the AmeriSpeak Panel is supplemented and refreshed regularly over time to grow the panel, compensate for panel attrition, and improve panel representation for specific subpopulations. For example, the Latino Panel and Teen Panel are created to support studies of Hispanics and teenagers, respectively; the 2019 recruitment is primarily designed to improve sample representation at the state level. As panelists are added or/and removed from the panel, the panel refreshment process takes place to ensure that the refreshed panel fully represents the corresponding target population.

ABOUT NORC AT THE UNIVERSITY OF CHICAGO

As one of the world's foremost independent research institutions, NORC at the University of Chicago delivers objective data and meaningful analysis to help decision-makers and leading organizations make informed choices and identify new opportunities. Since 1941, NORC has applied sophisticated methods and tools, innovative and cost-effective solutions, and the highest standards of scientific integrity and quality to conduct and advance research on critical issues. Today, NORC expands on this tradition by partnering with government, business, and nonprofit clients to create deep insight across a broad range of topics and to disseminate useful knowledge throughout society.

Headquartered in downtown Chicago, NORC works in over 40 countries around the world, with additional offices on the University of Chicago campus, the DC metro area, Atlanta, Boston, and San Francisco.

ADDITIONAL RESOURCES

To learn more about AmeriSpeak or to share an RFP, please contact AmeriSpeak at AmeriSpeak-BD@norc.org. Information about AmeriSpeak capabilities and research papers are available online at AmeriSpeak.NORC.org.
